# A NIR fluorotag reporter CETIF6a enables bright pan-tumor labeling and functional proteomic profiling

**DOI:** 10.1038/s41467-026-75356-3

**Published:** 2026-07-08

**Authors:** Jia Li, Feiran Zhang, Jianing Cheng, Xu Peng, Cheng Li, Chengbin Zhang, Yuewei Zhang, Songling Zhang, Shoujun Zhu

**Affiliations:** 1 Department of Obstetrics and Gynecology, The First Hospital of Jilin University, Changchun, PR China; 2 Joint Laboratory of Opto-Functional Theranostics in Medicine and Chemistry, The First Hospital of Jilin University, Changchun, PR China; 3 Changbaishan Laboratory, Jilin University, Changchun, PR China; 4 State Key Laboratory of Supramolecular Structure and Materials, Center for Supramolecular Chemical Biology, College of Chemistry, Jilin University, Changchun, PR China; 5 Department of Hepatobiliary and Pancreatic Surgery, General Surgery Center, The First Hospital of Jilin University, Changchun, PR China; 6 Department of Pathology, The First Hospital of Jilin University, Changchun, PR China

**Keywords:** Fluorescence imaging, Fluorescent probes, Cancer imaging, Target identification

## Abstract

Tumor-seeking fluorescent dyes enable precise lesion localization by recognizing overexpressed receptors, providing a critical adjunctive technology for cancer histopathology. However, tumor heterogeneity and the poor understanding of targeting mechanisms limit their efficacy. Here we engineer CETIF6a, a click chemistry-compatible heptamethine cyanine dye, for multi-cancer targeting, intraoperative histopathology, and proteome-wide target identification. CETIF6a demonstrates margin delineation across multiple cancer types ( > 90% concordance with H&E staining). Quantitative proteomics reveals that the dye targets 5-15 times more tumor-specific proteins than in paracancerous tissues. Synergistic pan-cancer targeting is achieved through 491 conserved tumor-enriched proteins involved in ribosomal, proteasomal, and metabolic pathways, effectively overcoming heterogeneity. Mechanistic studies confirm that CETIF6a emits bright fluorescence upon covalent binding to targets via nucleophilic substitution at cysteine thiol residues within hydrophobic cavities. The modifiable scaffold of CETIF6a supports both intraoperative tumor diagnosis and functional targets profiling, providing a foundation for systematic probe optimization.

## Abstract (web summary)

A click-compatible near-infrared dye, CETIF6a, enables multi-cancer labeling and proteome-wide target identification. It targets 491 conserved tumor-enriched proteins via covalent binding, overcoming tumor heterogeneity and supporting probe optimization.

## Introduction

The global cancer burden continues to grow^1,2^, and surgical resection remains the most established treatment for malignant solid tumors. Intraoperative rapid histopathological examination of resected tissue is essential to confirm complete tumor removal and improve postoperative outcomes. Conventional methods such as morphological analysis and immunohistochemistry face limitations including morphological ambiguity in some cases, subjective interpretation by pathologists, and considerable tumor heterogeneity. Recently, emerging tumor-seeking fluorescent dyes have shown great potential to overcome these constraints by enabling imaging-guided detection^3–7^.

Unlike conventional antibody-based probes, a specific class of near-infrared (NIR) heptamethine cyanine dyes have attracted considerable interest due to their notable photophysical properties and intrinsic tumor-seeking capability without the need for immunological recognition^8–11^. These dyes allow precise lesion localization through specific targeting of overexpressed receptors in tumors, providing a powerful fluorescence-based tool for cancer margin detection^12–19^. Among these, IR-6B3, a meso-chloro (Cl) and cyclohexenyl ring-functionalized heptamethine cyanine dye, has demonstrated strong tumor-targeting performance in preclinical studies^20–24^. Notably, IR-6B3 operates through a multi-target synergistic mechanism, which effectively reduced the loss of diagnostic sensitivity caused by tumor heterogeneity^25^. Further development and optimization of such multi-targeting fluorescent dyes could offer clinicians an efficient, low-cost method to greatly support clinical decision-making.

However, the molecular mechanisms governing the targeting specificity of these dyes remain poorly understood, impeding the broad application of novel pathological imaging techniques and hindering further probe optimization. Current research primarily emphasizes the improvement of photophysical properties^26–29^, with insufficient attention paid to systematic, proteome-wide profiling of interactions between dyes and targets. The inherent complexity of proteomes often results in low coverage in exhaustive studies and imposes high experimental costs. Moreover, substantial inter-sample heterogeneity in target protein expression contributes to elevated false-negative rates. Thus, there is a pressing need for a universal strategy that enables comprehensive and comparative investigation of probe targeting mechanisms across diverse clinical specimens.

To address these challenges, this study integrates activity-based protein profiling (ABPP), a robust platform, for functional proteome-wide analysis^30–34^. By engineering probe derivatives with orthogonal reactive groups and implementing click chemistry-mediated biotinylating strategies, ABPP enables systematic capture of probe targeted interactions across the entire proteome. We selected IR-6B3 as the model compound for probe engineering and carefully discussed whether it could retain tumor-targeting capability and compatibility with ABPP-oriented modifications. Through a modular probe design strategy, we constructed an IR-6B3 derivative library designated as click-enrichable tumor-specific in situ fluorescent tag (CETIF) series comprising different structural variants to achieve an enhancement in target protein enrichment efficiency while maintaining native tumor-seeking ability.

**Fig. 1:**
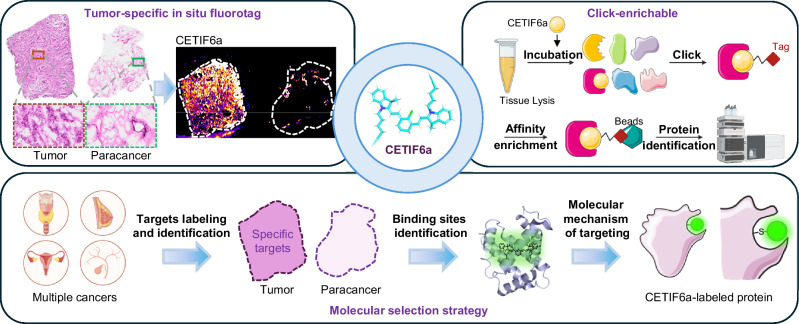
Schematic diagram illustrates the application of the dual-functional dye CETIF6a for rapid intraoperative tumor pathology detection and simultaneous exploration of its targeting strategy. In all figures of this work, if “Tumor (or T)” appears, it refers to a malignant solid tumor. Schematic diagram was designed using BioRender and FigDraw.

Based on this platform, we combined in situ histological staining with click chemistry to evaluate tumor margin delineation, target enrichment, and identification across multiple cancer types using the optimal candidate, CETIF6a (Fig. 1). Systematic mechanistic studies revealed a global landscape of all targets, and further analysis identified critical binding determinants. Through Foldseek-assisted structural profiling^35^, we deciphered principal structural and functional characteristics of target proteins and revealed CETIF6a’s molecular recognition preferences, thereby providing a rational basis for designing high-specificity probes.

## Results

### Engineering and characterization of CETIF with different click chemistry functional groups

**Fig. 2:**
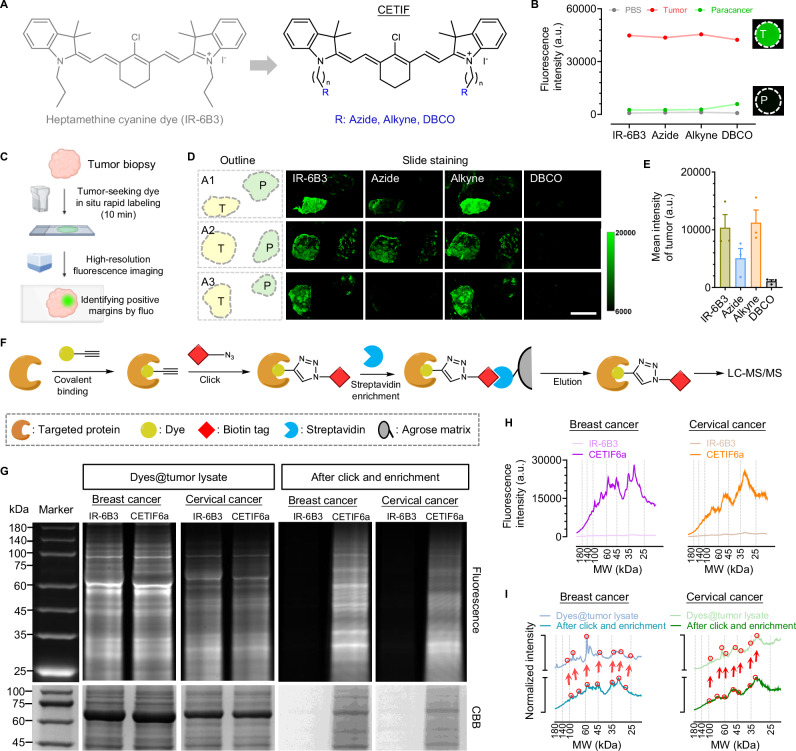
Design and characterization of CETIF. **A** CETIF was generated by introducing click chemistry moieties into IR-6B3. R denotes the incorporated click chemistry functional groups including Azide, Alkyne and DBCO, while n represents the number of carbon atoms in the side chain of CETIF. **B** Fluorescence intensity of the dyes in PBS and in protein lysates from clinical samples, with both the dyes and proteins at 10 µM (mean ± SEM, *n* = 3 samples per group). **C** Schematic diagram illustrating the principle of slide staining for intraoperative rapid detection of tumor margins using tumor seeking dyes. **D** CETIF-mediated slide staining distinguishes tumor tissues from adjacent paracancerous tissues in 3 clinical samples (the length of the scale bar: 5 mm). The contours of the tumors and the adjacent areas identified after H&E staining are presented using an outline. **E** Statistical data of mean fluorescence intensity of the tumor areas labeled by different dyes in (**D**) (mean ± SEM, *n* = 3 samples per group). **F** Schematic workflow of Alkyne labeling and enrichment for dye-targeted proteome identification. The graphical elements in the gray dashed box below annotate the representations of various symbols. **G** Representative analysis of CETIF6a’s capability to label and enrich proteins from clinical cancer tissue lysates (*n* = 3 independent experiment). CBB: Coomassie Brilliant Blue staining. **H** Fluorescence intensity of covalently bound complexes after click and enrichment detected by electrophoresis in SDS-PAGE gel. **I** Fluorescence intensity of covalently bound complexes before and after click. The intensity of the strongest fluorescence was normalized as 1.00. The similar fluorescence fluctuations before and after enrichment were marked with red circles, and the corresponding relationship was indicated by arrows. Schematic diagrams C and F were designed using BioRender and Chemdraw separately. Source data are provided as a Source Data file.

Given the superior photophysical properties of heptamethine cyanine dye IR-6B3 with meso-Cl and cyclohexenyl ring in cancer margins imaging applications, we preserved its core structure as a scaffold to synthesize a CETIF probe library featuring click chemistry compatible functional groups (Fig. 2A). Modifications were focused on the side chains, where click chemistry moieties including azide, alkyne or dibenzo cyclooctyne (DBCO) were introduced (structural formulas were shown in Supplementary Fig. 1A). Derivatives functionalized with azide, alkyne, and DBCO groups were respectively designated as Azide, Alkyne, and DBCO.

In vitro characterization of the dyes’ photophysical properties was performed. All dyes retained absorption peaks near 800 nm and emission peaks around 830 nm (Supplementary Fig. 1B, C), indicating that side-chain modifications and functional group additions did not compromise their NIR spectral properties. To assess the impact of structural modifications on the tumor labeling capability of CETIF, we further evaluated its brightness using clinical cervical tumor (T) and adjacent non-tumor (paracancerous, P) tissue samples whose hematoxylin & eosin (H&E) staining result was shown in Supplementary Fig. 1D for reference. We examined the fluorescence intensity of the dyes in protein lysates derived from both T and P tissues (Fig. 2B). All probes, including the parent compound IR-6B3, exhibited minimal fluorescence in aqueous environments, consistent with the inherent hydrophobicity of cyanine dyes. Conversely, they demonstrated significantly enhanced brightness in T tissue protein lysates. The modified probes exhibited comparable or slightly increased fluorescence intensities relative to IR-6B3, which all stood in sharp contrast to the weak fluorescence observed in P tissue lysates. SDS-PAGE analysis of the dye@tissue protein lysate complexes revealed the formation of fluorescent complexes via covalent binding (Supplementary Fig. 1E–G).

Subsequently, we evaluated the ability of CETIF to specifically label tumors under rapid detection conditions. Based upon reported methodology for rapid intraoperative pathological detection via tissue section staining (Fig. 2C), we investigated tumor margin delineation capabilities of dyes. Using IR-6B3 as a positive control and H&E staining as the diagnostic standard (Supplementary Fig. 1H), we evaluated the tumor margin outlining performance of CETIF derivatives. Tissue section slides staining assays performed across 3 clinical specimens demonstrated that the Alkyne derivative, similar to IR-6B3, selectively stained tumor tissues through direct incubation within 10 min (Fig. 2D). Its brightest intensity in tumor regions and the significant fluorescence intensity difference between T and P tissues confirmed its diagnostic utility (Fig. 2E). In contrast, the tissues stained with Azide and DBCO had lower brightness. Among them, DBCO exhibited minimal staining of both T and P tissues, indicating that the steric bulk of the DBCO group impedes access to protein under rapid detection conditions. These findings conclusively established Alkyne as a reliable candidate for subsequent research.

### Evaluation of CETIF with different side chain lengths for target protein labeling and enrichment

Considering that the length of the dye’s side chain might influence the solvent accessibility of the alkyne group within the dye@protein complex, we systematically adjusted the side chain length to investigate its impact on both imaging performance and functional group reactivity. Derivatives featuring side chains of Alkyne with 4–6 carbons were designated as CETIF4a, CETIF5a, and CETIF6a (corresponding to the previously mentioned Alkyne), respectively (Supplementary Fig. 2A).

We first characterized the spectral properties (Supplementary Fig. 2B, C) and specific tumor protein labeling capability of CETIF4a/5a/6a (Supplementary Fig. 2D–F). Consistent with the first generation CETIF and using the same cervical cancer specimen as before, the CETIF4a/5a/6a series exhibited high fluorescence intensity upon covalent binding to T tissue lysates, achieving approximately 10-fold higher brightness compared to P tissues lysates. Consistent results were observed in other tumor types (Supplementary Fig. 2G, H for breast cancer, Supplementary Fig. 2I, J for thyroid cancer), collectively validating the enhanced tumor labeling intensity of CETIF4a/5a/6a across all 3 cancer types. We further evaluated the performance of CETIF4a/5a/6a in rapid intraoperative section staining applications (Supplementary Fig. 2K). All probes clearly highlighted T tissues. Among them, CETIF4a yielded the highest fluorescence intensity in T regions (Supplementary Fig. 2L), followed by CETIF6a and CETIF5a. However, CETIF4a also exhibited higher background fluorescence in P tissues, resulting in diminished T/P contrast (Supplementary Fig. 2M). Quantification revealed that CETIF6a achieved the highest T/P ratio (IR-6B3: 3.89 ± 1.56, CETIF4a: 2.43 ± 0.27, CETIF5a: 2.55 ± 0.68, CETIF6a: 3.66 ± 1.19), indicating superior imaging contrast.

**Fig. 3:**
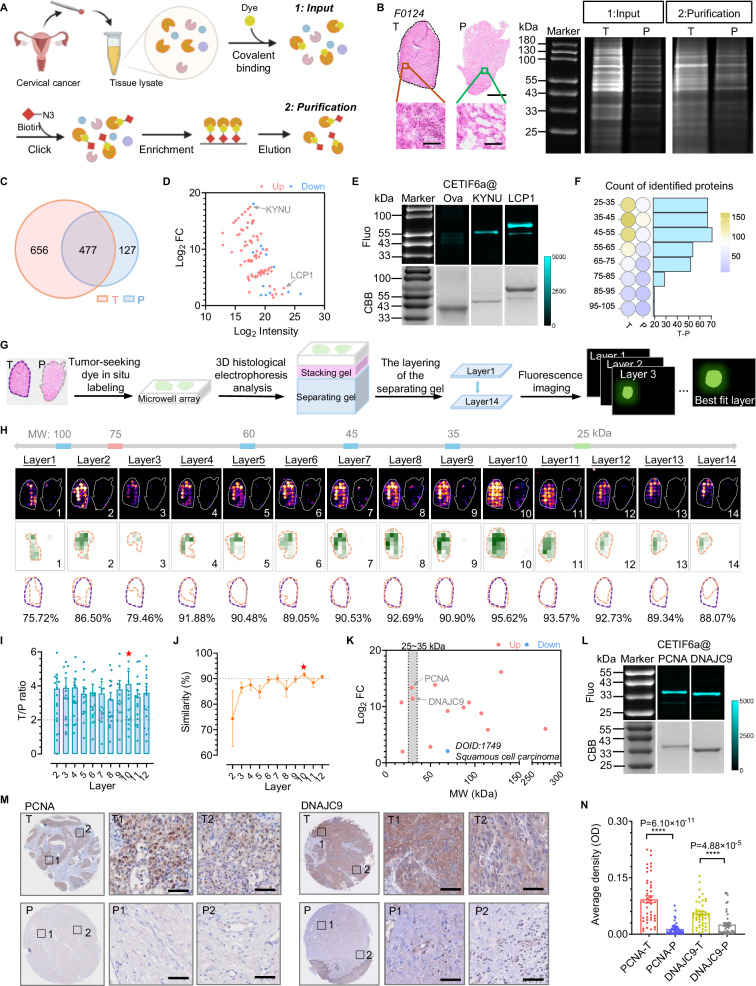
Quantitative profiling of CETIF6a-targeted proteome in cervical squamous carcinoma. **A** Schematic workflow of CETIF6a labeling and enrichment for identifying the targeted proteome in CSC. **B** Proteome labeled (input) and enriched (purification) following the workflow in (**A**) (*n* = 5 independent replicates with consistent results). Scale bars: 1 mm (1 × H&E), 100 μm (20 × H&E). **C** Venn diagram of upregulated target proteins identified in the T group versus the P group across 5 CSC samples. **D** Scatter plot of intensity versus fold change (FC) for targeted proteins. Two candidates are highlighted. **E** Fluorescence intensity of CETIF6a@KYNU and LCP1 (*n* = 3 independent replicates with consistent results). CBB: Coomassie Brilliant Blue staining. **F** Molecular weight distribution of upregulated target proteins across 5 CSC samples. **G** Schematic diagram illustrates the principle of 3D histological electrophoresis. **H** Imaging results of layers 1-14 in 3D histological electrophoresis for F0124. Regions outlined by purple and orange dashed lines correspond to tumor areas defined by H&E staining and 3D histological electrophoresis. **I**, **J** Pooled statistical analysis of T/P ratios and similarity across layers 2-12. The layer with the highest average value is marked with a red pentagram (mean ± SEM, n = 5 samples, all samples were analyzed with 3 replicates). **K** Scatter plot of molecular weight versus fold change (FC) for targeted proteins highly correlated with squamous cell carcinoma. Two candidates (PCNA and DNAJC9) are highlighted. **L** Fluorescence intensity of Dye@protein complexes of CETIF6a@PCNA and DNAJC9. (*n* = 3 independent replicates with consistent results). **M** The typical immunohistochemical patterns of PCNA and DNAJC9 proteins in clinical samples of CSC. Scale bars: 50 μm, *n* = 15 samples per group. **N** Statistical results of immunohistochemical intensities for 15 samples (mean ± SEM, *P* = 6.10 × 10^-11^ & 4.88 × 10^-5^, paired t-test, two tailed). Schematic diagram A and G were designed using BioRender and Microsoft PowerPoint separately. Source data are provided as a Source Data file.

To specifically enrich target proteins, we employed copper catalyzed click chemistry to conjugate biotin-azide (Biotin-PEG4-Azide, structural formulas were shown in Supplementary Fig. 3A) tags to the dyes@proteins as outlined in Fig. 2F. We validated labeling and enrichment efficiency using purified protein (human serum albumin, HSA) and complex biological systems. CETIF6a achieved the highest enrichment efficiency of HSA across 3 independent replicates, outperforming CETIF4a and CETIF5a (Supplementary. Fig. 3B, C, relative CBB intensity normalized to DMSO = 1.00, IR-6B3 = 0.44 ± 0.22, CETIF4a = 14.79 ± 2.21, CETIF5a = 14.72 ± 4.86, CETIF6a = 21.80 ± 4.46), and enriched 2.45-fold and 1.41-fold more target proteins from mouse tissue proteins than CETIF4a and CETIF5a, respectively (Supplementary Fig. 3D, E, average CBB intensity of CETIF4a = 2764.07, CETIF5a = 4806.30, CETIF6a = 6769.02).

The results of the aforementioned tests collectively indicate that CETIF6a is the optimal dye choice. Finally, we tested the ability of CETIF6a to enrich its targets in human clinical tumor samples. Using CETIF6a and streptavidin-agarose resin (optimized concentration: 40 μL/mL, Supplementary Fig. 4), we affinity-purified biotinylated CETIF6a@proteins from clinical cervical and breast cancer samples (Fig. 2G). In both tumor types, CETIF6a successfully enriched target proteins, unlike IR-6B3 which lacked enrichment capability (Fig. 2H). Furthermore, the protein species profile remained consistent before and after enrichment, indicating no significant disruption (Fig. 2I). These findings conclusively establish CETIF6a as the definitive dual functional probe enabling protein-level resolution of its imaging specificity.

### Quantitative chemoproteomic profiling of dye target proteome in cervical cancer

To investigate the molecular mechanism behind the tumor seeking capability of heptamethine cyanine-based dyes, we conducted a systematic enrichment and quantitative analysis of their target proteome in T versus P tissues using CETIF6a technology. Using 5 cervical squamous cancer (CSC) specimens as representative models, our experimental workflow (Fig. 3A) demonstrated effective preservation of labeled proteins after purification, as confirmed by fluorescence imaging of SDS-PAGE gels (representative sample F0124 shown in Fig. 3B, with remaining samples presented in Supplementary Fig. 5).

Using a fold change (FC) threshold > 2, LC-MS/MS analysis identified 1,133 T upregulated and 604 P upregulated proteins across all specimens. Comparative profiling revealed 477 shared proteins, along with 656 T-exclusive and 127 P-exclusive targets (Fig. 3C). These findings demonstrate that CETIF6a exhibits superior binding specificity for tumor associated proteins, with T-exclusive targets being 5-fold more abundant than their P counterparts. This obvious disparity enables CETIF6a to serve as an effective fluorescent marker for delineating tumor margins through specific protein labeling.

To reduce the impact of inter-patient tumor heterogeneity, we intersected target proteins across the 5 specimens. Intersection analysis identified 89 T upregulated and 16 P upregulated proteins common to all samples (Supplementary Fig. 6A-D). Scatter plots of their abundance and FC values (Fig. 3D) revealed that two extreme value targets, kynureninase (KYNU, extreme FC) and plastin-2 (LCP1, extreme intensity), were not outliers. This suggested that CETIF6a-mediated tumor visualization arises from the synergistic effect of multiple targets rather than a single dominant biomarker. To further validate target specificity, we cross-referenced the 89 tumor-associated proteins with a public single cell transcriptomic dataset (11,289 cervical cancer cells, 9649 adjacent normal cells, Supplementary Fig. 6E)^36^. T upregulated targets were mainly expressed in malignant and immune cells, while P targets localized to fibroblasts and stromal cells. Disease Ontology (DO) enrichment analysis of the 89 tumor proteins (Supplementary Fig. 6F) revealed strong associations with cancer-related pathways, including squamous cell carcinoma, colorectal cancer, and intestinal cancer. These results verify the accuracy of our proteomic findings against larger external datasets, confirming that CETIF6a-labeled targets overcome tumor heterogeneity.

Functional validation of these targets was performed using recombinant proteins of representative candidates (KYNU and LCP1), with minimal-binding ovalbumin (Ova) as a control. Co-incubation of CETIF6a and recombinant proteins confirmed covalent binding and bright fluorescence via SDS-PAGE (Fig. 3E).

To identify the key targets responsible for CSC demarcation, we performed molecular weight-based stratification of the identified proteins. Distribution analysis (Fig. 3F) revealed T-enriched proteins predominated across all mass ranges (10 kDa intervals). Inspired by the principles of three-dimensional (3D) histological electrophoresis (Fig. 3G)^21–23^, we systematically evaluated the fluorescence intensity and tumor margin delineation accuracy for each molecular weight subgroup. Using preserved tissue sections from the same 5 CSC specimens, we conducted 3D electrophoretic separation followed by fluorescence imaging (with F0124 as a representative example in Fig. 3H, other samples were shown in Supplementary Fig. 7A). The results demonstrated distinct spatial distribution patterns of labeled targets across different molecular weight layers (layer 1-14). Quantification of T/P fluorescence ratios (threshold > 2, Supplementary Fig. 7B, C) confirmed specific tumor localization. Following exclusion of error-prone edge layers (positions 1, 13, and 14), aggregate analysis of all specimens identified layer 10 (25-35 kDa) as exhibiting optimal T/P ratio and margin delineation accuracy (Fig. 3I, J).

Following the identification of the optimal molecular weight range (25-35 kDa, Layer 10), we prioritized 2 CETIF6a targets within this range that were implicated in squamous cell carcinoma pathways, including Proliferating Cell Nuclear Antigen (PCNA, associated with cell proliferation) and DnaJ Homolog Subfamily C Member 9 (DNAJC9, involved in protein transcription and folding) (Fig. 3K). Based on rigorous analysis, we identified PCNA and DNAJC9 as highly promising CETIF6a targets for CSC clinical samples. SDS-PAGE confirmed the covalent binding of both PCNA and DNAJC9 to CETIF6a, resulting in bright fluorescence emission (Fig. 3L). Immunohistochemical analysis of 15 specimens robustly validated PCNA and DNAJC9 as key biomarkers for CSC (Fig. 3M, N). The results indicated these targets exhibited ubiquitous expression across tumor regions in all patient samples and markedly lower expression in P tissues. Collectively, these targets enable CETIF6a to specifically bind tumor tissue, forming bright tumor-specific labels that accurately delineate tumor margins during detection.

### Molecular mechanism of CETIF6a targeting in cervical cancer

After identifying key targets of the dye, we further investigated the molecular mechanism of its covalent binding to explain complex formation, understand its targeting mode, and provide insights for future dye optimization. Molecular docking predicted binding sites for KYNU, LCP1, PCNA, and DNAJC9 with CETIF6a. Analysis of the electrostatic potential distribution within these binding pockets revealed predominantly hydrophobic environments, with cysteine residues identified as critical sites for covalent bond formation (Supplementary Fig. 8).

**Fig. 4:**
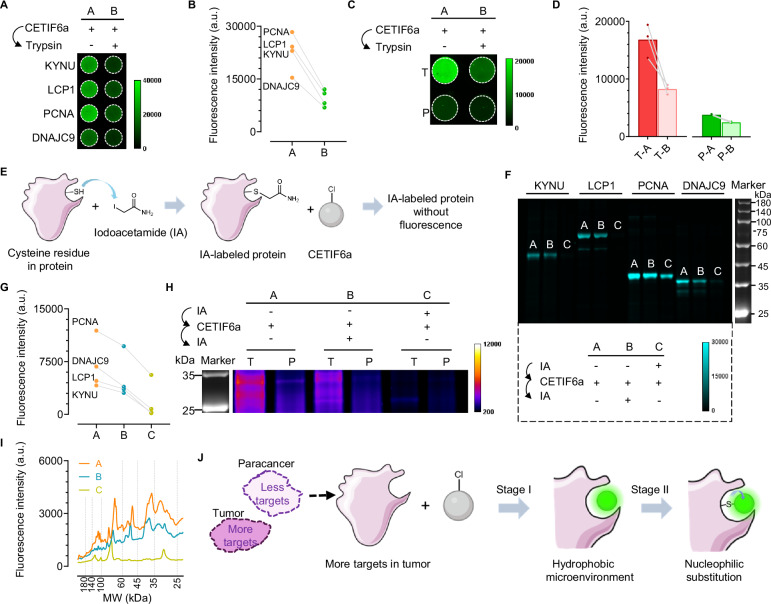
The binding mechanism of CETIF6a with the targets. **A** The brightness changes of the CETIF6a@targets after treatment with trypsin (*n* = 3 independent experiment). **B** Fluorescence intensity analysis of the orifice plate in (**A**). **C** The brightness changes of the CETIF6a@tissue proteins after treatment with trypsin (*n* = 3 independent experiment). **D** Fluorescence intensity analysis of the orifice plate in (**C**). **E** Schematic of the competition assay using iodoacetamide for CETIF6a binding sites on targets. **F** Validation of accessible cysteine residues for CETIF6a-target binding using recombinant key target proteins in CSC (*n* = 3 independent experiment). **G** Quantitative comparison of band fluorescence intensity in (**F**). **H** Essentiality of accessible cysteines for CETIF6a binding verified with clinical tissue lysates from CSC samples (*n* = 3 independent experiment). **I** Comparative fluorescence intensity of T-group bands in (**H**). **J** Proposed molecular mechanism of covalent binding between CETIF6a and targets. Schematic diagram E and J were designed using BioRender. Source data are provided as a Source Data file.

To test whether the protein microenvironment within the binding pocket sustains fluorescence enhancement, we disrupted the protein structure of pre-formed dye@protein complexes (incubated 2 h at RT) using trypsin digestion. NIR fluorescence decreased upon destruction of the binding cavity, observed for all recombinant target proteins (Fig. 4A, B) and tissue lysates (Fig. 4C, D). This confirms that the structural integrity of the binding pocket is essential for maintaining probe brightness.

For experimental validation of cysteine-dependent covalent binding, competitive binding assays using iodoacetamide (IA), a non-fluorescent alkylating agent, were performed (Fig. 4E). Free thiols can be alkylated by IA, thereby preventing their covalent reaction with CETIF6a. After CETIF6a incubation allowed covalent complex formation, subsequent IA treatment (1 h, 37 °C) caused minimal fluorescence reduction (SDS-PAGE, lane B, Fig. 4F–I). However, the pre-treatment of proteins with iodoacetamide (IA) to block thiol groups prior to the addition of CETIF6a resulted in a significant reduction or complete elimination of fluorescence (SDS-PAGE, lane C, Fig. 4F–I). These results confirmed that covalent binding requires accessible free cysteine thiols. CETIF6a (and related meso-Cl heptamethine cyanine dyes like IR-6B3) acts as an organic halide, whose meso-Cl is susceptible to nucleophilic substitution by cysteine thiols, forming a thioether bond.

Based on computational and experimental evidence, we propose the binding mechanism depicted in Fig. 4J. In stage I of the binding, CETIF6a was guided into the target’s hydrophobic pocket via supramolecular interactions. The hydrophobic microenvironment restricted intramolecular rotation or vibration, reducing non-radiative decay and enhancing fluorescence. In stage II, a nucleophilic cysteine thiol attacked the meso-Cl, forming a stable thioether bond. The protein scaffold shields the dye from solvent quenching and aggregation-induced quenching, further enhancing brightness and complex stability.

### Interpretation ability of CETIF6a in the histological detection of different types of tumors

**Fig. 5:**
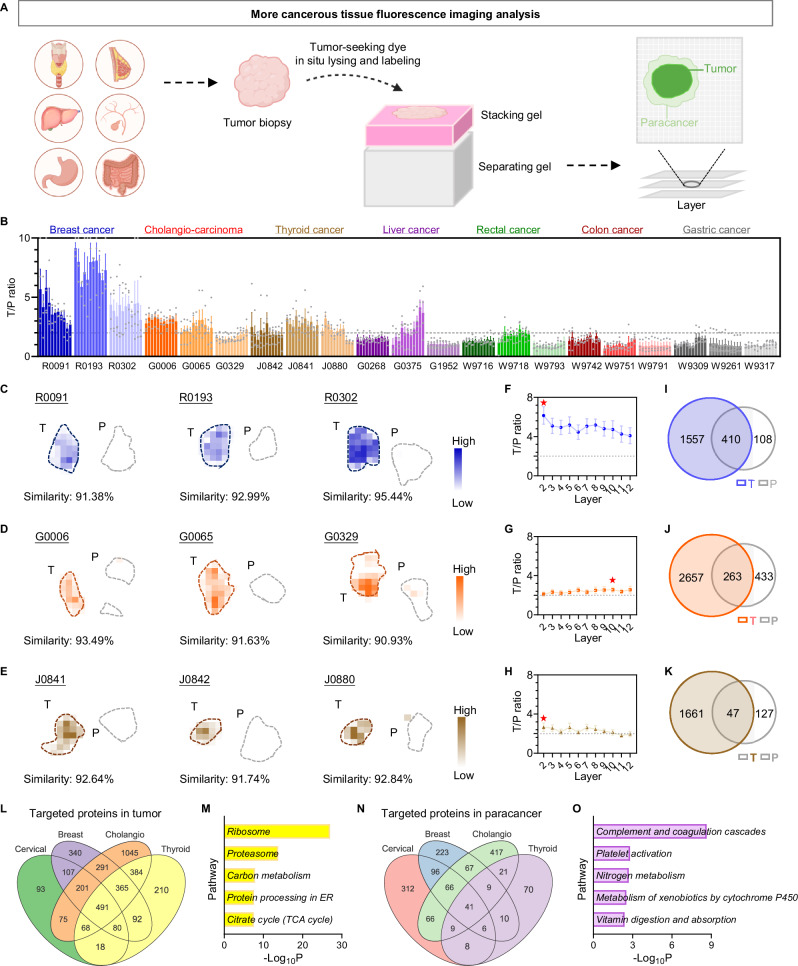
CETIF6a-mediated 3D histological electrophoresis for tumor margin detection in diverse solid tumors. **A** Schematic diagram of the workflow for testing the ability of CETIF6a to identify different types of clinical tumor samples. **B** Statistical analysis of T/P ratios across layers 2-12 for each sample. A cutoff value of 2 is indicated by a gray dashed line. T/P ratio greater than 10.00 are displayed as 10.00 (mean ± SEM, *n* = 3 samples per cancer type, all samples were analyzed with 3 replicates). **C–E** Tumor margin concordance of breast cancer (**C**), cholangiocarcinoma (**D**) and thyroid cancer (**E**). (**F–H**) Pooled T/P ratios across layers 2-12 for breast (**F**), cholangio (**G**) and thyroid (**H**) cancer samples (mean ± SEM, *n* = 3 samples per cancer type, all samples were analyzed with 3 replicates). A cutoff value of 2 is indicated by a gray dashed line. Red pentagrams: layers with peak values. **I–K** Venn diagram of upregulated target proteins identified in the T group versus the P group across breast (**I**), cholangio (**J**), and thyroid cancers (**K**) (for breast, cholangio and thyroid cancer, *n* = 5, 5 and 3 samples). **L** Venn diagram of upregulated tumor-enriched proteins across 4 cancer types. **M** Functional annotation of 491 overlapping targets from (**L**). **N** Venn diagram across 4 cancer types of upregulated targets in paracancerous tissues. **O** Functional annotation of 41 shared targets from (**N**). Schematic diagram A was designed using BioRender and FigDraw. Source data are provided as a Source Data file.

To assess tumor-discrimination capability of CETIF6a beyond CSC, we analyzed clinical specimens from 7 types of clinical tumors (each represented by 3 patient-derived samples, Fig. 5A). For each specimen, tumor margin delineation was tested using 3D histological electrophoresis (*n* ≥ 3).

In 3D histological electrophoresis, tissue proteins were resolved across 14 layers. Following exclusion of error-prone edge layers (positions 1, 13, and 14), T/P fluorescence ratios for layers 2-12 were quantified (Fig. 5B). This technique successfully demarcated tumor margins in all tested breast cancer (BC), cholangiocarcinoma (CCA), and thyroid cancer (TC) specimens using the diagnostic threshold established for CSC (T/P ratio > 2). Partial discrimination was achieved in subsets of hepatocellular carcinoma (HCC) and gastric cancer (GC) samples, while colorectal cancer (CRC) exhibited limited diagnostic accuracy.

For cancers demonstrating effective discrimination (BC, CCA, TC), comparative analysis against H&E-stained sections confirmed > 90% concordance in margin delineation (Fig. 5C–E and H&E datasets in Supplementary Fig. 9). Further stratification analysis by layer revealed cancer-specific optimal discrimination zones, such as layer 2 (Fig. 5F, H, and MW range: 75–100 kDa) exhibited the highest T/P ratios in BC and TC, layer 10 (Fig. 5G, MW range: 25–35 kDa) demonstrated optimal discrimination in CCA.

By isolating target proteins within specific mass ranges, this method overcomes the signal overlap inherent in conventional slide-staining imaging. This layer-specific efficacy highlights CETIF6a’s multi-target capability, enabling it to report key cancer-specific antigens across diverse tumor types.

### Further profiling of pan-cancer targets in various types of tumors

To identify pan-cancer targets of CETIF6a and facilitate its optimization, we profiled its protein targets in BC, CCA and TC specimens (*n* ≥ 3 per type) using the established CSC target identification workflow. An analysis of the union of targets across patients (Fig. 5I–K) revealed that, consistent with the findings from CSC results, T tissues consistently exhibited significantly higher numbers of upregulated proteins bound by CETIF6a compared to P tissues in all cancer types. This confirms the probe’s capacity to covalently report a diverse set of T-enriched proteins, whose collective abundance and diversity underlie fluorescence-based tumor delineation.

Intersection analysis of tumor-specific targets across the 4 cancer types (CSC, BC, CCA, TC, Fig. 5L) identified 491 proteins commonly and significantly upregulated. KEGG pathway enrichment analysis of these shared targets (Fig. 5M) revealed significant enrichment in 5 core pathways (Ribosome, Proteasome, Carbon metabolism, Protein processing in endoplasmic reticulum and Citrate cycle). These enriched pathways collectively depict a cellular state characterized by heightened proliferation, hyperactive metabolism, significant cellular stress, enhanced adaptive and survival capacity as hallmarks of malignant transformation. To further elucidate the biological relevance of these enriched pathways in the context of cancer biology, we mapped them onto the Hallmarks of Cancer framework proposed by Hanahan and Weinberg^37,38^. The Ribosome pathway supports sustained proliferative signaling by facilitating the elevated protein synthesis required for rapid cell division. The Proteasome pathway, which governs protein degradation and turnover, is critically linked to genome instability and mutation. The pathway enables cancer cells to tolerate the accumulation of aberrant or misfolded proteins resulting from oncogenic stress, thereby promoting survival. Carbon metabolism and the citrate cycle are key components of reprogrammed energy metabolism, a hallmark that allows cancer cells to adapt their metabolic fluxes to meet the biosynthetic and energetic demands of proliferation. Lastly, protein processing in the endoplasmic reticulum contributes to resistance to cell death by alleviating ER stress and maintaining cellular survival under unfavorable conditions. Collectively, these pathways converge on a coordinated set of hallmark capabilities that are fundamental to malignant transformation and maintenance. Thus, the protein networks captured by CETIF6a not only reflect the molecular landscape of tumor tissues but also directly engage with core oncogenic processes, providing a mechanistic basis for the probe’s robust pan-cancer labeling performance.

The analysis of paracancerous targets identified only 41 proteins common to all 4 cancer types (Fig. 5N), accounting for merely 8% of the total number of shared tumor targets. KEGG analysis of these targets (Fig. 5O) linked them to pathways including complement and coagulation cascades, platelet activation, nitrogen metabolism, xenobiotic metabolism by cytochrome P450 and vitamin digestion and absorption. These pathways primarily reflect tumor microenvironment interactions and systemic metabolic processes, showing minimal association with core tumor cell autonomous malignancy. Collectively, these results demonstrate that CETIF6a reports conserved protein networks across 4 cancer types, revealing common features in tumor evolution. This ability to stably capture pan-cancer signatures provides a molecular foundation for developing CETIF6a as a single dye for multi-cancer diagnostic and therapeutic strategies.

Notably, CETIF6a exhibited suboptimal pan-cancer labeling efficacy in HCC and gastrointestinal cancers. The analysis indicated that the G1952 specimen exhibited the lowest level of discrimination among the 3 HCC samples, with its adjacent non-tumor tissue presenting cirrhotic pathology, suggesting an inadequate distinction between tumor-associated proteins and those associated with cirrhotic damage. Furthermore, we speculate that normal liver tissue inherently exhibits a greater diversity and abundance of target proteins, which may lead to increased target availability in P tissues and consequently higher background signal.

### Structural and functional clustering of CETIF6a targets

While our results demonstrate CETIF6a’s ability to serve as a tumor-specific fluorescent label for highly expressed proteins, the huge number and functional diversity of these targets need systematic classification. Grouping targets by function would clarify their collective value and guide targeted structural optimization of CETIF6a for specific biological applications.

**Fig. 6:**
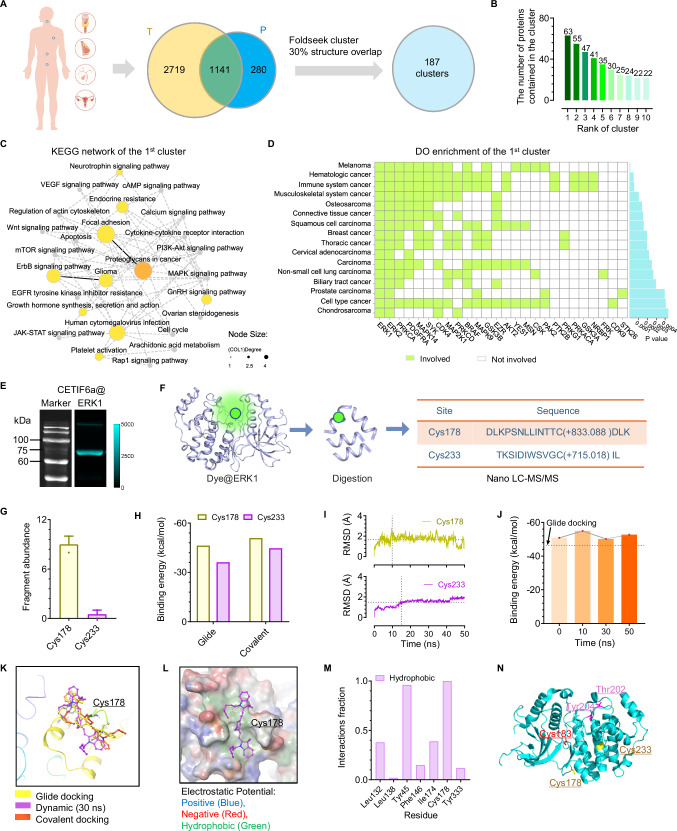
Structural clustering and covalent binding mechanism of CETIF6a-targeted tumor markers. **A** Foldseek-based structural clustering of target proteins (30% similarity threshold). **B** Statistical analysis of protein abundance in the top 10 clusters with the highest protein content post-clustering. **C** KEGG pathway network of 63 proteins within the top-ranked cluster. Core pathway is highlighted in orange. **D** Disease Ontology (DO) pathway enrichment analysis of 63 proteins within the top-ranked cluster. Cancer-related pathways in the top 20 pathways ranked by corrected-p value were shown. Proteins involved in the pathways were marked in green. **E** Fluorescence intensity of CETIF6a@ERK1 with GST tag complexes (*n* = 3 independent experiment). **F** CETIF6a modification sites on ERK1. **G** Fragment ion abundance for modified residues Cys178 and Cys233 (mean ± SEM. *n* = 4 samples). **H** Docking binding energies for CETIF6a complexes with ERK1-Cys178/233. **I** Complex stability analysis. RMSD evolution during initial 50 ns MD simulation. **J** Binding energies profile for covalent complexes during the initial 50 ns of the combination between CETIF6a and ERK1-Cys178. **K** Binding cavity conformational transition from non-covalent docking to stable covalent state. **L** The electrostatic potential distribution within CETIF6a-ERK1-Cys178 binding cavity. **M** CETIF6a-residue interaction profiling of key contacts with ERK1 amino acids. **N** Spatial mapping of functional (Cys183, Thr202, Tyr204) and modification sites (Cys178, Cys233) on ERK1. Schematic diagram A was designed using FigDraw. Source data are provided as a Source Data file.

Given that structure determines function, elucidating the major biological roles of CETIF6a targets requires a clear understanding of their structural features. We therefore performed comprehensive structural analyses. We compiled intersecting proteins across all cases, followed by a union analysis across cancer types. For structural clustering, we employed Foldseek to cluster these proteins based on structural similarity (30% overlap threshold, singleton clusters excluded), yielding 187 distinct structural clusters (Fig. 6A).

Analysis of the top 10 clusters by member count (Fig. 6B, representative cluster structures in Supplementary Fig. 10) revealed remarkable functional convergence. Notably, the largest cluster (63 structurally related proteins) exhibited strong functional associations with Proteoglycans in cancer and Focal adhesion pathways via pathway interaction network analysis (Fig. 6C). These pathways are critically involved in tumor signal transduction, cell migration, and invasion. DO enrichment analysis of this 63-protein cluster further demonstrated its profound link to oncogenesis. More than 80% of the top 20 enriched pathways were cancer-related (e.g., melanoma, hematologic malignancies, Fig. 6D). These converging lines of evidence, such as structural clustering, pathway enrichment, and disease association strongly support the direct involvement of proteins within this dominant cluster in tumor signaling and metastasis.

To explain the binding mechanism of CETIF6a to targets involved in tumor signaling and metastasis, we selected Extracellular Signal-Regulated Kinase 1 (ERK1), a functionally and structurally representative protein from cluster 1 for detailed investigation. Fluorescence assays confirmed covalent binding between CETIF6a and ERK1 (Fig. 6E). Proteolytic digestion of the complex followed by mass spectrometry identified Cys178 and Cys233 as the specific modification sites on ERK1 (Fig. 6F). For comparative analysis of binding sites, we tested the site preference. Quantification of MS/MS spectral counts (Fig. 6G, Supplementary Fig. 11) revealed higher spectral resolution for Cys178, indicating greater confidence in its identification as a binding site. Molecular docking (MD) calculated lower binding energies for both non-covalent and covalent interactions at Cys178 compared to Cys233 (Fig. 6H) indicating stronger affinity between Cys178 and dye. Molecular dynamics simulations demonstrated faster complex stabilization at Cys178 (~10 ns) versus Cys233 (~15 ns), based on RMSD convergence (Fig. 6I). Collectively, these results identify Cys178 as the primary CETIF6a binding site on ERK1.

MD simulations visualized the CETIF6a binding process to ERK1-Cys178 (Fig. 6J, K). We observed covalent bond formation with the Cys178 thiol group and minor rotational adjustments of the dye preceded stable complex formation, reflected in transient fluctuations of binding energy. Stable positioning within a hydrophobic binding pocket (Fig. 6L, M) was critical for maintaining dye brightness and complex stability. Similar binding dynamics were observed for Cys233 (Supplementary Fig. 12). These findings confirm the binding mechanism proposed in Fig. 4J. ERK1, the terminal kinase in the oncogenic RAS-RAF-MEK-ERK pathway, regulates cell proliferation, differentiation, and survival (Supplementary Fig. 13). Its frequent dysregulation in cancers ( ~ 30% of tumors) makes it an attractive therapeutic target with lower susceptibility to resistance mutations compared to upstream pathway components. Notably, CETIF6a binds at sites structurally proximal to key functional regions, Cys178 neighbors Cys183 (within the ATP-binding pocket), Cys233 neighbors the phosphorylation sites Thr202 and Tyr204 (critical for ERK activation) (Fig. 6N). This spatial proximity suggests CETIF6a could serve as a scaffold for developing optimized, dual-function ERK-targeting molecules capable of both specific fluorescent labeling and potential functional modulation.

## Discussion

This study demonstrated that the incorporation of clickable functional groups at the side chain provides a viable strategy for functionalizing tumor-seeking dyes without compromising their targeting capability. By preserving the meso-Cl-cyclohexenyl core of IR-6B3 while introducing click chemistry handles, we developed probes retaining critical hydrophobicity driven tumor selectivity. The six-carbon side chain in CETIF6a achieves an optimal balance of spectroscopic properties, minimizing steric hindrance for rapid tissue penetration and maximizing biotin-azide conjugation efficiency. This design effectively attained an optimal equilibrium between the brightness and functionality of cyanine dyes.

Our structural clustering analysis revealed that CETIF6a reports diverse protein targets through conformationally adaptable interactions rather than a conserved binding motif. The identification of 491 conserved tumor-upregulated proteins provides a molecular basis for pan-cancer imaging. Unlike single-target probes^39,40^, CETIF6a exploits collective signal amplification from multiple conserved tumor proteins, ensuring signal robustness when individual biomarkers fail. This multi-target engagement strategy mitigates tumor heterogeneity-driven diagnostic inaccuracies. In addition to these tumor-upregulated proteins, whether high abundance metabolites in tumor cells can also bind to CETIF6a and contribute to fluorescence merits further discussion. To preliminarily explore this, we tested the binding of CETIF6a with glutathione, a metabolite commonly elevated in tumors (Supplementary Fig. 14). Under identical binding conditions, the fluorescence brightness of the probe upon binding to glutathione was substantially lower than that observed upon binding to proteins, indicating that the probe achieves a significant increase in brightness primarily through recognition and binding to target proteins.

While CETIF6a achieves > 90% margin concordance in CSC, BC, CCA and TC, reduced efficacy in HCC and GC or CRC highlights the limitations of pan-cancer interpretations. These are not probe failures but biological insights, which suggest that cirrhotic stroma mimics tumor protein profiles^41–43^. Future improvements can be achieved by discussing the differences in T/P tissue targets among these indistinguishable cancer types, which will help the probes to iteratively optimize for denser samples in normal tissues, thereby increasing the tumor specificity of the probes in such samples.

CETIF6a represents more than a surgical tool. Its conserved 491-target signature reveals fundamental Achilles’ heels in tumor evolution. The structural map of targets provides a blueprint for developing second generation probes with superior tumor-targeting capability. ERK targeted theranostics may be worthy of in-depth study combining fluorescence and kinase inhibition^44^. This mechanism not only aids in the precise delivery of therapeutics but also enhances imaging capabilities, thereby improving our understanding of cellular uptake and drug release mechanisms.

The development of probes operating in the NIR region to support surgical navigation systems has become a major focus in modern medical research. Probes within the NIR spectrum offer greater tissue penetration depth and higher signal to noise ratios, thereby largely improving the precision and effectiveness of surgical navigation. Our probe, CETIF6a, functions within this NIR window and is compatible with existing surgical navigation platforms. The design principles of CETIF6a provide a strategic foundation for developing targeted probes that enhance intraoperative guidance^45^.

Overall, this study provides a mechanistic investigation of tumor-seeking fluorescent probes. The unified dual functionality CETIF6a encompassing precision histological imaging and proteome wide covalent target profiling, unlocks opportunities for in-depth exploration of tumor-associated proteomes and structure guided refinement of probe specificity.

## Methods

### Ethics statement

All animal experiments were conducted under the institutional guidelines and were approved by the Animal Ethical Committee of the First Hospital of Jilin University (20210642). Human specimens were obtained from the Department of Biobank, Division of Clinical Research, the First Hospital of Jilin University. All experiments involving human specimens had passed the review of the Ethics Committee of the First Hospital of Jilin University (2023-284-1).

### Synthesis protocols and characterizations of CETIF

The synthesis protocols and characterizations of CETIF are available in the Supplementary Methods section of the Supplementary Information.

### Spectral characterization

Absorption and emission spectra of dyes were recorded with MARS-IRIS 400-1700 nm absorbance/fluorescence spectrophotometer (Artemis).

### Tissue protein extraction

The tissue samples were minced into small fragments, mixed with 200 μL of RIPA lysis buffer (BL504A, Biosharp), and subjected to ultrasonication for complete tissue disruption. After centrifugation of the lysate, the supernatant was collected, and the protein concentration was quantified using a detergent compatible Bradford protein assay kit (P0006C, Beyotime).

### Synthesis of dye@protein complex

To assess the covalent binding between the dye and tissue proteins, the tissue proteins were dissolved in 1 × phosphate-buffered saline (PBS) and adjusted to a final protein concentration of 10 μM. The dye was added at a 1:1 molar ratio, and the mixture was incubated under light-protected conditions with continuous agitation at room temperatures for 2 h. Similarly, to assess the covalent binding between the dye and recombinant candidate proteins, the dye and protein were mixed at a molar concentration of 1:1 (the final concentration was 2 μM) and incubated with disturbance at room temperature for 2 h.

### Gel electrophoresis

SDS-PAGE was performed using a PAGE Gel Quick Preparation Kit (20326ES62, Yeasen) to prepare polyacrylamide gels (10%-12.5% separating gel and 4% stacking gel). The Dye@protein complexes were mixed with loading buffer and loaded onto the gel. Electrophoresis was initially conducted at 80 V, and the voltage was increased to 120 V after the samples migrated to the interface between the stacking and separating gels.

### Fluorescence imaging

For fluorescence imaging of dye@protein complexes in solution (in tube) and post-electrophoresis gel bands, the Sapphire FL Biomolecular Imager (Azure biosystems) was employed. The excitation wavelength was set to 784 nm, and emitted light was captured within the 832 nm bandpass (37 nm width) using AZURE software. All imaging data were processed with ImageJ software.

### H&E staining

The Tissue-Tek® O.C.T. Compound (4583, Sakura)-embedded tissue blocks were sectioned using a cryostat (FS800, RWD Life Science) to a thickness of 5–15 μm, and the sections were mounted onto glass slides for H&E staining. The staining protocol was performed sequentially as follows: 95% ethanol (1 min), water (30 s), hematoxylin (BKMAM Biotechnology) (6-8 min), water (30 s), differentiation solution (3–5 s), water (30 s), water-soluble eosin (PH0500, Phygene Biotechnology) (30 s), water (10 s), 80% ethanol (10 s), 95% ethanol (10 s, twice), absolute ethanol (10 s, twice). After staining, the slides were gently dried using low-heat airflow at a distance and mounted with a coverslip using mounting medium. The stained slides were then scanned with an Olympus VS200 research-grade whole-slide scanning system equipped with a UPLXAPO20X NA0.8 objective (Olympus). Image processing and analysis were performed using OlyVIA software.

### Slide staining

The operation steps can be referenced from our previous publication^20^. The sections were gently washed 3 times with PBST (phosphate-buffered saline with 0.1% Tween-20) to remove residual OCT. Subsequently, the sections were incubated with a 5 nM dye solution (prepared by diluting the dye in 1× PBS) for 15 min. After incubation, the sections were washed twice with DMSO (1 min per wash) and once with PBST (1 min). Finally, the slides were air-dried in the dark and imaged using a Sapphire FL Biomolecular Imager (Azure biosystems) at a resolution of 10 μm for fluorescence analysis. The excitation wavelength was set to 784 nm, and emitted light was captured within the 832 nm bandpass (37 nm width).

### Animal experiments

BALB/c mice were purchased from Liaoning Changsheng Biotechnology Co., Ltd. Bedding, nesting material, food, and water were provided ad libitum. The ambient temperature was maintained at 20 °C–22 °C with a 12‑hour light/12‑hour dark cycle. For mouse tumor model construction and tumor tissue extraction, 5 × 10⁶ breast cancer cells (4T1, purchased from Shanghai Enzyme Research Biotechnology Co., LTD) were suspended in 100 μL PBS and injected subcutaneously into the thigh of 3 female Balb/c mice (6–8 weeks old). Then, 21 days after tumor inoculation, the mice were used for tumor tissue extraction. The maximal tumor size permitted by the ethics committee is 1000 mm³, and this limit was not exceeded during the study.

### Human Biospecimens

Tissue biospecimens were collected from patients who underwent surgical resection at The First Hospital of Jilin University. Written informed consent was obtained from all patients. The cohort included 20 cervical cancer, 13 breast cancer, 8 cholangiocarcinoma, 4 thyroid cancer, 4 liver cancer, 3 gastric cancer, 3 colon cancer, and 3 rectal cancer cases. Sex/gender was not considered because the study focused on pan‑tumor labeling across cancer types, not sex differences. The cohort included both female‑only cancers (cervical, breast) and sex‑balanced cancers. All samples came from clinical resections without sex preselection. More detailed sample information, including sample type and clinical diagnosis, is provided in Supplementary Tables 1-2. For each patient, paired tumor and adjacent non‑tumor tissues ( ≥ 2 cm from the tumor margin) were collected intraoperatively and stored at −80 °C until further analysis. All patients were alive at the time of biospecimen acquisition. Clinical and pathological diagnoses were confirmed by pathologists. Tissue sections were reviewed to ensure a minimum tumor nuclei percentage of 80% and a maximum necrotic area of 20% before inclusion.

### Protein labeling, cycloaddition, and purification

A total of 36 clinical tissue samples were analyzed, comprising 18 tumor tissue samples and 18 paired paracancerous tissue samples. Tumor tissues of 4 cancer types were included as the experimental group, with the corresponding paracancerous tissues from the same patient serving as controls. Specifically: cervical cancer (5 cases), breast cancer (5 cases), cholangiocarcinoma (5 cases), and thyroid cancer (3 cases).

*Labeling:* Proteins were diluted to 2 mg/mL in 1× PBS (1 mL total volume). Dye was added at a 1:1 molar ratio to the protein, ensuring that the volume of DMSO did not exceed 20 μL. The mixture was incubated in the dark at room temperature for 2 h with gentle agitation. Following incubation, 10 μL of the sample was aliquoted for subsequent analysis.

*Cycloaddition:* Each sample was divided into 4 aliquots (250 μL each). To each aliquot, the following reagents were sequentially added: Biotin-PEG4-Azide (B5546, TCI): 1.4 µL of stock solution (20 mM) to a final concentration of 110 µM (vortexed). Tris-(2-carboxyethyl)-phosphine hydrochloride (TCEP, 20330ES03, Yeasen): 5 µL of stock solution (50 mM) to a final concentration of 1 mM (no vortexing). Tris (1-benzyl-1H-1,2,3-triazol-4-yl) methyl amine (TBTA, 678937, Sigma-Aldrich): 15 µL of stock solution (1.7 mM) to a final concentration of 102 µM (vortexed). CuSO_4_ (A603008, Sangon biotech): 5 µL of stock solution (50 mM) to a final concentration of 1 mM (vortexed). The reaction mixtures were incubated in the dark at room temperature for 1 h, with brief vortexing at 30 min. After completion, all 4 aliquots were pooled. Methanol (4 mL) and chloroform (1 mL) were added to the combined sample, followed by vortexing. Water (3 mL) was added, and the mixture was centrifuged at 4 °C (4000 × *g*, 15 min). Protein precipitates formed at the aqueous-organic interface were collected by removing the upper and lower phases. The precipitates were transferred to a 1.5 mL tube using 2 × 300 µL methanol. Chloroform (150 µL) and water (600 µL) were added, followed by centrifugation (4 °C, 9000 × *g*, 5 min). The upper and lower phases were discarded, and the protein pellet was washed twice with 600 µL ice-cold methanol (sonication for resuspension, centrifugation at 9000 × *g*, 5 min). The pellet was dissolved in 1 mL of 1.2% SDS/PBS at 60 °C–70 °C for 5 min, cooled on ice for 2 min, and centrifuged (4 °C, 19,000 × *g*, 5 min). The supernatant was collected, diluted with 5 mL PBS to reduce SDS concentration to 0.2%, and stored at −80 °C.

*Purification:* Streptavidin-agarose beads (20512ES08, Yeasen) were equilibrated by removing ethanol storage solution and washing 3 times with 5 mL PBS. The beads were resuspended in PBS (equal bead volume) and added to the cycloaddition product. The mixture was incubated at 4 °C on a vertical rotator for 4 h. After incubation, the supernatant was removed, and the beads were sequentially washed with 5 mL 0.2% SDS/PBS (10 min rotation), 5 mL PBS (3 times) and 5 mL water (3 times). Beads were then transferred to a 1.5 mL tube using 1 mL water, centrifuged (500 × *g*, 5 min), and the supernatant was discarded. Enriched proteins were eluted by boiling the beads with 2.5 × loading buffer at 100 °C for 5 min. Samples were analyzed via SDS-PAGE for fluorescence imaging and Coomassie blue staining to evaluate enrichment efficiency. Data were processed using ImageJ, and figures were generated using GraphPad Prism 8.

### Quantitative mass spectrometry for proteomic samples

Label-free quantitative proteomics analysis was performed by BGI Protein (Shenzhen, China). Protein was transferred to a 10 kDa molecular weight cutoff filter unit. After buffer exchange with 0.5 M triethylammonium bicarbonate (TEAB) by centrifugation (20 °C, 12,000 × *g*, 20 min, repeated 3 times), trypsin was added at an enzyme: protein ratio of 1:20 and digestion was carried out at 37 °C for 4 h. The resulting peptides were collected by centrifugation, dried under vacuum, and reconstituted in mobile phase A (2% acetonitrile, 0.1% formic acid). The peptide mixture was separated on an eksigent ultra 2D nano‑liquid chromatography system (SCIEX) using a self‑packed C18 column (75μm inner diameter, 3μm particle size, 20 cm length) at a flow rate of 300nL/min. The separation gradient was: 0–30 min, mobile phase B (98% acetonitrile, 0.1% formic acid) from 5% to 24%; 30-40 min, B from 24% to 30%; 40-45 min, B from 30 to 80%; 45–50 min, hold at 80% B; 50–60 min, re‑equilibration at 5% B. The LC system was coupled to a TripleTOF 5600 system (SCIEX, Framingham) equipped with a Nanospray III source (SCIEX, Framingham) and a quartz emitter (New Objectives, Woburn). The ion spray voltage was set to 2300 V, nitrogen pressure to 35 psi, nebulizer gas to 15, and interface temperature to 150 °C. MS1 spectra were acquired in high‑sensitivity mode over the mass range of 350–1500 m/z with an accumulation time of 250 ms. The top 40 precursor ions with intensity above 150 counts per second, charge states 2-5, and m/z 350–1250 were selected for MS/MS fragmentation using rolling collision energy, with an accumulation time of 50 ms. Dynamic exclusion was set to approximately 12 s (half of the peak width). Raw data were processed using the Andromeda search engine integrated into MaxQuant (version 2.1.3.0). Spectra were searched against the UniProtKB/Swiss-Prot Homo sapiens database (20,308 sequences). The search parameters included: trypsin as the protease with up to 2 missed cleavages; fixed modification of carbamidomethyl (C); variable modifications of oxidation (M), acetylation (protein N‑term), deamidation (NQ), and Gln to pyro‑Glu; precursor mass tolerance of 10ppm; fragment mass tolerance of 20ppm; and a minimum peptide length of 7 amino acids. Peptide‑spectrum matches (PSMs) and protein identifications were both filtered at a false discovery rate (FDR) of ≤ 1%. Protein quantification was based on the intensity values (peak area and retention time alignment) extracted by MaxQuant. Differentially expressed proteins were defined as those with a fold change > 2 between tumor and paracancerous tissues.

### 3D histological electrophoresis

The 3D electrophoresis system was custom-built, and its design can be referenced from our previous publication^21–23^. For each sample, 4 adjacent 60 μm-thick frozen tissue sections were mounted on the rough surface of a 20×20 array plate, while adjacent 10 μm-thick sections were placed on glass slides for H&E staining and histological validation. The array plates with tissue sections were then immersed in RIPA lysis buffer containing 1 μM dye and incubated in the dark for 30 min to simultaneously achieve tissue lysis and in situ protein staining. Subsequently, 3D electrophoresis was performed using a 10% SDS-PAGE gel. After electrophoresis, the PAGE gel blocks were completely frozen in -80 °C freezer, and the separating gel regions were sectioned using a cryostat with a strictly maintained thickness of 600 μm per slice. The sliced gel sections were imaged using a Sapphire FL Biomolecular Imager (Azure biosystems) at a resolution of 100 μm. The excitation wavelength was set to 784 nm, and emitted light was captured within the 832 nm bandpass (37 nm width). The acquired images were processed with Image Studio software, and the data were visualized using GraphPad Prism 8 for plotting and analysis.

### Plasmids construction

Total RNA was extracted from the tissue samples and used as the template to synthesize cDNA via a reverse transcription kit. The target genes of *KYNU*, *LCP1*, *PCNA* and *DNAJC9* were amplified from the cDNA using specific primers. PCR primers are documented as below:

KYNU-F: 5’ CATCATCACCACCATGAGCCTTCATCTCTTGAGCTG 3’

KYNU-R: 5’ ACCGAGCTCCATTCAATTTTTTGTTTCTGCAGAG 3’

LCP1-F: 5’ CATCATCACCACCATGCCAGAGGATCAGTGTCCGA 3’

LCP1-R: 5’ ACCGAGCTCCATTCACACCCTCTTCATTCCTTTCC 3’

PCNA-F: 5’ ATGTTCGAGGGGCGCC 3’

PCNA-R: 5’ CTCAGTGGTGGTGGTGGTGGTGAGATCCTTCTTCATCCTCG 3’

DNAJC9-F: 5’ ATGGGGCTGCTGGACC 3’

DNAJC9-R: 5’ GATCTCAGTGGTGGTGGTGGTGGTGTTTCTTTTCTTTCTTGAGA 3’

The amplified gene fragments were then precisely inserted into the Multiple Cloning Site (MCS) of the pColdII vector with the help of Ready-to-Use Seamless Cloning Kit (B632219, Sangon biotech). All constructed plasmids were validated by sequencing prior to experimental use.

### Bacterial expression and purification of recombinant proteins

Rosetta (DE3) bacteria (D1065S, Beyotime) was transformed with pColdII series plasmids and cultured in LB medium to OD_600_ 0.6–0.8 (37 °C, 220 rpm). 200 μM IPTG was added to induce the expression of recombinant proteins (15 °C, 24 h, 180 rpm). After induction, the cells were suspended in PBS buffer and lysed by sonication to extract total proteins. Recombinant proteins with his tags were then purified by Ni Sepharose (SA036100, Smart-Lifesciences). The molecular weight and the purity of the purified protein was confirmed by Coomassium blue R250 staining (20309ES03, Yeasen). The ERK1 protein was purchased from MedChemExpress (MCE) at a purity suitable for biochemical assays. The catalog number is HY-P701678.

### Immunohistochemical Staining

15 paired paraffin-embedded human cervical cancer and paracancerous tissue microarray (TMA) sections (2 mm core diameter) were baked at 65 °C for 1 h. Slides underwent deparaffinization in xylene and rehydration through graded ethanol solutions. Antigen retrieval was performed using Tris-EDTA buffer (pH 9.0) or citrate buffer (pH 6.0) under microwave heating. After blocking endogenous peroxidase activity, sections were incubated with 5% BSA for 30 min at room temperature. Sections were then incubated overnight at 4 °C with rabbit monoclonal anti-PCNA (Abcam, ab92552, 1:250 dilution) and rabbit monoclonal anti-DNAJC9 (Abcam, ab166612, 1:100 dilution). Following washes, horseradish peroxidase (HRP)-conjugated goat anti-rabbit secondary antibody was applied. Signals were developed using DAB substrate (20 s). Nuclei were counterstained with hematoxylin, followed by dehydration, clearing, and cover slipping. Imaging was performed using an Olympus VS200 research-grade whole-slide scanning system.

### Molecular docking

The crystal structure of the target protein was imported, followed by assignment of bond orders and formal charges to the ligand. Ionization states and tautomeric forms were optimized for both protein and ligand, after which energy minimization with conformational sampling was performed. The active site was identified according to the provided protein structure, and the CETIF6a structure was optimized. Non-covalent molecular docking was executed using the Glide module, with subsequent binding energy calculations via MM-GBSA. For covalent docking studies, the Covalent Docking program was employed, followed by 50 ns molecular dynamics simulations of the resulting complexes using Desmond. Binding energies for covalent complexes were finally calculated using MM-GBSA methodology.

### Structural clustering and analysis

All protein crystal structures corresponding to the UniProt IDs were downloaded from the Protein Data Bank (PDB). Foldseek was employed to perform structural clustering analysis. Structures were aligned and clustered based on their 3D similarity. Structures were grouped into clusters using a structural similarity threshold of 30% (measured by TM-score or aligned residue coverage). Each cluster was annotated with UniProt IDs, PDB accession codes, and structural metadata. The number of proteins in each cluster was quantified and visualized using bar plots with GraphPad Prism 8.

### Site-specific analysis of dye-modified target proteins by LC-MS

Protein site-specific analysis was performed by Beijing Bio-Tech Pack Technology using liquid chromatography-mass spectrometry (LC-MS). For each sample, 50 μL protein solution was mixed with 50 μL water in a 1.5 mL tube. For reduced samples, 10 mM DTT was added, followed by incubation at 56 °C for 1 h. Alkylation was then performed with 20 mM iodoacetamide (IAM) in the dark at room temperature for 40 min. The reaction was quenched by adding 10 mM DTT. For non-reduced samples: 20 mM IAM was added and incubated in the dark at room temperature for 40 min. All samples were enriched using SP3 beads (800 rpm, 15 min, room temperature) and washed 3 times with 80% ethanol. Each sample was digested in duplicate. For trypsin digestion, 2 μg trypsin was added, and samples were incubated at 37 °C with agitation for 16 h. For chymotrypsin digestion, 2 μg chymotrypsin was added, and samples were incubated at 30 °C with agitation for 16 h. After digestion, samples were placed on a magnetic rack for 5 min. The supernatant was transferred to a new tube, vacuum-dried, reconstituted in 0.1% formic acid (FA), desalted, and dried again prior to LC-MS analysis.

Chromatographic separation was performed on a Vanquish Neo nL UHPLC system (Thermo Fisher Scientific) with the following parameters: Mobile phase A: 0.1% FA in water. Mobile phase B: 0.1% FA in 80% acetonitrile. Gradient: 0–2 min, 4–8% B. 2–35 min, 8–28% B. 35–55 min, 28-40% B. 55–56 min, 40–95% B. 56–66 min, 95% B. Flow rate: 0.6 μL/min. Column: Reprosil-Pur 120 C18-AQ (1.9 μm, 150 μm i.d. × 170 mm).

Mass spectrometry (MS) data were acquired using an Orbitrap Fusion Lumos Tribrid mass spectrometer (Thermo Fisher Scientific). MS1 parameters: Resolution, 120000. AGC target, standard. Maximum injection time (Max IT), 20 ms. Scan range, 300–1800 m/z. MS2 parameters: Resolution, 15000. AGC target, standard. Max IT, 22 ms. Cycle time, 2 s. Normalized collision energy (NCE), 30 (stepped mode).

Raw data were analyzed using the Peptide Mapping module in BioPharma Finder 5.1 software (Thermo Fisher Scientific) with the following parameters: Protease: Trypsin or chymotrypsin. Variable modifications: Deamidation (N/Q), oxidation (M), carbamidomethylation (C), and small molecule adducts (-Cl-H, -I-Cl-H). Mass accuracy tolerance: 20 ppm.

### Statistical analysis

Results were presented as mean ± Standard Error of Mean (SEM). For all statistical tests, P value < 0.05 were considered significant. Quantified similarity was calculated using MATLAB R2014a.

For database analysis of KEGG and DO^46^, the calculation formula of P value is

1 $$P=1-\,\mathop{\sum }_{i=0}^{m-1}\frac{\left({{M}\atop{i}}\right)\left({{N-M}\atop{n-i}}\right)}{\left({{N}\atop{n}}\right)}$$

Among them, N is the number of all genes (background genes), n is the number of differentially expressed genes (target genes), M is the number of this pathway in all genes, and i is the number annotated to this pathway in the differentially expressed genes. After the calculated P value is corrected by FDR, with correction-P value ≤ 0.05 as the threshold, a pathway that meets this condition was defined as a pathway that was significantly enriched in differentially expressed genes.

For database analysis of single-cell transcriptomic, Expression profiles of target genes were cross-referenced with single-cell RNA sequencing data from 11,289 cervical cancer cells and 9,649 adjacent normal cervical cells^36,47^. Gene expression abundance was quantified across distinct cell populations.

For intuitive presentation of raw data in the Source Data file, mean values of fluorescence intensity and fluorescence ratios are shown to 2 decimal places, whereas SD and SEM are shown to 3 decimal places.

### Reporting summary

Further information on research design is available in the Nature Portfolio Reporting Summary linked to this article.

## Supplementary information

Supplementary Information

Reporting Summary

Transparent Peer Review file

## Source data

Source Data

## Data Availability

The mass spectrometry proteomics data generated in this study have been deposited to the ProteomeXchange Consortium via the iProX partner repository with the dataset identifier PXD069296. The data can be accessed via ProteomeXchange at: https://proteomecentral.proteomexchange.org/cgi/GetDataset?ID=PXD069296. The data are also available through the iProX repository at: https://www.iprox.cn/page/project.html?id=IPX0013731000. The source data generated in this study are provided in the Supplementary Information and Source Data file. Source data are provided with this paper.
