# Supplementary material for: A NIR fluorotag reporter CETIF6a enables bright pan-tumor labeling and functional proteomic profiling
Source: Nat Commun. 2026 Jul 8;17:8451. doi: 10.1038/s41467-026-75356-3 (PMC13478164; doi:10.1038/s41467-026-75356-3)

Supplementary Information (SI) for A NIR fluorotag reporter CETIF6a enables bright pan-tumor labeling and functional proteomic profiling

Jia Li et al.

Supplementary Figures

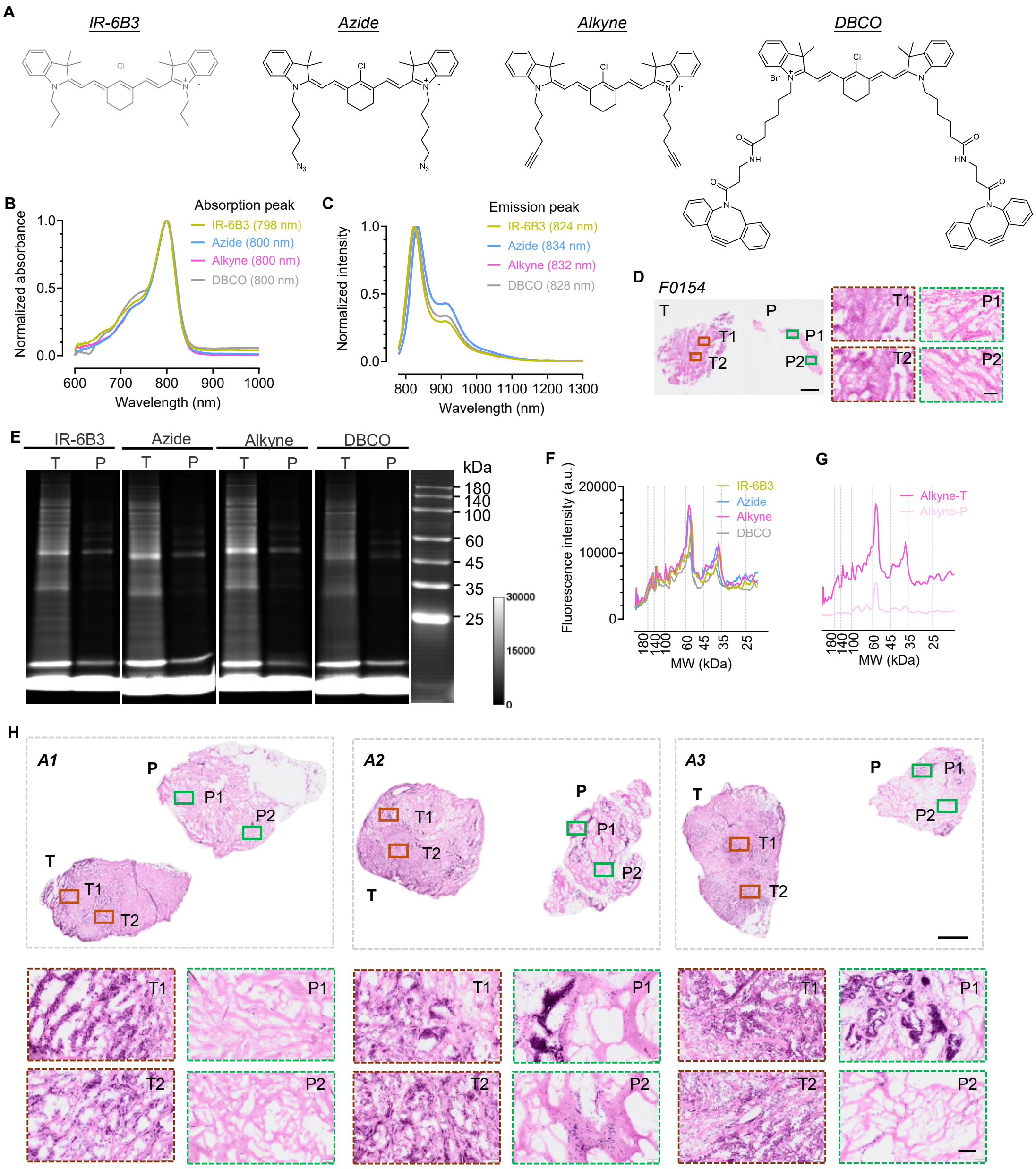

Supplementary Figure 1. Capability testing of CETIF with different chemical clicking functional groups. (A) The structural formula of CETIF. (B-C) Absorption and emission spectrum of IR-6B3, Azide, Alkyne and DBCO, with each spectrum normalized to a peak value of 1.000. (D) H&E staining of clinical samples used for fluorescent test in Figure 2B. Scale bars: 1 mm (1× H&E), 100 μm (20× H&E). (E) Unidirectional electrophoresis evaluated the ability of different probes to covalently target proteins in cervical cancer sample F0154. (F) Distribution of fluorescence bands across molecular weights in panel E, assessing consistency in tissue protein species targeted by different dyes. (G) Distribution of fluorescence bands labeled by Alkyne across molecular weights in panel E, assessing the brightness contrast of tumor and paracancer. (H) H&E staining of clinical samples used for fluorescent test in Figure 2D. Scale bars: 1 mm (1 × H&E), 100 μm (20 × H&E). Source data are provided as a Source Data file.

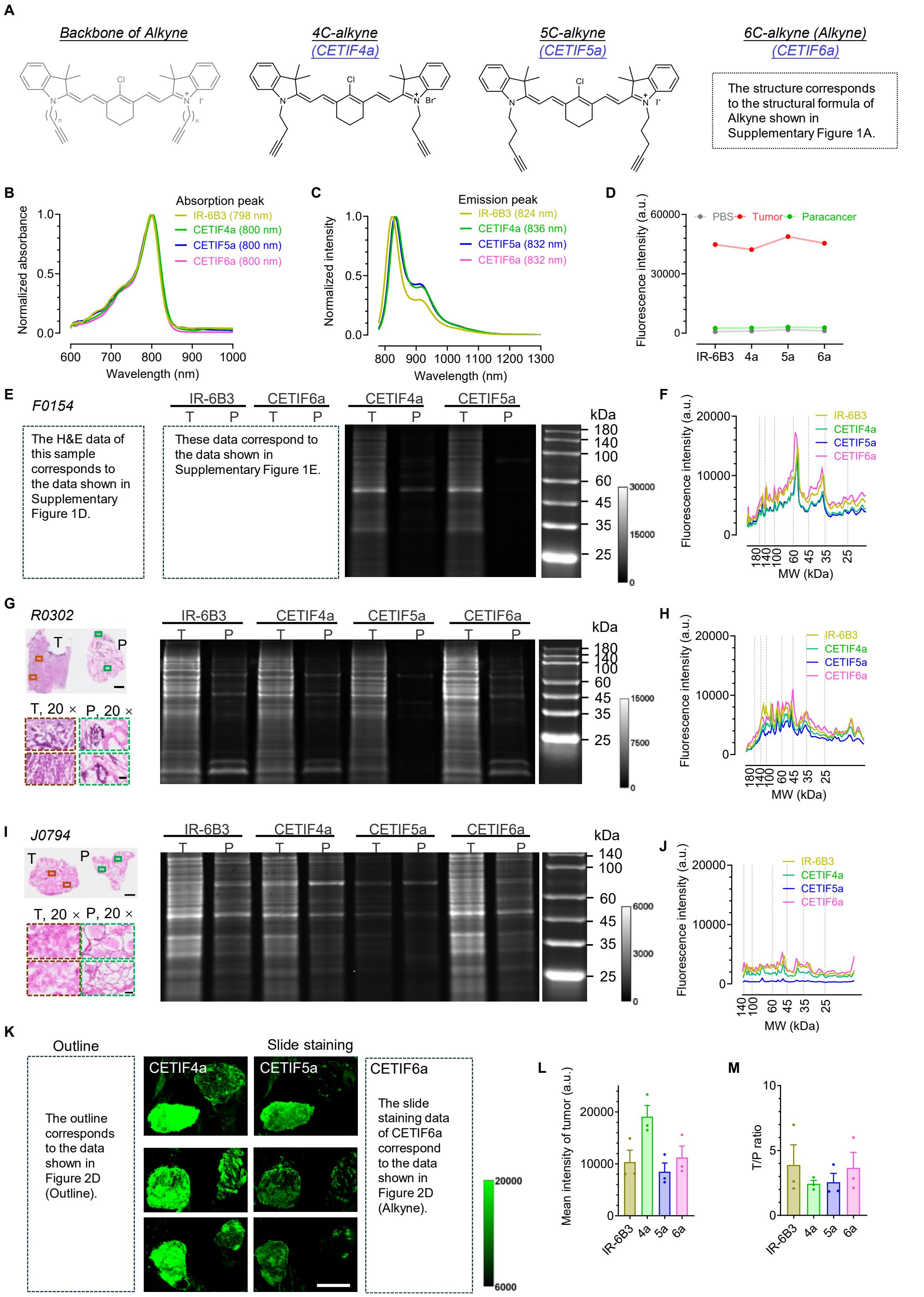

Supplementary Figure 2. Tumor-labeling capability testing of CETIF with different side chain lengths. (A) The structural formula of Alkyne with different side chain lengths. (B-C) Absorption and emission spectrum of IR-6B3 and CETIF4/5/6a, with each spectrum normalized to a peak value of 1.000. (D) Fluorescence intensity of dyes in PBS and tissue lysate as solvents, the final concentration of the probes was 10 μM (mean ± SEM, n = 3 samples per group). (E)

Unidirectional electrophoresis evaluated the ability of different probes to covalently target proteins in cervical cancer sample F0154. (F) Distribution of fluorescence bands across molecular weights in panel E. (G) Unidirectional electrophoresis evaluated the ability of different probes to covalently target proteins in breast cancer sample R0302. Scale bars: 1 mm (1× H&E), 100 µm (20× H&E). (H) Distribution of fluorescence bands across molecular weights in panel G. (I) Unidirectional electrophoresis evaluated the ability of different probes to covalently target proteins in thyroid cancer sample J0794. Scale bars: 1 mm (1× H&E), 100 µm (20× H&E). (J) Distribution of fluorescence bands across molecular weights in panel I. (K) Imaging efficacy of CETIF4/5/6a mediated slide staining in delineating tumor regions of clinical cancer samples (the length of the scale bar: 5 mm). (L) Statistical comparison of the fluorescence intensities of the tumor regions labeled by different dyes in panel K (mean ± SEM, n = 3 samples per group). (M) Statistical comparison of the fluorescence ratio between T and P regions labeled by different dyes in panel K (mean ± SEM, n = 3 samples per group). Source data are provided as a Source Data file.

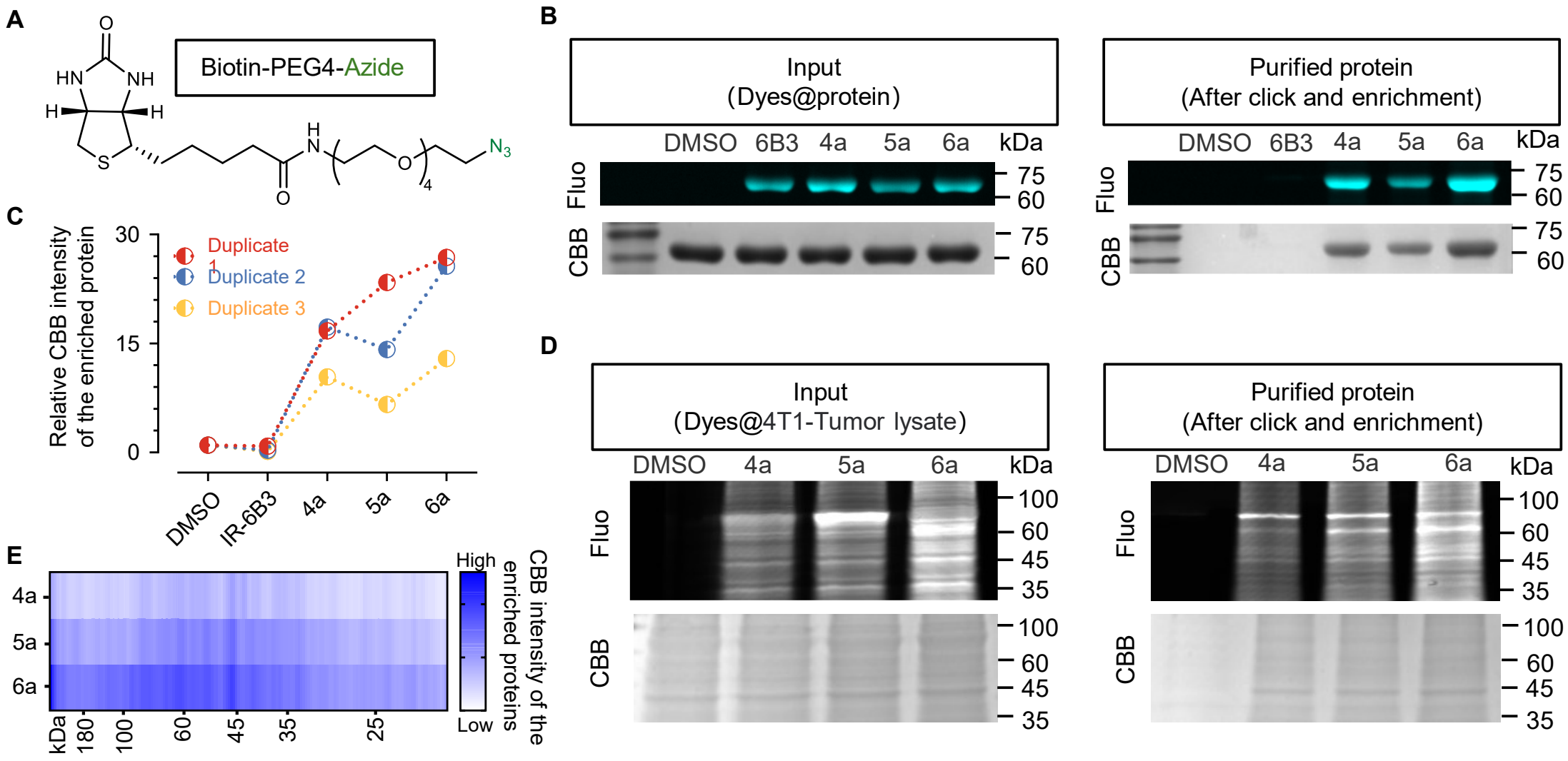

Supplementary Figure 3. Evaluation of Alkyne functionality for targeted proteome dentification. (A) Biotin-PEG4-Azide was designed for biotin labeling on Alkyne. (B) Representative analysis of alkyne's capability to label and enrich pure human serum albumin. Fluorescence lanes show the band brightness of covalent complexes before (input) and after (purified protein) enrichment. CBB lanes display protein content of covalent complexes before (input) and after (purified protein) enrichment (n = 3 independent experiment). (C) Quantitative analysis of enrichment efficiency from panel B. The y-axis represents the normalized content of enriched target protein (calculated as CBB intensity of purified protein / CBB intensity of input) to eliminate variations caused by input loading differences. (D) Representative analysis of alkyne's capability to label and enrich proteins from 4T1 tumor tissue lysates in mice. Fluorescence lanes show the band brightness of covalent complexes before (input) and after (purified protein) enrichment. CBB lanes display protein content of covalent complexes before (input) and after (purified protein) enrichment (n = 3 independent experiment). (E) Heatmap visualization of CBB band intensities for purified proteins enriched by different dyes in panel D, providing an intuitive comparison of protein content and diversity. Source data are provided as a Source Data file.

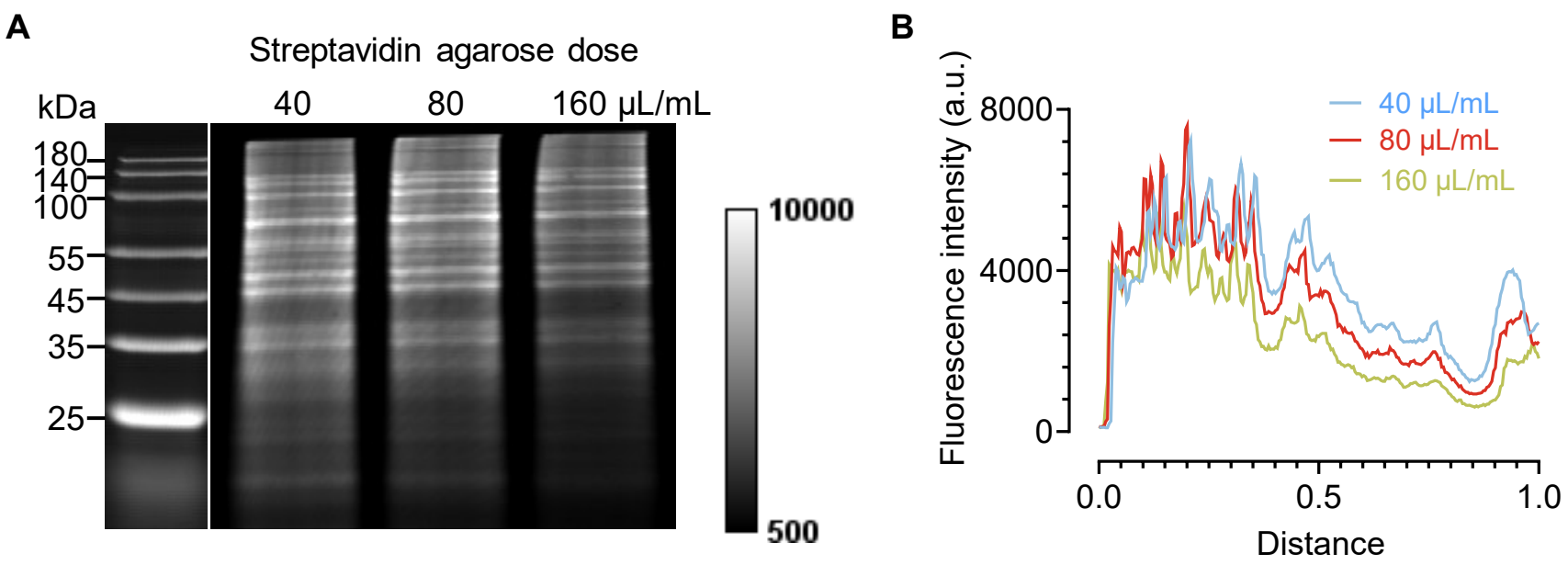

Supplementary Figure 4. Optimization of streptavidin-agarose usage. (A) Enrichment efficiency testing using the CETIF6a enriched R0302 tumor sample as a model. Electrophoretic analysis of fluorescence bands after enrichment with varying final concentrations of streptavidin-agarose, where fluorescence intensity reflects enriched protein content. (B) Quantitative analysis of fluorescence intensity from panel A. Source data are provided as a Source Data file.

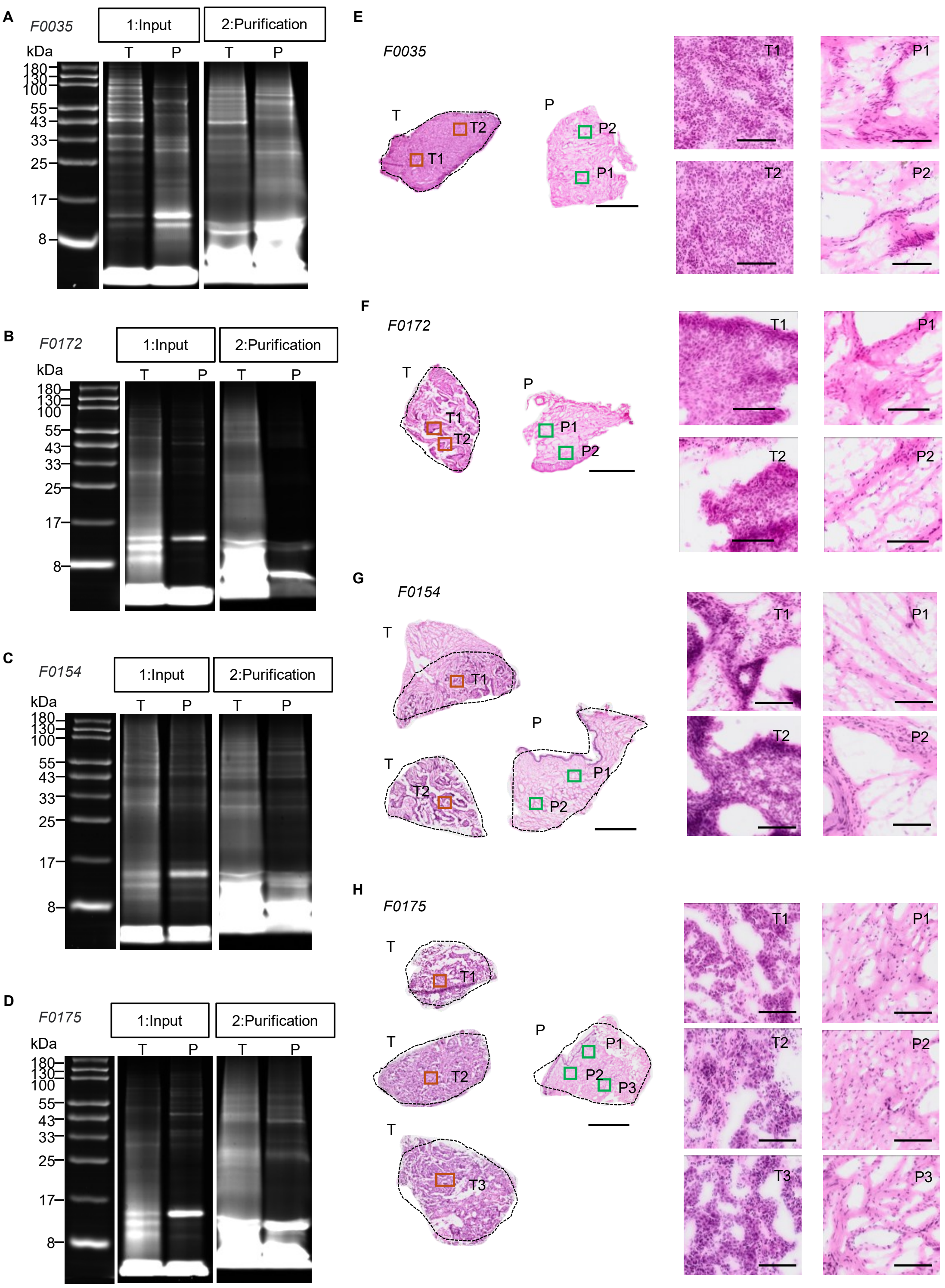

Supplementary Figure 5. Electrophoresis results of proteome labeled and enriched following the workflow. (A-D) Proteome labeled (input) and enriched (purified) from clinical cervical cancer samples. (E-H) H&E staining of clinical samples used for protein enrichment among A-D. Scale bars: 1 mm (1 × H&E), 100 μm (20 × H&E).

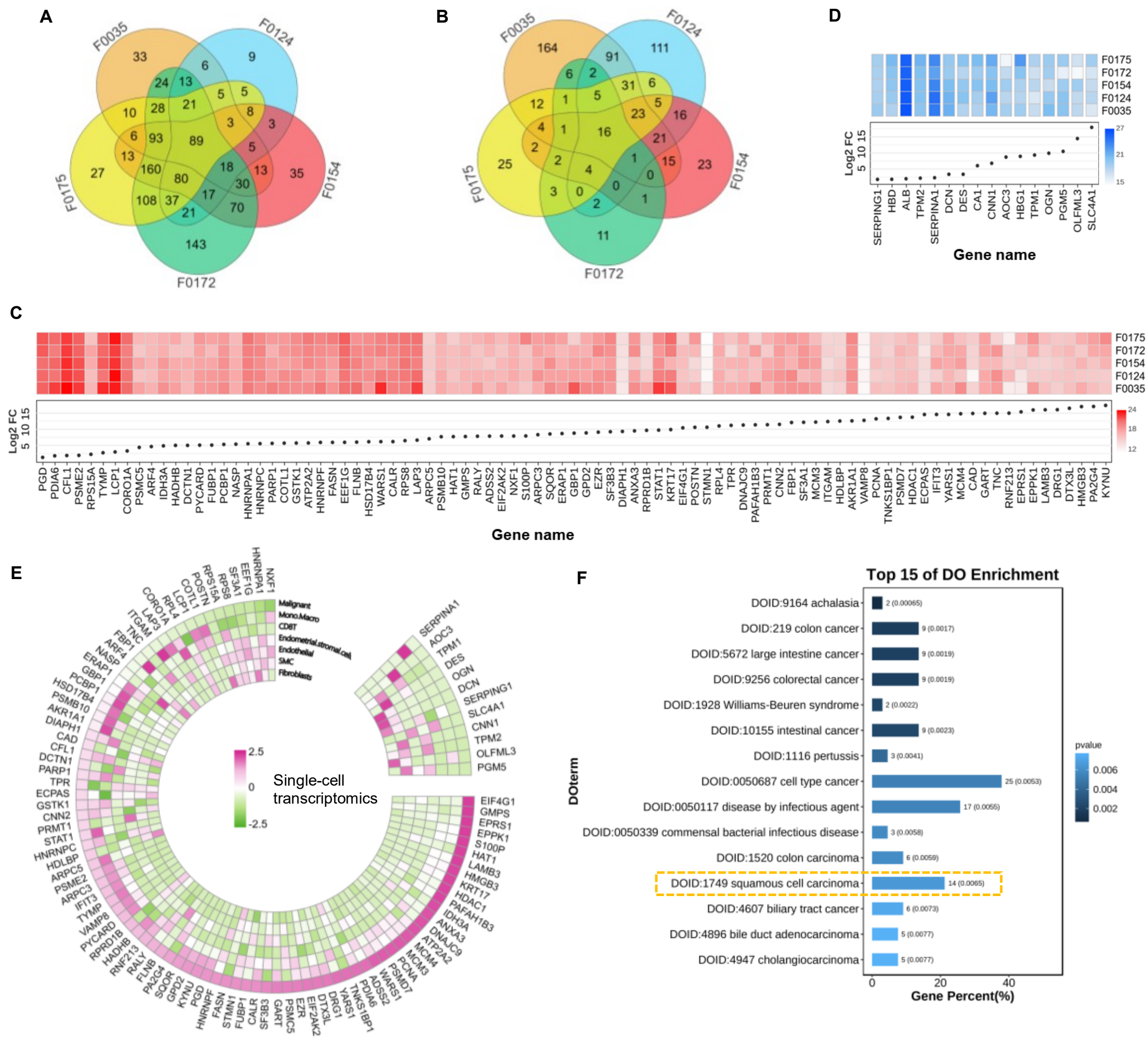

Supplementary Figure 6. Statistical analysis of CETIF6a-targeted proteins identified by chemical proteomics in 5 cervical cancer samples. (A) Venn diagram of upregulated proteins in tumor (T) groups across 5 samples. (B) Venn diagram of upregulated proteins in paracancerous (P) groups across 5 samples. (C-D) Intersection analysis of upregulated target proteins: Gene names, intensity, and FC for proteins common to all 5 samples in (C) T-group and (D) P-group. (E) Single-cell transcriptome heatmap of proteins from panels C and D, showing their abundance in different cervical cancer cell types. Malignant, Mono. Macro, and CDT8 are enriched in tumor tissues; Fibroblasts, SMC, Endometrial, and Endothelial are predominant in normal tissues. Data was normalized. (F) Disease Ontology (DO) pathway analysis of upregulated target proteins in tumor groups, ranked by p-value (top 15 pathways shown). Source data are provided as a Source Data file.

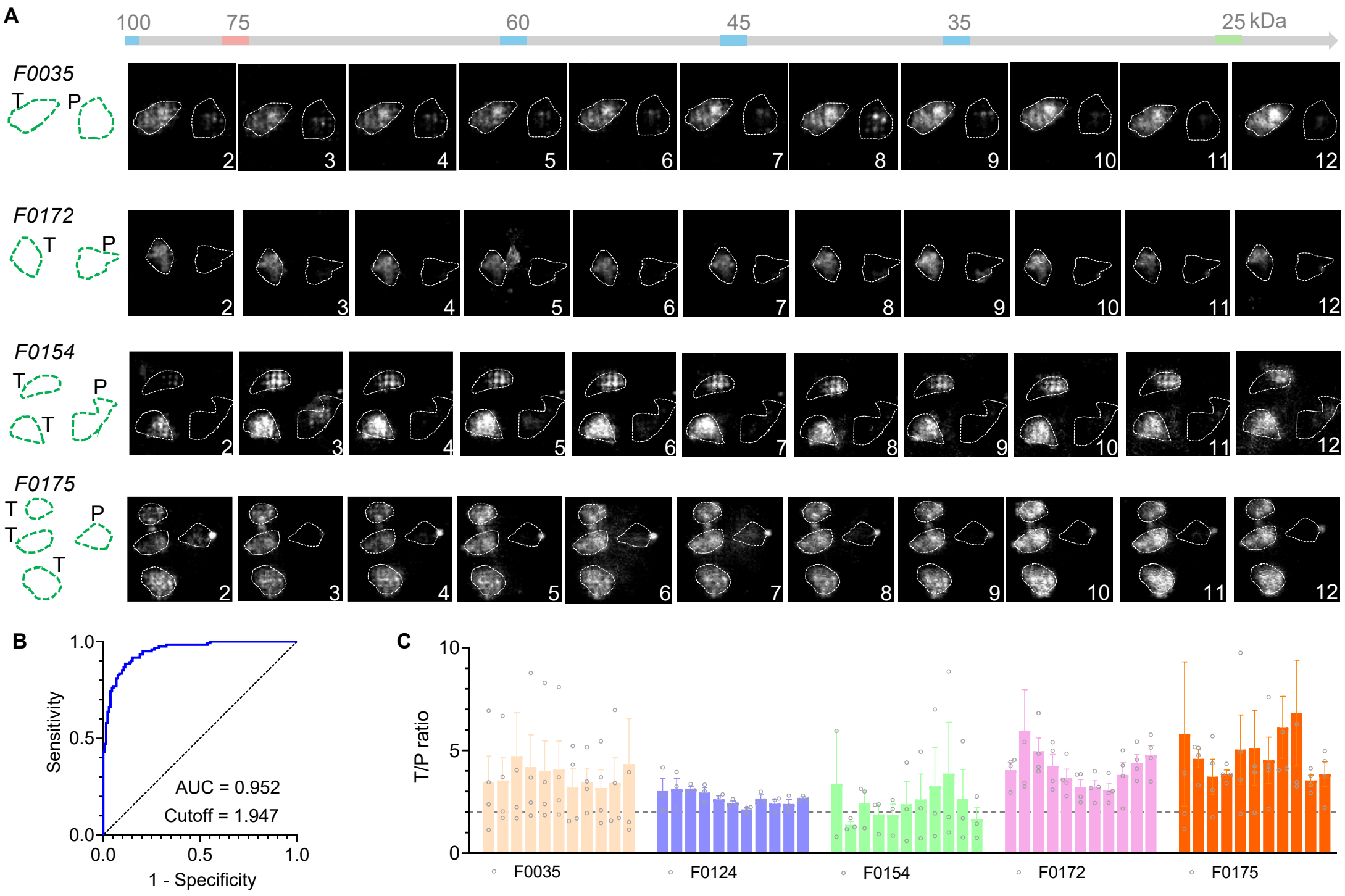

Supplementary Figure 7. CETIF6a-mediated 3D histoelectrophoresis for tumor margin delineation in 5 cervical cancer samples. (A) Imaging results of layers 2-12 in 3D histological electrophoresis for each sample. The molecular weight scale (top) was calibrated using protein marker bands. Regions outlined by white lines correspond to tumor and paracancerous areas defined by H&E staining. (B) Cutoff value determination for cervical cancer sample analysis using 3D histological electrophoresis. (C) Statistical analysis of T/P ratios across layers 2-12 for each sample (mean ± SEM, n = 5 samples, all samples were analyzed with n = 3 replicates.). Source data are provided as a Source Data file.

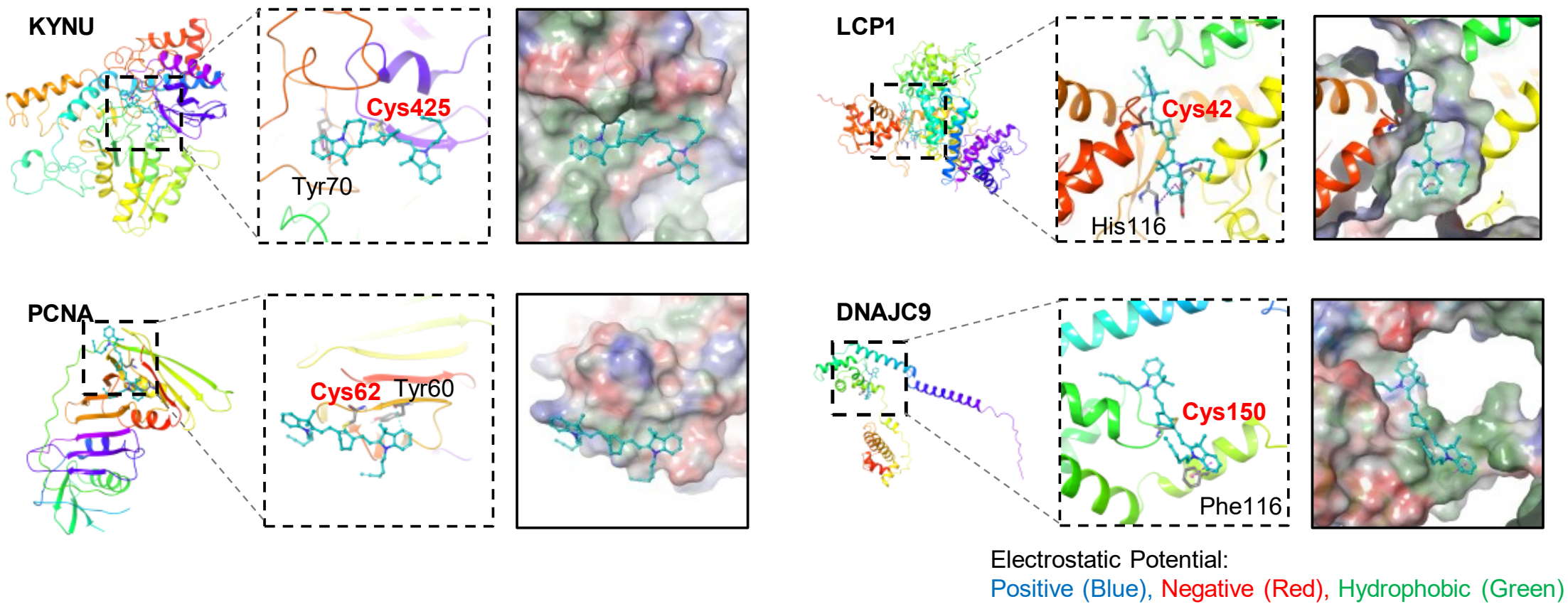

Supplementary Figure 8. Molecular docking of CETIF6a with CSC targets. Covalent binding sites are highlighted in red, interacting amino acids are labeled in black.

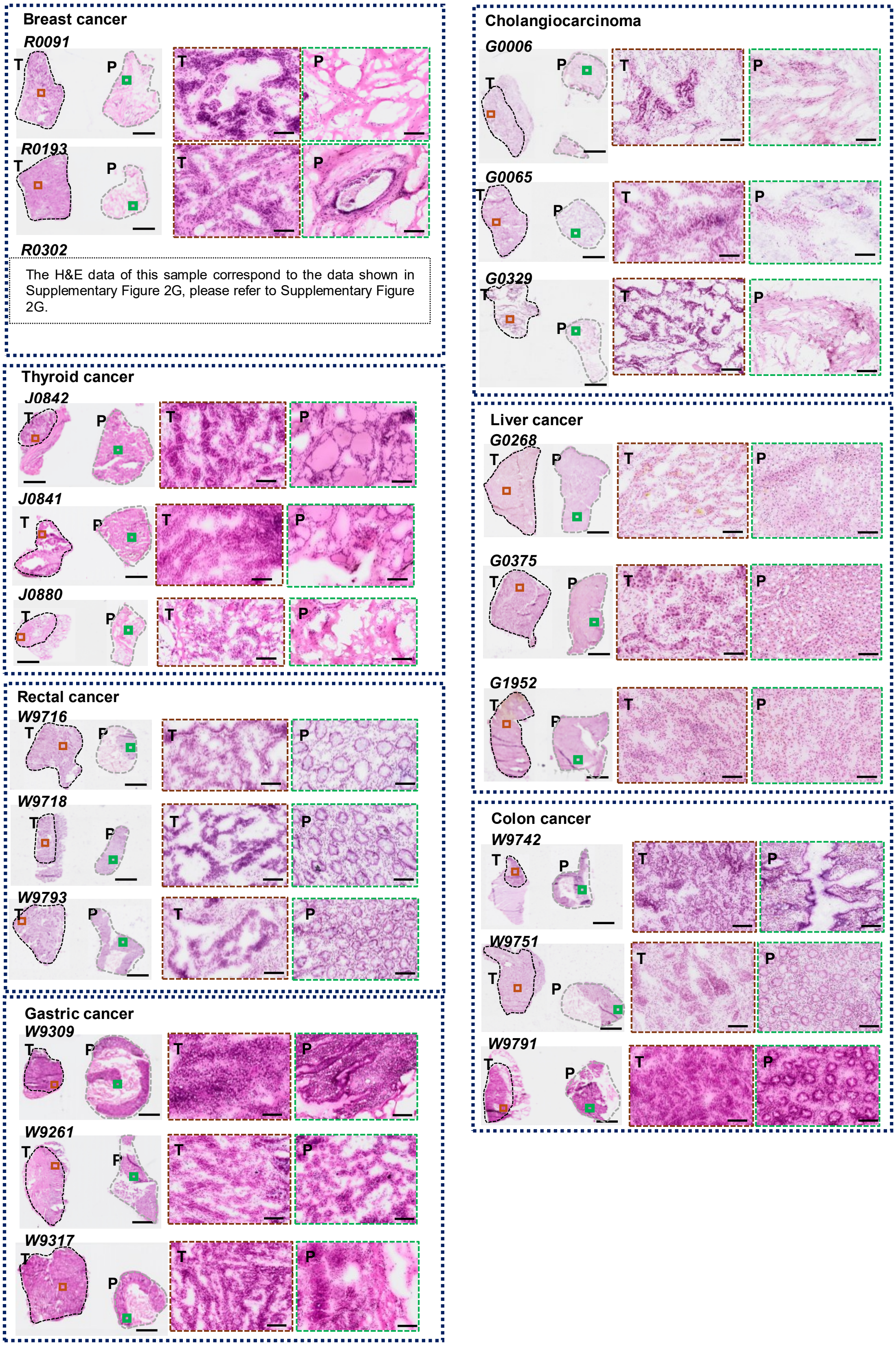

Supplementary Figure 9. H&E staining of clinical samples from 7 types of cancer. Columns from left to right represent 1× H&E staining (scale bar = 2 mm), 20× H&E staining of the magnified tumor tissue region (red box; scale bar = 100 μm), and paracancerous tissue region (green box; scale bar = 100 μm).

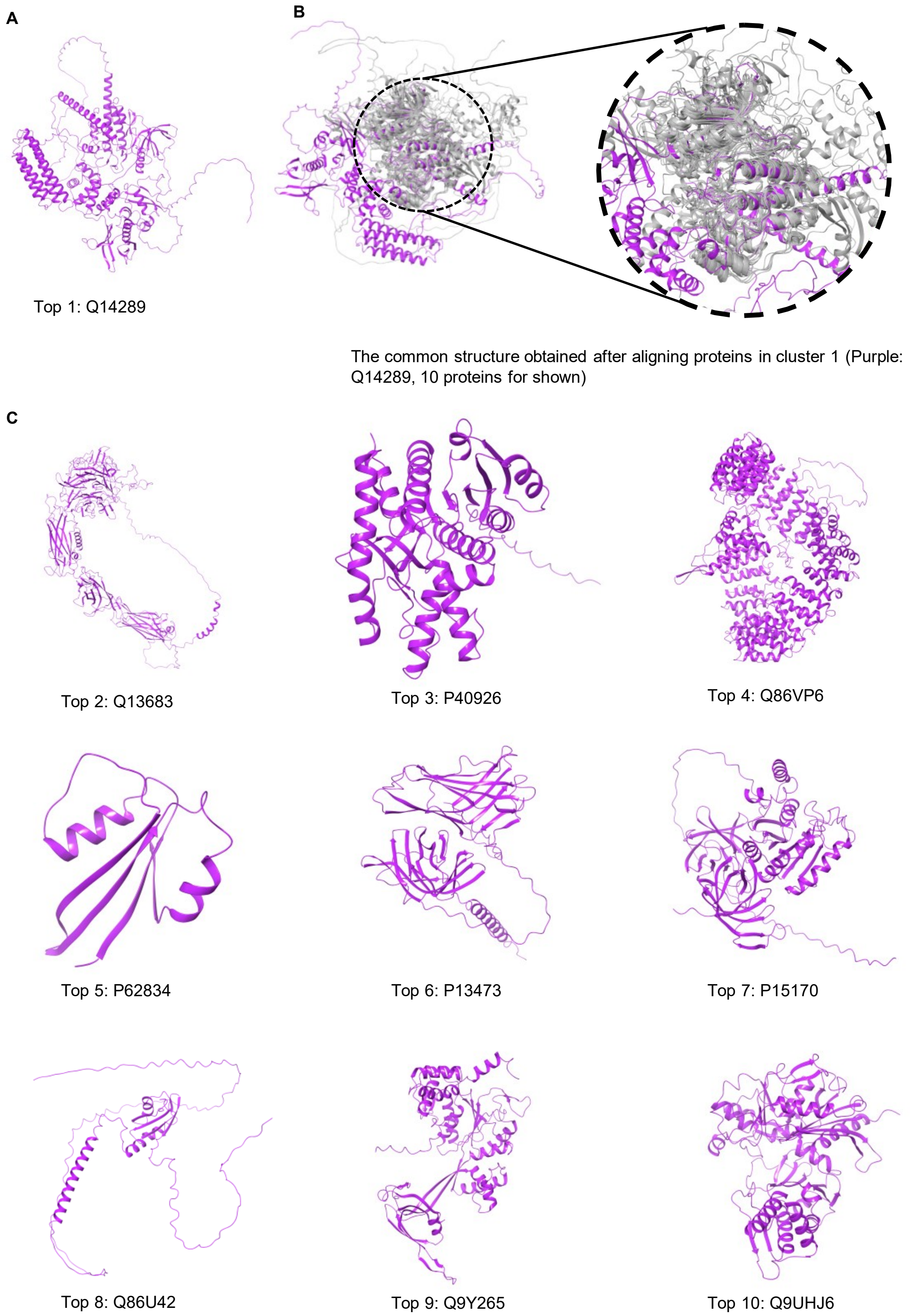

Supplementary Figure 10. The initial structures used for clustering of the top 10 clusters. (A) The initial structures used for clustering of the top 1 cluster. (B) Structure alignment of cluster 1. (C) The initial structures used for clustering of the top 2-10 clusters.

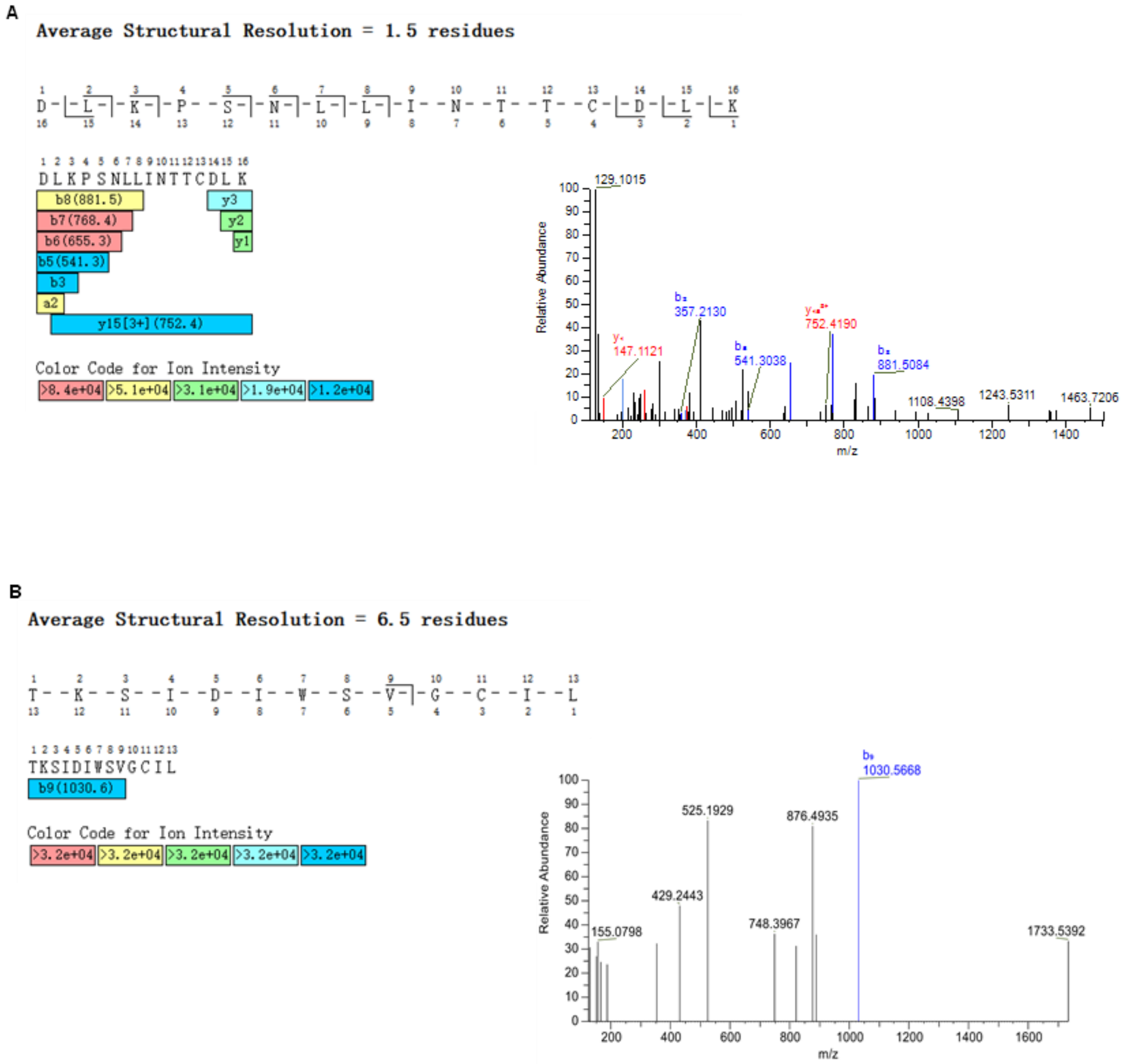

Supplementary Figure 11. The secondary mass spectra of the sequence of interest (non-reduction). The sequences of interest (A: Cys178, DLKPSNLLINTTCDLK) and (B: Cys233, TKSIDIWSVGCIL) were confirmed by proteomics approach. The average structural resolution was analyzed in detail.

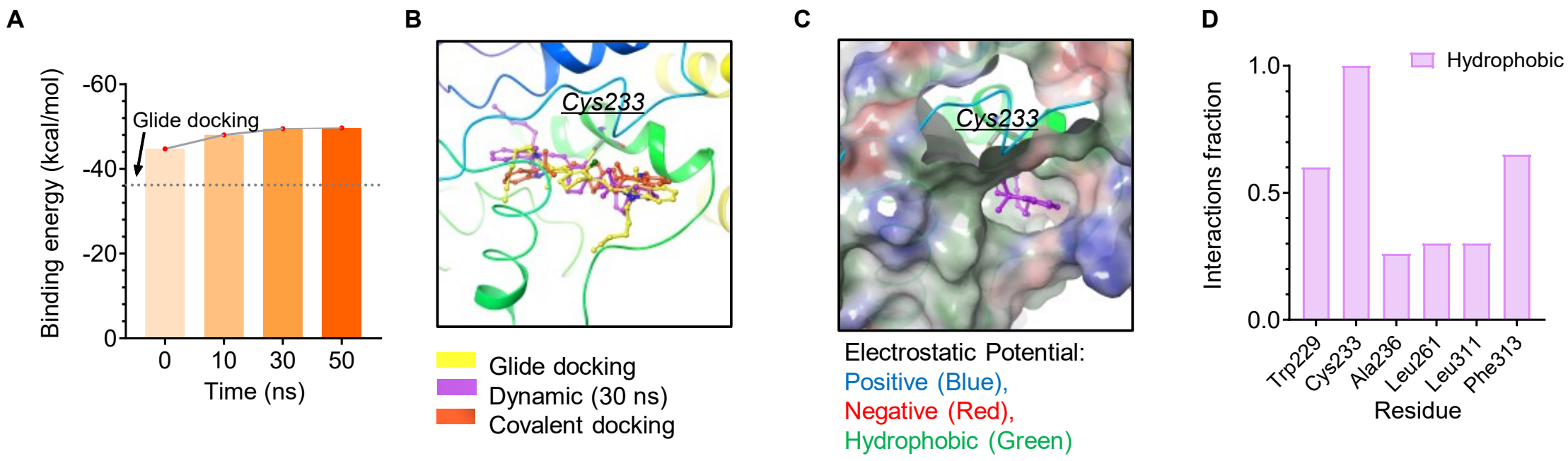

Supplementary Figure 12. Molecular dynamics simulations of CETIF6a@ERK1-Cys233. (A) Binding energies for covalent complexes during the initial 50 ns of the combination between CETIF6a and ERK1-Cys233. (B) The positional changes of molecules within the binding cavity during the transition from non-covalent docking to stable covalent docking. (C) The electrostatic potential distribution within CETIF6a-ERK1-Cys233 binding cavity. (D) The interaction between intracellular amino acids and CETIF6a. Source data are provided as a Source Data file.

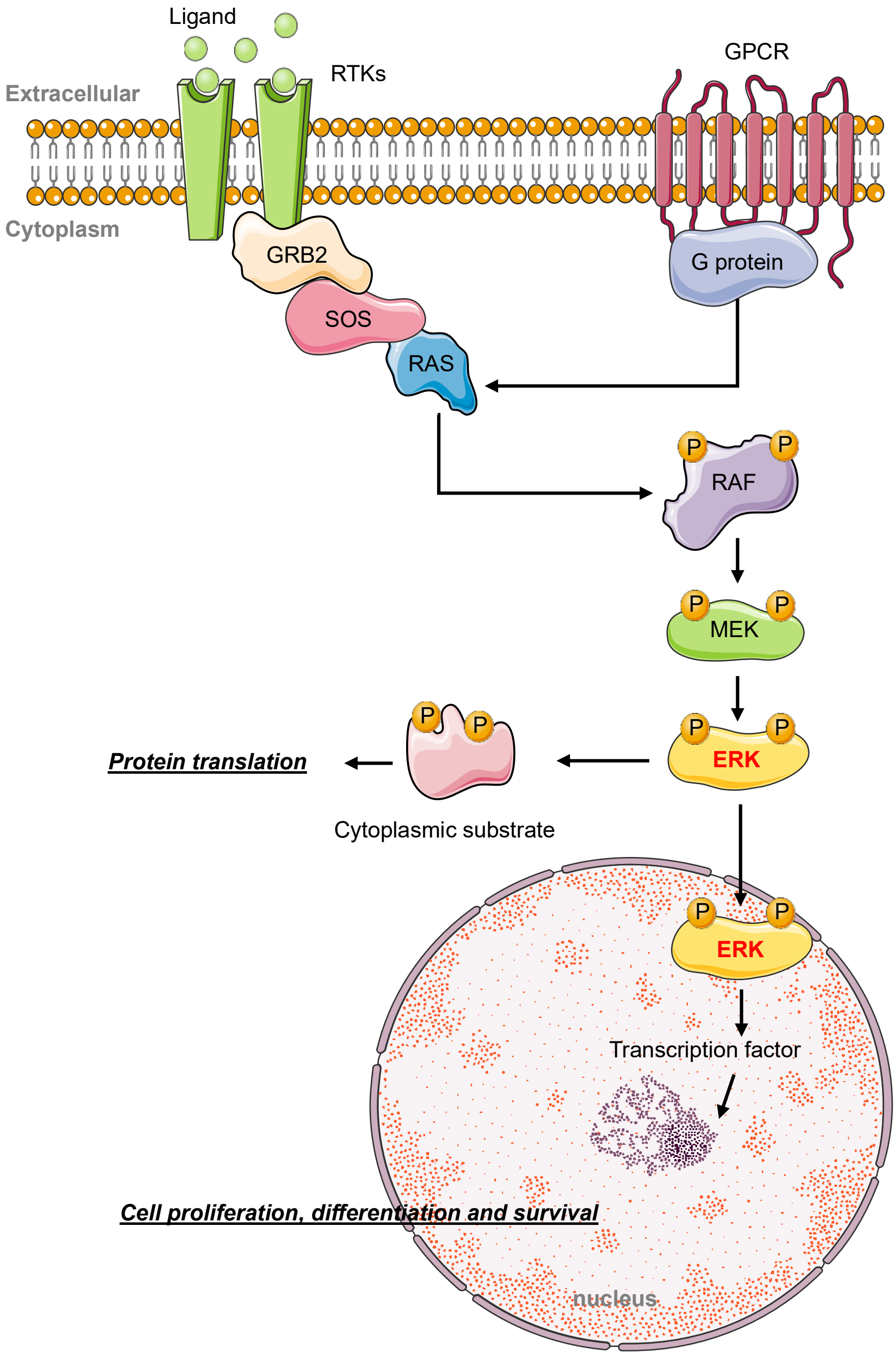

Supplementary Figure 13. Schematic diagram of the RAS-RAF-MEK-ERK pathway. Elements in this figure were adapted from Servier Medical Art, licensed under CC BY 4.0.

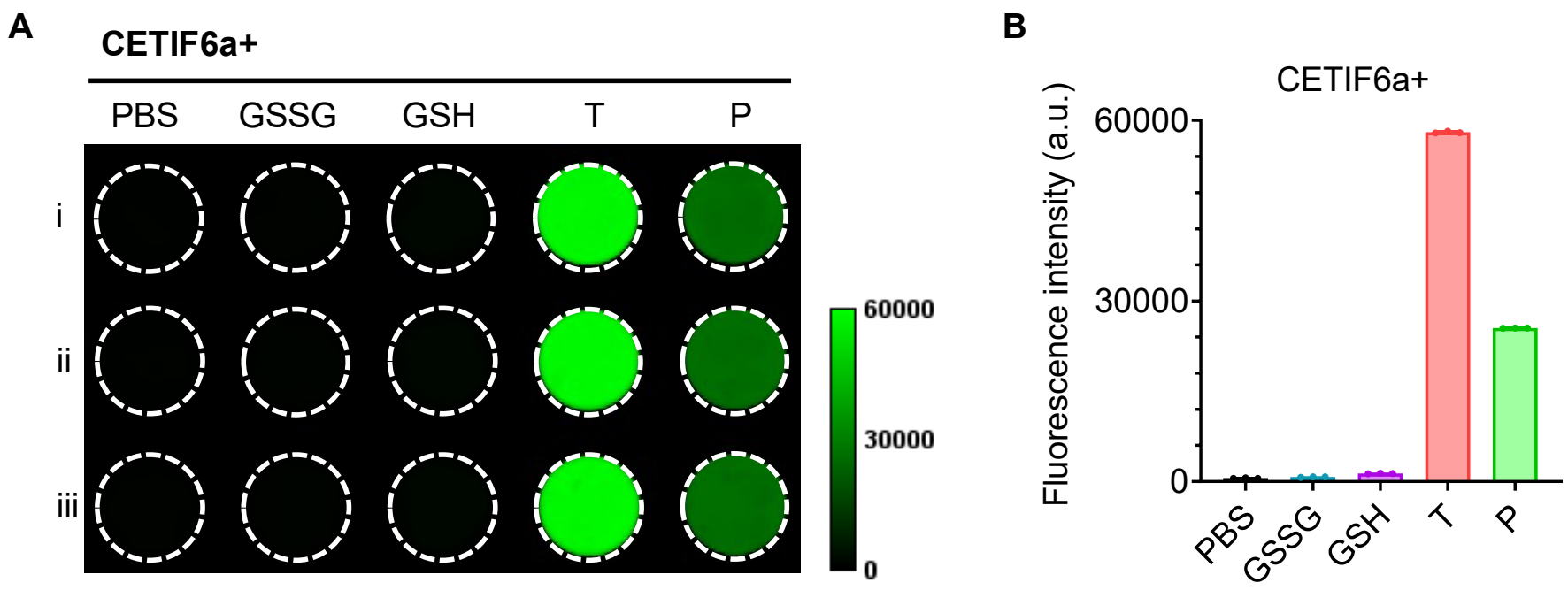

Supplementary Figure 14. The influence of cellular metabolites on brightness. (A) The fluorescence intensity after CETIF6a binds to glutathione (GSH: reduced glutathione, GSSG: oxidized glutathione, n = 3 samples per group). (B) Statistical comparison of the fluorescence in panel A (mean ± SEM). Source data are provided as a Source Data file.

Supplementary Tables

Supplementary Table 1. Clinical sample information.

ID	Gender	Age	Diagnosis	Tumor size	Classification	Grading	Staging
F0035	Female	56-60	Cervical cancer	2.5*2.2*1.1	Squamous cell carcinoma	III	T2N0
F0124	Female	56-60	Cervical cancer	2.5*2*2	Squamous cell carcinoma	II	T1N0
F0154	Female	36-40	Cervical cancer	3*2.5*1.3	Squamous cell carcinoma	II	T1N0
F0172	Female	36-40	Cervical cancer	3.5*2.5*2	Squamous cell carcinoma	II	T1N0
F0175	Female	51-55	Cervical cancer	4*3*2.5	Squamous cell carcinoma	III	T1N0
R0091	Female	46-50	Breast cancer	3.5*3.5*2.5	Ductal adenocarcinoma	III	T3N1
R0193	Female	71-75	Breast cancer	2.5*2*2	Ductal adenocarcinoma	III	T2N1
R0302	Female	36-40	Breast cancer	3.5*2.5*1.5	Ductal adenocarcinoma	II	T2N2
R0178	Female	66-70	Breast cancer	2*1.8*1.5	Ductal adenocarcinoma	II	T1N2
R0328	Male	56-60	Breast cancer	1.8*1.5*1.2	Ductal adenocarcinoma	–	T1N1
R0418	Female	46-50	Breast cancer	3*2*1.6	Ductal adenocarcinoma	II	T2N1
R0445	Female	56-60	Breast cancer	3.5*3*2.8	Ductal adenocarcinoma	II	T2N0
R0491	Female	41-45	Breast cancer	2.2*2*1.5	Ductal adenocarcinoma	II	T2N1
G0006	Male	71-75	Cholangiocarcinoma	2.5*1.5	Adenocarcinoma	II	T2NX
G0065	Male	56-60	Cholangiocarcinoma	2*2*2	Adenocarcinoma	II	T2N0
G0329	Female	61-65	Cholangiocarcinoma	2.5*2.5*2.2	Adenocarcinoma	–	T2N0
BD64	Female	71-75	Cholangiocarcinoma	2.5*1.5*1.1	Adenocarcinoma	–	T2N1
BD67	Male	71-75	Cholangiocarcinoma	3.1*1.7*1	Adenocarcinoma	–	T2NX
BD70	Male	61-65	Cholangiocarcinoma	1.5*1.0*0.5	Adenocarcinoma	–	T1N0
BD76	Male	66-70	Cholangiocarcinoma	3*2.5*1.5	Adenocarcinoma	–	T1N0
BD85	Male	71-75	Cholangiocarcinoma	2.5*1.5*0.8	Adenocarcinoma	–	T2N0
J0794	Male	31-35	Thyroid cancer	2.1	Papillary carcinoma	–	T2N1
J0841	Female	56-60	Thyroid cancer	0.2	Papillary carcinoma	–	T1N0
J0842	Female	51-55	Thyroid cancer	1	Papillary carcinoma	–	T1N0
J0880	Female	36-40	Thyroid cancer	1.2	Papillary carcinoma	–	T1N1
G0268	Male	36-40	Liver cancer	4.7*4.5*3.2	Hepatocellular carcinoma	I	T2NX
G0375	Male	56-60	Liver cancer	7*6*5	Hepatocellular carcinoma	II	T2NX
G1952	Male	41-45	Liver cancer	8.5*7*6.5	Hepatocellular carcinoma	II	T2NX
W9716	Female	66-70	Rectal cancer	3.5*2.7*1.8	Adenocarcinoma	II	T2N0
W9718	Male	51-55	Rectal cancer	3.1*2.5*1.2	Adenocarcinoma	II	T3N1
W9793	Female	61-65	Rectal cancer	5.5*4*2	Adenocarcinoma	II	T2N0
W9742	Female	51-55	Colon cancer	3.5*3.5*2.5	Adenocarcinoma	II	T3N1
W9751	Male	46-50	Colon cancer	6*4*2	Adenocarcinoma	III	–
W9791	Female	81-85	Colon cancer	8*7*3	Adenocarcinoma	III	T3N1
W9309	Male	–	Gastric cancer	5.5*4.5*1	Adenocarcinoma	II	T4N1
W9261	Female	–	Gastric cancer	3.5*2.5*1	Adenocarcinoma	III	T4N1
W9317	Male	–	Gastric cancer	6.5*5*2	Adenocarcinoma	II	T3N2

Supplementary Table 2. Information of tissue microarray samples for immunohistochemistry.

ID$	Gender	Age	Diagnosis	Tumor size	Classification	Grading	Staging
A01/02	Female	41-45	Cervical cancer	3*2*2	Squamous cell carcinoma	II	T2N0
A03/04	Female	41-45	Cervical cancer	2	Squamous cell carcinoma	II	T1N1
A05/06	Female	61-65	Cervical cancer	2.5*1.5	Squamous cell carcinoma	II	–
A07/08	Female	51-55	Cervical cancer	2	Squamous cell carcinoma	III	T1N1
B01/02	Female	–	Cervical cancer	–	Squamous cell carcinoma	–	–
B03/04	Female	31-35	Cervical cancer	3	Squamous cell carcinoma	II	T1N1
B05/06	Female	51-55	Cervical cancer	9*3.5*1.5	Squamous cell carcinoma	I	–
B07/08	Female	41-45	Cervical cancer	5*5*4.3	Squamous cell carcinoma	II	–
C01/02	Female	26-30	Cervical cancer	3	Squamous cell carcinoma	II	T1N1
C03/04	Female	41-45	Cervical cancer	3.5*2.4*2	Squamous cell carcinoma	III	–
C05/06	Female	36-40	Cervical cancer	3.5*2.5*1	Squamous cell carcinoma	I	–
C07/08	Female	41-45	Cervical cancer	6*3*1.5	Squamous cell carcinoma	II	–
D01/02	Female	46-50	Cervical cancer	4*4*1.8	Squamous cell carcinoma	II	–
D03/04	Female	41-45	Cervical cancer	3.5*2*1	Squamous cell carcinoma	II	–
D05/06	Female	56-60	Cervical cancer	5*3*1.3	Squamous cell carcinoma	II	–

Note: $Diagram showing the array arrangement of tissue microarray samples for immunohistochemistry

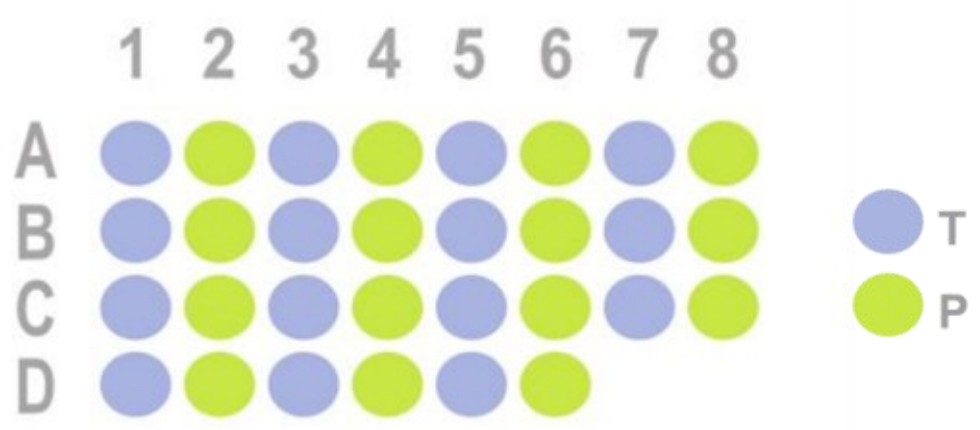

Supplementary Methods

Synthesis protocols and characterizations of CETIF

Azide:

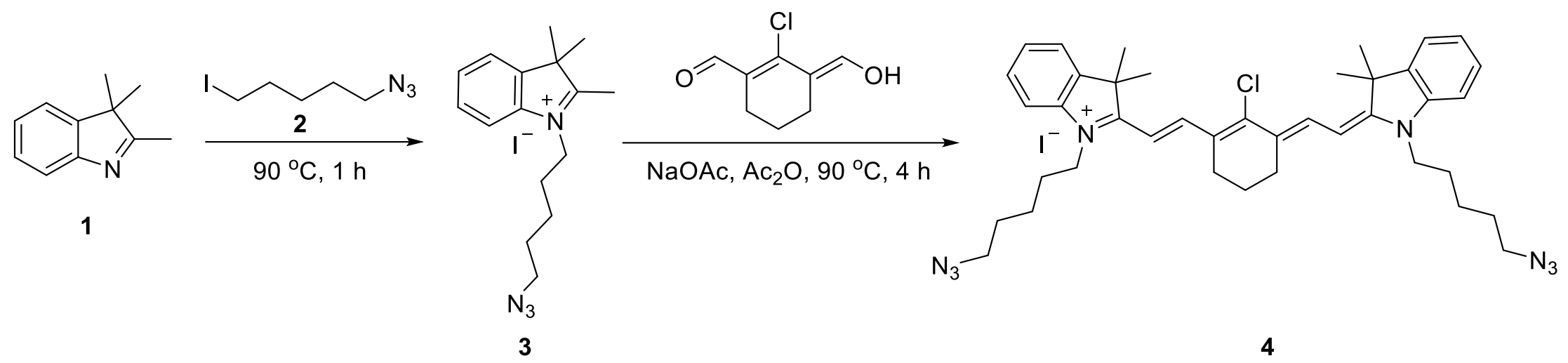

Synthesis of Compound 3: 2,3,3-Trimethylindole (compound 1) (200 mg, 0.31 mmol) and compound 2 (301 mg, 0.31 mmol) were added to the reaction tube. After a 4.5-hour reaction at 90℃, the reaction was completed. The reaction mixture was then naturally cooled to room temperature. 10 mL of ether was added to homogenize for 0.5 hours. The clear liquid was filtered off, and 5 mL of ether was added to the viscous solid obtained above. After stirring for 0.5 hours, the viscous solid was dissolved in dichloromethane, concentrated, and a purple oily viscous substance of 436 mg was obtained, with a yield of 87.2%. ^{1}H NMR (400 MHz, $CDCl_3$) δ 7.72-7.69 (m, 1H), 7.63-7.52 (m, 3H), 4.76 (t, *J* = 7.2 Hz, 2H), 3.35 (t, *J* = 6.4 Hz, 2H), 3.17 (s, 3H), 2.07-1.99 (m, 2H), 1.74-1.67 (s, 8H), 1.64 – 1.60 (m, 2H). ^{13}C NMR (100 MHz, $CDCl_3$) δ 195.93, 141.61, 140.90, 130.23, 129.65, 123.40, 115.53, 54.72, 50.99, 49.93, 28.43, 27.69, 24.02, 23.26, 17.34. HRMS (ESI-TOF) m/z: calcd. for $[M]^+$ $C_{16}H_{23}N_4^+$ = 271.1917, Found 271.1912.

Synthesis of Compound 4: A mixture of compound 3 (0.4 g, 1 mmol), 2-chloro-3-(hydroxymethylene) cyclohex-1-enecarbaldehyde (86 mg, 0.5 mmol), and NaOAc (164 mg, 2 mmol) in acetic anhydride (10 mL) was stirred at 90 °C for 4 hours. After completion, the reaction was cooled to room temperature, and the solvent was removed under reduced pressure. The resulting crude product was purified by silica gel column chromatography (DCM/MeOH = 50:1) to afford 240 mg of a green solid in 30% yield. ^{1}H NMR (400 MHz, $CDCl_3$) δ 8.33 (d, *J* = 14.0 Hz, 2H), 7.42-7.39 (m, 4H), 7.37 – 7.23 (m, 2H), 7.18 (d, *J* = 7.6 Hz, 2H), 6.25 (d, *J* = 14.0 Hz, 2H), 4.24 (t, *J* = 7.2 Hz, 4H), 3.32 (t, *J* = 6.4 Hz, 4H), 2.76 (t, *J* = 6.0 Hz, 4H), 2.02 – 1.97 (m, 2H), 1.91 – 1.87 (m, 4H), 1.71-1.67 (m, 16H), 1.62-1.56 (m, 4H). ^{13}C NMR (100 MHz, $CDCl_3$) δ 172.25, 150.54, 144.35, 142.20, 141.03, 128.90, 127.84, 125.36, 122.31, 110.93, 101.57, 51.14, 49.35, 44.94, 28.69, 28.20, 27.08, 24.28, 20.69. HRMS (ESI-TOF) m/z: calcd. for $[M]^+$ $C_{40}H_{50}ClN_8^+$ = 677.3841, Found 677.3832.

CETIF4a:

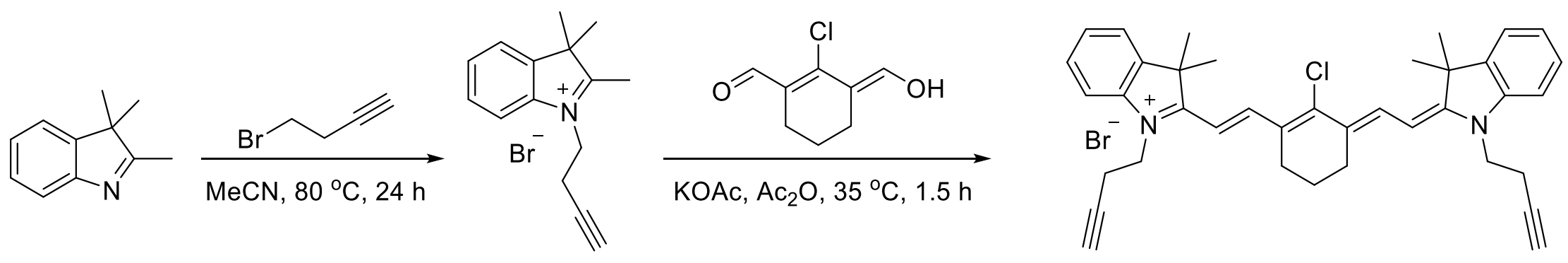

5 **6**

Synthesis of Compound 5: A mixture of 2,3,3-trimethyl-*3H*-indole (1.6 mL, 10 mmol) and anhydrous acetonitrile (10 mL) was added to a 100 mL pressure-resistant flask. Subsequently, 4-bromo-1-butyne (1.1 mL, 12 mmol) was introduced, and the reaction was heated in an oil bath at 80°C for 24 h. After cooling to room temperature, the solvent was removed under reduced pressure. Without purification, compound 5 was directly introduced into the next reaction step.

Synthesis of Compound 6: Compound 5 (50 mg, 0.15 mmol) and 2-chloro-3-(hydroxymethylene)-1-cyclohexene-1-carbaldehyde (12 mg, 0.07 mmol) were added to a 10 mL reaction flask. Potassium acetate (10 mg, 0.1 mmol) and acetic anhydride (1 mL) were then introduced, and the mixture was stirred at 35°C for 1.5 h. After solvent removal under reduced pressure, the residue was purified by silica gel chromatography (dichloromethane/methanol = 100:1, v/v) to afford 32 mg of a green solid (66.6% yield). ^{1}H NMR (400 MHz, CD_3OD) δ 8.45 (d, *J* = 14.1 Hz, 2H), 7.53 (dd, *J* = 7.5, 1.2 Hz, 2H), 7.43 (ddd, *J* = 8.3, 7.3, 1.2 Hz, 2H), 7.39 – 7.35 (m, 2H), 7.29 (td, *J* = 7.4, 1.1 Hz, 2H), 6.45 (d, *J* = 14.1 Hz, 2H), 4.40 (t, *J* = 6.6 Hz, 4H), 2.86 – 2.72 (m, 8H), 2.37 (t, *J* = 2.7 Hz, 2H), 2.03 – 1.91 (m, 2H), 1.75 (s, 12H). ^{13}C NMR (100 MHz, CD_3OD) δ 173.48, 149.85, 144.07, 142.03, 141.09, 128.41, 127.09, 125.21, 122.13, 111.06, 101.88, 79.40, 71.70, 49.36, 48.25, 48.04, 47.82, 47.61, 47.40, 47.19, 46.97, 42.17, 27.11, 25.97, 20.73, 16.60. HRMS (ESI-TOF) m/z: calcd. for $[M]^+$ $C_{38}H_{40}ClN_2^+$ = 559.2875, Found 559.2872.

CETIF5a:

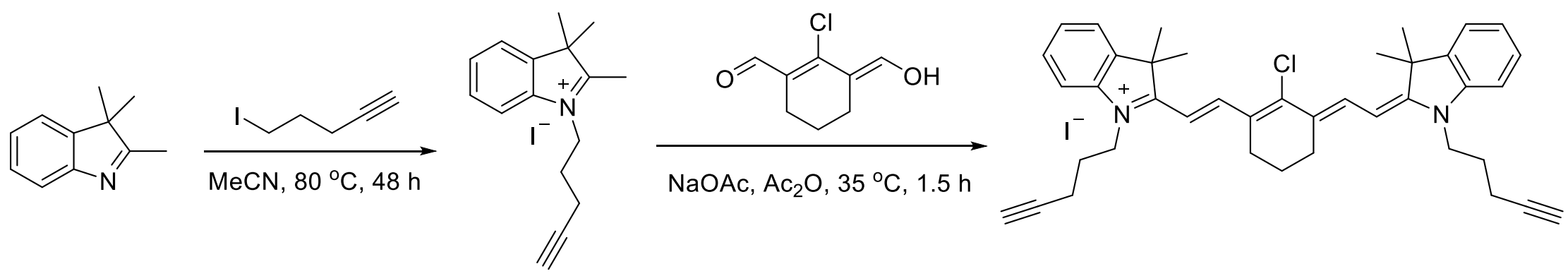

7 **8**

Synthesis of Compound 7: A mixture of 2,3,3-trimethyl-*3H*-indole (0.77 mL, 4.8 mmol) and anhydrous acetonitrile (5 mL) was added to a 35 mL pressure-resistant flask. 5-Iodo-1-pentyne (0.46 mL, 4.0 mmol) was introduced, and the reaction was heated at 80°C for 48 h. After cooling, diethyl ether was added to precipitate the product. Filtration yielded 480 mg of a pale pink solid (34.0% yield). ^{1}H NMR (400 MHz, DMSO-d_6) δ 7.99 – 7.92 (m, 1H), 7.88 – 7.81 (m, 1H), 7.69 – 7.58 (m, 2H), 4.49 (t, *J* = 7.6 Hz, 2H), 2.95 (t, *J* = 2.7 Hz, 1H), 2.84 (s, 3H), 2.42 (td, *J*

= 7.2, 2.6 Hz, 2H), 2.13 – 2.01 (m, 2H), 1.54 (s, 6H). ^{13}C NMR (100 MHz, DMSO-d_6) δ 197.56, 142.34, 141.61, 129.92, 129.44, 124.04, 115.72, 83.57, 72.95, 54.75, 47.30, 40.64, 40.48, 40.43, 40.28, 40.22, 40.02, 39.81, 39.60, 39.39, 26.53, 22.47, 15.73, 14.58. HRMS (ESI-TOF) m/z: calcd. for $[M]^+$ $C_{16}H_{20}N^+$ = 226.1590; found 226.1596.

Synthesis of Compound 8: Compound 7 (20 mg, 0.056 mmol) and 2-chloro-3-(hydroxymethylene)-1-cyclohexene-1-carbaldehyde (4.5 mg, 0.026 mmol) were reacted with sodium acetate (8 mg, 0.1 mmol) in acetic anhydride (1 mL) at 35°C for 1.5 h. Purification by silica gel chromatography (dichloromethane/methanol = 100:1, v/v) yielded 14 mg of a metallic-green solid (75.3% yield). ^{1}H NMR (400 MHz, DMSO-d_6) δ 8.28 (d, J = 14.1 Hz, 2H), 7.65 (d, J = 7.3 Hz, 2H), 7.46 (qd, J = 8.3, 1.5 Hz, 4H), 7.30 (td, J = 7.1, 1.8 Hz, 2H), 6.38 (d, J = 14.2 Hz, 2H), 4.27 (t, J = 7.3 Hz, 4H), 2.99 (t, J = 2.6 Hz, 2H), 2.72 (t, J = 6.0 Hz, 4H), 2.36 (td, J = 6.9, 2.7 Hz, 4H), 1.92 (m, J = 11.6, 5.2 Hz, 6H), 1.69 (s,12H). ^{13}C NMR (100 MHz, DMSO-d_6) δ 172.91, 148.65, 143.66, 142.49, 141.56, 129.10, 126.85, 125.71, 123.06, 111.80, 102.03, 84.01, 72.76, 55.40, 49.54, 43.32, 40.64, 40.48, 40.43, 40.27, 40.22, 40.01, 39.81, 39.60, 39.39, 27.97, 26.42, 20.81, 15.79. HRMS (ESI-TOF) m/z: calcd. for $[M]^+$ $C_{40}H_{44}ClN_2^+$ = 587.3188; Found 587.3182.

CETIF6a (Alkyne):

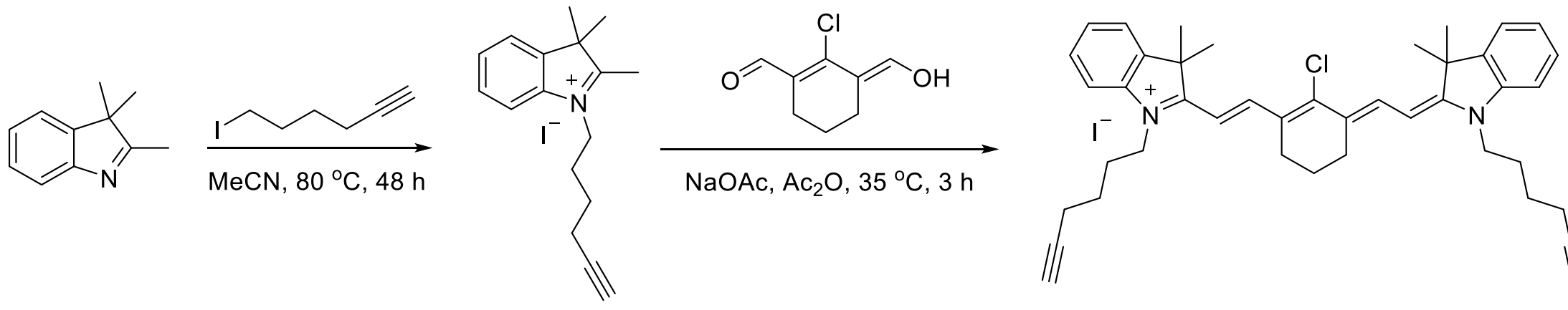

9

10

Synthesis of Compound 9: A mixture of 2,3,3-trimethyl-*3H*-indole (0.75 mL, 4.67 mmol) and anhydrous acetonitrile (5 mL) was reacted with 6-iodo-1-hexyne (0.5 mL, 3.85 mmol) at 80°C for 48 h. Ethyl acetate was added to precipitate the product, yielding 610 mg of a light gray solid (43.2% yield). ^{1}H NMR (400 MHz, CDCl$_3$) δ 7.77 – 7.75 (m, 1H), 7.61 – 7.58 (m, 3H), 4.77 (t, J = 8.0 Hz, 2H), 3.18 (s, 3H), 2.38 – 2.34 (m, 2H), 2.16 – 2.09 (m, 2H), 2.00 (q, J = 2.4 Hz, 1H), 1.81 – 1.76 (m, 2H), 1.67 (s, 6H). ^{13}C NMR (100 MHz, CDCl$_3$) δ 195.90, 141.64, 141.02, 129.61, 123.35, 115.49, 82.89, 69.93, 54.72, 49.65, 26.64, 25.14, 23.24, 17.82, 17.24. HRMS (ESI-TOF) m/z: calcd. for $[M]^+$ $C_{17}H_{22}N^+$ = 240.1747; found 240.1758.

Synthesis of Compound 10: Compound 9 (200 mg, 0.55 mmol) and 2-chloro-3-(hydroxymethylene)-1-cyclohexene-1-carbaldehyde (50 mg, 0.27 mmol) were reacted with sodium acetate (80 mg, 0.9 mmol) in acetic anhydride (10 mL) at 35°C for 3 h. Purification by silica gel chromatography (dichloromethane/methanol = 100:1, v/v) yielded 150 mg of a green solid (75.0% yield). ^{1}H NMR (400 MHz, CDCl$_3$) δ 8.34 (d, J = 14.0 Hz, 2H), 7.42 – 7.37 (m, 4H),

7.23-7.20 (m, 4H), 6.30 (d, *J* = 14.4 Hz, 2H), 4.29 (t, *J* = 7.6 Hz, 4H), 2.78 (t, *J* = 6.0 Hz, 4H), 2.36-2.32 (m, 4H), 2.05 – 1.97 (m, 8H), 1.79-1.73 (m, 16H). ^{13}C NMR (100 MHz, $CDCl_3$) *δ* 172.29, 150.47, 144.33, 142.23, 141.09, 128.87, 127.82, 125.34, 122.28, 110.99, 101.65, 83.44, 69.41, 44.66, 44.66, 28.20, 26.93, 26.24, 25.56, 20.72, 18.13. HRMS (ESI-TOF) m/z: calcd. for [M^+] $C_{42}H_{48}ClN_2^+$ = 615.3501, Found 615.3492.

DBCO:

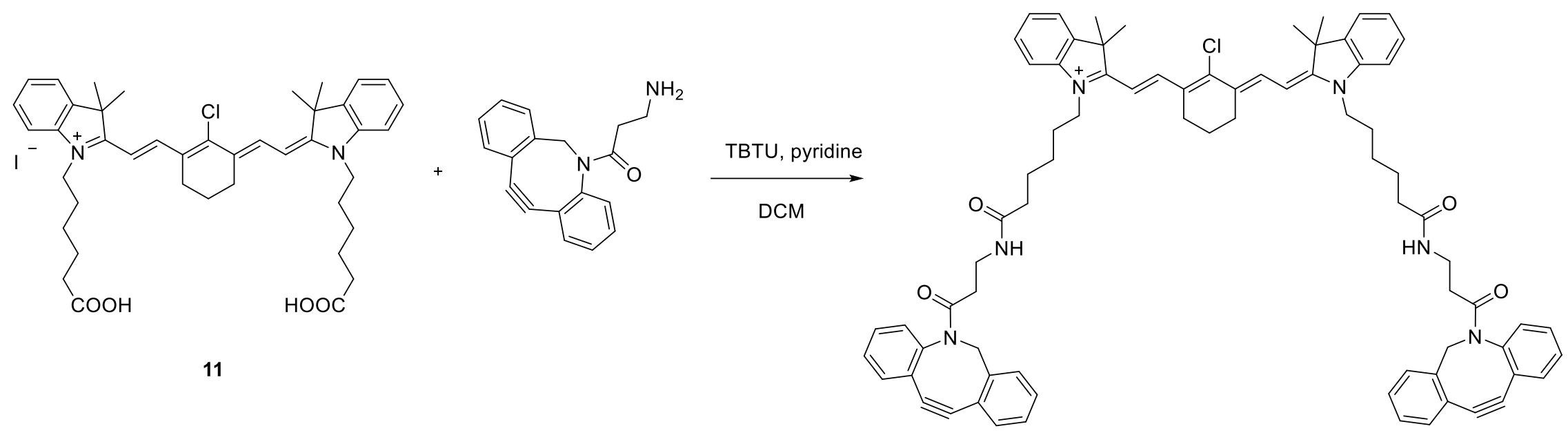

Synthesis of DBCO: Compound 11 (40 mg, 0.05 mmol) and TBTU (96.3 mg, 0.3 mmol) were dissolved in dichloromethane (5 mL). Pyridine (24 µL, 0.3 mmol) and DBCO (27.6 mg, 0.1 mmol) were added sequentially. The reaction was stirred at room temperature overnight, monitored by TLC, and purified by column chromatography (dichloromethane/methanol = 10:1) to yield 8 mg of a green solid (16% yield). ^{1}H NMR (400 MHz, CD_3OD) *δ* 8.46 (d, *J* = 14.1 Hz, 2H), 7.70 – 7.61 (m, 2H), 7.55 (d, *J* = 7.4 Hz, 2H), 7.51 – 7.40 (m, 10H), 7.38 – 7.25 (m, 8H), 7.20 (dd, *J* = 7.5, 1.4 Hz, 2H), 6.30 (d, *J* = 14.1 Hz, 2H), 5.14 (d, *J* = 14.1 Hz, 2H), 4.23 – 4.15 (m, 4H), 3.70 (d, *J* = 14.0 Hz, 2H), 3.24 (dt, *J* = 12.6, 6.2 Hz, 2H), 3.11 (ddd, *J* = 13.7, 7.8, 6.4 Hz, 2H), 2.70 (s, 4H), 2.45 (dt, *J* = 15.8, 6.0 Hz, 2H), 2.12 – 1.98 (m, 6H), 1.86 (td, *J* = 15.8, 8.1 Hz, 3H), 1.76 (t, *J* = 1.0 Hz, 12H), 1.57 (p, *J* = 7.4 Hz, 4H), 1.46 – 1.26 (m, 7H). ^{13}C NMR (100 MHz, CD_3OD) δ 174.14, 174.12, 171.70, 151.19, 148.16, 132.11, 129.06, 128.61, 128.28, 127.85, 127.50, 126.76, 125.03, 122.92, 122.17, 114.25, 107.59, 55.19, 49.29, 48.32, 48.25, 48.11, 48.04, 47.83, 47.61, 47.40, 47.19, 46.98, 35.28, 35.06, 34.12, 26.97, 26.72, 25.92, 24.95. HRMS (ESI-TOF) m/z: calcd. for $[M]^{2+}$ $C_{78}H_{81}ClN_6O4^{2+}$ = 600.2999; found 600.2987.

^{1}H-NMR of Compounds:

^{1}H-NMR of Compound 3

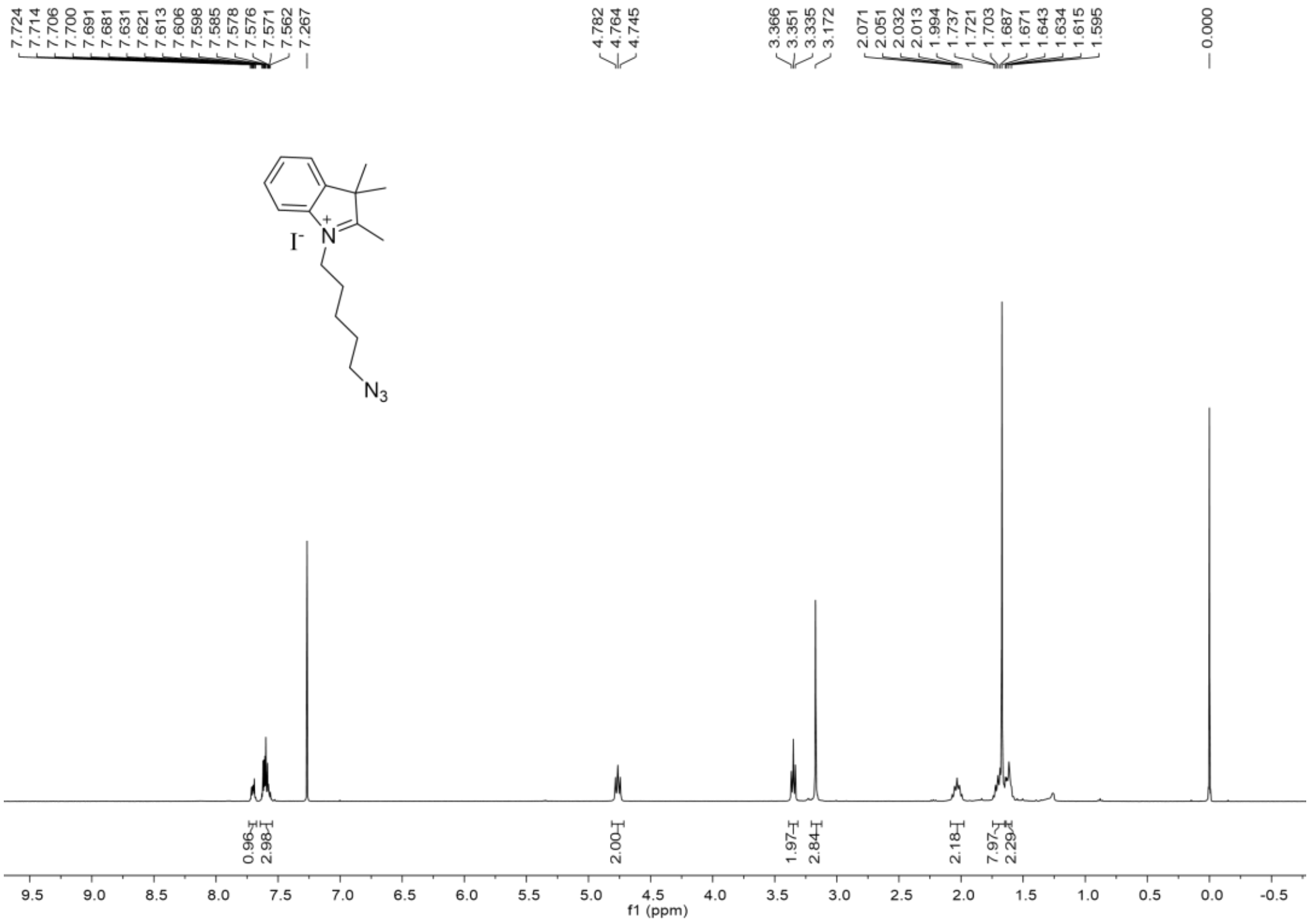

^{1}H-NMR of Compound 4

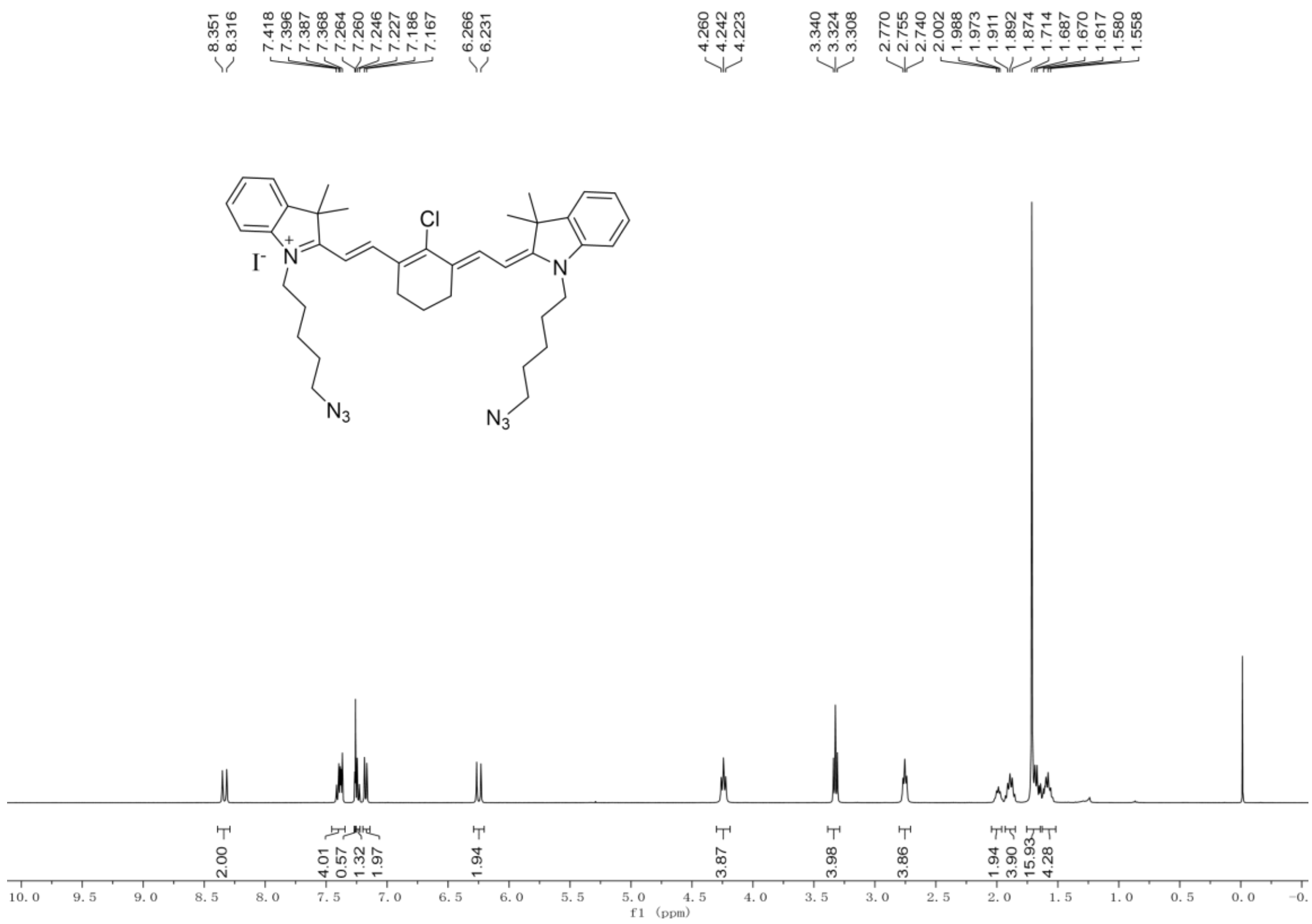

^{1}H-NMR of Compound 6

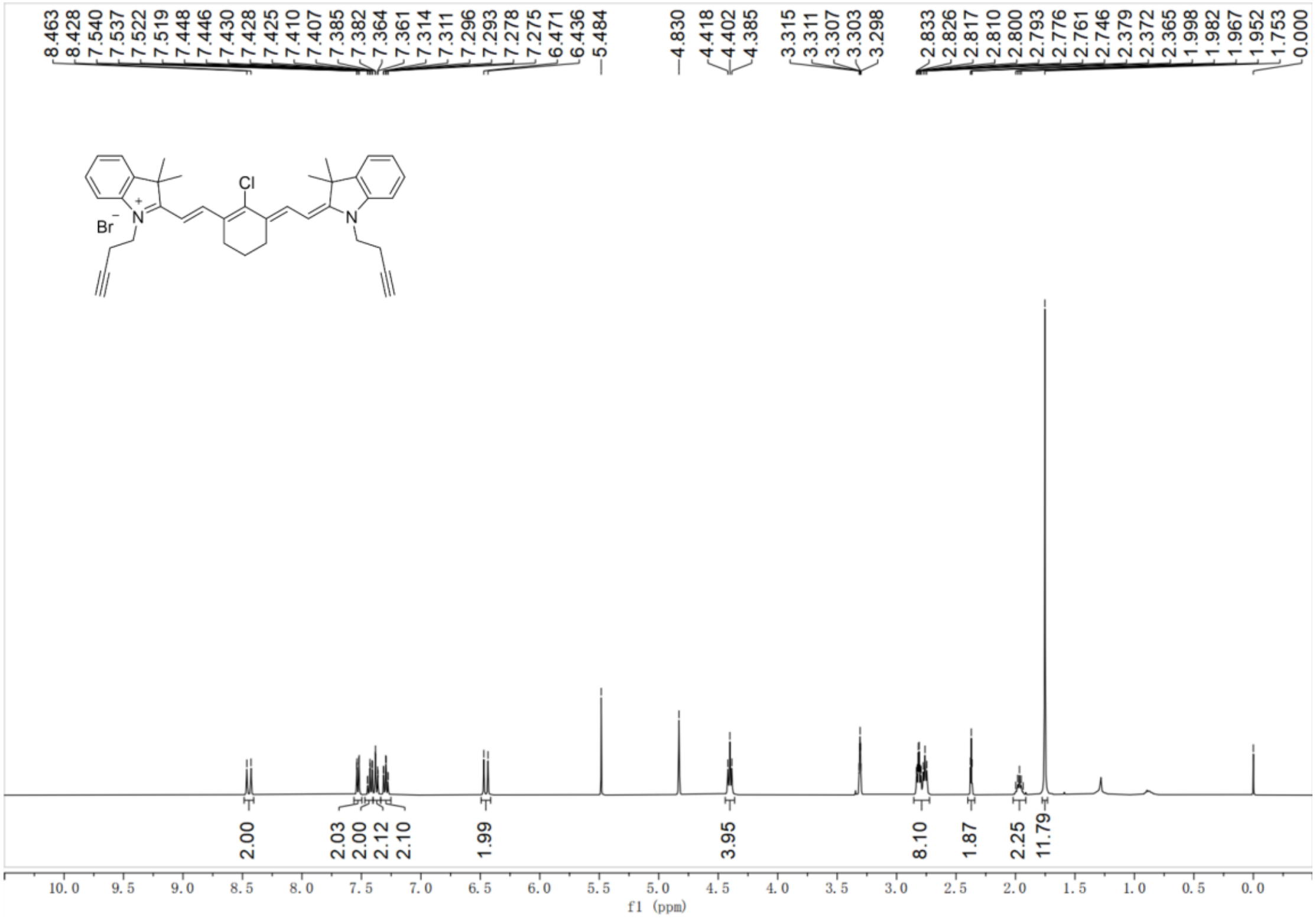

^{1}H-NMR of Compound 7

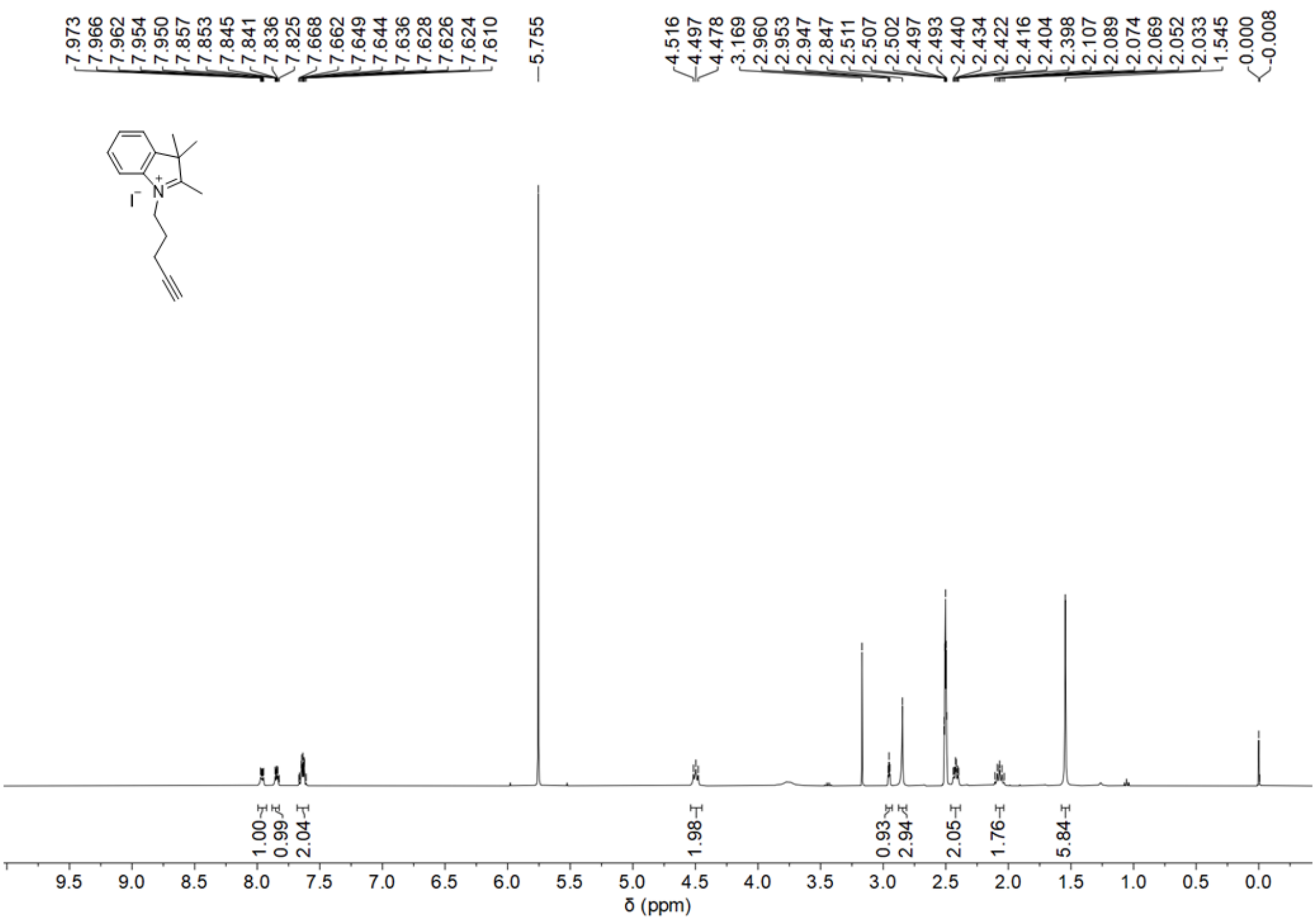

^{1}H-NMR of Compound 8

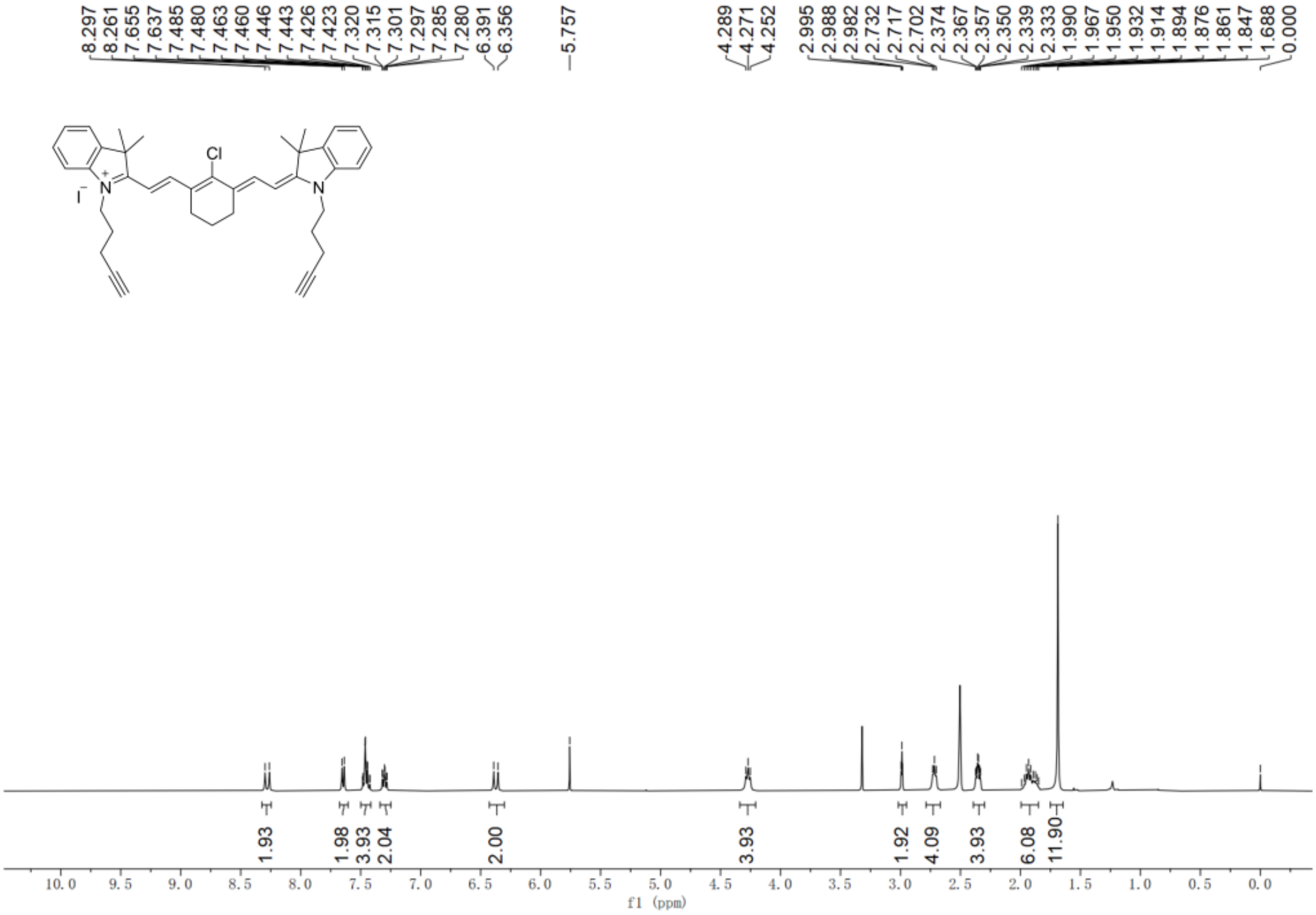

^{1}H-NMR of Compound 9

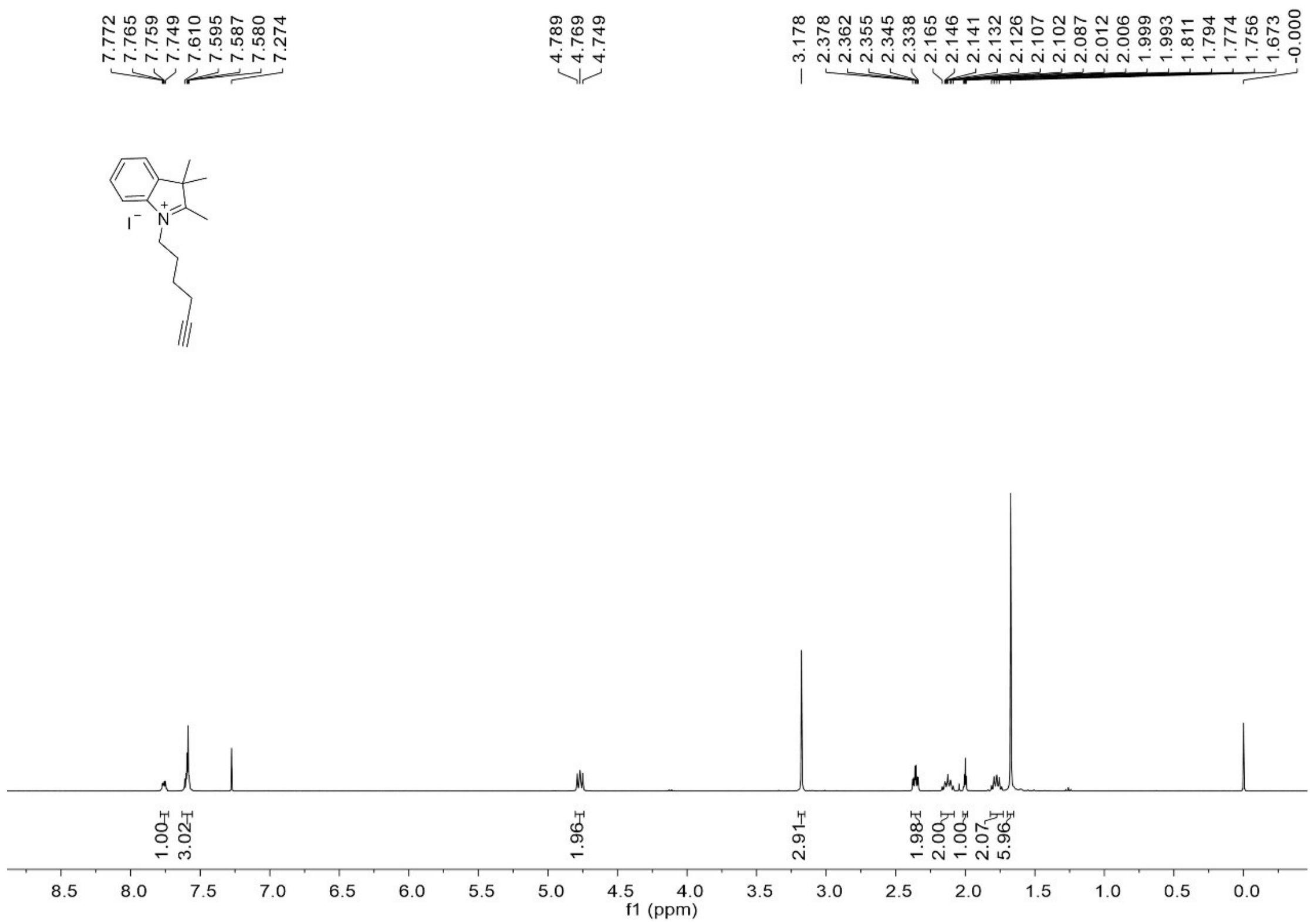

^{1}H-NMR of Compound 10

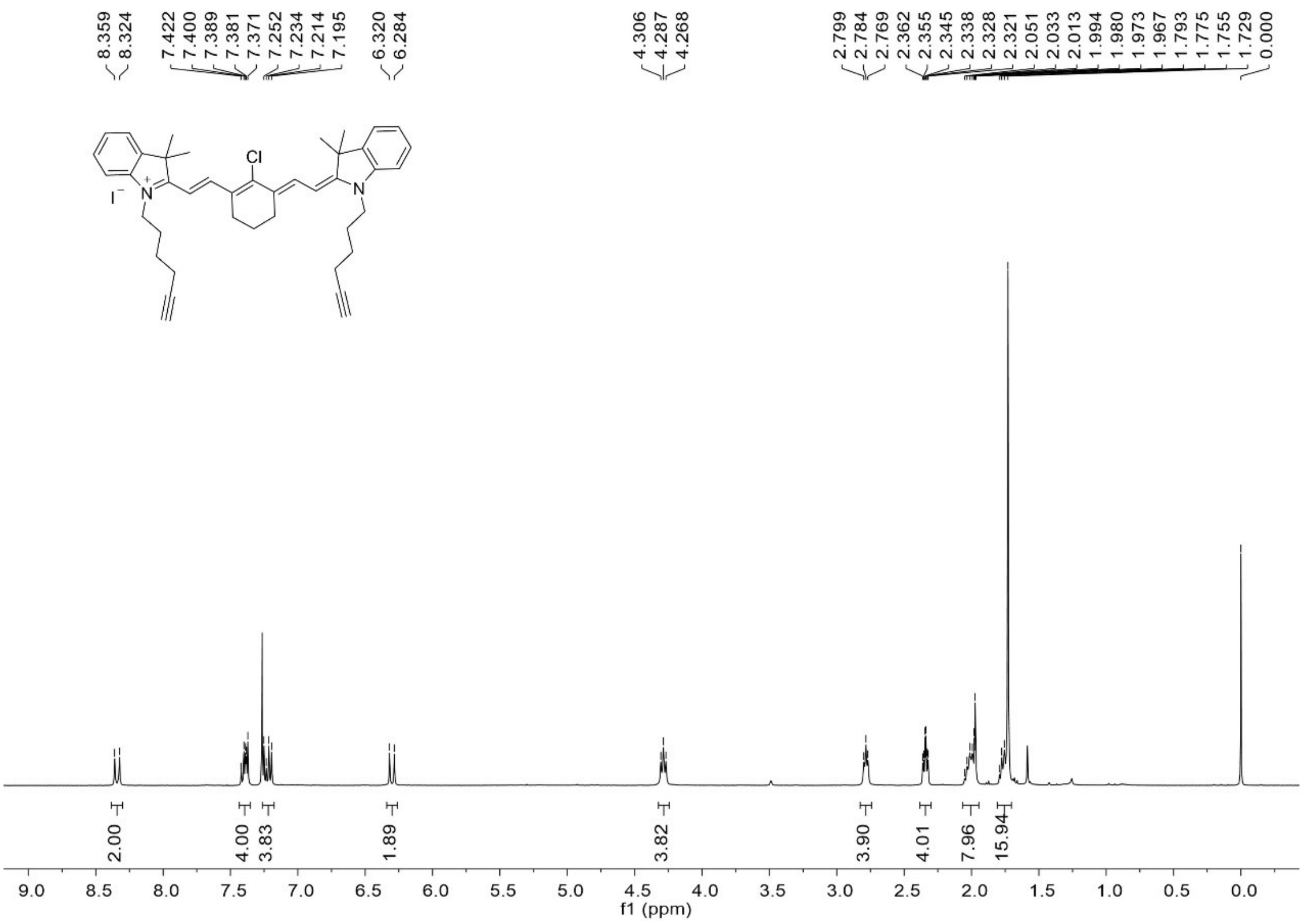

^{1}H-NMR of DBCO

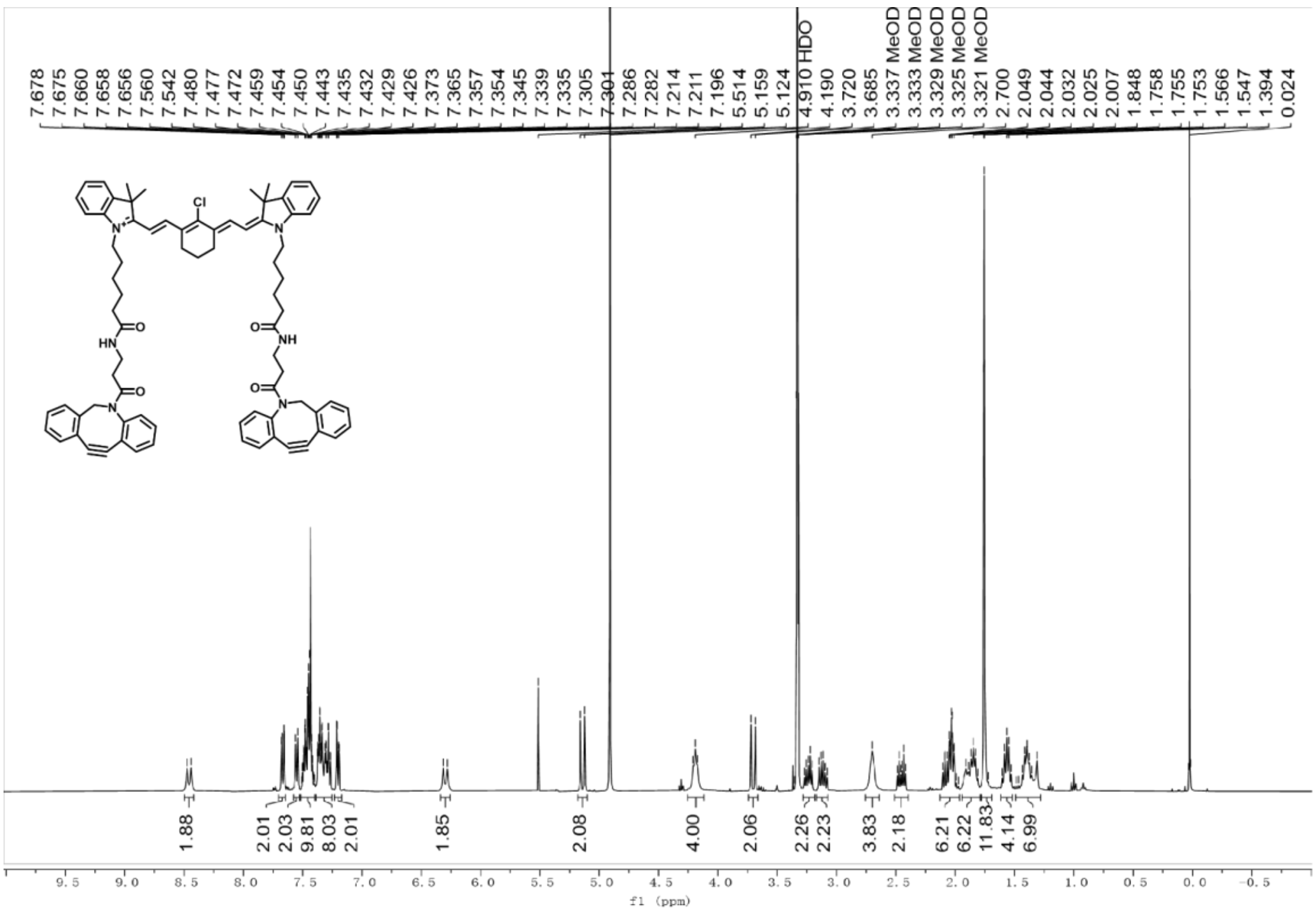

^{13}C-NMR of Compounds:

^{13}C-NMR of Compound 3

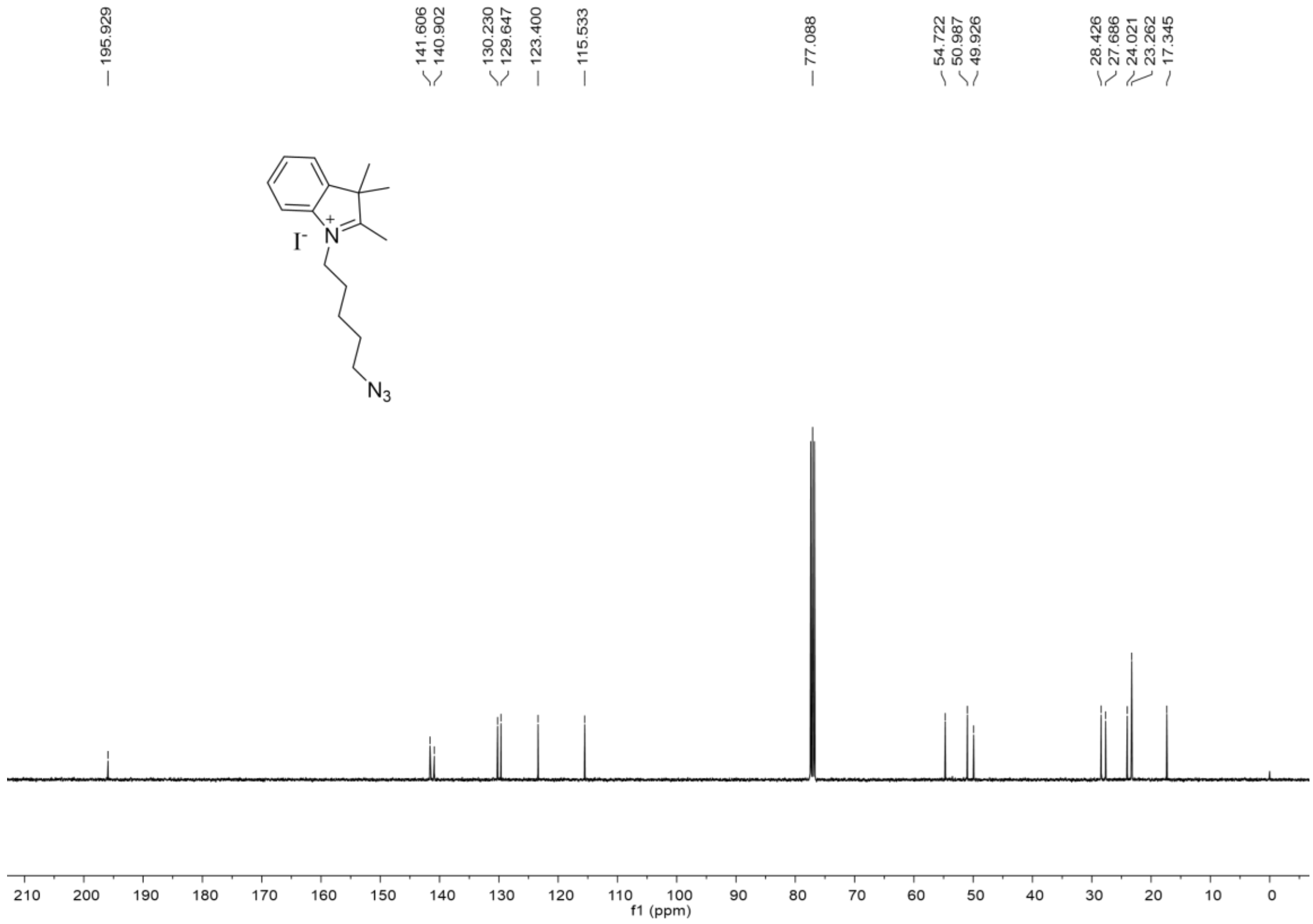

^{13}C-NMR of Compound 4

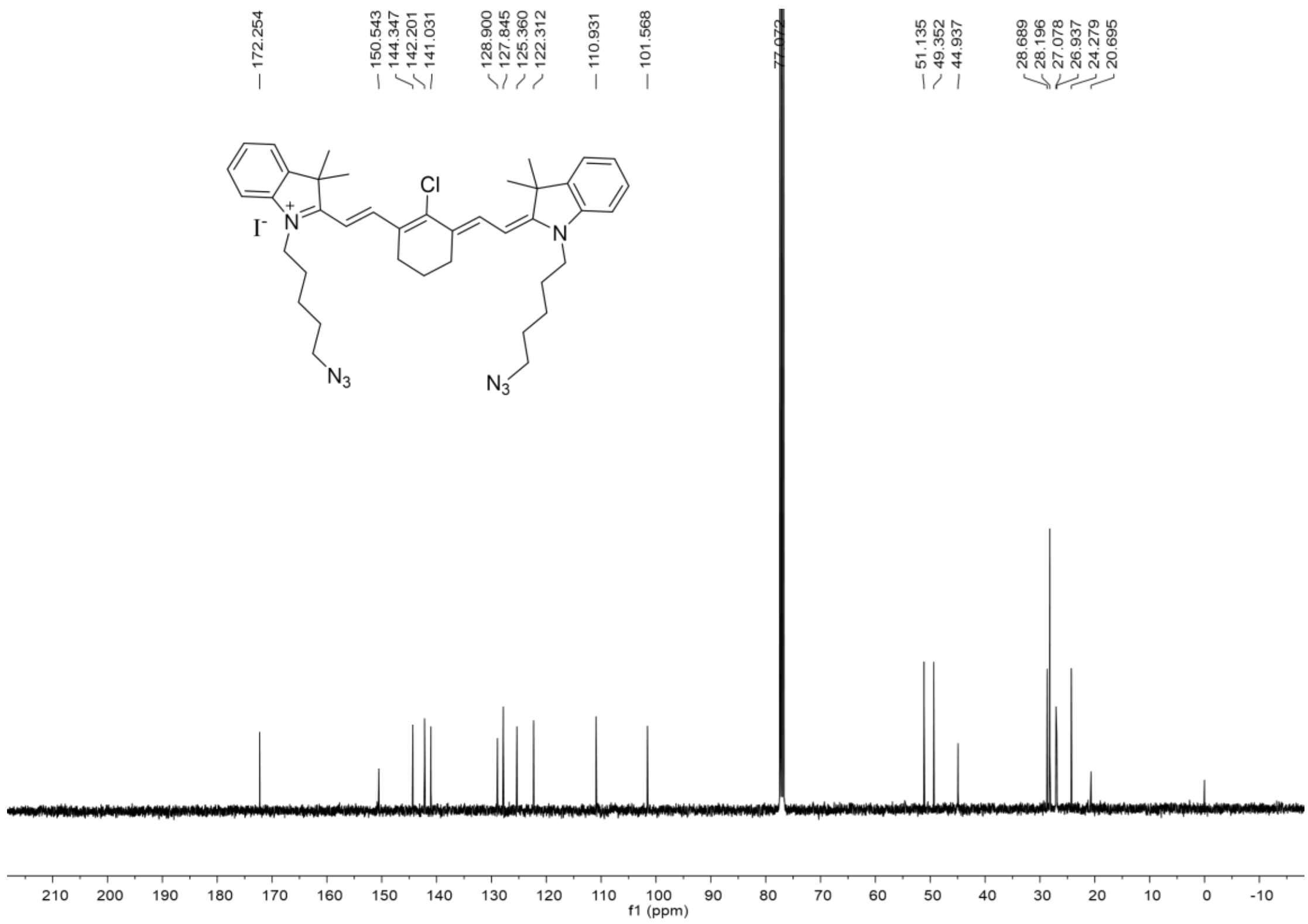

^{13}C-NMR of Compound 6

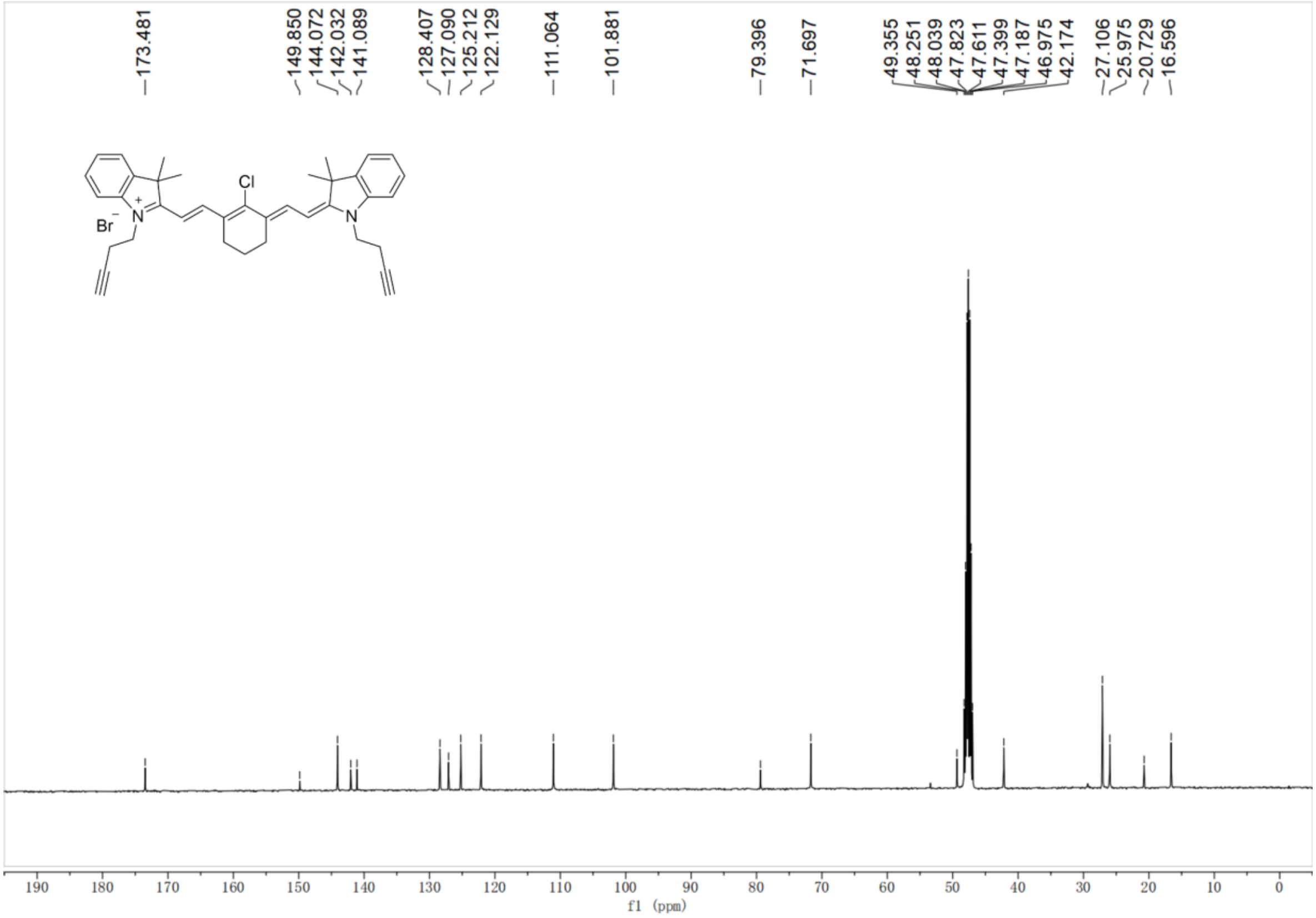

^{13}C-NMR of Compound 7

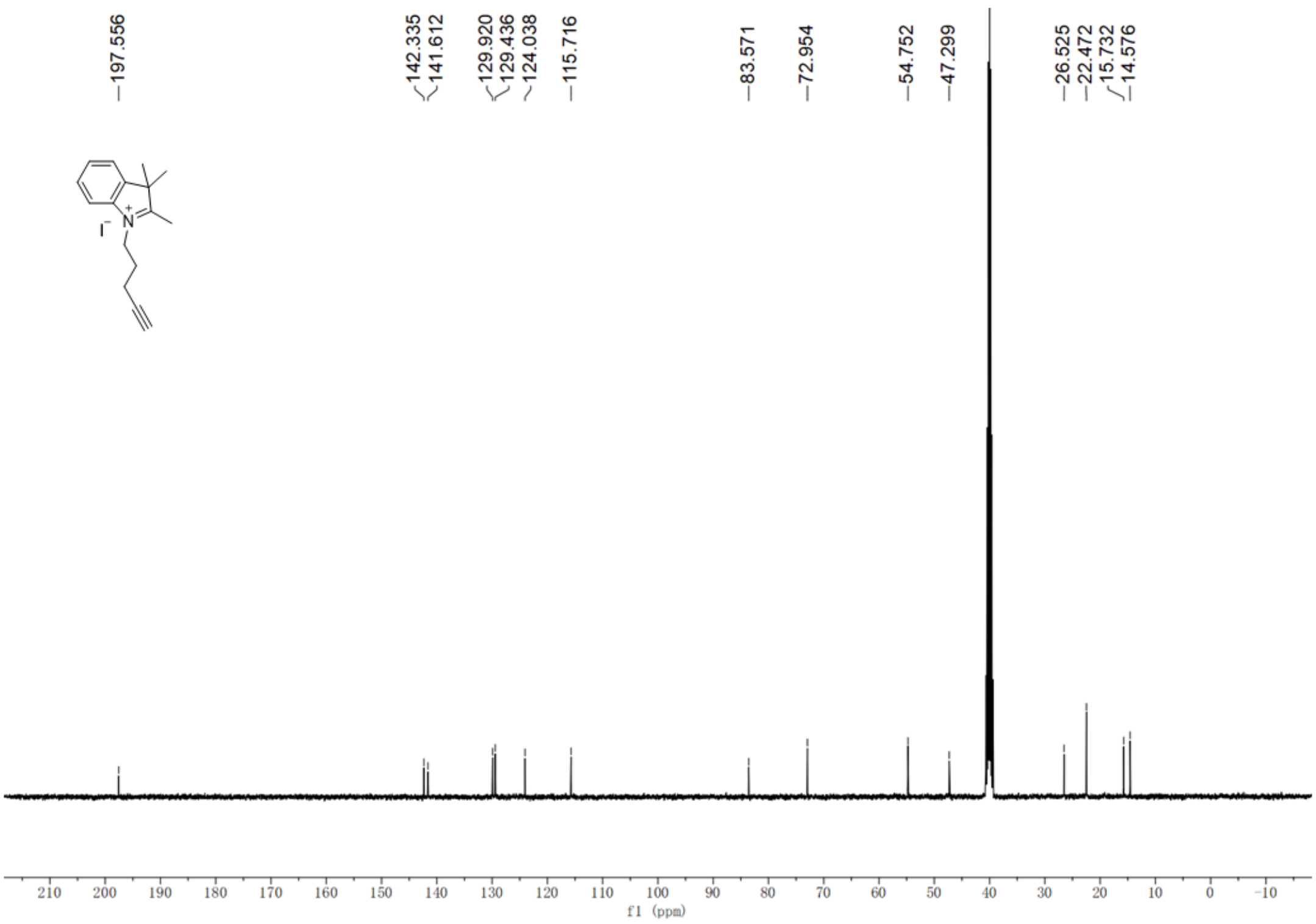

^{13}C-NMR of Compound 8

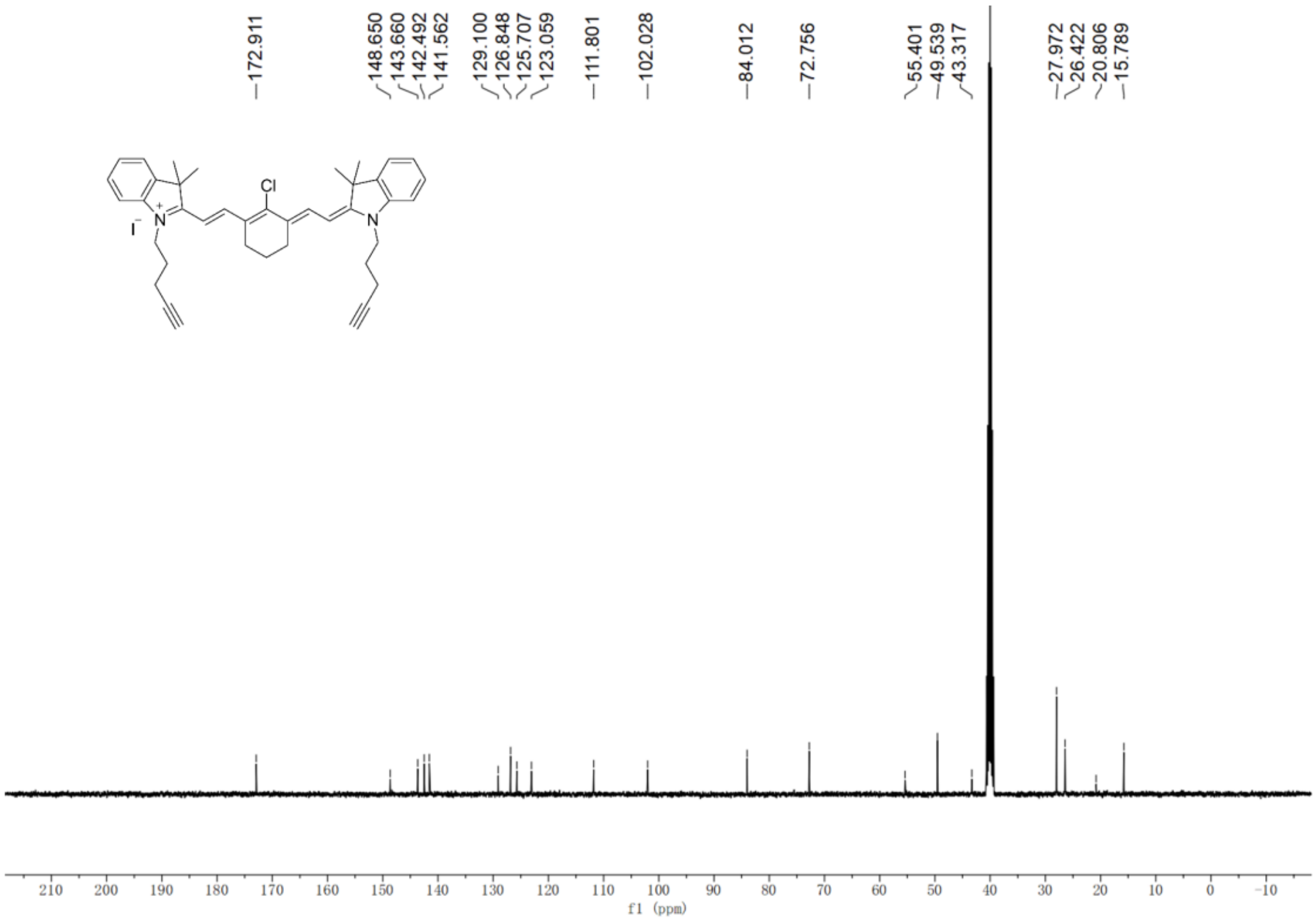

^{13}C-NMR of Compound 9

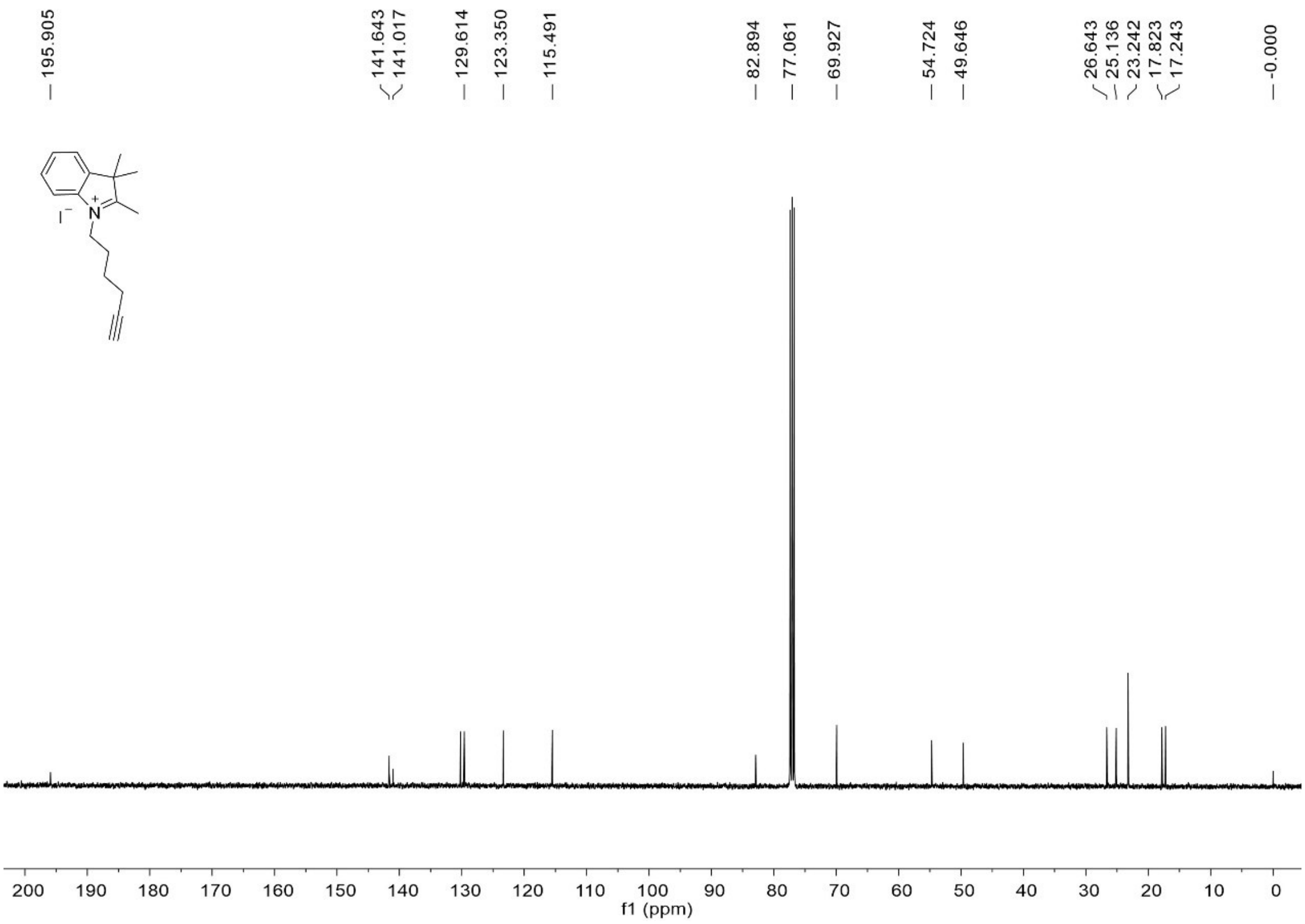

^{13}C-NMR of Compound 10

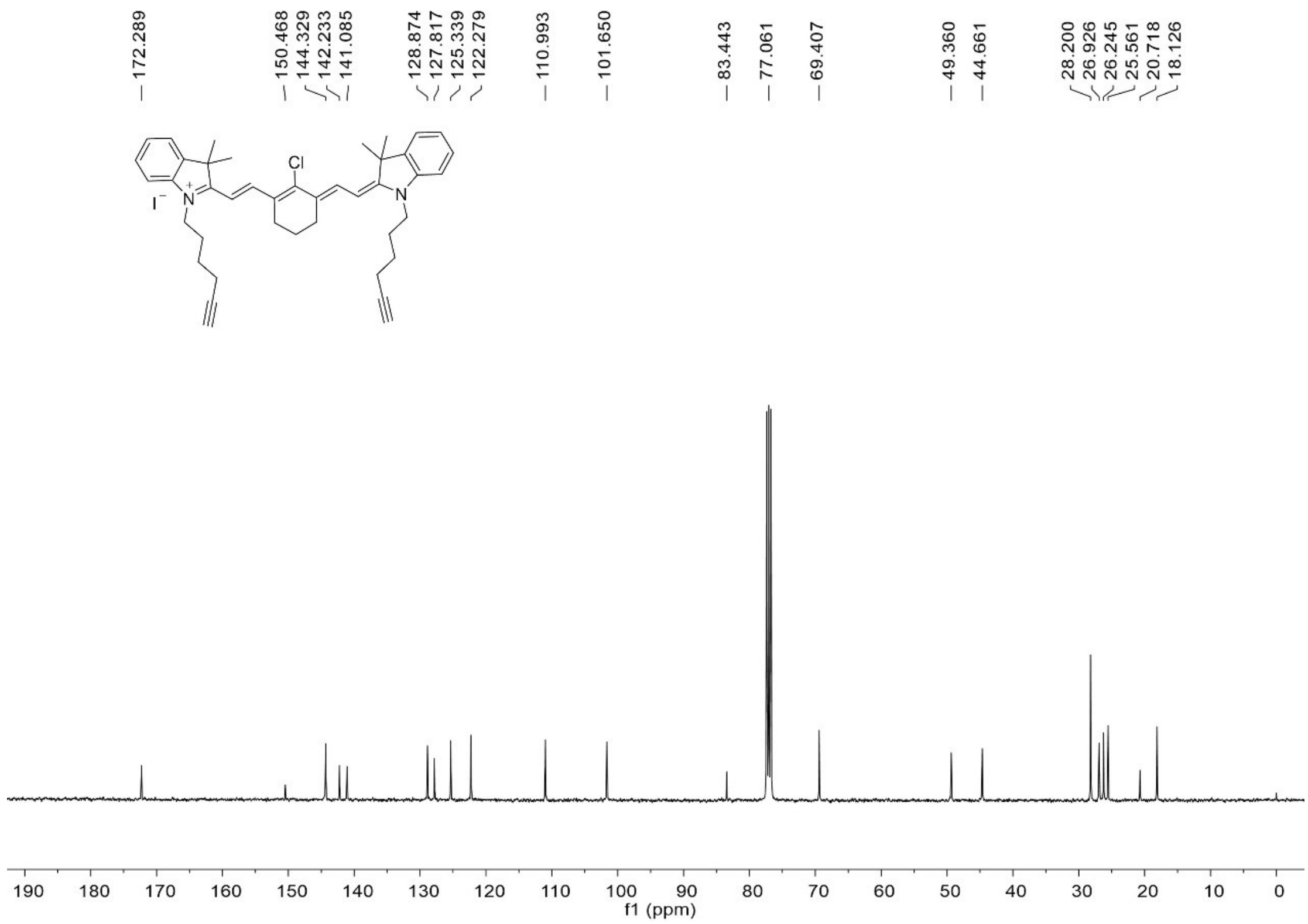

^{13}C-NMR of DBCO

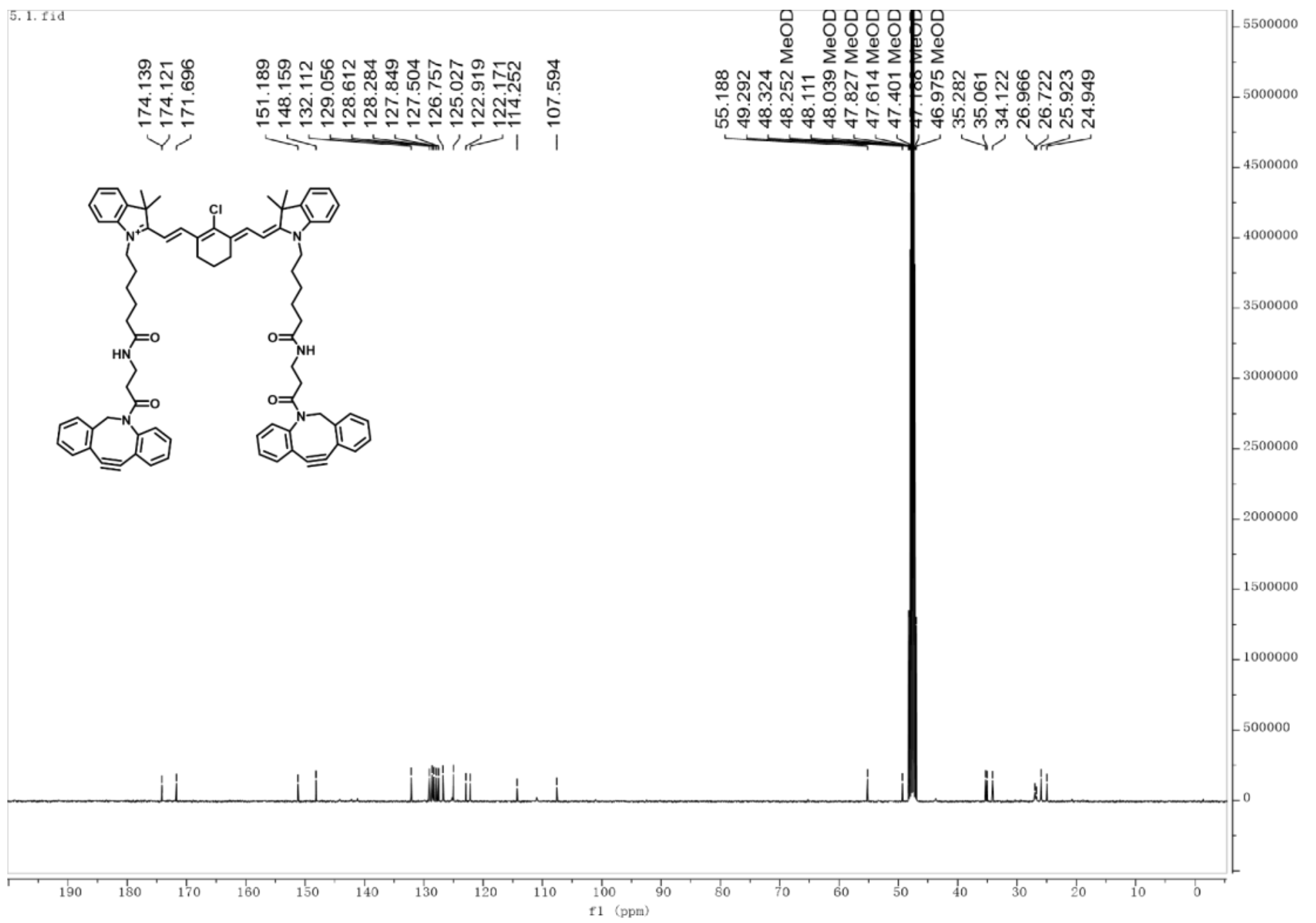

HPLC of Compounds:

HPLC of Compound 4

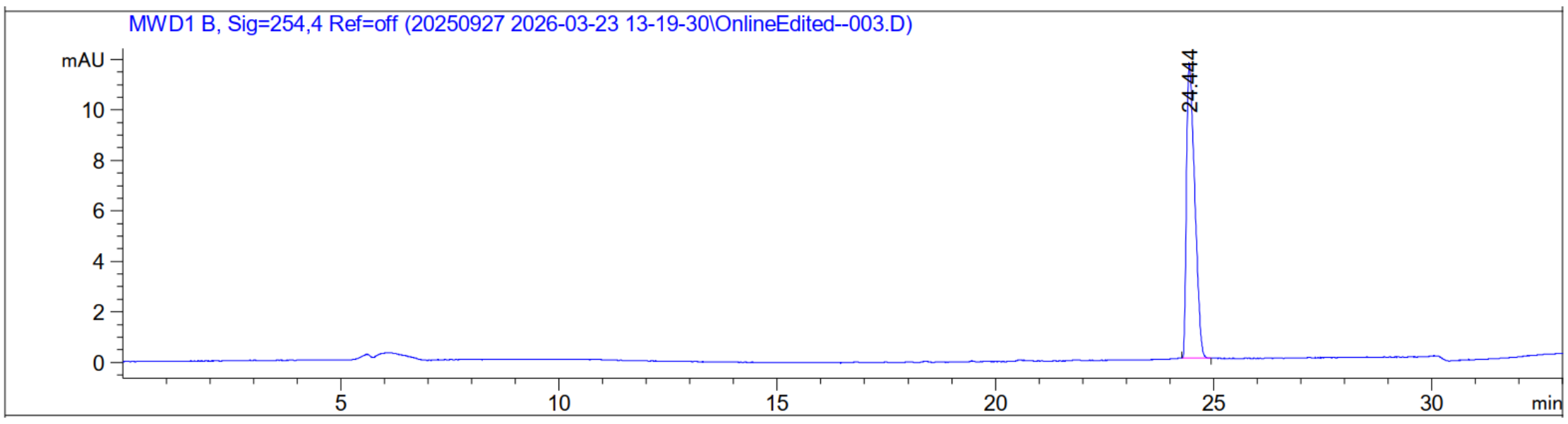

HPLC of Compound 6

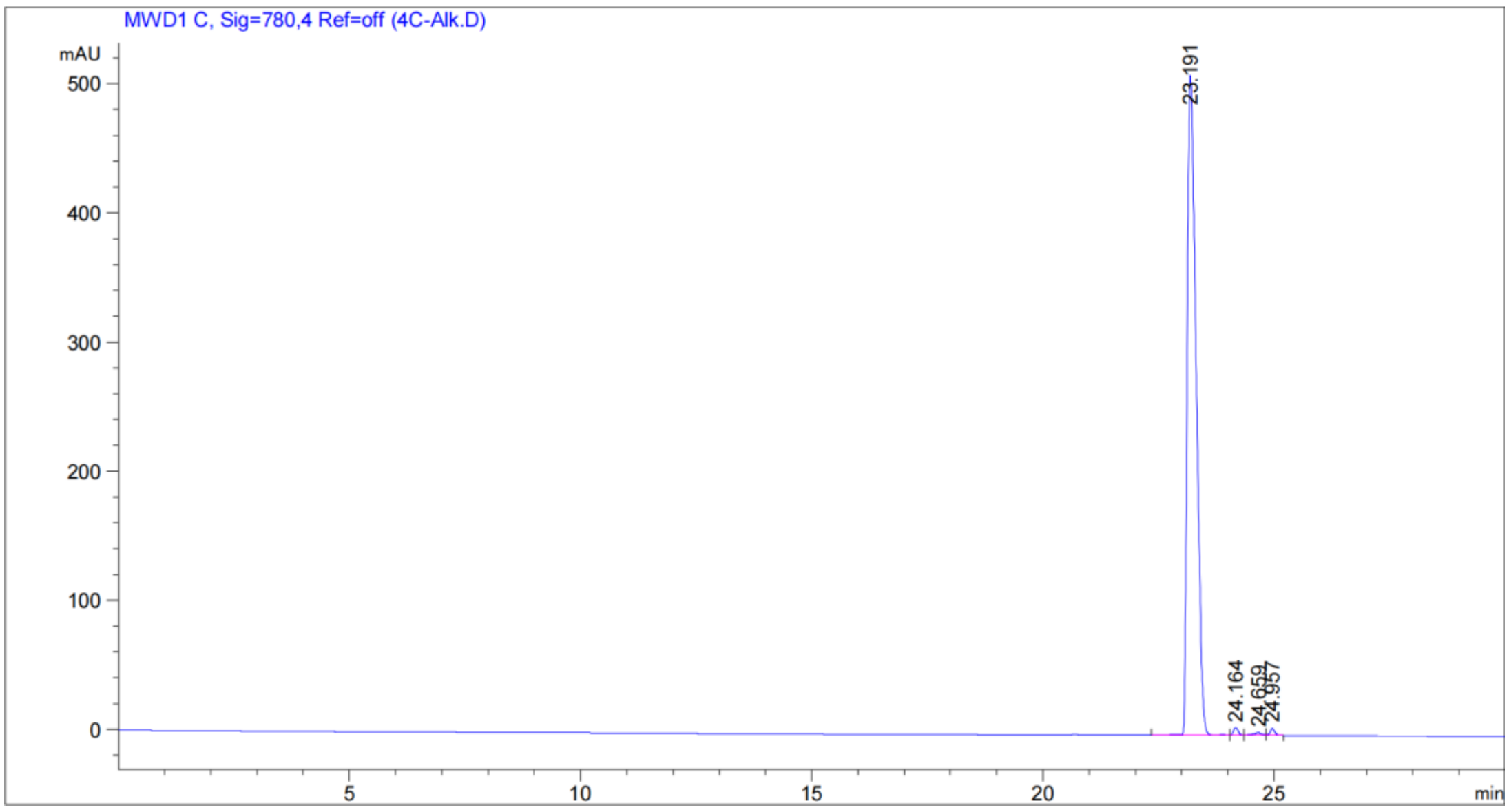

HPLC of Compound 8

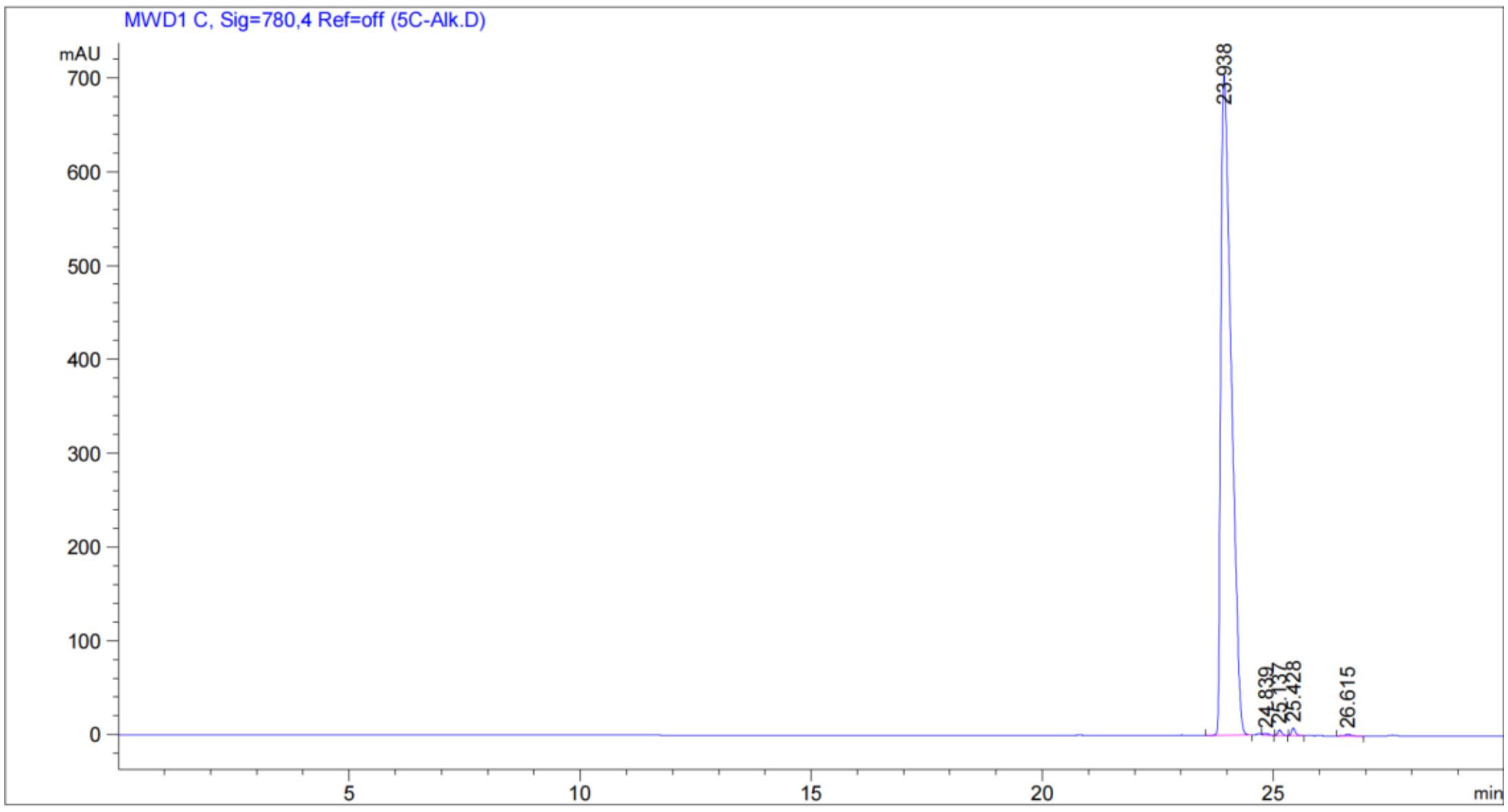

HPLC of Compound 10

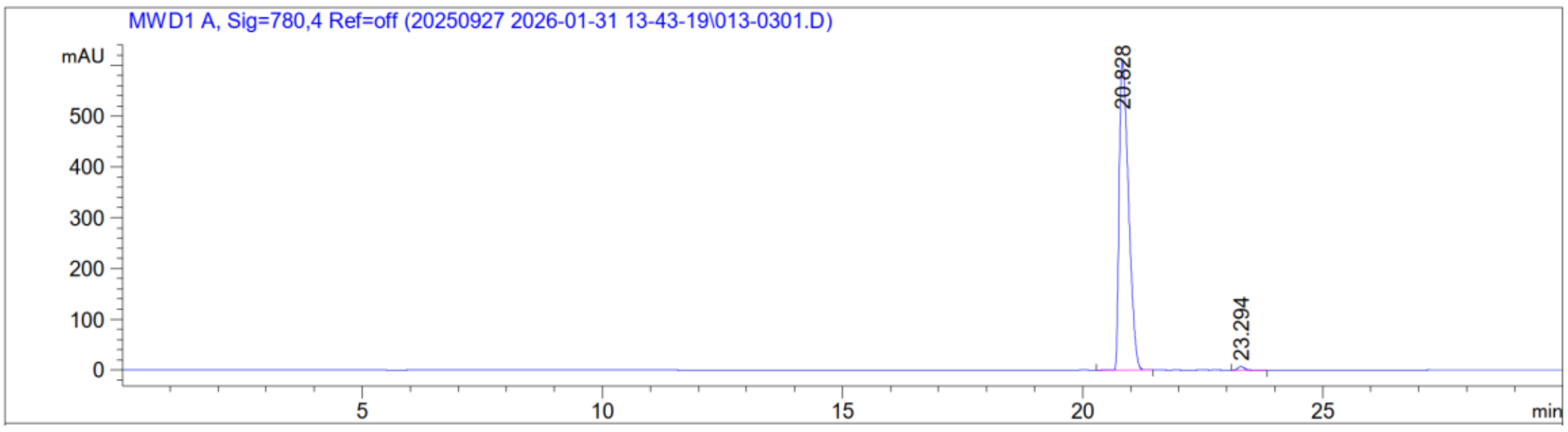

HPLC of Compound DBCO

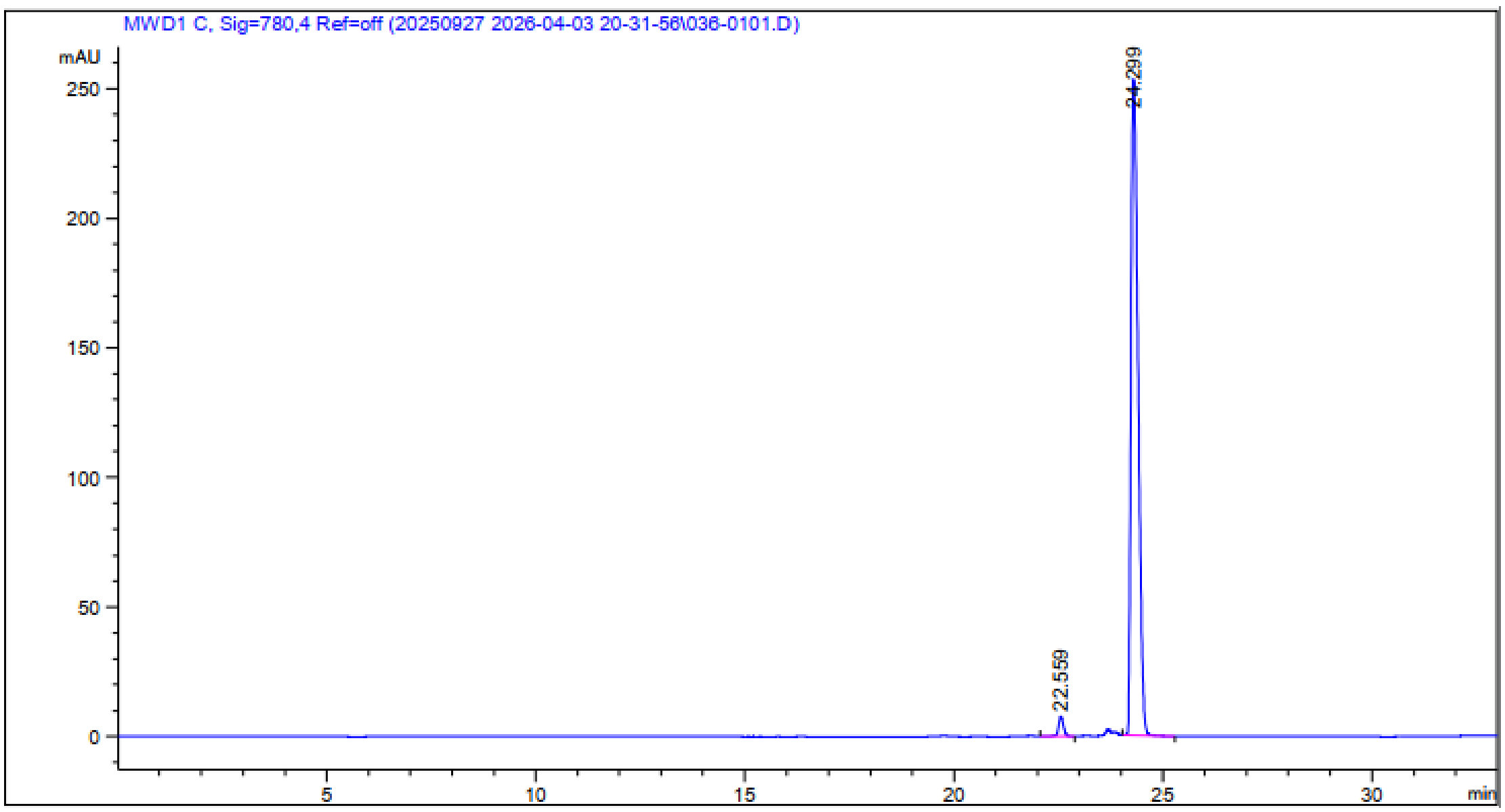

Supplement: Supplementary file 1 [file 41467_2026_75356_MOESM1_ESM.pdf]
