# Supplementary material for: A NIR fluorotag reporter CETIF6a enables bright pan-tumor labeling and functional proteomic profiling
Source: Nat Commun. 2026 Jul 8;17:8451. doi: 10.1038/s41467-026-75356-3 (PMC13478164; doi:10.1038/s41467-026-75356-3)
Supplement: Supplementary file 2 [file 41467_2026_75356_MOESM2_ESM.pdf]

nature portfolio

Corresponding author(s): Shoujun Zhu

Last updated by author(s): Jun 10, 2026

Reporting Summary

Nature Portfolio wishes to improve the reproducibility of the work that we publish. This form provides structure for consistency and transparency in reporting. For further information on Nature Portfolio policies, see our Editorial Policies and the Editorial Policy Checklist.

Statistics

For all statistical analyses, confirm that the following items are present in the figure legend, table legend, main text, or Methods section.

n/a	Confirmed	
☐	☒	The exact sample size (*n*) for each experimental group/condition, given as a discrete number and unit of measurement
☐	☒	A statement on whether measurements were taken from distinct samples or whether the same sample was measured repeatedly
☐	☒	The statistical test(s) used AND whether they are one- or two-sided<br>*Only common tests should be described solely by name; describe more complex techniques in the Methods section.*
☒	☐	A description of all covariates tested
☐	☒	A description of any assumptions or corrections, such as tests of normality and adjustment for multiple comparisons
☐	☒	A full description of the statistical parameters including central tendency (e.g. means) or other basic estimates (e.g. regression coefficient) AND variation (e.g. standard deviation) or associated estimates of uncertainty (e.g. confidence intervals)
☐	☒	For null hypothesis testing, the test statistic (e.g. *F*, *t*, *r*) with confidence intervals, effect sizes, degrees of freedom and *P* value noted<br>*Give P values as exact values whenever suitable.*
☒	☐	For Bayesian analysis, information on the choice of priors and Markov chain Monte Carlo settings
☒	☐	For hierarchical and complex designs, identification of the appropriate level for tests and full reporting of outcomes
☐	☒	Estimates of effect sizes (e.g. Cohen's *d*, Pearson's *r*), indicating how they were calculated

Our web collection on statistics for biologists contains articles on many of the points above.

Software and code

Policy information about availability of computer code

Data collection	MARS-IRIS 400-1700 nm absorbance/fluorescence spectrophotometer (Artemis); Sapphire FL Biomolecular Imager (Azure biosystems); UPLXAPO20X NA0.8 objective (Olympus); TripleTOF 5600 system (SCIEX); Nanospray III source (SCIEX); Vanquish Neo nL UHPLC system (Thermo Fisher Scientific); Orbitrap Fusion Lumos Tribrid mass spectrometer (Thermo Fisher Scientific).
Data analysis	PRISM 8 graphing software (Graphpad); ImageJ 1.54p (Wanye Rasband); Microsoft Excel 16.46 (Microsoft); Matlab R2016a (MathWorks); OlyVIA 4.1 (Olympus); BioPharma Finder 5.1 (Thermo Fisher Scientific); MaxQuant (version 2.1.3.0); Schrödinger 2021-2 (Schrödinger); The PyMOL Molecular Graphics System 2.5.7 (Schrödinger).

For manuscripts utilizing custom algorithms or software that are central to the research but not yet described in published literature, software must be made available to editors and reviewers. We strongly encourage code deposition in a community repository (e.g. GitHub). See the Nature Portfolio guidelines for submitting code & software for further information.

Data

Policy information about availability of data

All manuscripts must include a data availability statement. This statement should provide the following information, where applicable:

- Accession codes, unique identifiers, or web links for publicly available datasets
- A description of any restrictions on data availability
- For clinical datasets or third party data, please ensure that the statement adheres to our policy

The mass spectrometry proteomics data generated in this study have been deposited to the ProteomeXchange Consortium via the iProX partner repository with the

dataset identifier PXD069296. The data can be accessed via ProteomeXchange at: https://proteomecentral.proteomexchange.org/cgi/GetDataset?ID=PXD069296. The data are also available through the iProX repository at: https://www.iprox.cn/page/project.html?id=IPX0013731000. The source data generated in this study are provided in the Supplementary Information and Source Data file. Source Data file is provided with this paper.

Research involving human participants, their data, or biological material

Policy information about studies with human participants or human data. See also policy information about sex, gender (identity/presentation), and sexual orientation and race, ethnicity and racism.

Reporting on sex and gender	Sex/gender was not considered because the study focused on pan-tumor labeling across cancer types, not sex differences. The cohort included both female-only cancers (cervical, breast) and sex-balanced cancers. All samples came from clinical resections without sex preselection.
Reporting on race, ethnicity, or other socially relevant groupings	We confirm that this study does not include any analysis or reporting based on race, ethnicity, or other socially relevant groupings. These variables were not considered in the study design or data interpretation.
Population characteristics	For the use of clinical resected tumors and adjacent tissue samples for tissue section staining and proteomic identification, we collected tissue samples from 13 breast cancer, 8 cholangiocarcinoma, 20 cervical cancer, 4 thyroid cancer, 4 liver cancer, 3 rectal cancer, 3 colon cancer and 3 gastric cancer patients. Patient demographic information (age, sex) and detailed clinical and pathological characteristics are provided in Supplementary Table 1-2.
Recruitment	Human specimens were obtained from the Department of Biobank, Division of Clinical Research, the First Hospital of Jilin University.
Ethics oversight	All experiments involving human specimens had passed the review of the Ethics Committee of the First Hospital of Jilin University (2023-284-1).

Note that full information on the approval of the study protocol must also be provided in the manuscript.

Field-specific reporting

Please select the one below that is the best fit for your research. If you are not sure, read the appropriate sections before making your selection.

[x] Life sciences [ ] Behavioural & social sciences [ ] Ecological, evolutionary & environmental sciences

For a reference copy of the document with all sections, see nature.com/documents/nr-reporting-summary-flat.pdf

Life sciences study design

All studies must disclose on these points even when the disclosure is negative.

Sample size	In intraoperative histopathological examination, we performed 8 breast cases, 3 cholangiocarcinoma, 5 cervical cancer, 3 thyroid cancer, 4 liver cancer, 3 rectal cancer, 3 colon cancer and 3 gastric cancer patients. In the assessment of proteomic identification, we collected tissue samples from 5 breast cancer, 5 cholangiocarcinoma, 5 cervical cancer, 3 thyroid cancer and 1 liver cancer patients. In the assessment of immunohistochemical staining, we collected tissue samples from 15 cervical cancer.
Data exclusions	No data was excluded.
Replication	We performed three independent experiments and data are shown as the mean±SEM. All experiments were performed a minimum of three replicates in independent experiments with similar results.
Randomization	All human samples were randomly selected for all experiments.
Blinding	The investigators were blinded to group allocation during data collection and analysis.

Reporting for specific materials, systems and methods

We require information from authors about some types of materials, experimental systems and methods used in many studies. Here, indicate whether each material, system or method listed is relevant to your study. If you are not sure if a list item applies to your research, read the appropriate section before selecting a response.

Materials & experimental systems

n/a	Involved in the study
☐	☒ Antibodies
☐	☒ Eukaryotic cell lines
☒	☐ Palaeontology and archaeology
☐	☒ Animals and other organisms
☒	☐ Clinical data
☒	☐ Dual use research of concern
☒	☐ Plants

Methods

n/a	Involved in the study
☒	☐ ChIP-seq
☒	☐ Flow cytometry
☒	☐ MRI-based neuroimaging

Antibodies

Antibodies used	Antibodies include recombinant rabbit monoclonal anti-PCNA (Abcam, ab92552, 1:250 dilution), rabbit monoclonal anti-DNAJC9 (Abcam, ab166612, 1:100 dilution).
Validation	Specificity and detailed information of the antibodies used in this work were stated on the following websites: https://www.abcam.com/en-us/products/primary-antibodies/pcna-antibody-epr3821-ab92552; https://www.abcam.com/en-us/products/primary-antibodies/dnajc9-antibody-epr9856-ab166612.

Eukaryotic cell lines

Policy information about cell lines and Sex and Gender in Research

Cell line source(s)	The 4T1 cell lines were purchased from the Shanghai Enzyme Research Biotechnology Co., LTD.
Authentication	The cell lines have not been independently authenticated by our laboratory.
Mycoplasma contamination	The cell lines were negative for mycoplasma.
Commonly misidentified lines (See ICLAC register)	No misidentified cell lines were used.

Animals and other research organisms

Policy information about studies involving animals; ARRIVE guidelines recommended for reporting animal research, and Sex and Gender in Research

Laboratory animals	BALB/c mice were purchased from Liaoning Changsheng Biotechnology Co., Ltd. Bedding, nesting material, food, and water were provided ad libitum. The ambient temperature was maintained at 20–22 °C with a 12-hour light/12-hour dark cycle.
Wild animals	No wild animals were used in this study.
Reporting on sex	All mice were female gender.
Field-collected samples	The study did not involve samples collected from the field.
Ethics oversight	All animal experiments in this work were handled under the protocol approved by the Institutional Animal Care and Use Committee of The First Hospital of Jilin University (Protocol number: 20210642).

Note that full information on the approval of the study protocol must also be provided in the manuscript.

Plants

Seed stocks	*Report on the source of all seed stocks or other plant material used. If applicable, state the seed stock centre and catalogue number. If plant specimens were collected from the field, describe the collection location, date and sampling procedures.*
Novel plant genotypes	*Describe the methods by which all novel plant genotypes were produced. This includes those generated by transgenic approaches, gene editing, chemical/radiation-based mutagenesis and hybridization. For transgenic lines, describe the transformation method, the number of independent lines analyzed and the generation upon which experiments were performed. For gene-edited lines, describe the editor used, the endogenous sequence targeted for editing, the targeting guide RNA sequence (if applicable) and how the editor was applied.*
Authentication	*Describe any authentication procedures for each seed stock used or novel genotype generated. Describe any experiments used to assess the effect of a mutation and, where applicable, how potential secondary effects (e.g. second site T-DNA insertions, mosiacism, off-target gene editing) were examined.*
