# Supplementary material for: A NIR fluorotag reporter CETIF6a enables bright pan-tumor labeling and functional proteomic profiling
Source: Nat Commun. 2026 Jul 8;17:8451. doi: 10.1038/s41467-026-75356-3 (PMC13478164; doi:10.1038/s41467-026-75356-3)
Supplement: Supplementary file 3 [file 41467_2026_75356_MOESM3_ESM.pdf]

nature portfolio

Peer Review File

An NIR fluorotag reporter CETIF6a enables bright pan-tumor labeling and functional proteomic profiling

Corresponding Author: Professor Shoujun Zhu

This file contains all reviewer reports in order by version, followed by all author rebuttals in order by version.

Attachments originally included by the reviewers as part of their assessment can be found at the end of this file.

Version 0:

Reviewer comments:

Reviewer #1

(Remarks to the Author)
See attached

Reviewer #2

(Remarks to the Author)
The authors describe derivatization of the known cancer-selective dye IR-6B3 with an alkyne handle to enable target identification. Additional version of the modifications were developed to constitute a small library, based on some initial screening alkyne handle with 5 carbon was carried forward. Several cancerous tissue types were used to evaluate the fluorescens and protein targets.
However, this modification does not constitute a systematic structure–activity relationship, and therefore the claim that the study establishes a rational design paradigm for tumor-seeking dyes is not fully supported. The work reports modification of IR-6B3 by introducing an acetylene group at the terminus of the N-propyl substituent, which largely retains the optical properties of the parent dye. This represents a single-axis perturbation introduced primarily to enable click chemistry rather than to tune tumor-seeking behavior. The results suggest that the probes predominantly react with protein thiols, and pathway analysis highlights processes commonly associated with cancer cells. The manuscript presents a large number of figures and analyses, some of which may be more appropriate for inclusion in the Supporting Information. I draw attention to the following points:
1.The probe library and intermediates are not fully characterized to meet community standards. Preparation of intermediates is not referenced to reported procedures nor cross-checked against existing characterization data. In addition, the final probes are insufficiently characterized. At a minimum, high-quality 1H and 13C NMR spectra together with low or high resolution mass spectrometry (LRMS/HRMS) data should be provided. The Supporting Information currently contains only ^1H NMR spectra, which is insufficient.
2. The authors highlight pathways enriched by the alkyne probes; however, it would strengthen the manuscript to discuss how these pathways relate to established pan-cancer phenotypes or hallmarks of cancer, rather than presenting pathway enrichment primarily in a descriptive manner.
3. Overall, the study reports molecular targets associated with IR-6B3 using newly developed alkyne-enabled probes. While these probes retain near-infrared imaging capability and facilitate target identification, the limited size of the probe library and lack of systematic SAR analysis make it difficult to support broad claims of rational dye design.
4. The trypsin digestion and subsequent gel-based fluorescence experiments are used as evidence for the importance of the protein microenvironment. However, following trypsin digestion, the largest cysteine-containing peptides expected from the reported targets would likely be below ~5 kDa, making SDS–PAGE an inappropriate method to assess fluorescence persistence. Bulk solution-phase fluorescence measurements before and after trypsin treatment would provide a more accurate assessment of the role of the microenvironment. This also raises the question of how much of the observed fluorescence arises from probe reaction with cellular metabolites such as glutathione, which are generally elevated in cancer cells. This potential contribution is not directly addressed.

Version 1:

Reviewer comments:

Reviewer #1

(Remarks to the Author)
The revised manuscript entitled “A NIR fluorotag reporter CETIF6a enables bright pan-tumor labeling and functional proteomic profiling” presents a click-compatible heptamethine cyanine fluorotag that integrates tumor-targeting imaging with activity-based protein profiling. This work addresses a long-standing challenge in the field by systematically elucidating the molecular basis of tumor-seeking cyanine dyes and expanding their functionality toward proteome-wide target identification.

In the revised version, the authors have carefully considered the reviewer’s comments and have performed substantial additional experiments, including proteomic analyses, mechanistic validation, toxicity evaluation, and in vivo tumor-targeting assessment. These additions significantly strengthen the manuscript in terms of rigor, clarity, and completeness. The responses are clear and convincing, and the newly added data adequately support the conclusions. Overall, the study demonstrates strong scientific significance, clear innovation, and solid experimental support for the conclusions.

In view of the substantial improvements made by the authors and the satisfactory resolution of the previous concerns, I believe that the revised manuscript is suitable for publication in its current form.

I recommend acceptance of this revised manuscript.

Reviewer #2

(Remarks to the Author)
The authors have addressed my concerns by performing additional experiments to provide characterization data, testing fluorescence from key competing metabolites, clarifying the SAR, and revising the language to more accurately describe the findings.

Open Access This Peer Review File is licensed under a Creative Commons Attribution 4.0 International License, which permits use, sharing, adaptation, distribution and reproduction in any medium or format, as long as you give appropriate credit to the original author(s) and the source, provide a link to the Creative Commons license, and indicate if changes were made.
In cases where reviewers are anonymous, credit should be given to 'Anonymous Referee' and the source.
The images or other third party material in this Peer Review File are included in the article’s Creative Commons license, unless indicated otherwise in a credit line to the material. If material is not included in the article’s Creative Commons license and your intended use is not permitted by statutory regulation or exceeds the permitted use, you will need to obtain permission directly from the copyright holder.
To view a copy of this license, visit https://creativecommons.org/licenses/by/4.0/

We sincerely thank the editor and all reviewers for the time and effort on our manuscript entitled "A NIR fluorotag reporter CETIF6a enables bright pan-tumor labeling and functional proteomic profiling" (NCOMMS-25-76273). We also thank you for offering a resubmission opportunity to re-assess our manuscript. We have carefully gone through these important points, conducted all suggested experiments, and incorporated corresponding results into the manuscript. Our changes made throughout the main text were shown in red. Our point-to-point responses are shown in blue. In addition, we would like to seek your permission to change coauthor Feiran Zhang to a co-corresponding author, so as to appropriately recognize her contribution during the revision process.

Response to Reviewers' comments

Reviewer #1 (Remarks to the Author):

Comments: The submitted manuscript "A NIR fluorotag reporter CETIF6a enables bright pan-tumor labeling and functional proteomic profiling" that reported a click chemistry-compatible heptamethine cyanine fluorotag, CETIF6a, enables pan-cancer targeting, intraoperative histopathology, and proteome-wide target identification. The dual-functional reporter integrating tumor-seeking and activity-based protein profiling identifies hundreds of tumor-enriched targets across multiple cancer types and mechanism analysis, demonstrating that CETIF6a retains intraoperative tumor diagnosis and functional targets profiling. This study fills the gap in the application of cyanine dyes in tumor seeking, which is vitally significant for intraoperative diagnostics, and potentially theranostics. However, several critical issues have to be addressed before publication:

1. Although the authors cited several fluorescent dyes for tumor-seeking, there are missing some recent work, particularly for those imaging-guided detection probes (Angew. Chem. Int. Ed., 2022, 61, e202116807; J. Control. Release, 2024, 376, 961-971.), which should be included.

Response:

We thank the reviewer for the valuable suggestion to include more recent and relevant literature on imaging-guided detection probes.

The recommended articles describe highly sensitive prodrug systems, such as Bio-(8)-MB-CPT (Angew. Chem. Int. Ed., 2022, 61, e202116807) and the FDROS series (J. Control. Release, 2024, 376, 961–971) for cancer diagnosis and therapy. In these studies, Bio-(8)-MB-CPT targets overexpressed biotin receptors on cancer cells for tumor-seeking, followed by reactive oxygen species (ROS) triggered drug release in the tumor microenvironment, which is trackable via the simultaneous release of near-infrared (NIR) emitting methylene blue (MB). The FDROS series incorporates galactose as a targeting moiety for hepatocellular carcinoma, allowing rapid ROS-responsive drug release and NIR emission. We fully agree that citing these important works strengthens the background of our study.

In accordance with the reviewer's recommendation, we have now carefully cited the suggested articles in the revised manuscript. Specifically, the work from Angew. Chem. Int. Ed., 2022, 61, e202116807 has been cited as reference 6, and the study from J. Control. Release, 2024, 376, 961–971 has been cited as reference 7 in the Introduction section to support the discussion on the design of advanced fluorescent probes for tumor imaging.

2. As the author described, CETIF6a exhibited suboptimal pan-cancer labeling efficacy in HCC due to the increased density and a greater diversity and abundance of proteins in the pathological tissues. We would like to determine if any potential non-specific or off-target binding in liver tissue, considering that the heptamethine cyanine dye is readily captured by liver tissue.

Response:

We thank the reviewer for the valuable comments regarding the suboptimal fluorescence labeling efficacy of CETIF6a in distinguishing between hepatocellular carcinoma (HCC) and adjacent paracancerous tissues. In response to the reviewer's suggestions, we have performed further analyses and conducted additional experiments to investigate this phenomenon.

First, we extended our analysis of the 2 cases (G0268, G1952) of hepatocellular carcinoma (HCC) that were difficult to distinguish as described in the manuscript. We presented the three-dimensional histological electrophoresis results for each specimen (**Fig. R1a**) and demonstrated that the difficulty in distinguishing tumors (T) from paracancerous (P) tissues arised from the high fluorescence observed in both, which lead to a reduced T/P ratio (**Fig. R1b**). This finding suggests that both tumor and adjacent liver tissues contain abundant target proteins that can covalently bind to CETIF6a, thereby significantly compromising the HCC specificity of the probe. This observation aligns with your suggestion that non-specific binding in liver tissue may lead to excessive background signal interference.

To further investigate the targets responsible for the high fluorescence intensity in P tissues, we performed target protein enrichment and analysis on HCC tissues and their corresponding adjacent normal tissues, followed by quantitative analysis of the enriched target proteins. Our results indicated that CETIF6a could label a broader range of proteins in HCC tissues rather than paracancerous tissues (**Fig. R1c**). However, the top 50 most abundant target proteins were present in both T and P tissues, with no significant differences in their abundance (**Fig. R1d**). This likely explains the strong fluorescence observed in P tissues, which can be attributed to the high expression of numerous non-specific targets in paracancerous tissues. We further listed the top 5 targets in **Fig. R1d**, which include HSPD1 (a heat shock protein involved in mitochondrial protein import and macromolecular assembly), CPS1 (carbamoyl-phosphate synthase, a key enzyme in the urea cycle responsible for ammonia removal), KRT1 (a cytoskeletal keratin), ATP5F1A (the alpha subunit of the mitochondrial ATP synthase complex),

and ALB (albumin, which regulates blood colloid osmotic pressure and participates in the transport of zinc, calcium, and magnesium) and believed that these targets are representative non-specific targets in HCC. The abundant presence of these non-specific targets in liver tissues contributes to the suboptimal differentiation.

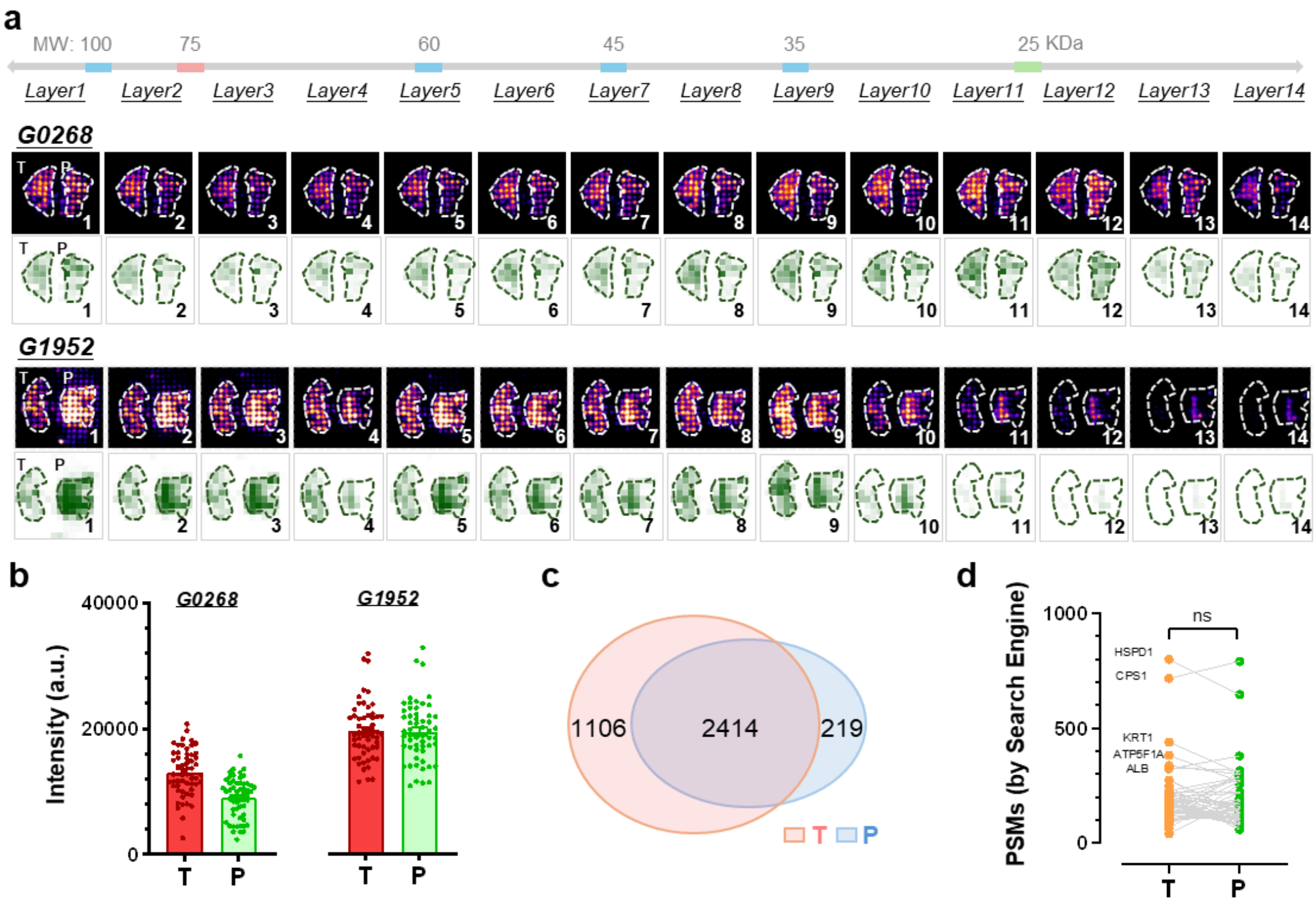

Figure R1. Three-dimensional histological electrophoresis imaging and target protein group analysis of HCC. (a) The three-dimensional histological electrophoresis imaging of 2 cases of indistinguishable HCC. **(b)** Statistical graph of T and P tissue fluorescence intensity for the HCC cases. **(c)** Venn diagram illustrating the overlap of target proteins identified in the T group versus the P group across the HCC samples. **(d)** The content of the 50 most abundant target proteins in the target protein groups of T and P tissues.

Based on these findings, we have revised the description in the manuscript regarding the reduced efficacy in HCC. The revised content of the manuscript is as follow "Furthermore, we speculate that normal liver tissue inherently exhibits a greater diversity and abundance of target proteins, which may lead to increased target availability in P tissues and consequently higher background signal".

3. The azide–alkyne cycloaddition reaction requires Cu(I) catalysis, however, copper ions pose significant physiological toxicity. Please check that probe concentrations used for labeling are within clinically safe ranges, and have any relevant toxicity assessments been performed?

We appreciate the reviewer's thoughtful comments. In this study, the CETIF series of probes were used exclusively for in vitro labeling experiments, where the pathological tissue removed

during the clinical procedure were sliced and stained for imaging. Therefore, the safety concerns regarding the in vivo use of probes or copper ions are beyond the scope of this study. The probe concentrations used in this study were optimized based on labeling efficacy. The copper ion concentration used is the standard concentration for ABPP experiments, based on methodological articles[1,2]. Thus, the concentrations of the probes and catalyst used in this paper are both reasonable and yield optimal labeling performance.

Given that CETIF6a is a NIR emitting probe, we also mentioned in the Discussion section that CETIF6a has the potential to be compatible with intraoperative navigation platforms. Based on the reviewer's constructive suggestion, we further performed an in vivo toxicity evaluation of CETIF6a. Referring to the clinically safe concentration of indocyanine green (ICG), we tested whether CETIF6a causes toxicity in mice. The probe was administered via tail vein injection (female BALB/c mice, probe concentration 0.5 mg/kg). CETIF6a was first dissolved in DMSO as a 2 mM stock solution, then diluted with PBS to 0.05 mg/mL. Each mouse received 200 µL of the diluted solution, and an equal volume of PBS was injected as control. After 24 hours of the injection, the heart, liver, spleen, lungs, and kidneys of mice were collected for histopathological analysis. The results showed in **Fig. R2**. Compared with the control group, the organ structures in the experimental group were normal, indicating that CETIF6a does not cause toxic damage to mice at a safe concentration.

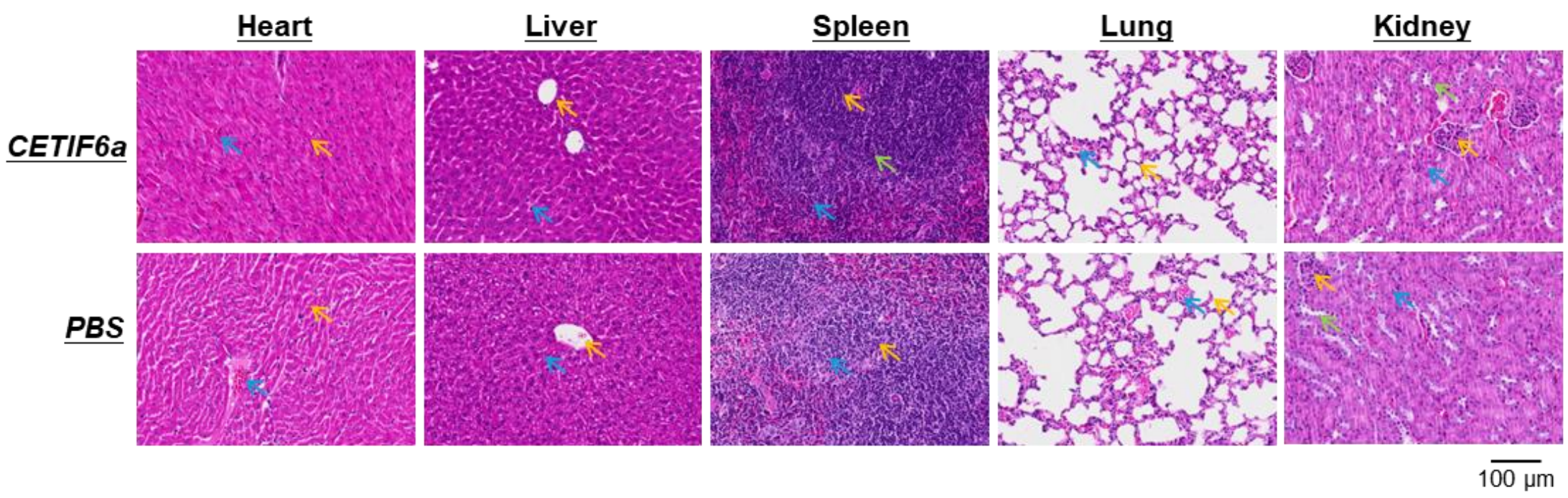

Figure R2. Toxicity assessments of CETIF6a. Heart: The myocardial tissue structure is intact. The myocardial fibers (indicated by the yellow arrows) are neatly and densely arranged, distributed in bundles, with clear striations visible. A small amount of connective tissue and capillaries (blue arrows) can be seen between the muscle fibers. The vascular lumen is unobstructed, and no obvious pathological changes are observed. **Liver**: The liver tissue structure is clear, the hepatic lobule structure is intact, and the hepatic cell cords are radially arranged around the central vein (yellow arrow); the hepatic sinus structure is clear and unobstructed, and the portal area structure (blue arrow) is normal, with no obvious abnormal changes. **Spleen**: The structure of the spleen is intact, with a clear boundary between the white pulp (yellow arrow) and the red pulp (green arrow); the structure of the splenic trabeculae is also intact (blue arrow), and no obvious abnormalities such as bleeding, necrosis, or inflammatory cell infiltration are observed. **Lung**: The lung tissue structure is clear, the alveoli are evenly distributed, the size of the alveolar cavities is basically the same, and the alveolar septa (yellow arrows)

are thin and intact; a small amount of capillaries (blue arrows) can be seen in the interstitium, and the structure is normal; the epithelium of the bronchi and bronchioles is arranged neatly; there is no obvious inflammatory cell infiltration or exudate in the alveolar cavities, and no obvious edema, thickening or fibrosis changes are observed in the lung interstitium. **Kidney**: The renal tissue structure is clear, and the morphology of the glomeruli (yellow arrow) is intact; the renal tubules are arranged neatly, the epithelial cells of the proximal tubules (blue arrow) have relatively strong eosinophilic cytoplasm, and the distal tubules (green arrow) have a lighter staining; there are few abnormal changes in the renal interstitium, the vascular structure is normal, and no obvious inflammatory cell infiltration, necrosis or fibrosis is observed.

4. How efficient is the reaction within living tissues, and is the applicability to larger solid tumor constrained by penetration depth?

We thank the reviewer for the valuable suggestion regarding the efficiency of the in vivo azide-alkyne cycloaddition reaction. In this study, all cycloaddition experiments were performed using materials derived from in vitro tissue samples. Therefore, we only evaluated the efficiency of the CETIF6a-mediated cycloaddition reaction in vitro, and the reaction efficiency within living tissues was not a consideration for this study. Furthermore, we fully agree with your point raised in the previous comment regarding the physiological toxicity of copper ions, and thus we would not conduct copper-catalyzed cycloaddition reactions in living tissues. We acknowledge that existing studies have indeed performed click chemistry reactions in vivo, primarily using copper-free clickable functional groups such as DBCO[3]. These groups offer milder reaction conditions and improved biosafety. Consequently, for applications involving in vivo tumor imaging or enrichment of tumor target proteins, such copper-free click handles should be considered over the copper-catalyzed approach used in this study.

Although CETIF6a cannot be directly applied to living tissues for click chemistry labeling, your comment prompted us to further investigate whether CETIF6a exhibits tumor-targeting properties in vivo and whether such targeting is limited by probe penetration depth. To address this, we established mouse models bearing tumors of different sizes (using 4T1 cells to generate a murine breast cancer model). After intravenous injection of CETIF6a, we continuously monitored the mice for 24 hours. The results in **Fig. R3a** showed that the probe exhibited tumor-targeting properties, accumulating at the tumor site and reaching peak fluorescence intensity and signal-to-noise ratio at 8 hours after the injection (**Fig. R3b-c**). Following fluorescence imaging, we extracted total protein from tumor (T) and muscle (M) tissues and performed polyacrylamide gel electrophoresis. The probe-labeled protein bands were visualized using a fluorescence imaging system, and the results are shown in **Fig. R3d-e**. The findings indicated that the probe primarily binds to albumin, and this complex was significantly enriched in tumor tissues compared with healthy tissues (muscle). Specifically, CETIF6a covalently binds to abundant albumin in the blood, and due to the enhanced

permeability and retention (EPR) effect, the covalent complex accumulates in tumors through the circulation, leading to marked tumor-targeting capability. Due to the high activity and extremely high concentration of albumin compared to tumor proteins, when applied in vivo, almost all of the injected dose of the dye is bound to albumin. Therefore, the targeting is mediated by albumin, rather than by tumor proteins in the ex vivo tissue.

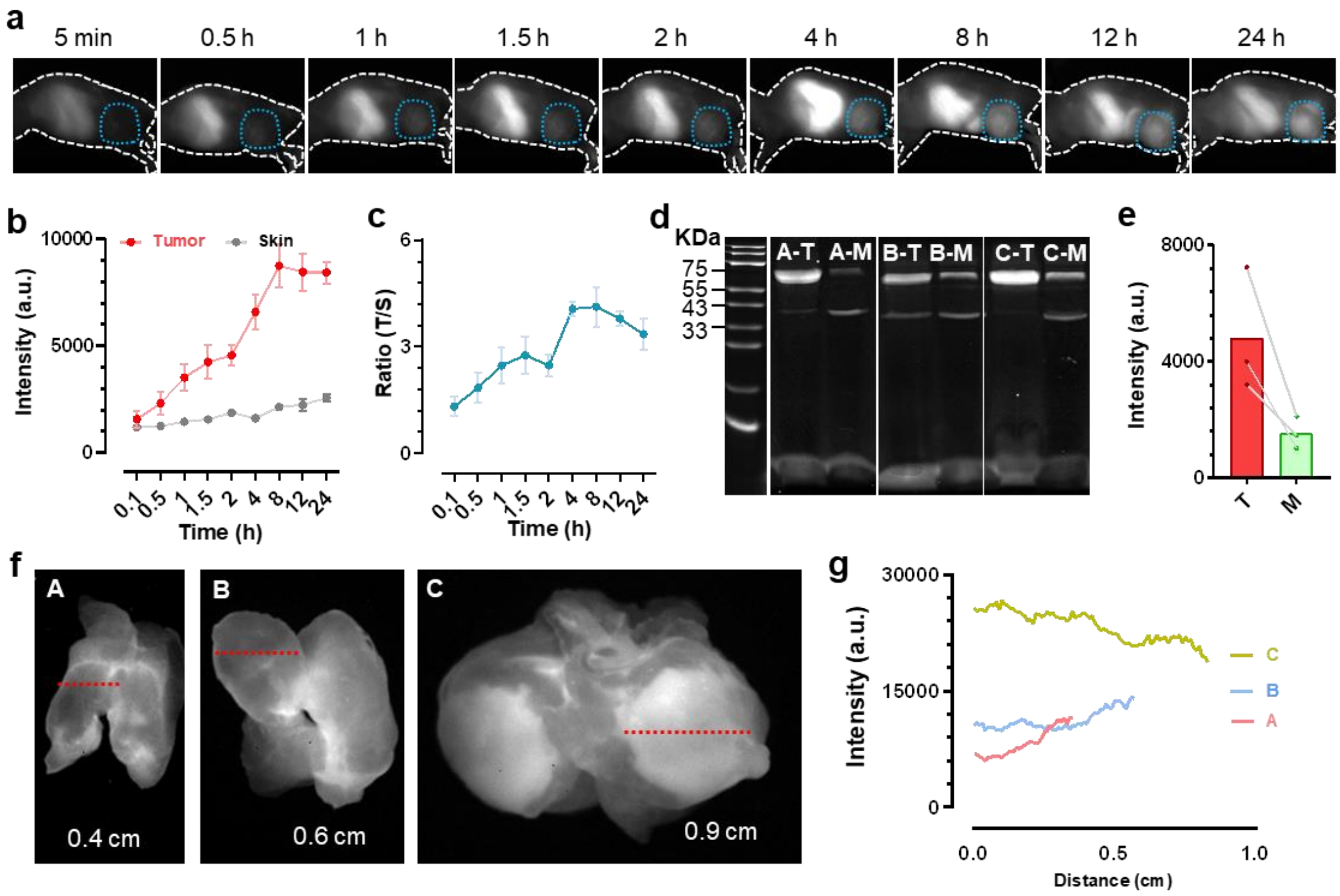

Figure R3. The tumor targeting property of CETIF6a in vivo. (a) The probe distribution within 24 hours after intravenous injection of CETIF6a into tumor-bearing mice. The outline of the mice is delineated by the gray dotted line, and the outline of the tumor is delineated by the blue dotted line (n=3). **(b)** Statistical graph of tumor and skin fluorescence intensity in 24 h. **(c)** Statistical graph of the ratio between tumor and skin fluorescence intensity in 24 h. **(d)** Fluorescence brightness of covalently bound complexes detected by electrophoresis in SDS-PAGE gel. **(e)** The statistical results of the brightness of the albumin band in figure d. **(f)** Fluorescence imaging of cross-sections of tumors of different sizes. **(g)** The fluorescence intensity corresponding to the red dotted line in figure f.

We then evaluated whether the ability of CETIF6a to label tumors in vivo is limited by penetration depth. After completing the 24 h in vivo imaging shown in **Fig. R3a**, we sectioned the tumors of varying sizes along their central axis and assessed the uniformity of fluorescence distribution across the section. As shown in **Fig. R3f-g**, no significant differences in fluorescence intensity were observed between the center and the periphery of tumors with different sizes. Therefore, we consider that CETIF6a possesses favorable penetration capacity even in larger solid tumors.

5. For the stage I of the binding, could you clarify whether all hydrophobic pockets can

participate in binding? Additionally, is there any binding or modification observed for amino acids incapable of forming thioether bond? Please clarify in detail.

We appreciate the reviewer's attention to the universality of the binding model. Based on the comments, we have performed additional experiments to address this issue.

First, regarding the question about the first stage of binding: "whether all hydrophobic pockets can participate in binding", we expressed and purified representative hydrophobic pockets using a eukaryotic expression system. Since the mass spectrometry results for cervical cancer and adjacent normal tissues revealed a large number of target proteins, it was not feasible to recombinantly express and test every hydrophobic pocket. Therefore, we selected 2 representative targets: LCP1, the most abundant target enriched in tumor tissues, and albumin, the most abundant target enriched in adjacent normal tissues (as shown in **Fig. 2d** of the manuscript). We expressed the hydrophobic pocket domain IV of LCP1 (**Fig. R4a**), as well as domains I, II, and III of albumin (**Fig. R4b**), and tested whether these 4 hydrophobic pockets participate in the first stage of the binding. The probe and each recombinantly expressed domain were mixed at a 1:1 molar ratio (10 μM), and the brightness of the resulting complexes was measured.

The results showed that CETIF6a selectively binds to domain III of albumin and binds very weakly to the other 3 domains (**Fig. R4c-d**). This indicates that not all hydrophobic pockets are capable of participating in the binding, and whether binding occurs likely depends on the compatibility between the hydrophobic pocket and CETIF6a, as well as the thiol activity on cysteine conferred by the pocket structure.

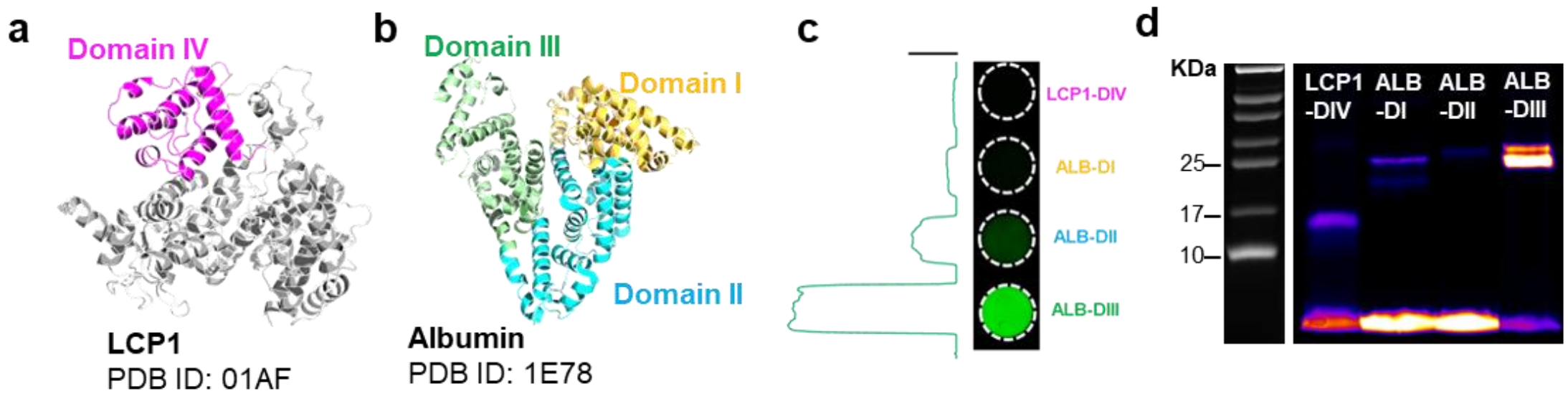

Figure R4. The binding state of CETIF6a to different hydrophobic pockets. (a) The protein structure of LCP1. **(b)** The protein structure of Albumin. **(c)** The fluorescence intensity after the probe was incubated with different hydrophobic pockets (intensity bar = 10000). **(d)** The fluorescence intensity of the band showing the covalent binding of the probe to the proteins.

In response to your question regarding the specificity of probe conjugation, we performed further experiments to determine the modification sites for our targets of interest in breast cancer and cholangiocarcinoma, analogous to the analysis shown in **Fig. 5f** of the manuscript. The proteomic results for 9 candidates selected from the targets of breast cancer and cholangiocarcinoma revealed that probe modification occurred exclusively on cysteine

residues (**Table R1**). No probe binding or modification was observed on amino acids that cannot form thioether bonds. We hope these additional data address your concerns.

Table R1. CETIF6a modification sites on 9 candidates.

No.	Protein Name	Site
1	A2M	C540, C572, C619, C540, C572, C619, C798, C824, C826, C898, C949
2	AKT1	C60, C77, C224, C310, C344, C460
3	B2M	C46, C101
4	BCAM	C22, C140, C205, C243
5	FUS	C428, C433
6	ACAA1	C26, C218
7	SERPINB5	C183, C205, C287, C323
8	ANXA13	C114
9	HSD17B2	C12, C99

6. The slide staining images of A1 in Figure S2k, are the slides for CETIF4a, 5a, and 6a from the same slide? They appear to be identical. Also, the scale bars need to be added in Figure 1d and S2k.

We appreciate the reviewer's careful review and valuable comments. Regarding **Fig. S2k**, the images shown for CETIF4a, 5a, and 6a were obtained from adjacent sections, rather than from the same slide. As a result, their tissue morphology appears similar but not identical. To further clarify this point, we have included below the original fluorescence scanning results of tissue sections from three patient samples imaged under the same field of view (**Fig. R5**).

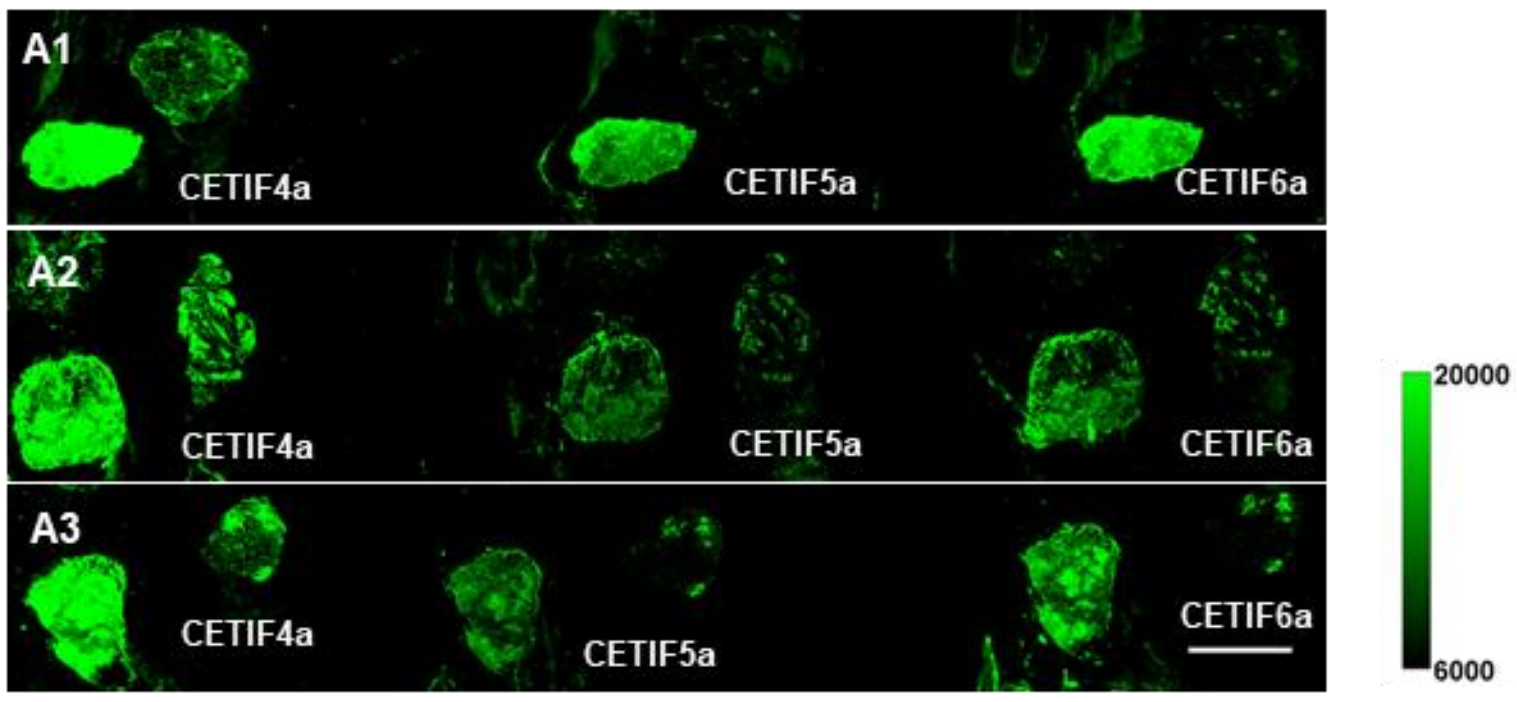

Figure R5. Imaging efficacy of CETIF4/5/6a mediated slide staining in delineating tumor regions of clinical cancer samples (Scale bar: 5 mm).

We have also added scale bars to **Fig. 1d** and **Fig. S2k** and have included the following sentence into the figure legends to address this issue: "the length of the scale bar: 5 mm". Thank you again for your thorough review and helpful suggestions.

Reviewer #2 (Remarks to the Author):

The authors describe derivatization of the known cancer-selective dye IR-6B3 with an alkyne handle to enable target identification. Additional version of the modifications were developed to constitute a small library, based on some initial screening alkyne handle with 5 carbon was carried forward. Several cancerous tissue types were used to evaluate the fluorescens and protein targets. However, this modification does not constitute a systematic structure–activity relationship, and therefore the claim that the study establishes a rational design paradigm for tumor-seeking dyes is not fully supported. The work reports modification of IR-6B3 by introducing an acetylene group at the terminus of the N-propyl substituent, which largely retains the optical properties of the parent dye. This represents a single-axis perturbation introduced primarily to enable click chemistry rather than to tune tumor-seeking behavior. The results suggest that the probes predominantly react with protein thiols, and pathway analysis highlights processes commonly associated with cancer cells. The manuscript presents a large number of figures and analyses, some of which may be more appropriate for inclusion in the Supporting Information. I draw attention to the following points:

We appreciate the reviewer for pointing out these important issues. We acknowledge your suggestion that the design of the CETIF probe may not sufficiently support a rational paradigm for tumor-targeted dye development. In response to this concern, we have further tested additional structural modifications beyond the original single-axis perturbation to evaluate their effects on tumor-seeking performance. We have expanded the probe library and conducted a systematic structure activity relationship (SAR) analysis. A detailed description of these additional experiments and the corresponding discussion addressing this key issue are provided in our response to the 3rd comment.

Regarding the pathway analysis, we have also supplemented the corresponding data and discussion in our response to the 2nd comment.

Finally, based on your valuable suggestions, we have reorganized the figures and analytical data in the manuscript. We agree that the molecular docking results are derived from computational modeling and simulation and serve as supporting evidence for exploring the binding mechanism. Therefore, we have moved the molecular docking data originally presented in Figure 3a to Supplementary Information to improve the layout of the main text.

1.The probe library and intermediates are not fully characterized to meet community standards. Preparation of intermediates is not referenced to reported procedures nor cross-checked against existing characterization data. In addition, the final probes are insufficiently characterized. At a minimum, high-quality 1H and 13C NMR spectra together with low or high resolution mass spectrometry (LRMS/HRMS) data should be provided. The Supporting Information currently contains only ^1H NMR spectra, which is insufficient.

We appreciate the reviewer for the valuable suggestion. Based on your comments, we have fully characterized the intermediate and final products described in this study. We now provide high-quality ^{1}H and ^{13}C NMR spectra for the intermediates, as well as high-quality ^{1}H and ^{13}C NMR spectra together with HRMS and high-performance liquid chromatography (HPLC) data for the final products. These relevant data have been presented on **pages 20-33** of the revised Supplementary Information.

2. The authors highlight pathways enriched by the alkyne probes; however, it would strengthen the manuscript to discuss how these pathways relate to established pan-cancer phenotypes or hallmarks of cancer, rather than presenting pathway enrichment primarily in a descriptive manner.

We thank the reviewer for the insightful suggestion to further connect the enriched pathways to established pan-cancer phenotypes and cancer hallmarks. We agree that moving beyond descriptive reporting of pathway enrichment can substantially strengthen the mechanistic implications of our findings.

In the revised manuscript, we performed an additional functional interpretation of the KEGG pathway enrichment results. Specifically, we mapped the 5 core pathways enriched in tumor tissues (Ribosome, Proteasome, Carbon metabolism, Protein processing in endoplasmic reticulum, and Citrate cycle) onto the well-established Hallmarks of Cancer framework (**Fig. R6**) [4,5].

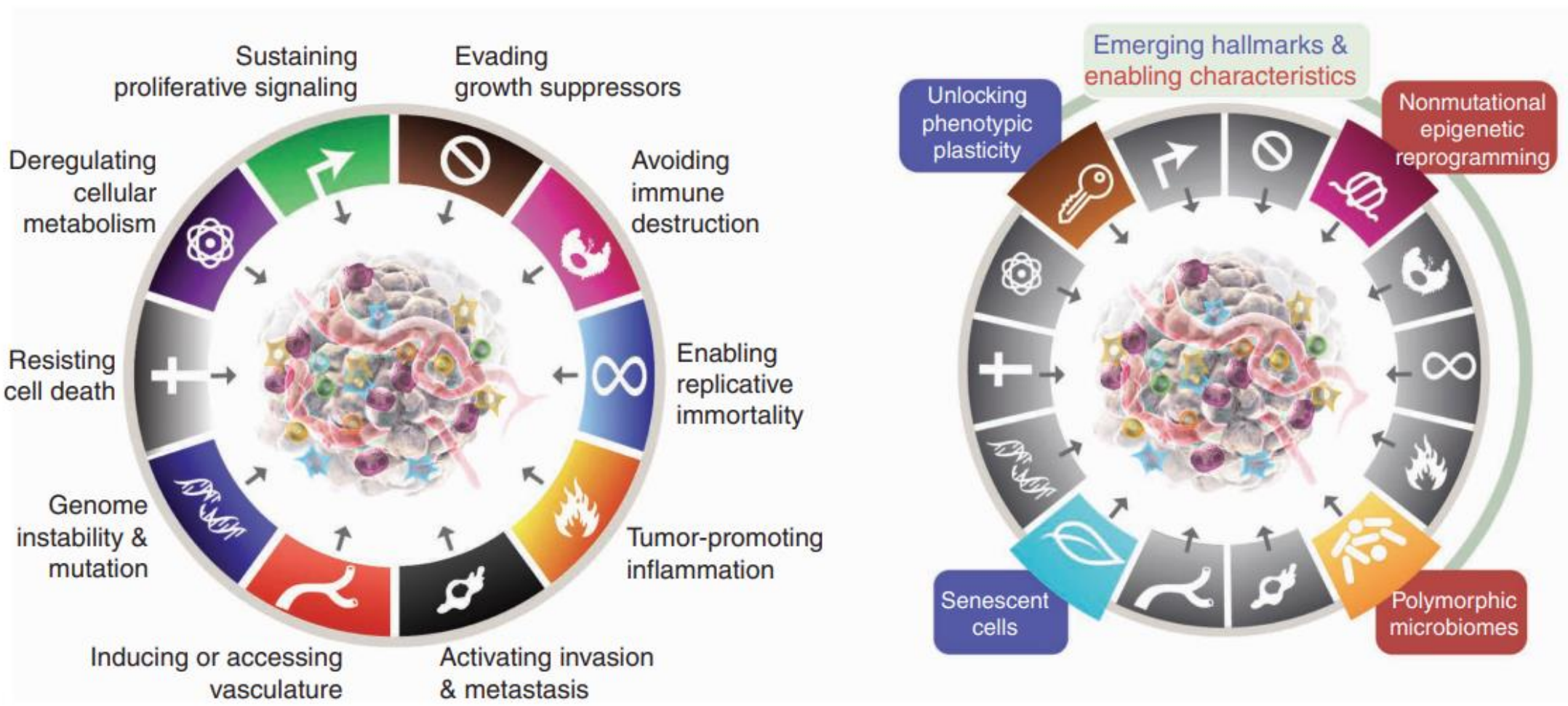

Figure R6. The hallmarks of cancer[4].

This analysis revealed that these pathways collectively support several hallmark capabilities of malignant cells, including sustained proliferative signaling, resisting cell death, metabolic reprogramming, and evading growth suppressors. Together, they depict a coordinated cellular state that underpins tumor cell survival and proliferation. This expanded interpretation has been incorporated into the Results sections to provide a more mechanistic link between the enriched targets and core cancer biology: "To further elucidate the biological relevance of

these enriched pathways in the context of cancer biology, we mapped them onto the Hallmarks of Cancer framework proposed by Hanahan and Weinberg. The Ribosome pathway supports **sustained proliferative signaling** by facilitating the elevated protein synthesis required for rapid cell division. The Proteasome pathway, which governs protein degradation and turnover, is critically linked to **genome instability and mutation**. The pathway enables cancer cells to tolerate the accumulation of aberrant or misfolded proteins resulting from oncogenic stress, thereby promoting survival. Carbon metabolism and the Citrate cycle are key components of **reprogrammed energy metabolism**, a hallmark that allows cancer cells to adapt their metabolic fluxes to meet the biosynthetic and energetic demands of proliferation. Lastly, protein processing in the endoplasmic reticulum contributes to **resistance to cell death** by alleviating ER stress and maintaining cellular survival under unfavorable conditions. Collectively, these pathways converge on a coordinated set of hallmark capabilities that are fundamental to malignant transformation and maintenance. Thus, the protein networks captured by CETIF6a not only reflect the molecular landscape of tumor tissues but also directly engage with core oncogenic processes, providing a mechanistic basis for the probe's robust pan-cancer labeling performance". We speculate that these tumor proteins labeled by CETIF6a play important roles in relevant pathways and possess relatively active structural characteristics, making them readily labeled by CETIF6a.

3. Overall, the study reports molecular targets associated with IR-6B3 using newly developed alkyne-enabled probes. While these probes retain near-infrared imaging capability and facilitate target identification, the limited size of the probe library and lack of systematic SAR analysis make it difficult to support broad claims of rational dye design.

We appreciate the reviewer for the thoughtful suggestions regarding probe structure design. In the manuscript, we focused on discussing how modifying the side chain of IR-6B3 with different clickable functional groups affects tumor targeting. In this revision, we performed systematic structural optimization and structure activity relationship (SAR) analysis on IR-6B3. First, we divided the molecular scaffold of IR-6B3 into three parts: 1) the heptamethine core, 2) the indole ring, and 3) the side chain alkyl group (**Fig. R7a**). Each part was sequentially modified and evaluated, and the resulting probes were named according to the modifications. By adjusting the heptamethine core, we examined the sensitivity of different cores for tumor-specific binding, generating probes IR-1B3, IR-2B3, IR-3B3, IR-4B3, IR-5B3, and IR-6B3. By modifying the indole ring, Benz[e]indole and Fischer indole were introduced as candidates to delocalize the frontier molecular orbitals and provide different steric hindrance[6]. Specifically, replacing the benzene ring of IR-6B3 with a naphthalene ring yielded IR-6N3. By varying the side chain length, we compared the impact of steric hindrance on targeting performance, testing probes with symmetric side chains containing 3 to 9 carbon atoms. We synthesized these probes to expand the probe library. The characterization data of the probe is presented

in the supporting data at the end of this document.

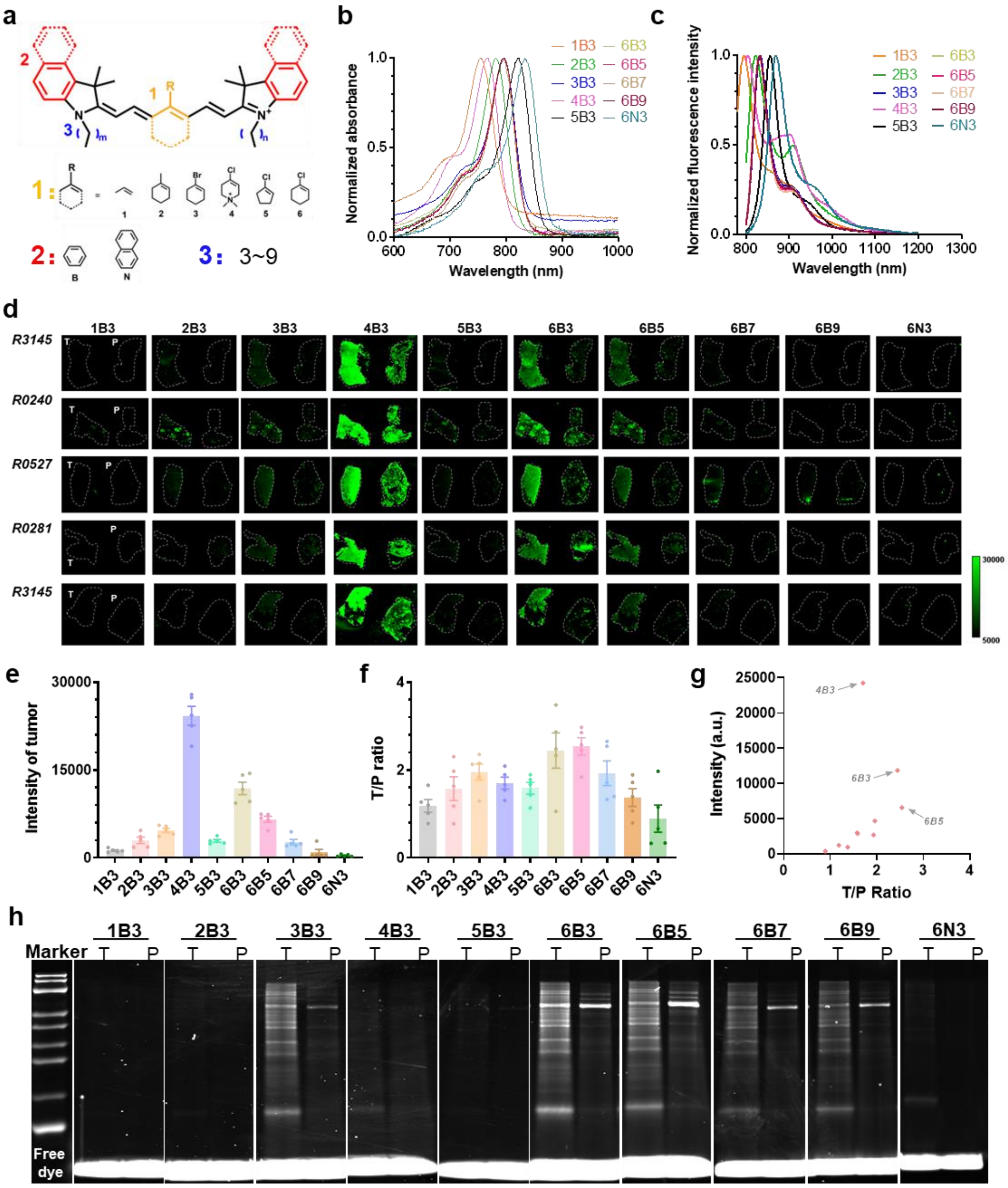

Figure R7. Expansion of the tumor-targeting probe library and structure-activity analysis. (a) The structure of the probes. **(b)** The absorption spectrum of the probes. **(c)** The emission spectrum of the probes. **(d)** Slide staining distinguishes tumor tissues from adjacent paracancerous tissues in 5 clinical samples. **(e)** Statistical data of mean fluorescence intensity of the tumor areas labeled by different dyes in panel d. **(f)** Statistical comparison of the fluorescence ratio between T and P regions labeled by different dyes in panel d. **(g)** The tumor-targeting performance of the probe. **(h)** Unidirectional electrophoresis evaluated the ability of different probes to covalently target proteins in 5 samples.

We first measured the spectral properties of these probes (**Fig. R7b-c**). Changing the side chain length had almost no effect on the spectra, whereas the other modifications caused

slight spectral shifts. Subsequently, we evaluated the staining performance of these probes on 5 clinical tumor tissue samples. Adjacent sections were stained using identical probe concentrations and procedures, and the staining results were imaged using the high-resolution imaging system. As shown in **Fig. R7d–g**, among all probes, IR-4B3, IR-6B3, and IR-6B5 yielded higher brightness in tumor tissues, with IR-6B3 and IR-6B5 exhibiting a T/P ratio >2, indicating favorable tumor targeting and imaging performance. These results suggest that probes with the structures of IR-6B3 and IR-6B5 possess a good capacity to distinguish tumor tissues.

We further analyzed the binding mode of these probes to target proteins (**Fig. R7h**). The results revealed that when the central rigid cyclohexene ring in the heptamethine conjugated chain bears a halogen atom, the probe undergoes covalent binding to proteins, indicating that the C-halogen on the cyclohexene serves as the active site for protein targeting. Notably, the brightness of covalent binding was higher for Cl than for Br. Replacing the benzene ring with a naphthalene ring (6N3) abolished protein binding, suggesting that the indole ring requires relatively low steric hindrance to achieve specific labeling of target proteins. In addition, longer side chains resulted in lower labeling brightness in tissues, further indicating that increased steric hindrance restricts probe-protein binding. This also demonstrates the rationality of our approach in the manuscript, where we added click functional groups based on the side chain lengths of 3 to 5 carbon atoms and conducted tests. Therefore, selecting a chlorine-substituted cyclohexene as the heptamethine core, together with the indole ring and an appropriate side chain length to minimize steric hindrance, while utilizing the C-Cl active site to achieve stable covalent binding with target proteins, collectively contributes to the tumor-targeting capability of the probe.

In the previously published work of our team, similar structure activity relationship studies were conducted, both the current results and those from previous work have identified IR-6B3 as a tumor-targeting probe. Hence, the present study focuses on introducing clickable functional groups into the side chain of IR-6B3 to investigate the targets and mechanisms underlying IR-6B3-mediated tumor targeting. We again thank the reviewer for your careful and thoughtful suggestions, which allowed us to thoroughly discuss the rationale for selecting IR-6B3 as the parent structure.

To be more rigorous and avoid ambiguity, we have replaced the inappropriate statement in the Discussion, "This study establishes a paradigm for rational design of tumor-seeking dyes" with the following: "This study demonstrated that the incorporation of clickable functional groups at the side chain provides a viable strategy for functionalizing tumor-seeking dyes without compromising their targeting capability."

4. The trypsin digestion and subsequent gel-based fluorescence experiments are used as evidence for the importance of the protein microenvironment. However, following trypsin

digestion, the largest cysteine-containing peptides expected from the reported targets would likely be below ~5 kDa, making SDS–PAGE an inappropriate method to assess fluorescence persistence. Bulk solution-phase fluorescence measurements before and after trypsin treatment would provide a more accurate assessment of the role of the microenvironment. This also raises the question of how much of the observed fluorescence arises from probe reaction with cellular metabolites such as glutathione, which are generally elevated in cancer cells. This potential contribution is not directly addressed.

We appreciate the reviewer for the valuable comments. We sincerely appreciate your identification of the inappropriate imaging method we used after trypsin treatment. To address this issue, we re-expressed the recombinant candidate target protein and re-extracted tissue proteins from clinical samples. After incubating the protein with the dye, we treated the complex with trypsin and directly detected the brightness of the complex, as shown in the revised **Fig. R8a-d**. The significant reduction in the brightness of the complex after trypsin treatment confirms that the integrity of the binding pocket is crucial for maintaining the probe's fluorescence. We also observed that the fluorescent brightness did not completely disappear after trypsin treatment. Therefore, we revised our original statement claiming a complete loss of brightness to ensure a more accurate description. Based on your valuable suggestions regarding the data collection approach for this section, and with the aim of conveying information concisely and accurately through the main figures, we have revised **Fig. 3** in the original manuscript. First, the molecular docking results originally shown in **Fig. 3a** have been moved to the supplementary information as **Supplemental Figure 8.** Subsequently, we replaced **Fig. 3b-e** with **Fig. R8a-d**. Accordingly, we have revised the corresponding text in the original manuscript, which now reads: "NIR fluorescence decreased upon destruction of the binding cavity, observed for all recombinant target proteins (Fig. 3a-b) and tissue lysates (Fig. 3c-d)."

To further clarify the proportion of observed fluorescence attributable to the reaction between the probe and cellular metabolites (e.g., glutathione), we tested the brightness of 10 μM probe CETIF6a after incubation with 10 μM reduced glutathione (GSH), 10 μM oxidized glutathione (GSSG), 10 μM tumor tissue protein, and 10 μM paracancerous tissue protein at room temperature for 2 h. The results are shown in the **Fig. R8e-f**. Although the brightness of CETIF6a increased approximately twofold after reaction with reduced GSH (brightness in PBS: 555.9 ± 30.82; brightness after co-incubation with GSH: 1338 ± 28), the brightness increased 104-fold after incubation with tissue proteins (brightness after co-incubation with tumor tissue: 58017 ± 93.29). These findings suggest that while the probe binds to GSH, a metabolite elevated in cancer cells, this binding does not lead to a significant enhancement of fluorescence under near-physiological conditions.

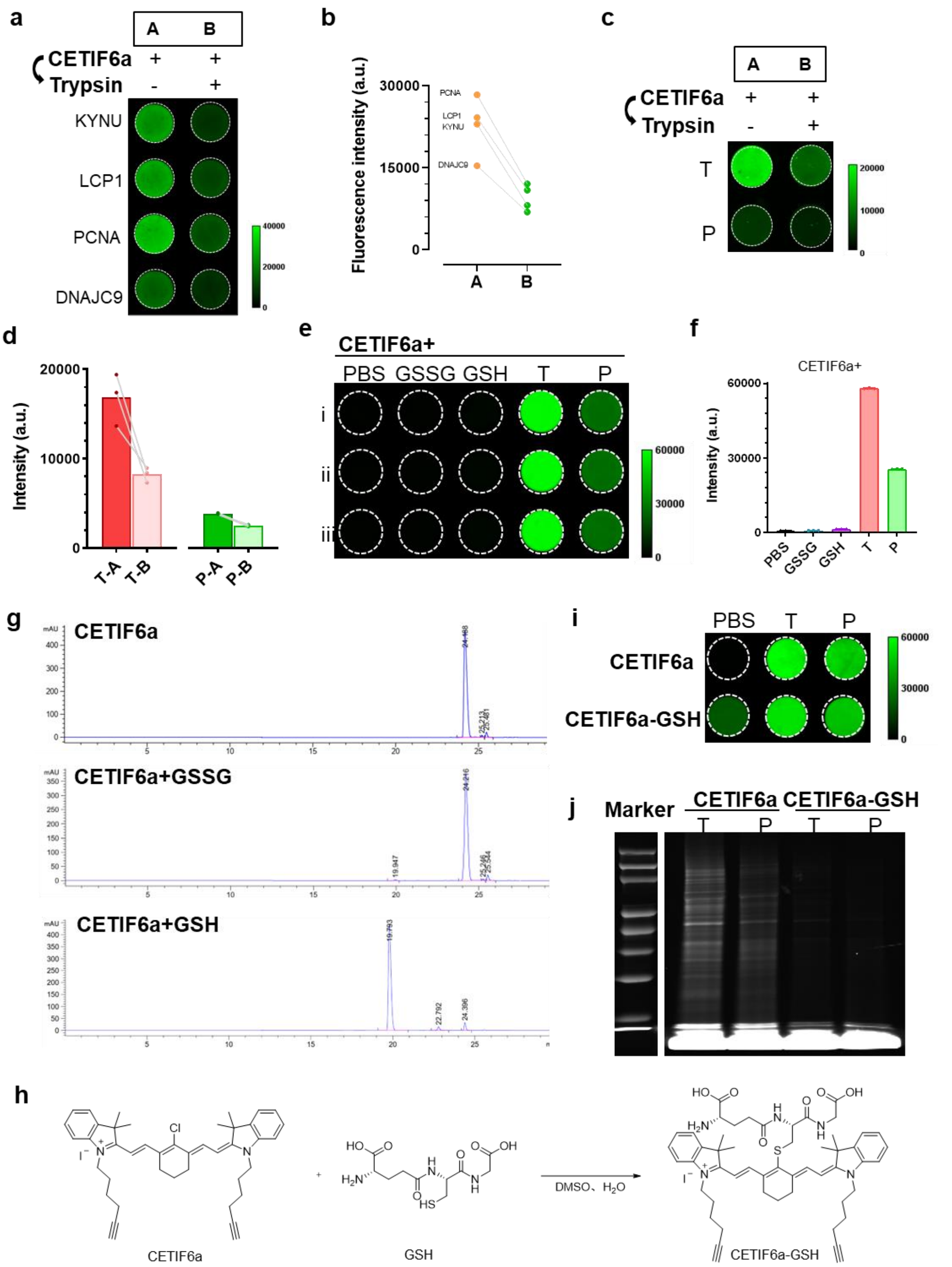

Figure R8. The influence of the protein microenvironment and cellular metabolites on brightness. (a) The brightness changes of the CETIF6a@targets after treatment with trypsin. **(b)** Fluorescence intensity analysis of the orifice plate in a. **(c)** The brightness changes of the CETIF6a-tissue proteins after treatment with trypsin. **(d)** Fluorescence intensity analysis of the orifice plate in c. **(e)** The fluorescence intensity after CETIF6a binds to glutathione. **(f)** Statistical comparison of the fluorescence in panel e. **(g)** HPLC analysis of the complex formed after incubation of CETIF6a with glutathione. **(h)** The synthesis process of CETIF6a-GSH. **(i)** The brightness after CETIF6a-GSH binds to the histone protein. **(J)** The covalent binding results of the probe protein complex in panel i.

The results of HPLC indicated that CETIF6a could form a covalent bond with GSH upon co-incubation, resulting in the formation of a new compound (**Fig. R8g**). When CETIF6a and GSH were co-incubated at a molar ratio of 1:3 at room temperature for 2 h, a compound named CETIF6a-GSH was formed with a yield of 25% (**Fig. R8h**) and was separated by reversed-phase preparative liquid chromatography. We then tested the tumor-targeting ability of CETIF6a-GSH. As shown in **Fig. R8i**, we found that the brightness of CETIF6a-GSH increased significantly after incubation with tissue proteins, and this increase in brightness was due to non-covalent binding of the probe to proteins (**Fig. R8j**), resulting in fluorescence similar to that of covalently bound CETIF6a.

Based on these results, we conclude that while the reaction between the probe and cellular metabolites may slightly enhance fluorescence, this enhancement is relatively minor. The product of the probe's reaction with cellular metabolites can still bind to proteins and produce fluorescence. Therefore, the primary source of fluorescence observed in our experiments is attributed to the binding of the probe to its target proteins. We have also supplied this explanation in the Discussion section. **Fig. R8e-f** have been added to the supplementary information as **Fig. S14**, accompanied by the following statement: "In addition to these tumor-upregulated proteins, whether high abundance metabolites in tumor cells can also bind to CETIF6a and contribute to fluorescence merits further discussion. To preliminarily explore this, we tested the binding of CETIF6a with glutathione, a metabolite commonly elevated in tumors (**Fig. S14**). Under identical binding conditions, the fluorescence brightness of the probe upon binding to glutathione was substantially lower than that observed upon binding to proteins, indicating that the probe achieves a significant increase in brightness primarily through recognition and binding to target proteins."

Reference

1 Weerapana, E., Speers, A. E. & Cravatt, B. F. Tandem orthogonal proteolysis-activity-based protein profiling (TOP-ABPP)—a general method for mapping sites of probe modification in proteomes. *Nature Protocols* **2**, 1414-1425, doi:10.1038/nprot.2007.194 (2007).

2 Yang, F., Jia, G., Guo, J., Liu, Y. & Wang, C. Quantitative chemoproteomic profiling with data-independent acquisition-based mass spectrometry. *Journal of The American Chemical Society* **144**, 901-911, doi:10.1021/jacs.1c11053 (2022).

3 Yoon, H. I. *et al.* Bioorthogonal copper free click chemistry for labeling and tracking of chondrocytes in vivo. *Bioconjugate Chemistry* **27**, 927-936, doi:10.1021/acs.bioconjchem.6b00010 (2016).

4 Hanahan, D. Hallmarks of cancer: new dimensions. *Cancer Discovery* **12**, 31-46, doi:10.1158/2159-8290.Cd-21-1059 (2022).

5 Hanahan, D. & Weinberg, R. A. Hallmarks of cancer: the next generation. *Cell* **144**, 646-674, doi:10.1016/j.cell.2011.02.013 (2011).

6 Beckford, G., Owens, E., Henary, M. & Patonay, G. The solvatochromic effects of side chain substitution on the binding interaction of novel tricarbocyanine dyes with human serum

albumin. *Talanta* **92**, 45-52, doi:10.1016/j.talanta.2012.01.029 (2012).

Supporting data

Synthesis route of IR-1B3：

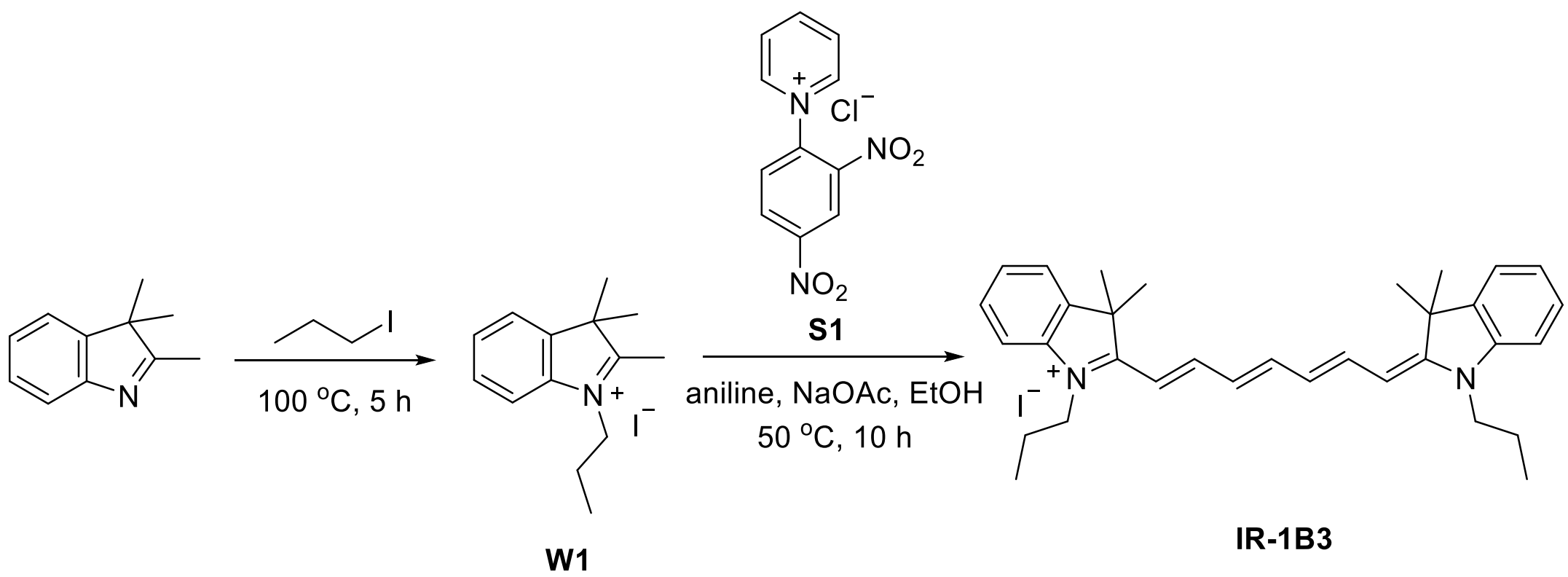

1-(2,4-dinitrophenyl)pyridin-1-ium chloride (S1)：

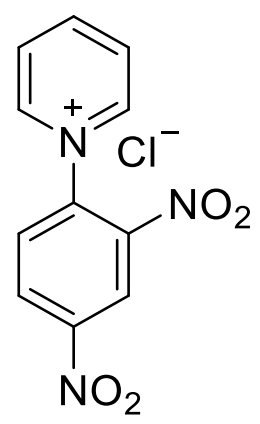

S1

Pyridine (1.00 mL, 13.36 mmol) and 2,4-dinitrochlorobenzene (2.56 g, 12.63 mmol) were dissolved in acetone (8.00 mL) The reaction mixture was heated at 57 °C for 12 hours. The precipitate was filtered, washed by *n*-hexane and dried in vacuum to afford **S1** as a yellow solid (2.80 g, 74.41% yield). ^{1}H NMR (400 MHz, DMSO-d_6) δ 9.52 (d, J = 6.0 Hz, 2H), 9.16 – 9.12 (m, 1H), 9.04 – 8.96 (m, 2H), 8.53 (d, J = 8.7 Hz, 1H), 8.48 (t, J = 7.2 Hz, 2H).

2,3,3-trimethyl-1-propyl-3*H*-indol-1-ium iodide (W1)：

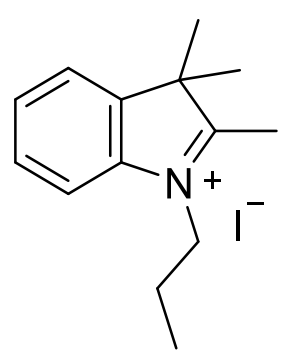

W1

2,3,3-trimethyldihydroindole (1.91 g, 12.00 mmol) was added to iodopropane (5.07 g, 30.00 mmol), and the reaction mixture was heated at 100 °C for 5 hours. After cooled to room temperature, ethyl acetate was added into the mixture, and the precipitate was filtered, washed by ethyl acetate, dried in vacuum to afford **W1** as a reddish brown solid (3.89 g, 98.48% yield). ^{1}H NMR (400 MHz, $CDCl_3$) δ 7.74 – 7.69 (m, 1H), 7.62 – 7.57(m, 3H), 4.70 (t, J = 7.6 Hz,

2H), 3.15 (s, 3H), 2.12 -1.99 (m, 2H), 1.68 (s, 6H), 1.11 (t, *J* = 7.6 Hz, 3H).

1. 2-((1*E*,3*E*,5*E*)-7-((*E*)-3,3-dimethyl-1-propylindolin-2-ylidene)hepta-1,3,5-trien-1-yl)-3,3-dimethyl-1-propyl-3*H*-indol-1-ium iodide (IR-1B3):

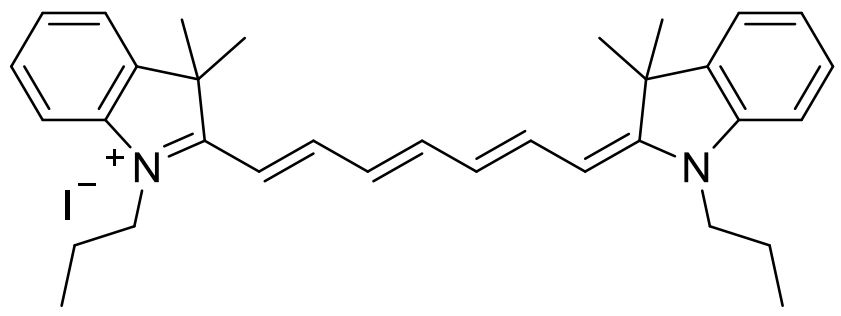

IR-1B3

Compound **S1** (105.63 mg, 0.375 mmol), compound **W1** (240.93 mg, 0.75 mmol), aniline (69.84 mg, 0.75 mmol) and sodium acetate (157.66 mg, 1.80 mmol) were dissolved in ethanol (6.00 mL). The reaction mixture was heated at 50 °C for 10 hours. After completion, the reaction mixture was cooled to room temperature. The solvent was removed under reduced pressure, and the residue was purified by chromatography on silica gel eluting with (dichloromethane: acetone = 100: 1, *v*/*v*) to afford **IR-1B3** as a red solid (45.00 mg, 20.25% yield). ^{1}H NMR (400 MHz, $CDCl_3$) δ 7.88 (t, *J* = 14.4 Hz, 2H), 7.80 – 7.70 (m, 1H), 7.43 – 7.35 (m, 4H), 7.23 (t, *J* =7.6 Hz, 2H), 7.09 (d, *J* = 8.0 Hz, 2H), 6.75 (t, *J* = 12.4 Hz, 2H), 6.32 (d, *J* = 13.4 Hz, 2H), 4.20 – 3.92 (m, 4H), 1.97 – 1.84 (m, 4H), 1.74 (s, 12H), 1.10 (t, *J* = 7.4 Hz, 6H). ^{13}C NMR (100 MHz, DMSO-d_6) δ 171.3, 151.1, 142.2, 140.9, 128.4, 125.3, 124.5, 122.4, 111.0, 103.7, 48.6, 44.8, 27.2, 20.4, 10.9. HRMS (ESI-TOF) m/z: calcd. for $[M]^+$ $C_{33}H_{41}N_2^+$ = 465.3264; found 465.3249.

Synthesis route of IR-2B3：

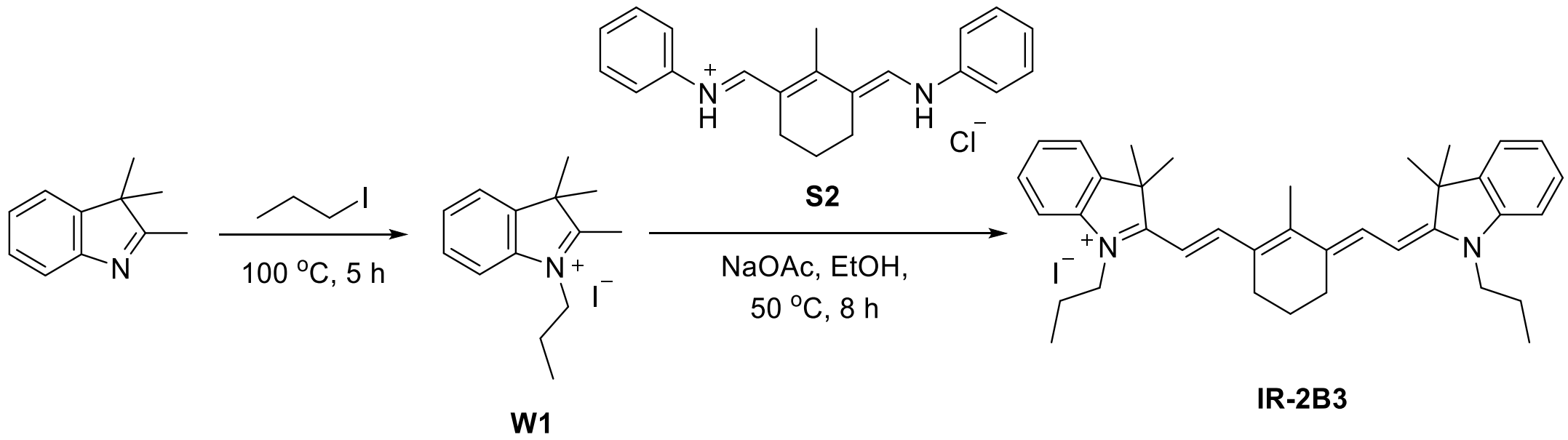

***N*-((*E*)-((*E*)-2-methyl-3-((phenylamino)methylene)cyclohex-1-en-1-yl)methylene)benzen-aminium chloride (S2)：**

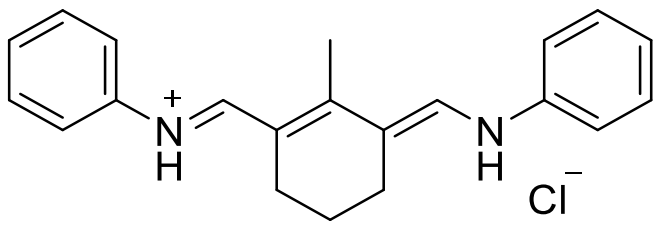

S2

Phosphorus oxychloride (2.80 mL, 30 mmol) was dropwise added into the solution of *N*-methyl-*N*-phenyl formamide (4.05 mL, 30 mmol) in chloroform (5.00 mL) at 0 °C. The mixture was stirred for 1 hour at 0 °C. 1-Methyl-1-cyclohexene (1.23 mL, 10 mmol) was dropwise added into the mixture. The reaction mixture was heated at 45 °C for 20 hours. After completion, the reaction mixture was cooled to room temperature. Distilled water (25.00 mL) was added into the mixture at 0 °C. Then potassium carbonate (3.30 g) was carefully added into the mixture, and stirred for 10 minutes. Subsequently, 5 mL aniline hydrochloride (2.96 g, 22.8 mmol) aqueous solution was added, and stirred for 30 minutes. After cooled, potassium carbonate (2.92 g) was added in batches, then the precipitate was filtered, washed by cold water and dried in vacuum to afford **S2** as a purple solid (1.56 g, 46.03% yield). ^{1}H NMR (400 MHz, MeOD) δ 8.46 (s, 2H), 7.52 – 7.41 (m, 8H), 7.29 – 7.22 (m, 2H), 2.63 – 2.52 (m, 7H), 1.99 – 1.89 (m, 2H).

2. 2-((*E*)-2-((*E*)-3-(2-((*E*)-3,3-dimethyl-1-propylindolin-2-ylidene)ethylidene)-2-methylcyclohex-1-en-1-yl)vinyl)-3,3-dimethyl-1-propyl-3*H*-indol-1-ium iodide (IR-2B3)：

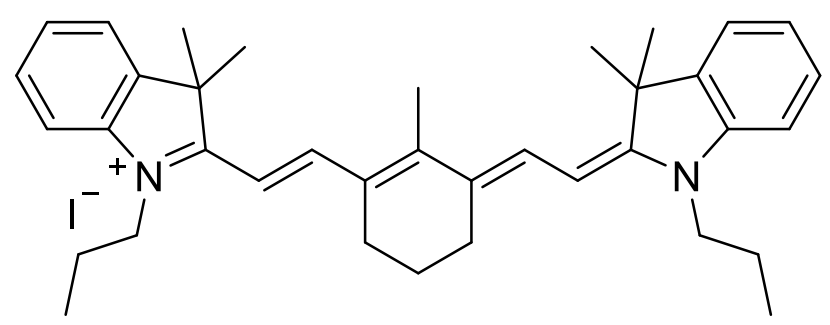

IR-2B3

Compound **S2** (33.89 mg, 0.20 mmol), compound **W1** (131.60 mg, 0.40 mmol) were dissolved in ethanol (4.00 mL), and then sodium acetate (32.82 mg, 0.40 mmol) was added into the solution. The reaction mixture was heated at 50 °C for 8 hours. After completion, the reaction mixture was cooled to room temperature. The solvent was removed under reduced pressure.

The residue was purified by chromatography on silica gel eluting with (dichloromethane: methanol = 50: 1, *v/v*) to afford **IR-2B3** as a green solid (50.96 mg, 39.40% yield). ^{1}H NMR (400 MHz, $CDCl_3$) δ 8.05 (d, *J* = 13.7 Hz, 2H), 7.41 – 7.33 (m, 4H), 7.22 (t, *J* = 7.6 Hz, 2H), 7.12 (d, *J* = 8.0 Hz, 2H), 6.15 (d, *J* = 13.7 Hz, 2H), 4.09 (t, *J* = 7.3 Hz, 4H), 2.62 – 2.54 (m, 4H), 2.42 (s, 3H), 1.98 – 1.83 (m, 6H), 1.72 (s, 12H), 1.07 (t, *J* = 7.4 Hz, 6H). ^{13}C NMR (100 MHz, $CDCl_3$) δ 171.3, 156.2, 142.6, 142.5, 140.7, 131.6, 128.8, 124.9, 122.4, 110.7, 100.3, 49.1, 46.1, 28.6, 25.7, 21.1, 20.8, 15.3, 11.8. HRMS (ESI-TOF) m/z: calcd. for $[M]^+$ $C_{37}H_{47}N_2^+$ = 519.3734; found 519.3727.

3. Synthesis route of IR-3B3：

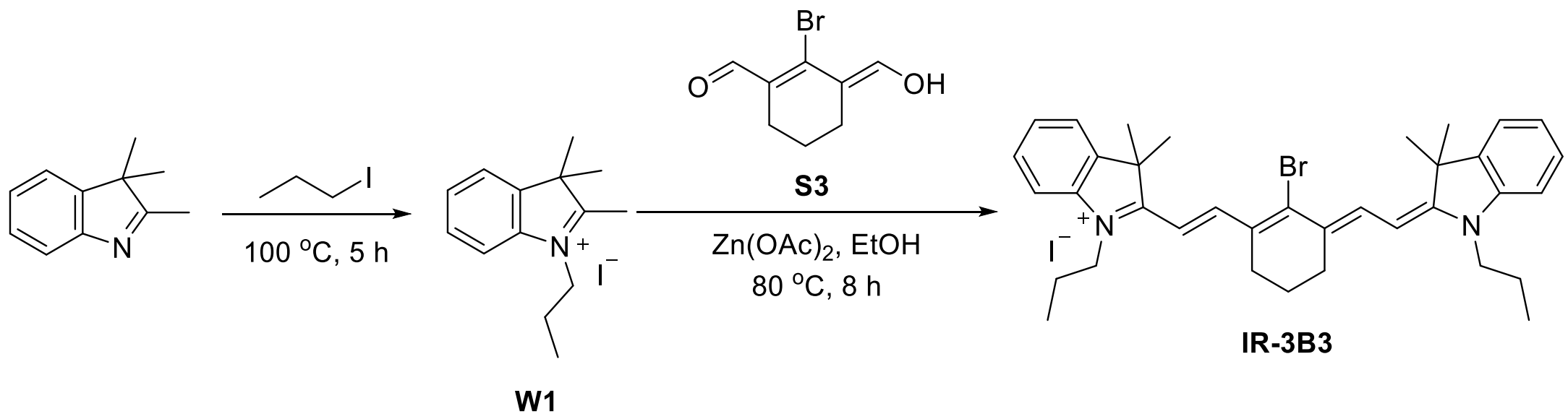

(*E*)-2-bromo-3-(hydroxymethylene)cyclohex-1-ene-1-carbaldehyde (S3)：

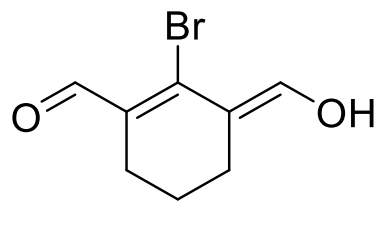

S3

Phosphorus oxybromide (4.50 g, 15.70 mmol) was added in bath to *N*,*N*-dimethylformamide (25.00 mL) at 0 °C and stirred at room temperature for 1 hour. The precipitate was filtered, washed by *N*,*N*-dimethylformamide, the white solid was removed to another flask. Cyclohexanone (0.50 g, 5.09 mmol) was added to this flask and stirred for 10 minutes at room temperature. Then the mixture was heated at 100 °C for 1 hour. After completion, the reaction mixture was cooled to room temperature. The mixture was poured into ice-water mixture (100.00 mL), stirred for another 1 hour, and sit overnight. The precipitate was filtered and washed by cold water to afford **S3** as a brown yellow solid (483.2 mg, 43.73% yield). ^{1}H NMR (400 MHz, DMSO-d_6) δ 11.32 – 10.50 (m, 1H), 10.29 – 9.54 (m, 1H), 7.92 – 7.28 (m, 1H), 2.46 – 2.25 (m, 4H), 1.64 -1.50 (m, 2H).

2-((*E*)-2-((*E*)-2-bromo-3-(2-((*E*)-3,3-dimethyl-1-propylindolin-2-ylidene)ethylidene)cyclohex-1-en-1-yl)vinyl)-3,3-dimethyl-1-propyl-3*H*-indol-1-ium iodide (IR-3B3)：

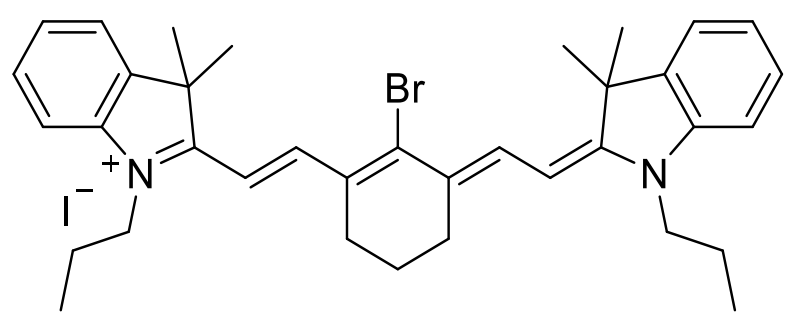

IR-3B3

Compound **S3** (21.70 mg, 0.10 mmol), compound **W1** (65.84 mg, 0.20 mmol) were dissolved in ethanol (2.00 mL), and then zinc acetate (36.69 mg, 0.2mmol) was added into the solution. The reaction mixture was heated at 80 °C for 8 hours. After completion, the reaction mixture was cooled to room temperature. The solvent was removed under reduced pressure. The residue was purified by chromatography on silica gel eluting with (dichloromethane: methanol = 50: 1, *v/v*) to afford **IR-3B3** as a green solid (37.00 mg, 51.99% yield). ^{1}H NMR (400 MHz,

DMSO-d_6) δ 8.28 (d, J = 14.1 Hz, 2H), 7.65 (d, J = 7.3 Hz, 2H), 7.51 – 7.41 (m, 4H), 7.30 (t, J = 7.3 Hz, 2H), 6.36 (d, J = 14.2 Hz, 2H), 4.21 (t, J = 7.6 Hz, 4H), 2.79 – 2.67 (m, 4H), 1.90 – 1.83 (m, 2H), 1.82 -1.73 (m, 4H), 1.70 (s, 12H), 0.97 (t, J = 7.4 Hz, 6H). ^{13}C NMR (100 MHz, $CDCl_3$) δ 172.6, 147.9, 147.0, 142.3, 141.1, 129.2, 128.8, 125.4, 122.3, 111.1, 101.8, 49.4, 46.5, 28.2, 27.7, 20.9, 20.8, 11.7. HRMS (ESI-TOF) m/z: calcd. for $[M]^+$ $C_{36}H_{44}BrN_2^+$ = 583.2682; found 583.2666.

Synthesis route of IR-4B3：

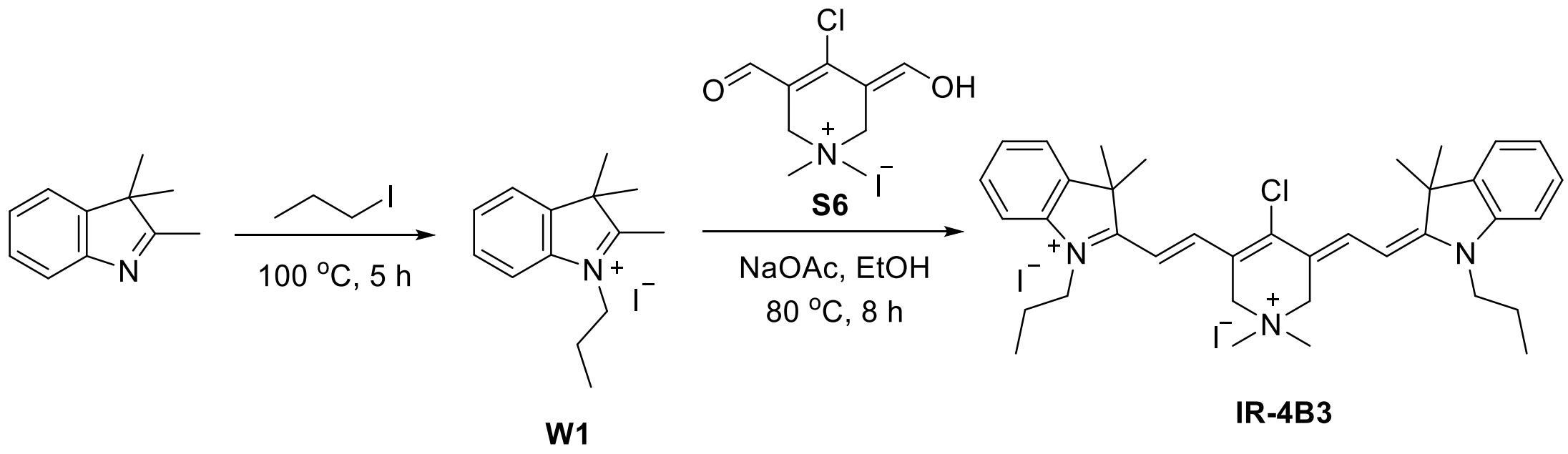

1,1-dimethyl-4-oxopiperidin-1-ium iodide (S4)：

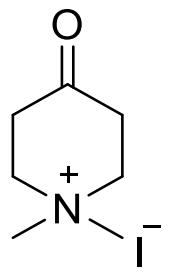

S4

N-methyl-4-piperidone (995.81 mg, 8.80 mmol) was dissolved in acetone (10.00 mL), and methyl iodide (2.75 g, 19.36 mmol) was added into the solution. The reaction mixture was heated at 25 °C for 4 hours. The precipitate was filtered, and washed by acetone to afford **S4** as a white solid (2.54 g, 100.00% yield). ^{1}H NMR (400 MHz, DMSO-d_6) δ 3.78 (t, J = 6.6 Hz, 4H), 3.30 (s, 6H), 2.73 (t, J = 6.6 Hz, 4H).

(*E*)-4-chloro-5-formyl-3-(hydroxymethylene)-1,1-dimethyl-1,2,3,6-tetrahydropyridin-1-ium iodide (S5)：

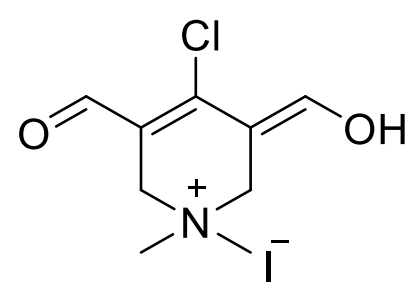

S5

Phosphorus oxychloride (5.50 mL, 30.00 mmol) was dropwise added into *N*,*N*-dimethylformamide (7.50 mL, 10.00 mmol) at 0 °C and stirred for another 30 minutes. Then compound **S5** (1.126 g, 5 mmol) was added into the solution. The mixture was heated at 80 °C for 3 hours. After completion, the reaction mixture was cooled to room temperature. 2 M hydrochloric acid aqueous solution (6 mL) was added into the mixture, and stirred at room temperature for 1 hour. Then the mixture was treated with ultrasonic for 20 minutes, and left in the refrigerator (-20 °C) overnight. The precipitate was filtered, washed by cold water, and dried in vacuum to afford **S5** as a brown yellow solid (0.11 g, 6.67% yield). ^{1}H NMR (400 MHz, D_2O) δ 8.95 (s, 2H), 4.22 (s, 4H), 3.12 (s, 6H).

2-((*E*)-2-((*E*)-4-chloro-5-(2-((*E*)-3,3-dimethyl-1-propylindolin-2-ylidene)ethylidene)-1,1-

dimethyl-1,2,5,6-tetrahydropyridin-1-ium-3-yl)vinyl)-3,3-dimethyl-1-propyl-3*H*-indol-1-ium iodide (IR-4B3)：

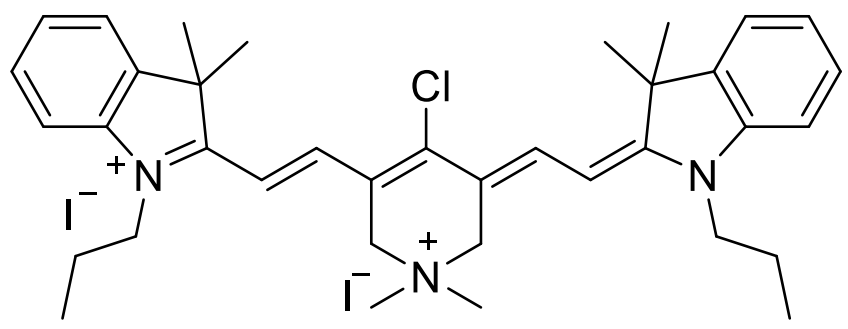

IR-4B3

Compound **S5** (98.88 mg, 0.30 mmol), compound **W1** (197.55 mg, 0.60 mmol) were dissolved in ethanol (6.00 mL), and then sodium acetate (49.23 mg, 0.60 mmol) was added into the solution. The reaction mixture was heated at 80 °C for 8 hours. After completion, the reaction mixture was cooled to room temperature. The solvent was removed under reduced pressure. The residue was purified by chromatography on silica gel eluting with (dichloromethane: methanol = 50: 1, *v/v*) to afford **IR-4B3** as a green solid (46.68 mg, 19.18% yield). ^{1}H NMR (400 MHz, $CDCl_3$) δ 8.31 (d, J = 14.7 Hz, 2H), 7.47 – 7.30 (m, 4H), 7.36 – 7.30 (m, 2H), 7.28 – 7.22 (m, 2H), 6.76 (d, J = 14.7 Hz, 2H), 5.35 – 5.27 (m, 4H), 4.58 (t, J = 7.4 Hz, 4H), 3.87 (s, 6H), 2.04 – 1.91 (m, 4H), 1.74 (s, 12H), 1.15 (t, J = 7.4 Hz, 6H). ^{13}C NMR (100 MHz, $CDCl_3$) δ 175.3, 144.3, 144.2, 142.1, 141.8, 129.1, 126.4, 122.3, 112.9, 112.2, 103.0, 61.9, 52.3, 49.9, 47.3, 28.2, 21.6, 11.8. HRMS (ESI-TOF) m/z: calcd. for $[M]^{2+}$ $C_{37}H_{48}ClN_3^{2+}$ = 284.6763; found 284.6745.

Synthesis route of IR-5B3：

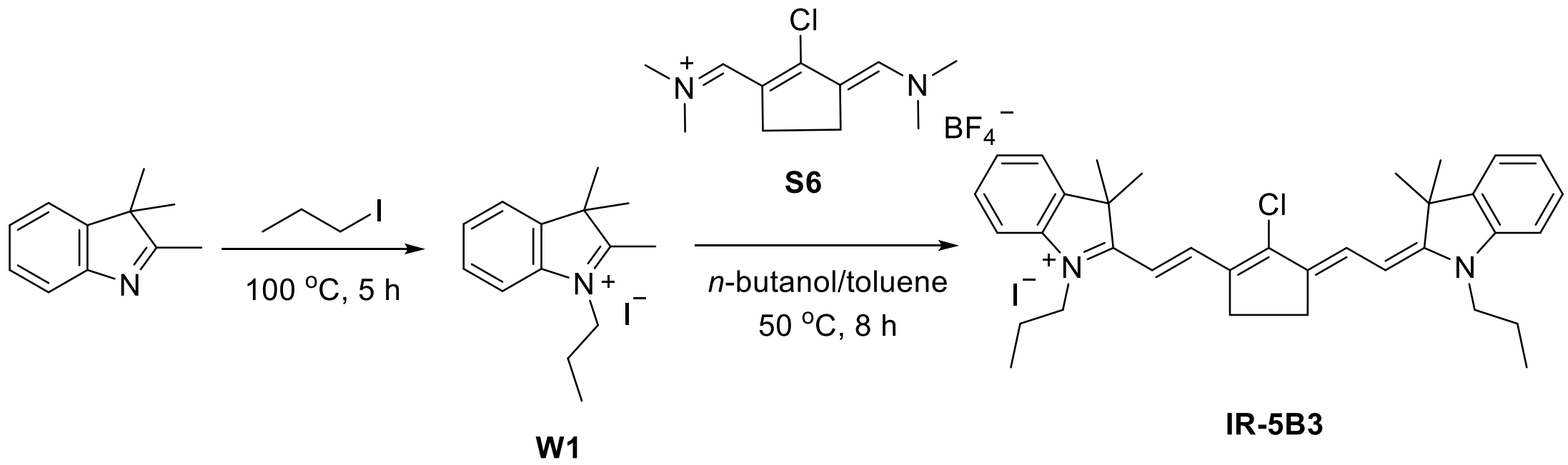

(*E*)-*N*-((2-chloro-3-((dimethylamino)methylene)cyclopent-1-en-1-yl)methylene)-*N*-methylmethanaminium tetrafluoroborate (S6)：

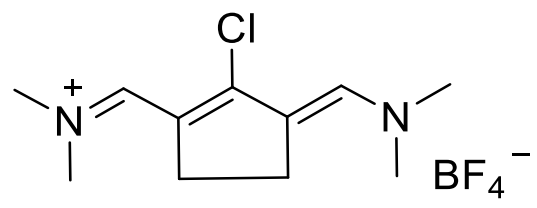

S6

Phosphorus oxychloride (1.60 mL, 17.50 mmol) was dropwise added into the mixture solution of *N,N*-dimethylformamide (1.96 mL) and dichloromethane (4.00 mL) at 0 °C. The reaction mixture was stirred for 1 hour at 0 °C. Then, cyclopentanone (0.44 g, 5.00 mmol) was dropwise added into the mixture, the reaction mixture was heated at 60 °C for 5 hours. After completion, the reaction mixture was cooled to room temperature. Subsequently, 40 mL $NaBF_4$ (1.79 g, 16.30 mmol) aqueous solution was added. The precipitate was filtered, washed by cold water and dried in vacuum to afford **S6** as a red solid (0.54 g, 35.93% yield). ^1H NMR (400 MHz, $CDCl_3$) δ 7.53 (s, 2H), 3.38 (s, 12H), 3.18 (s, 4H).

4. 2-((*E*)-2-((*E*)-2-chloro-3-(2-((*E*)-3,3-dimethyl-1-propylindolin-2-ylidene)ethylidene)cyclopent-1-en-1-yl)vinyl)-3,3-dimethyl-1-propyl-3*H*-indol-1-ium iodide (IR-5B3)：

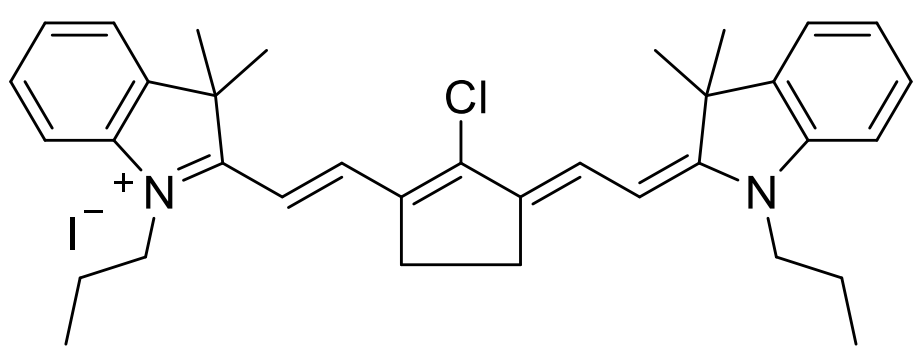

IR-5B3

Compound **S6** (90.15 mg, 0.30 mmol) and compound **W1** (197.40 mg, 0.60 mmol) were dissolved in *n*-butanol/toluene (*v/v*, 7:3, 6.00 mL). The reaction mixture was heated at 50 °C for 8 hours. After completion, the reaction mixture was cooled to room temperature. Then *n*-hexane (30.00 mL) was added, and the precipitate was filtered, washed by *n*-hexane (45.00 mL). The obtained crude product was purified by chromatography on silica gel eluting with (dichloromethane: methanol = 50: 1, *v/v*) to afford **IR-5B3** as a red solid (42.63 mg, 21.69%

yield). ^{1}H NMR (400 MHz, $CDCl_3$) δ 7.82 (d, *J* = 14.0 Hz, 2H), 7.42 – 7.34 (m, 4H), 7.28 – 7.19 (m, 2H), 7.12 (d, *J* = 7.9 Hz, 2H), 6.09 (d, *J* = 14.0 Hz, 2H), 4.09 (t, *J* = 7.3 Hz, 4H), 3.00 (s, 4H), 1.97 – 1.84 (m, 4H), 1.71 (s, 12H), 1.06 (t, *J* = 7.4 Hz, 6H). ^{13}C NMR (100 MHz, DMSO-d_6) δ 171.5, 149.7, 142.8, 141.5, 137.1, 136.1, 129.2, 125.6, 122.9, 112.0, 103.1, 49.4, 45.6, 27.9, 26.5, 21.0, 11.6. HRMS (ESI-TOF) m/z: calcd. for $[M]^+$ $C_{35}H_{42}ClN_2^+$ = 525.3031; found 525.3021.

Synthesis route of IR-6N3：

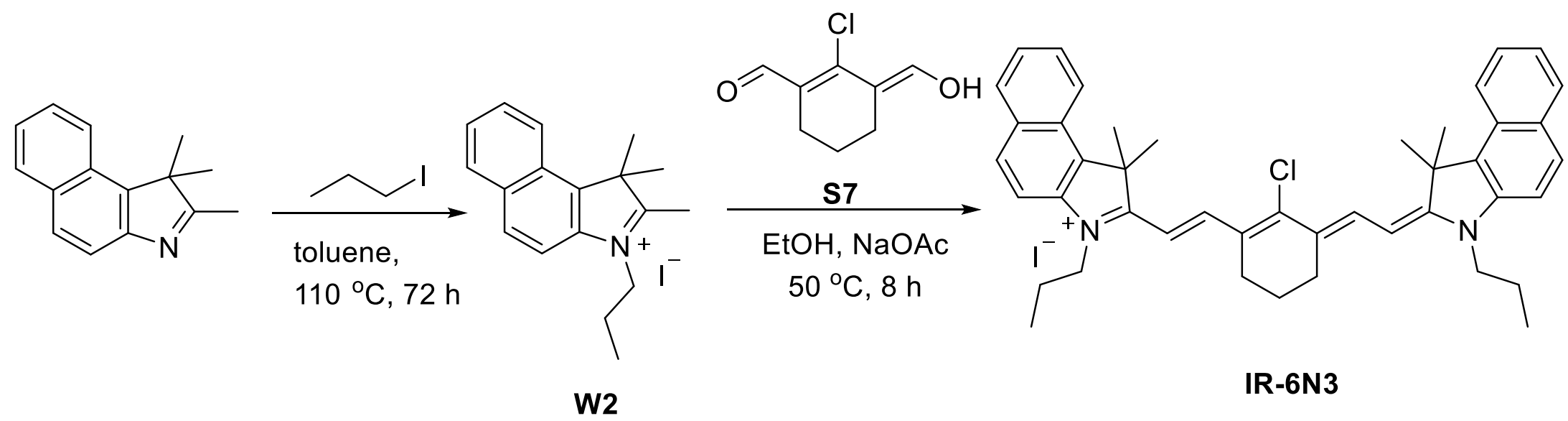

W2

IR-6N3

(*E*)-2-chloro-3-(hydroxymethylene)cyclohex-1-ene-1-carbaldehyde (S7)：

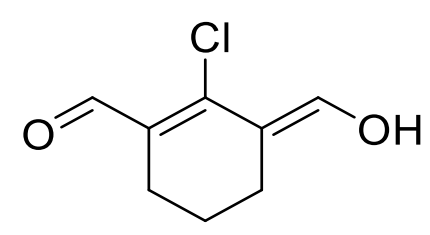

S7

A solution of phosphorus oxychloride (4.40 mL, 47.60 mmol) in dichloromethane (5.00 mL) was dropwise added into the mixture solution of *N,N*-dimethylformamide (5.00 mL) and dichloromethane (5.00 mL) at 0 °C. After stirred for 30 minutes at 0 °C, the cyclohexanone (1.32 mL, 12.6 mmol) was added into the mixture. The reaction solution was heated at 60 °C for 5 hours at argon atmosphere. After completion, the reaction mixture was cooled to room temperature, then poured into ice-water mixture (100.00mL), and allowed to stand overnight. Then the precipitate was filtered, washed by acetone and cold water, dried in vacuum to afford compound **S7** as a yellow solid (1.24 g, 57.01% yield). ^{1}H NMR (400 MHz, DMSO-d_6) δ 10.88 (s, 1H), 10.12 (s, 1H), 7.55 (s, 1H), 2.46 – 2.23 (m, 4H), 1.64 – 1.54 (m, 2H).

1,1,2-trimethyl-3-propyl-1*H*-benzo[*e*]indol-3-ium iodide (W2)：

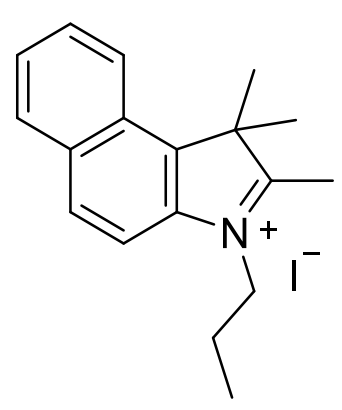

W2

Iodopropane (4.71 g, 27.72 mmol) was added to a solution of 1,1,2-trimethyl-1*H*-benzoindole (0.98 g, 8.40 mmol) in toluene (10.00 mL). The reaction mixture was heated at 110 °C for 72 hours. The precipitate was filtered, washed by toluene for three times and dried in vacuum to afford **W2** as a dusty blue solid (1.45 g, 45.51% yield). ^{1}H NMR (400 MHz, $CDCl_3$) δ 8.13 -8.08 (m, 2H), 8.56 (d, *J* = 7.6 Hz, 1H), 7.83 (d, *J* = 9.2 Hz, 1H), 7.75 (t, *J* = 7.7 Hz, 1H), 7.67 (t, *J* = 8.2 Hz, 1H), 4.81 (t, *J* = 7.5 Hz, 2H), 3.23 (s, 3H), 2.15 – 2.04 (m, 2H), 1.89 (s, 6H), 1.12 (t, *J* = 7.4 Hz, 3H).

5. 2-((*E*)-2-((*E*)-2-chloro-3-((*E*)-2-(1,1-dimethyl-3-propyl-1,3-dihydro-2*H*-benzo[*e*]indol-2-ylidene)-ethylidene)cyclohex-1-en-1-yl)vinyl)-1,1-dimethyl-3-propyl-1*H*-benzo[*e*]indol-3-ium (IR-6N3)：

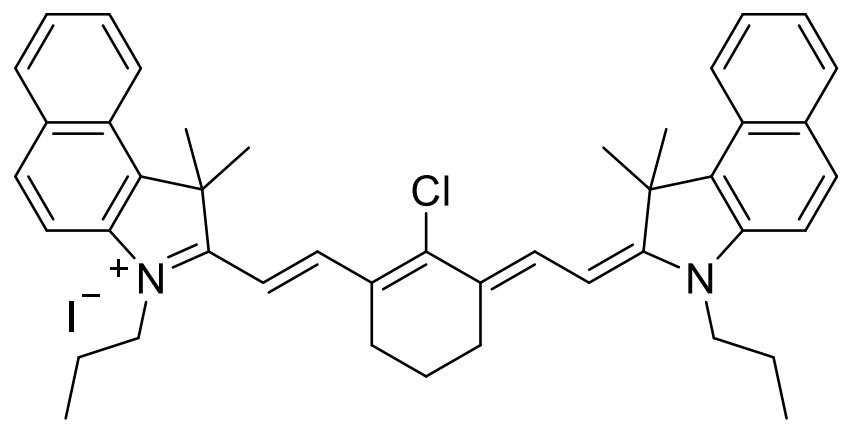

IR-6N3

Compound **S7** (17.20 mg, 0.10 mmol) and compound **W2** (75.86 mg, 0.20 mmol) were dissolved in ethanol (2.00 mL), and then sodium acetate (16.41 mg, 0.20 mmol) was added into the solution. The mixture solution was heated at 50 °C for 8 hours. Then *n*-hexane (30.00 mL) was added, and the precipitate produced was filtered and washed by *n*-hexane (15.00 mL). The crude product was purified by chromatography on silica gel eluting with (dichloromethane: methanol = 50: 1, *v*/*v*) to afford **IR-6N3** as a blue solid (33.24 mg, 43.33% yield). ^{1}H NMR (400 MHz, $CDCl_3$) δ 8.45 (d, *J* = 14.2 Hz, 2H), 8.14(d, *J* = 8.8 Hz, 2H), 7.95 (d, *J* = 8.5 Hz, 4H), 7.62 (t, *J* = 8.0 Hz, 2H), 7.52 – 7.42 (m, 4H), 6.30 (d, *J* = 14.2 Hz, 2H), 4.33 (t, *J* = 7.3 Hz, 4H), 2.86 – 2.75 (m, 4H), 2.03 (s, 12H), 2.02 – 1.93 (m, 4H), 1.62 – 1.57 (m, 2H), 1.11 (t, *J* = 7.4 Hz, 6H). ^{13}C NMR (100 MHz, $CDCl_3$) δ 173.8, 149.9, 143.3, 139.8, 133.8, 131.9, 130.8, 130.2, 128.1, 127.8, 127.2, 125.2, 122.1, 110.9, 100.9, 51.17, 46.6, 27.8, 26.8, 21.2, 20.8, 11.7. HRMS (ESI-TOF) m/z: calcd. for $[M]^+$ $C_{44}H_{48}ClN_2^+$ = 639.3501; found 639.3501.

Synthesis route of IR-6B3：

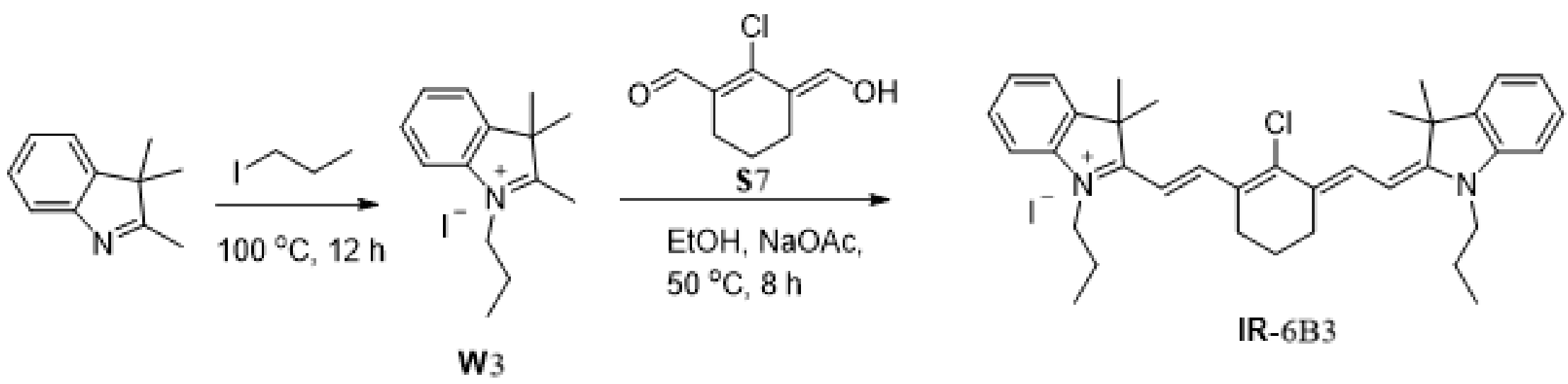

2,3,3-trimethyl-1-propyl-3*H*-indol-1-ium iodide (W3):

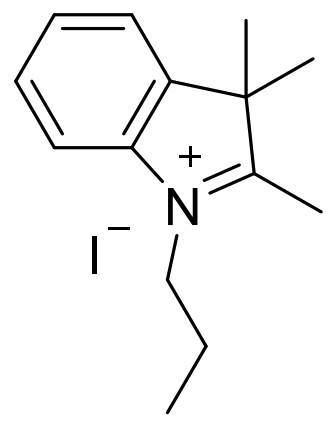

W3

In a round-bottom flask, add 2,3,3-trimethyl-3*H*-indole (1.59 g, 10 mmol) and 1-iodopropane (4.25 g, 25 mmol). Stir at 100 °C for 12 hours until complete reaction is achieved, then cool to room temperature. Add ethyl acetate (100 mL) to the reaction mixture and filter to collect the precipitate. Wash the solid with ethyl acetate (3×100 mL), dry it, and obtain a light beige solid weighing 2.75 g (yield: 83.5%). ^{1}H NMR (400 MHz, Chloroform-*d*) δ 7.64 (d, J = 6.8 Hz, 1H), 7.56 (m, 3H), 4.67 (t, J = 6.9 Hz, 2H), 3.11 (dd, J = 4.4, 2.1 Hz, 3H), 2.00 (q, J = 7.5 Hz, 2H), 1.64 (dd, J = 2.9, 1.5 Hz, 6H), 1.08 (m, 3H).

2-((E)-2-((E)-2-chloro-3-(2-((E)-3,3-dimethyl-1-propylindolin-2-ylidene)ethylidene)cyclohex-1-en-1-yl)vinyl)-3,3-dimethyl-1-propyl-3*H*-indol-1-ium iodide (IR-6B3)：

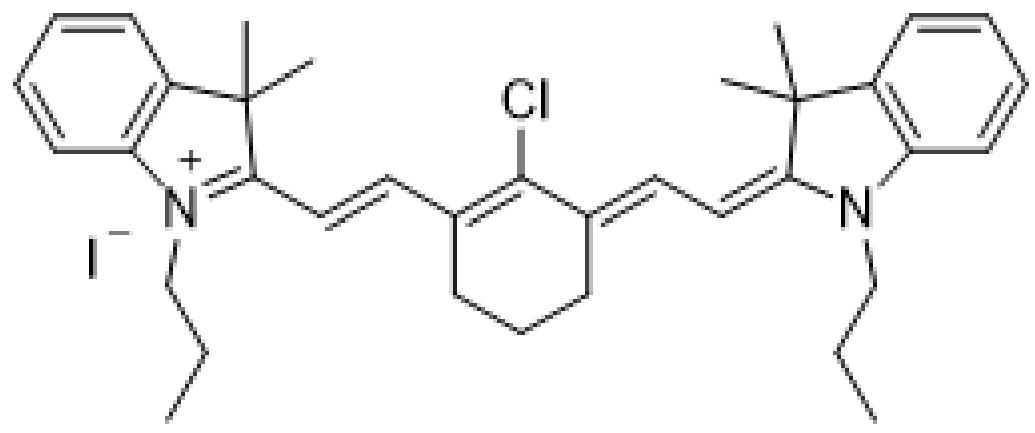

IR-6B3

Compounds **W3** (329 mg, 1 mmol), **S7** (86 mg, 0.5 mmol), and NaOAc (82 mg, 1 mmol) were dissolved in EtOH (10 mL). The mixture was stirred at 50℃ for 8 hours. After the reaction, the

solvent was removed under reduced pressure. The crude product was purified by silica gel column chromatography using a DCM:MeOH ratio of 50:1 (v/v), yielding a yellow-green solid 121 mg (yield 50.4%). ^{1}H NMR (400 MHz, Chloroform-*d*) δ 8.34 (d, *J* = 14.1 Hz, 2H), 7.40 (t, *J* = 7.6 Hz, 4H), 7.24 (d, *J* = 7.5 Hz, 2H), 7.18 (d, *J* = 7.9 Hz, 2H), 6.26 (d, *J* = 14.1 Hz, 2H), 4.20 (t, *J* = 7.3 Hz, 4H), 2.75 (t, *J* = 6.2 Hz, 4H), 2.03 – 1.97 (m, 2H), 1.93 (q, *J* = 7.4 Hz, 4H), 1.73 (s, 12H), 1.08 (t, *J* = 7.4 Hz, 6H). ^{13}C NMR (100 MHz, Chloroform-*d*) δ 172.52, 150.57, 144.37, 142.36, 141.01, 128.85, 127.25, 125.37, 122.33, 111.09, 101.35, 49.40, 46.41, 28.21, 26.70, 20.95, 20.71, 11.75. HRMS (ESI-TOF) m/z: calcd. for $[M]^+$ $C_{36}H_{44}ClN_2^+$ = 539.3188; found 539.3161.

Synthesis route of IR-6B5：

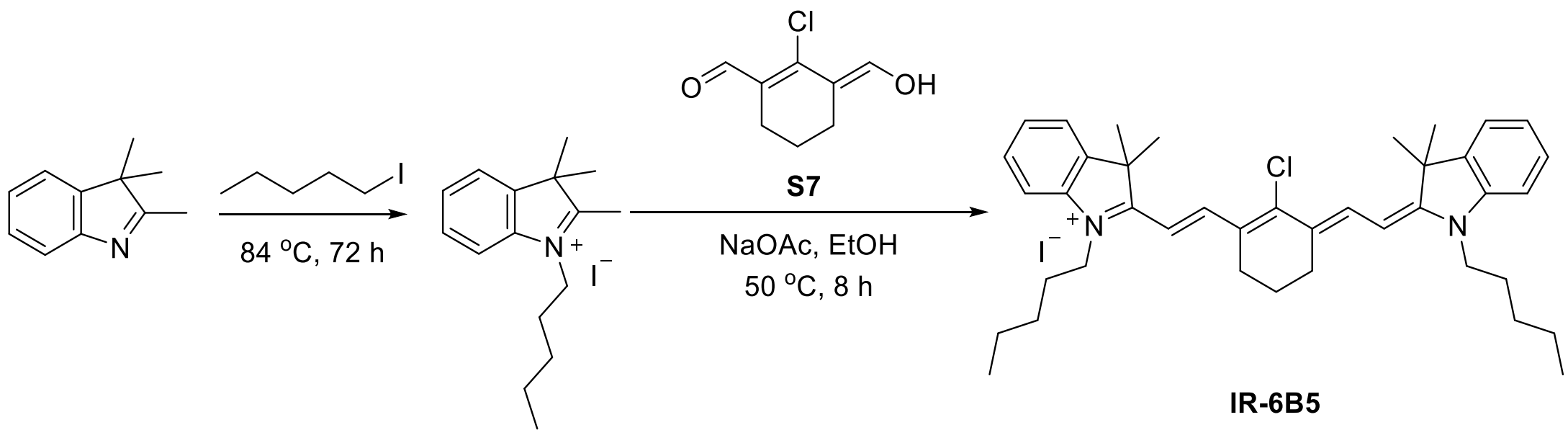

W4

2,3,3-trimethyl-1-pentyl-3H-indol-1-ium iodide (W4)：

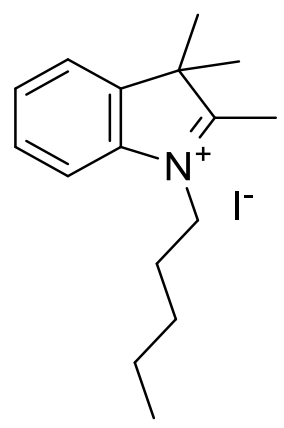

W4

2,3,3-trimethyldihydroindole (1.27 g, 8.00 mmol) and 1-iodopentane (2.94 g, 10 mmol) were dissolved in acetonitrile (5.00 mL), and the solution was heated at 84 °C for 72 hours. After completion, the reaction mixture was cooled to room temperature. The solvent was removed under reduced pressure, and ethyl acetate was added. The precipitate was filtered, washed by ethyl acetate to afford W4 as a purple solid (1.99 g, 69.63% yield). ^{1}H NMR (400 MHz, DMSO-d_6) δ 8.00 – 7.95 (m, 1H), 7.88 – 7.82 (m, 1H), 7.67 – 7.60 (m, 2H), 4.45 (d, J = 8.0 Hz, 2H), 2.84 (s, 3H), 1.89 – 1.79 (m, 2H), 1.54 (s, 6H), 1.45 – 1.31 (m, 4H), 0.89 (t, J = 7.8 Hz, 3H).

2-((*E*)-2-((*E*)-2-chloro-3-(2-((*E*)-3,3-dimethyl-1-pentylindolin-2-ylidene)ethylidene)cyclohex-1-en-1-yl)vinyl)-3,3-dimethyl-1-pentyl-3*H*-indol-1-ium iodide (IR-6B5)：

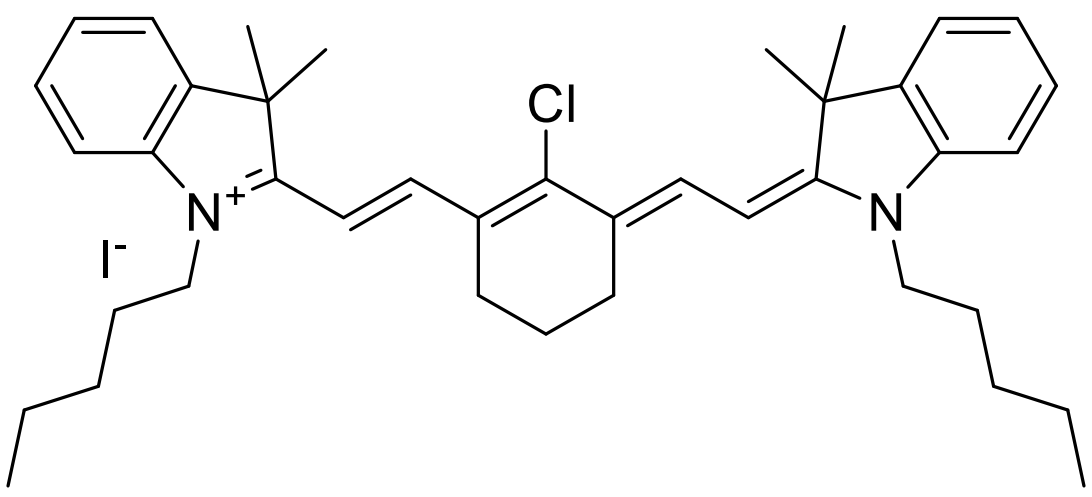

IR-6B5

Compound **S7** (17.20 mg, 0.10 mmol), compound **W4** (70.86 mg, 0.20 mmol) were dissolved in ethanol (2.00 mL), and then sodium acetate (16.41 mg, 0.20 mmol) was added into the solution.

The reaction mixture was heated at 50 °C for 8 hours. After completion, the reaction mixture

was cooled to room temperature. The solvent was removed under reduced pressure, and the residue was purified by chromatography on silica gel eluting with (dichloromethane: methanol = 50: 1, *v/v*) to afford **IR-6B5** as a green solid (33.24 mg, 43.33% yield). ^{1}H NMR (400 MHz, $CDCl_3$) δ 8.34 (d, J = 14.1 Hz, 2H), 7.43 – 7.36 (m, 4H), 7.28 – 7.22 (m, 2H), 7.17 – 7.15 (d, J = 7.9 Hz, 2H), 6.24 (d, J = 14.1 Hz, 2H), 4.20 (t, J = 7.5 Hz, 4H), 2.80 – 2.70 (m, 4H), 2.05 - 1.96 (m, 2H), 1.91 – 1.81 (m, 4H), 1.73 (s, 12H), 1.49 – 1.36 (m, 8H), 0.93 (t, J = 6.9 Hz, 6H). ^{13}C NMR (100 MHz, $CDCl_3$) δ 172.5, 150.6, 144.4, 142.2, 141.1, 128.9, 127.3, 125.5, 122.4, 111.1, 101.4, 49.5, 45.1, 29.2, 28.2, 27.2, 26.7, 22.5, 20.8, 13.9. HRMS (ESI-TOF) m/z: calcd. for $[M]^+$ $C_{40}H_{52}ClN_2^+$ = 595.3814; found 595.3807.

Synthesis route of IR-6B7：

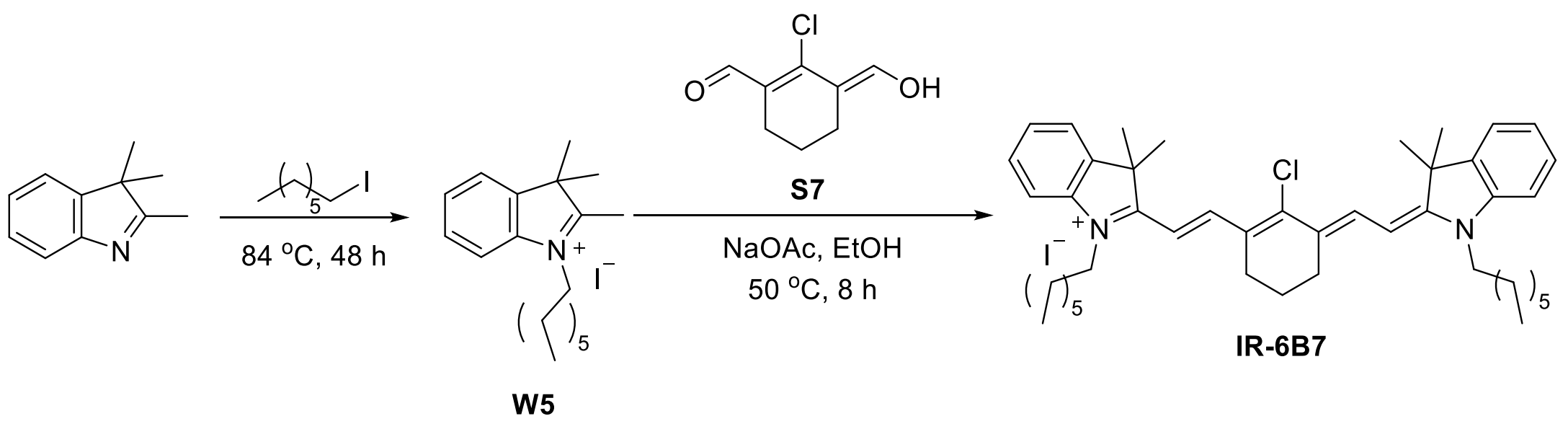

1-heptyl-2,3,3-trimethyl-3*H*-indol-1-ium iodide (W5)：

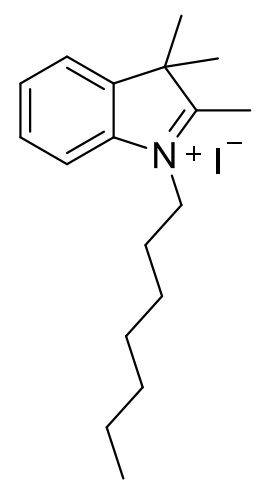

W5

2,3,3-trimethyldihydroindole (1.59 g, 10.00 mmol) and 1-iodoheptane (2.96 g, 13 mmol) were dissolved in acetonitrile (5.00 mL), and the reaction mixture was heated at 84 °C for 48 hours. After completion, the reaction mixture was cooled to room temperature. The solvent was removed under reduced pressure, and ethyl acetate was added. The precipitate was filtered, washed by ethyl acetate to afford W5 as a reddish brown solid (3.26 g, 84.68% yield). ^{1}H NMR (400 MHz, $CDCl_3$) δ 7.67 – 7.62 (m, 1H), 7.61 – 7.57 (m, 3H), 4.69 (t, J = 8.0 Hz, 2H), 3.14 (s, 3H), 1.99 – 1.87 (m, 2H), 1.67 (s, 6H), 1.52 – 1.42 (m, 2H), 1.42 – 1.33 (m, 2H), 1.33 – 1.24 (m, 4H), 0.88 (t, J = 6.8 Hz, 3H).

2-((*E*)-2-((*E*)-2-chloro-3-(2-((*E*)-1-heptyl-3,3-dimethylindolin-2-ylidene)ethylidene)cyclohex-1-en-1-yl)vinyl)-1-heptyl-3,3-dimethyl-3*H*-indol-1-ium iodide (IR-6B7)：

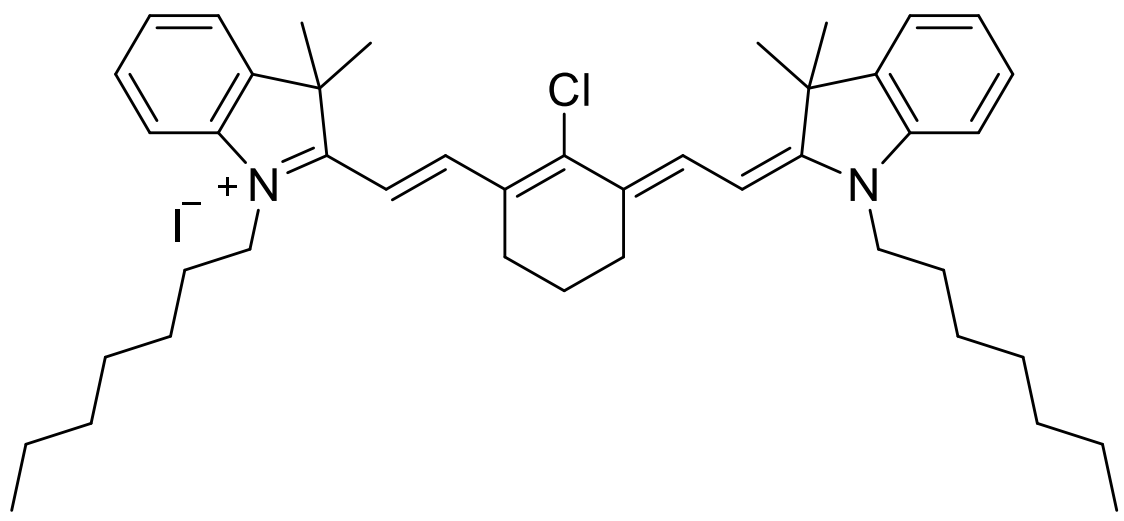

IR-6B7

Compound **S7** (34.40 mg, 0.20 mmol), compound **W5** (154.06 mg, 0.40 mmol) were dissolved in ethanol (4.00 mL), and then sodium acetate (32.82 mg, 0.40 mmol) was added into the solution.

The reaction mixture was heated at 50 °C for 8 hours. After completion, the reaction mixture was cooled to room temperature. The solvent was removed under reduced pressure, and the residue was purified by chromatography on silica gel eluting with (dichloromethane: methanol = 50: 1, *v/v*) to afford **IR-6B5** as a green solid (46.28mg, 29.69% yield). ^{1}H NMR (400 MHz, $CDCl_3$) δ 8.34 (d, *J* = 14.1 Hz, 2H), 7.43 – 7.36 (m, 4H), 7.30 – 7.22 (m, 2H), 7.16 (d, *J* = 8.0 Hz, 2H), 6.24 (d, *J* = 14.1 Hz, 2H), 4.20 (t, *J* = 7.4 Hz, 4H), 2.81 – 2.69 (m, 4H), 2.04 – 1.95 (m, 2H), 1.91 – 1.80 (m, 4H), 1.73 (s, 12H), 1.51 – 1.43 (m, 4H), 1.42 – 1.34 (m, 4H), 1.34 – 1.21 (m, 8H), 0.88 (t, *J* = 7.2 Hz, 6H). ^{13}C NMR (100 MHz, $CDCl_3$) δ 172.4, 150.5, 144.3, 142.3, 141.1, 128.8, 127.3, 125.4, 122.3, 110.9, 101.4, 49.4, 45.1, 31.6, 28.9, 28.2, 27.4, 26.9, 26.7, 22.6, 20.7, 14.0. HRMS (ESI-TOF) m/z: calcd. for $[M]^+$ $C_{44}H_{60}ClN_2^+$ = 651.4440; found 651.4440.

Synthesis route of IR-6B9：

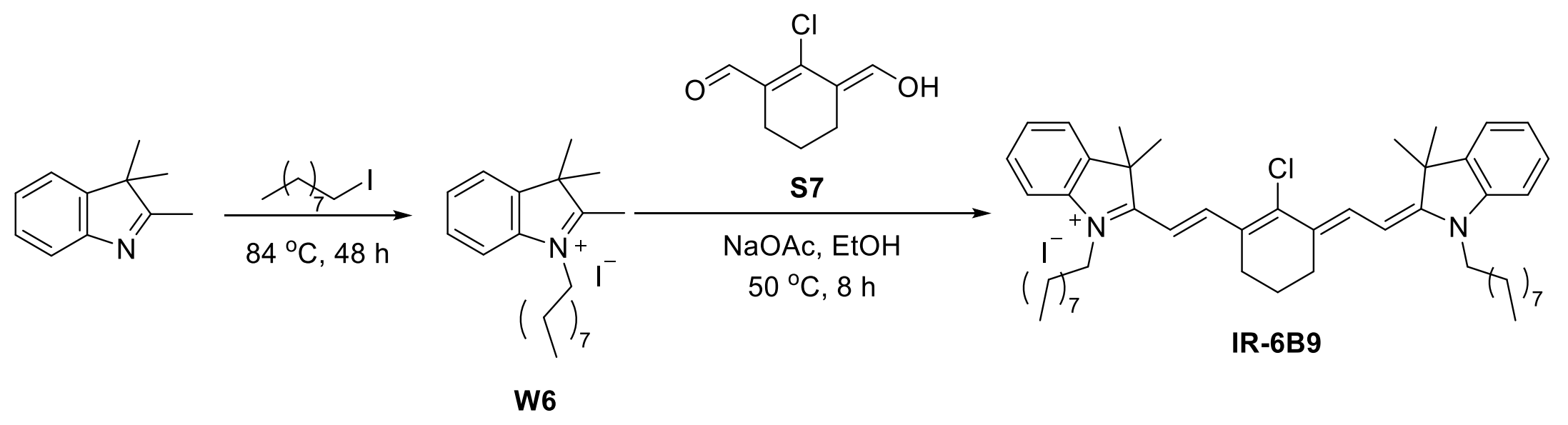

2,3,3-trimethyl-1-nonyl-3*H*-indol-1-ium iodide (W6)：

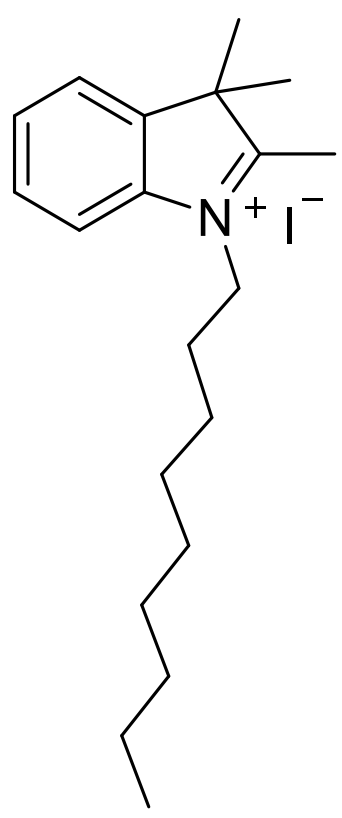

W6

2,3,3-trimethyldihydroindole (1.59 g, 10.00 mmol) and 1-iononane (3.31 g, 13 mmol) were dissolved in acetonitrile (5.00 mL), and the reaction mixture was heated at 84 oC for 48 hours. After completion, the reaction mixture was cooled to room temperature. The solvent was removed under reduced pressure, and ethyl acetate was added. The precipitate was filtered, washed by ethyl acetate to afford W6 as a reddish brown solid (2.14 g, 51.77% yield). ^{1}H NMR (400 MHz, DMSO-d_6) δ 8.03 – 7.94 (m, 1H), 7.90 – 7.81 (m, 1H), 7.67 – 7.58 (m, 2H), 4.45 (t, J = 7.8 Hz, 2H), 2.85 (s, 3H), 1.88 – 1.78 (m, 2H), 1.54 (s, 6H), 1.47 – 1.37 (m, 2H), 1.36 – 1.19 (m, 10H), 0.85 (d, J = 6.8 Hz, 3H).

2-((*E*)-2-((*E*)-2-chloro-3-(2-((*E*)-3,3-dimethyl-1-nonylindolin-2-ylidene)ethylidene)cyclohex-1-en-1-yl)vinyl)-3,3-dimethyl-1-nonyl-3*H*-indol-1-ium iodide (IR-6B9)：

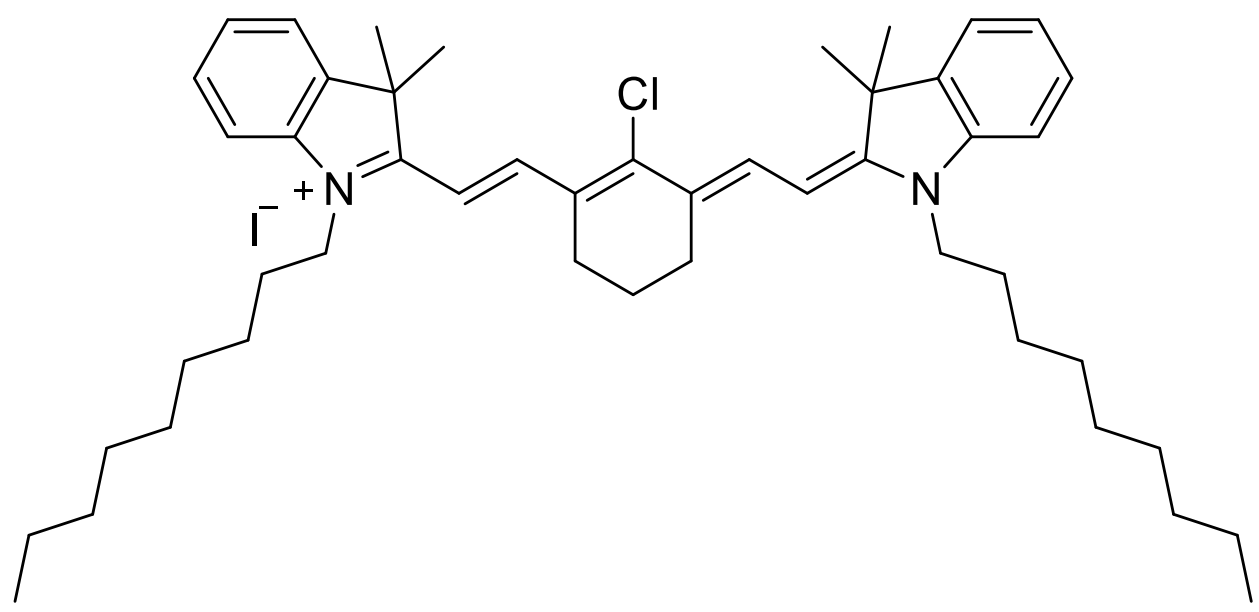

IR-6B9

Compound **S7** (34.40 mg, 0.20 mmol), compound **W6** (165.28 mg, 0.40 mmol) were dissolved in ethanol (4.00 mL), and then sodium acetate (32.82 mg, 0.40 mmol) was added into the solution.

The reaction mixture was heated at 50 °C for 8 hours. After completion, the reaction mixture was cooled to room temperature. The solvent was removed under reduced pressure, and the residue was purified by chromatography on silica gel eluting with (dichloromethane: methanol = 50: 1, *v/v*) to afford **IR-6B9** as a green solid (50.82mg, 30.42% yield). ^{1}H NMR (400 MHz, $CDCl_3$) δ 8.34 (d, *J* = 14.1 Hz, 2H), 7.44 – 7.36 (m, 4H), 7.29 – 7.22 (m, 2H), 7.16 (d, *J* = 8.0 Hz, 2H), 6.24 (d, *J* = 14.1 Hz, 2H), 4.20 (t, *J* = 7.4 Hz, 4H), 2.82 – 2.70 (m, 4H), 2.05 – 1.95 (m, 2H), 1.91 – 1.80 (m, 4H), 1.73 (s, 12H), 1.51 – 1.42 (m, 4H), 1.41 – 1.34 (m, 4H), 1.31 – 1.23 (m, 16H), 0.91 – 0.82 (m, 6H). ^{13}C NMR (100 MHz, $CDCl_3$) δ 172.4, 150.4, 144.2, 142.3, 141.1, 128.8, 127.3, 125.3, 122.3, 110.9, 101.4, 49.4, 45.1, 31.8, 29.37, 29.33, 29.2, 28.2, 27.4, 27.0, 26.7, 22.6, 20.7, 14.1. HRMS (ESI-TOF) m/z: calcd. for $[M]^+$ $C_{48}H_{68}ClN_2^+$ = 707.5066; found 707.5055.

Synthesis route of CETIF6a-GSH：

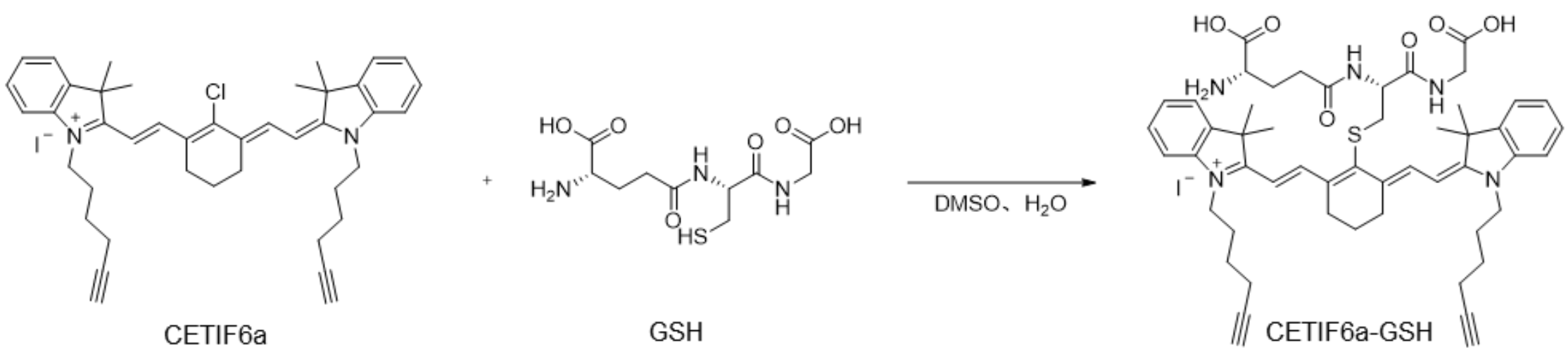

CETIF6a (50 mg, 0.065 mmol) was dissolved in a 10 mL round-bottom flask containing 2 mL dimethyl sulfoxide. Then, GSH (62 mg, 0.20 mmol) was dissolved in 0.55 mL water. The resulting aqueous solution was added dropwise to the above solution and stirred at room temperature for 2 hours. The reaction mixture was separated by reversed-phase preparative liquid chromatography (water: acetonitrile = 70: 30, v/v), and the green powder of 17 mg was collected, with a yield of 24.92%. ^{1}H NMR (400 MHz, CD_3OD) δ 8.80 (d, *J* = 14 Hz, 2H), 7.55 (d, *J* = 7.6 Hz, 2H), 7.45 (t, *J* = 8.0 Hz, 2H), 7.36 – 7.28 (m, 4H), 6.33 (d, *J* = 14.0 Hz, 2H), 4.61 (d, *J* = 5.2 Hz, 1H), 4.21 (t, *J* = 7.2 Hz, 4H), 3.90 (t, *J* = 7.2 Hz, 3H), 3.42(dd, *J* = 13.6, 5.2 Hz, 1H), 3.15 (dd, *J* = 13.6, 8.4 Hz, 1H), 2.72 (s, 4H), 2.62 – 2.46 (m, 2H), 2.35 – 2.32 (m, 6H), 2.16 – 2.13 (m, 2H), 2.03-1.96 (m, 6H), 1.78 (d, *J* = 4.8 Hz, 11H), 1.78 – 1.67 (m, 4H).

^{1}H-NMR of S1

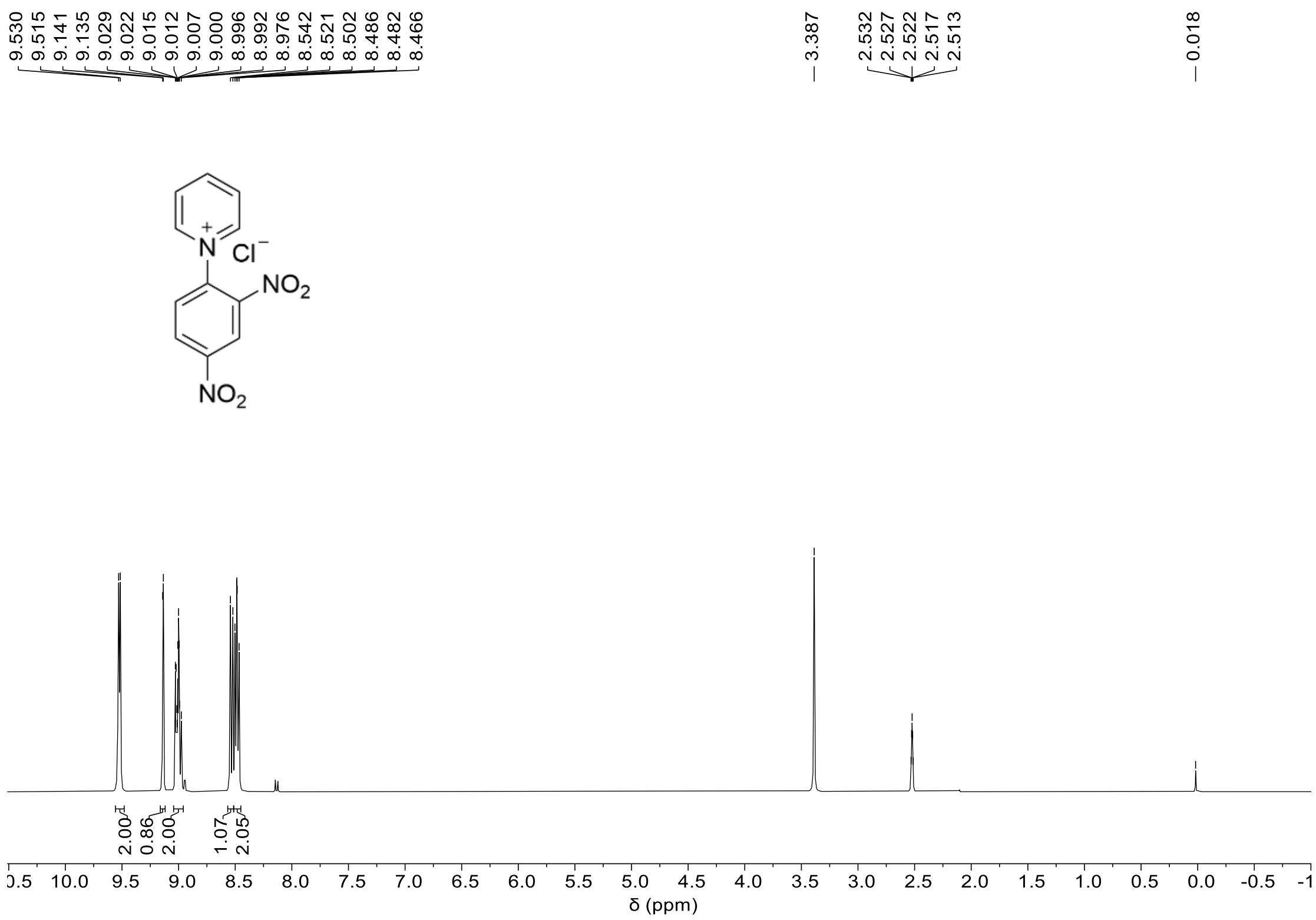

^{1}H-NMR of W1

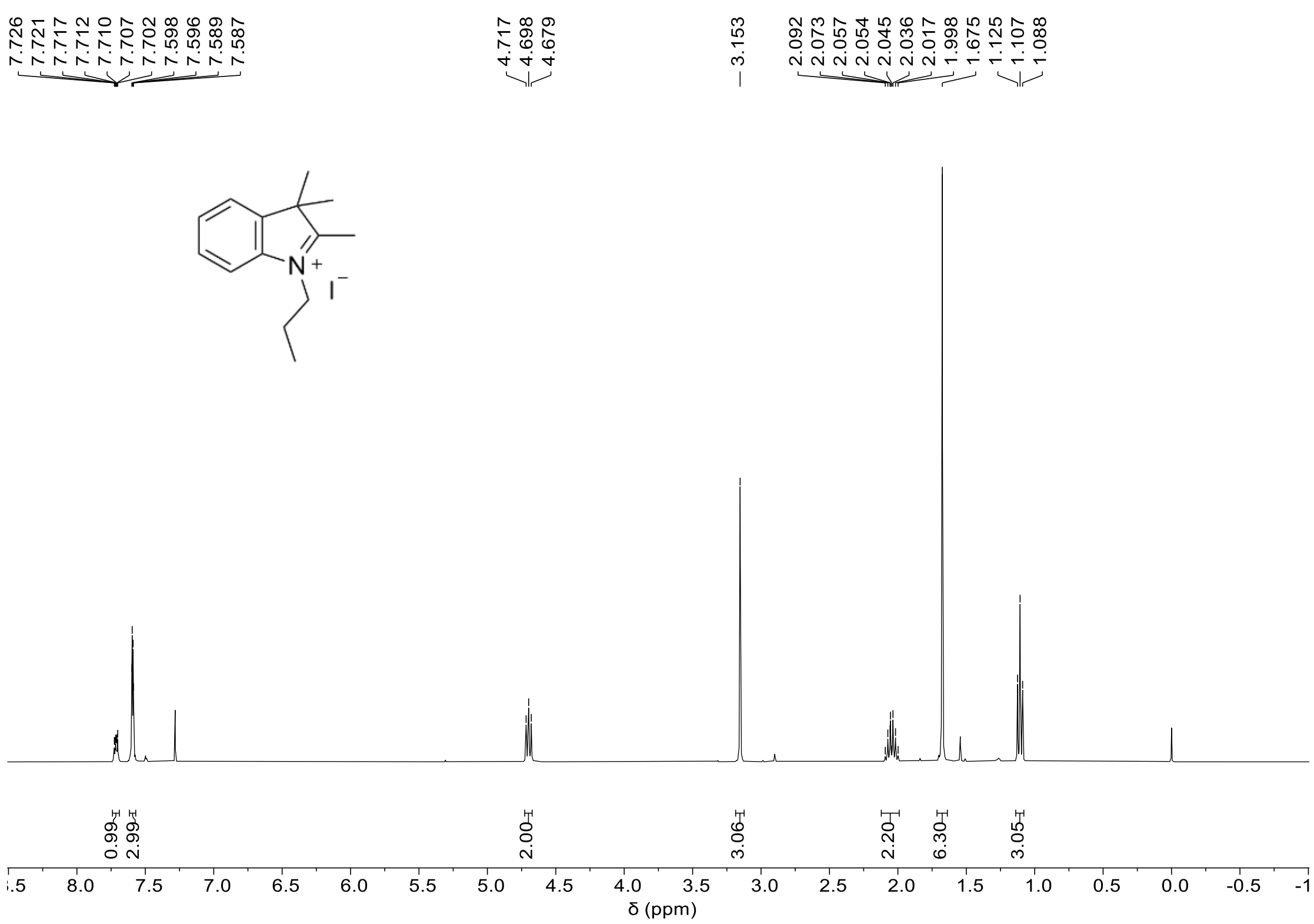

^{1}H-NMR of IR-1B3

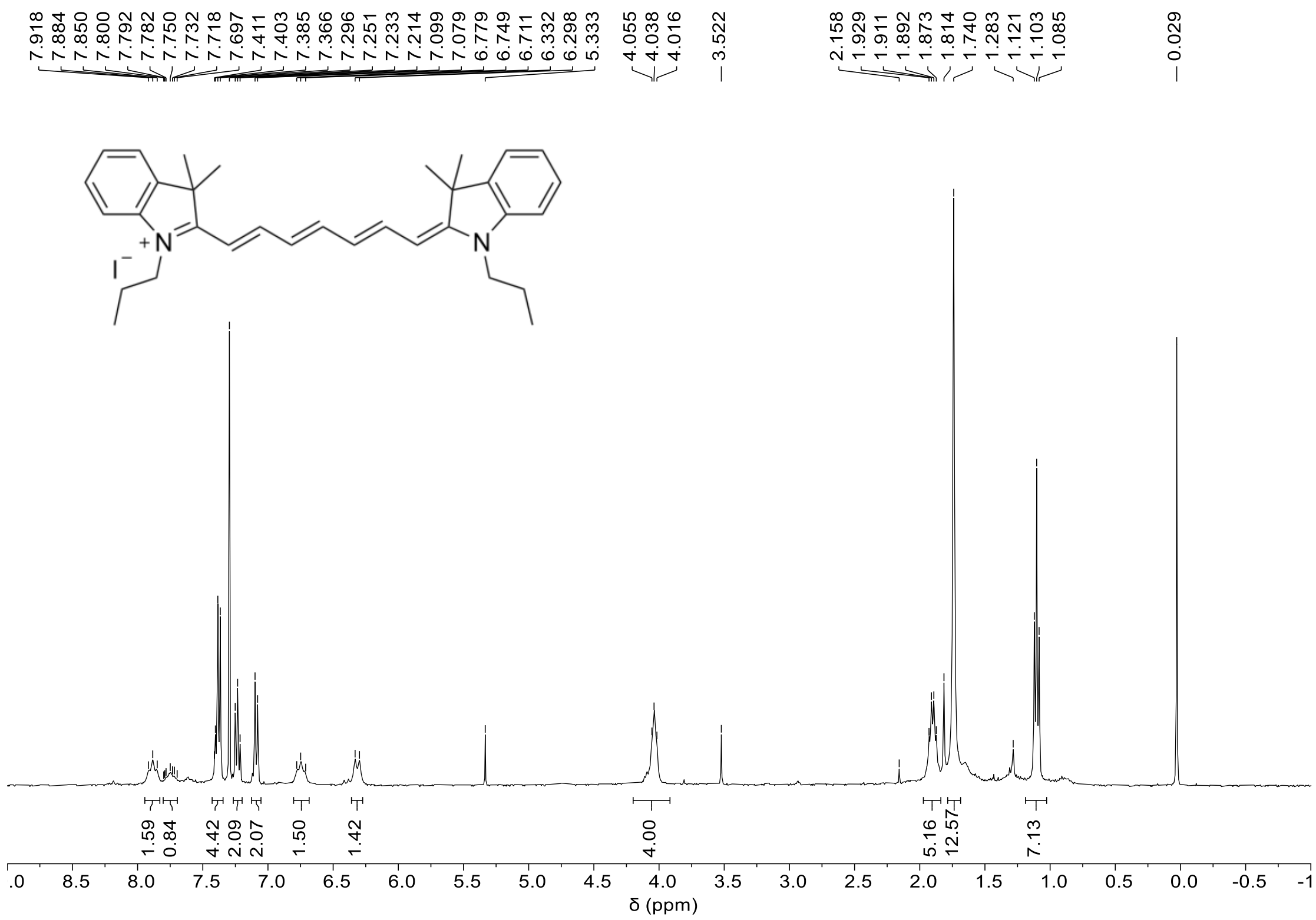

^{13}C-NMR of IR-1B3

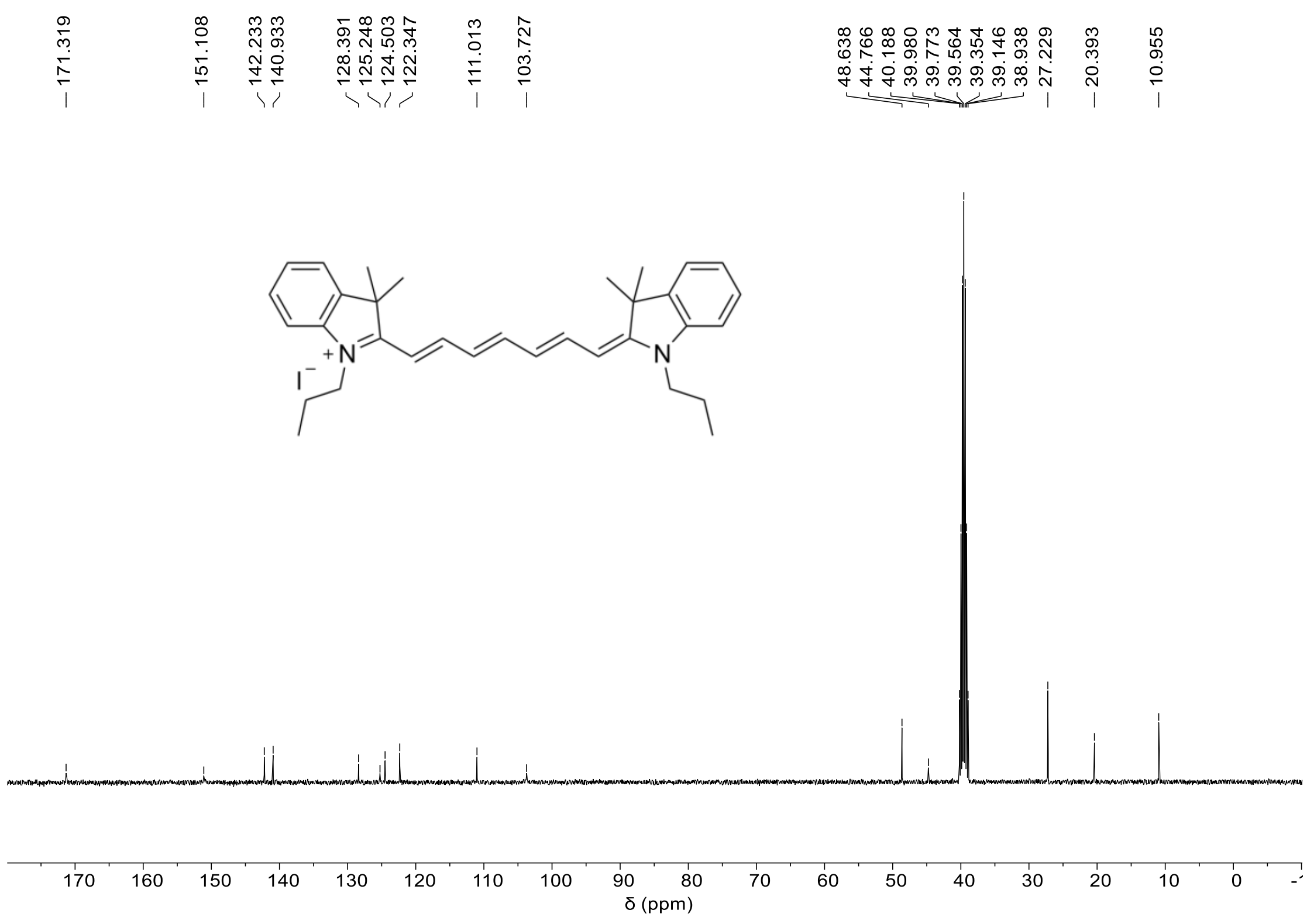

HRMS of IR-1B3

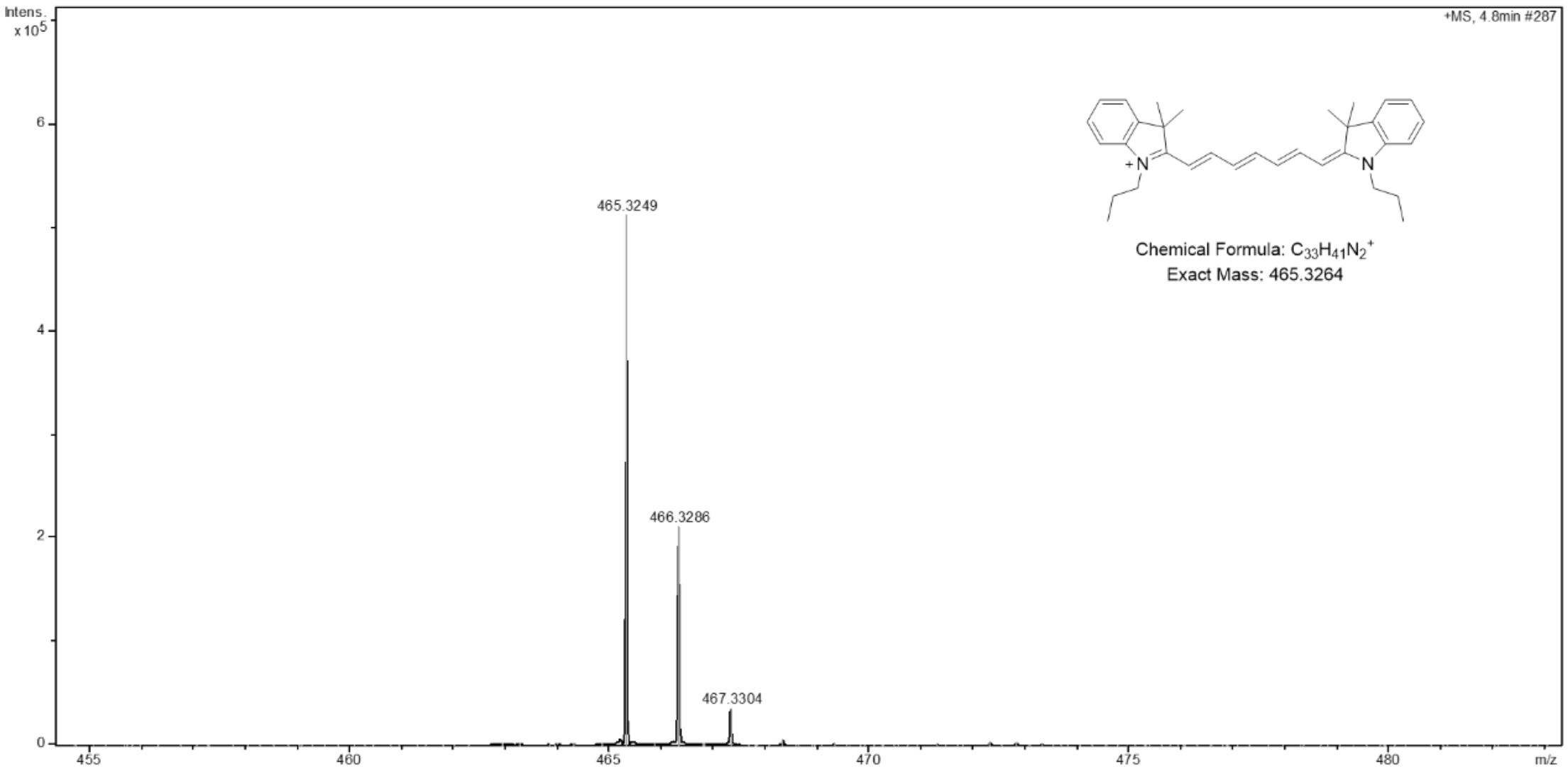

^{1}H-NMR of S2

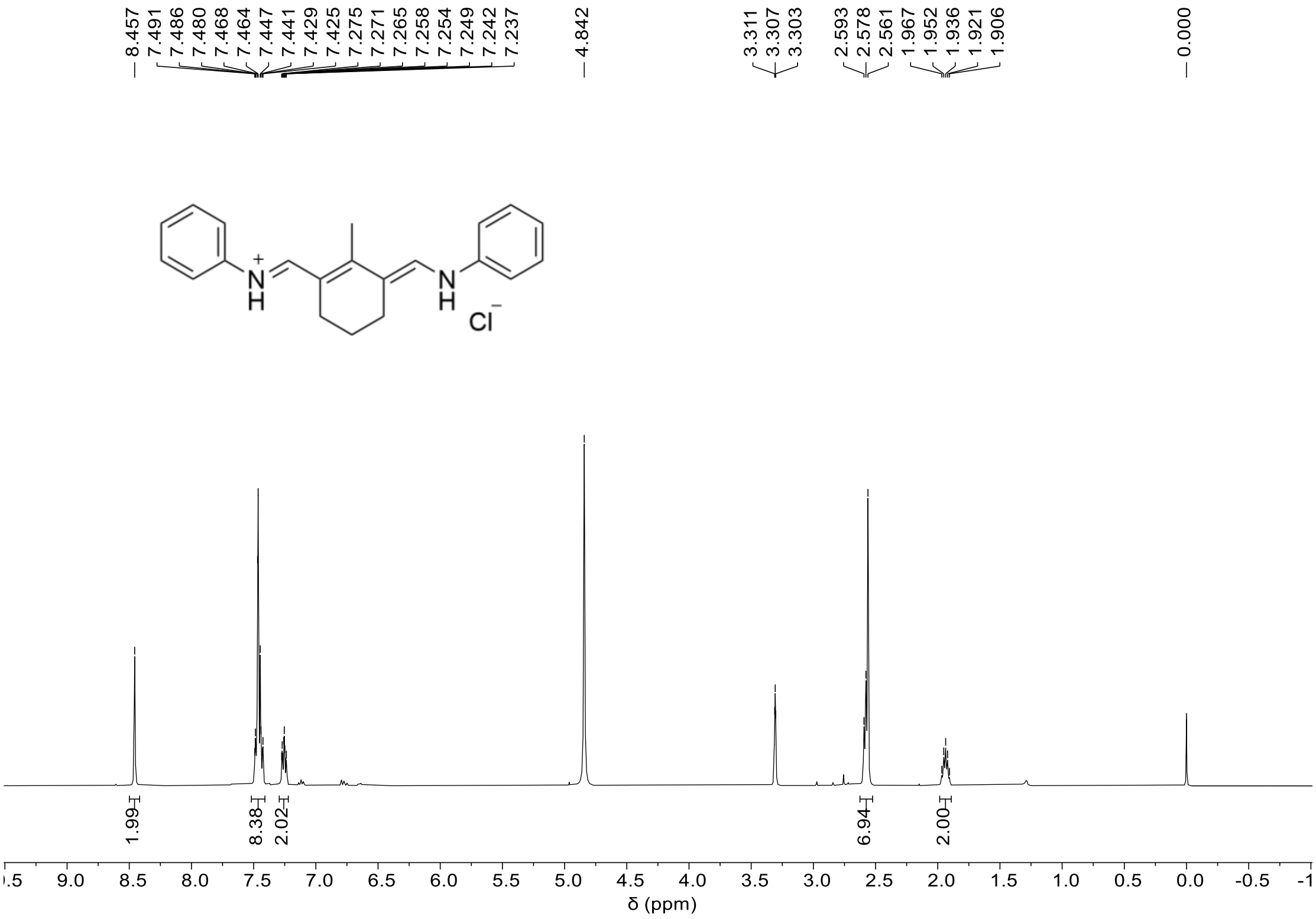

^{1}H-NMR of IR-2B3

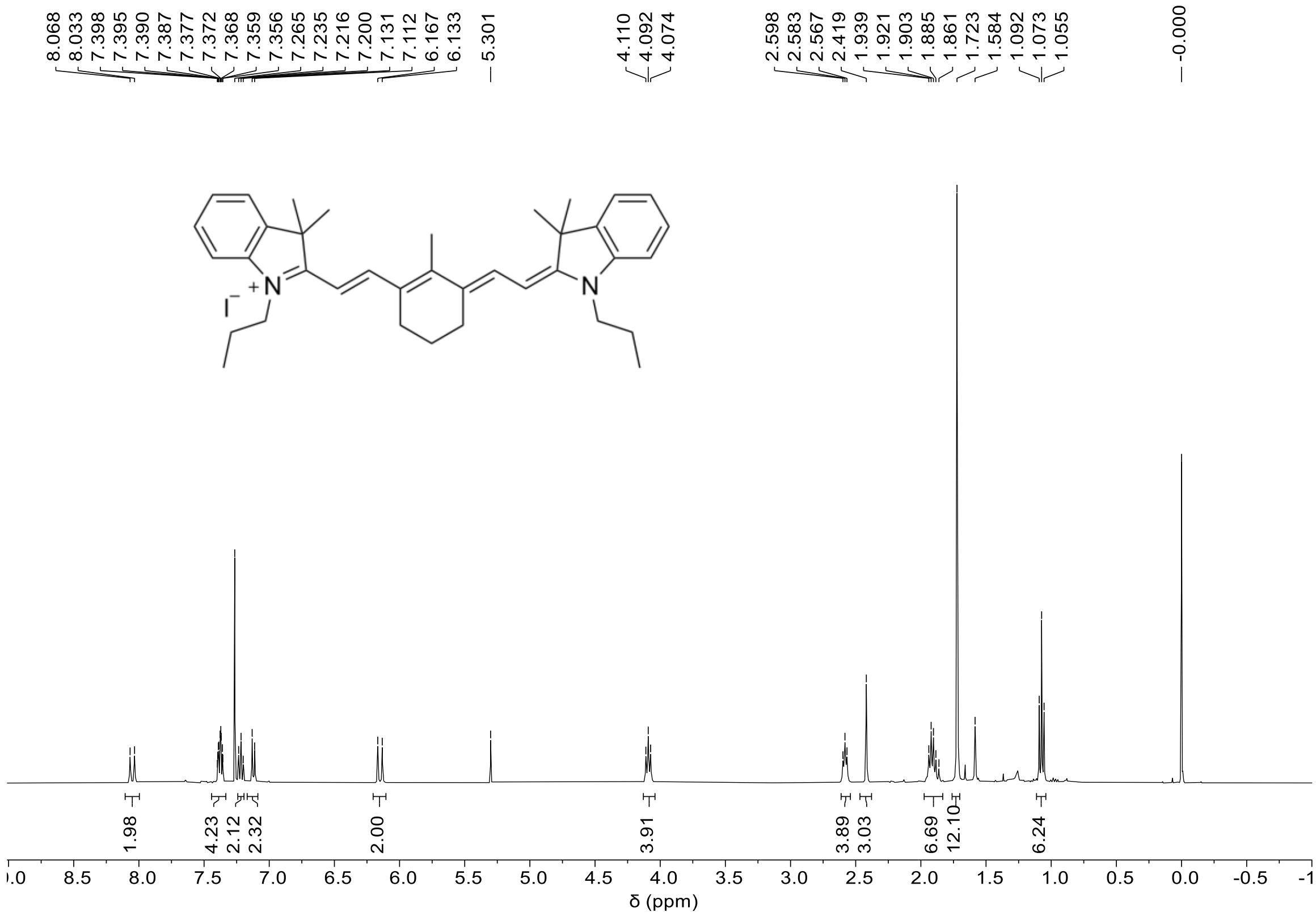

^{13}C-NMR of IR-2B3

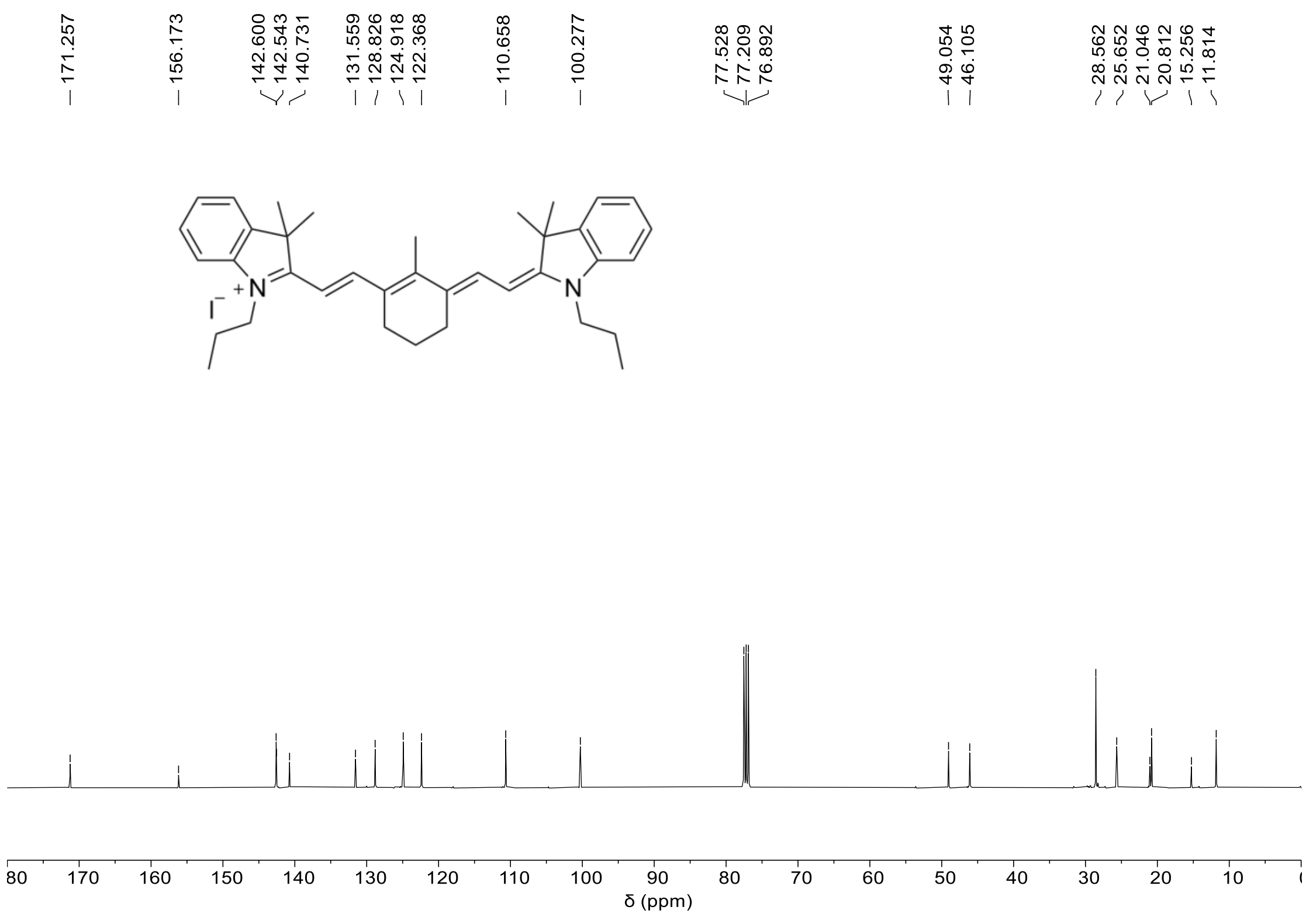

HRMS of IR-2B3

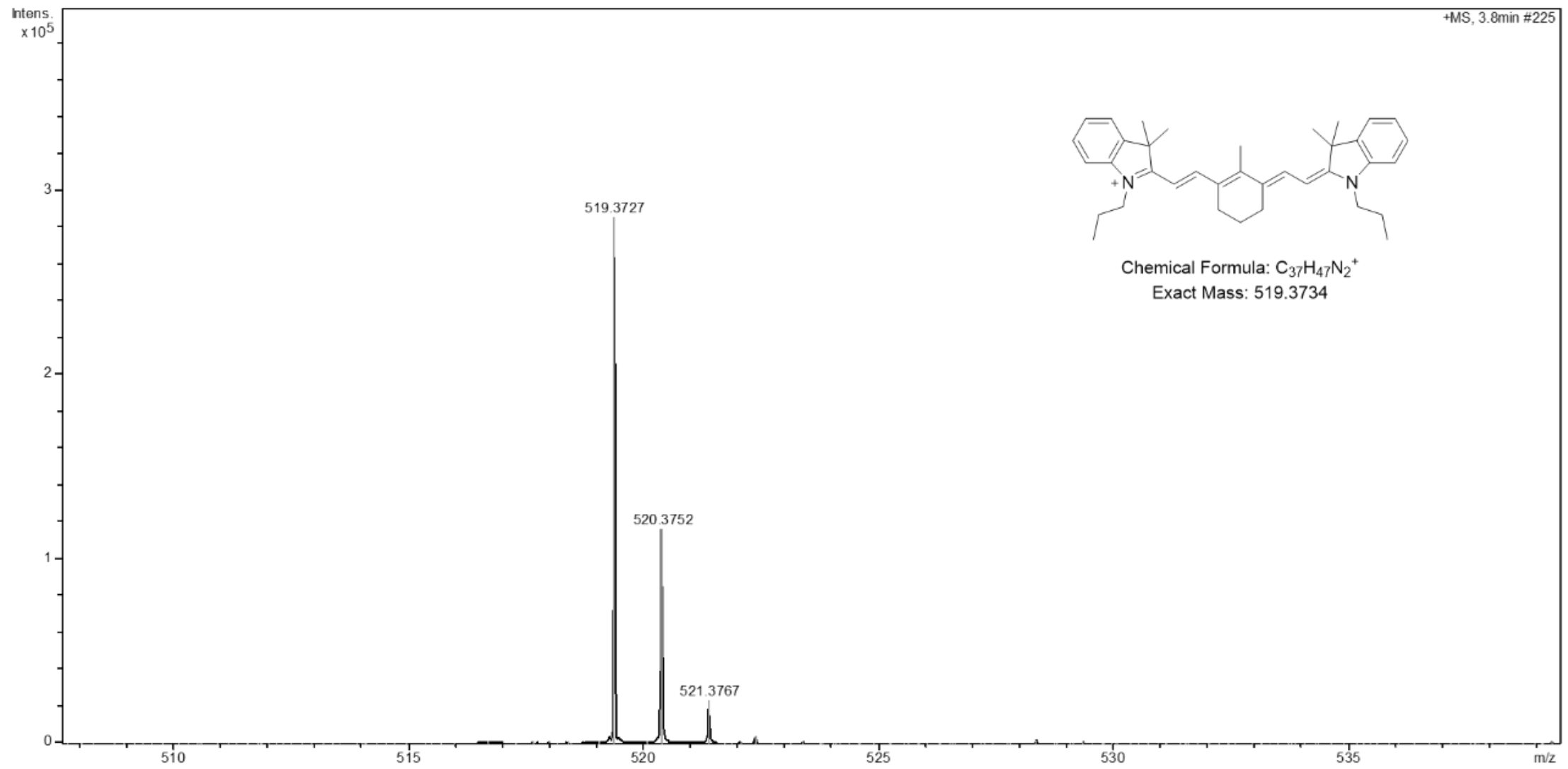

^{1}H-NMR of S3

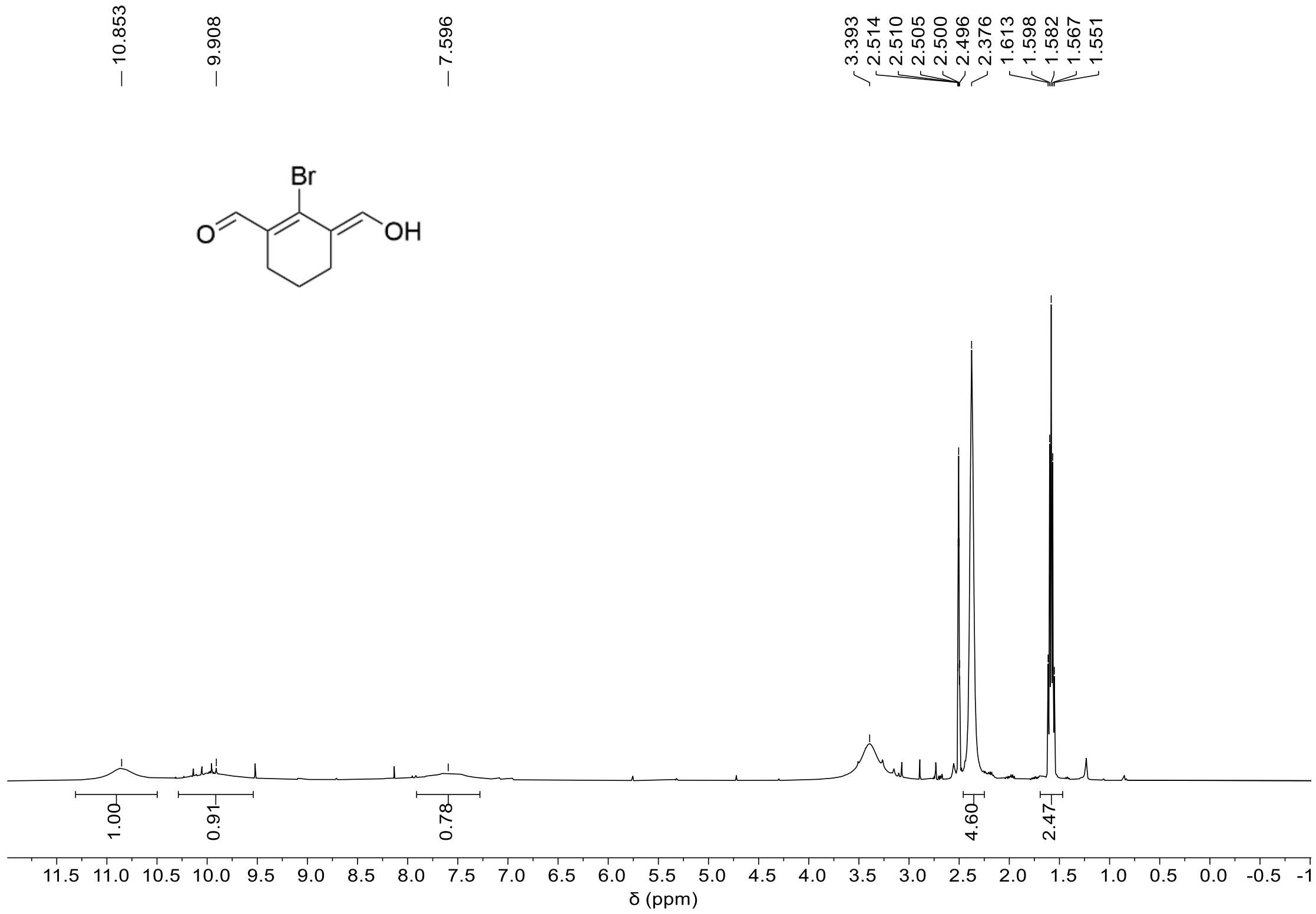

^{1}H-NMR of IR-3B3

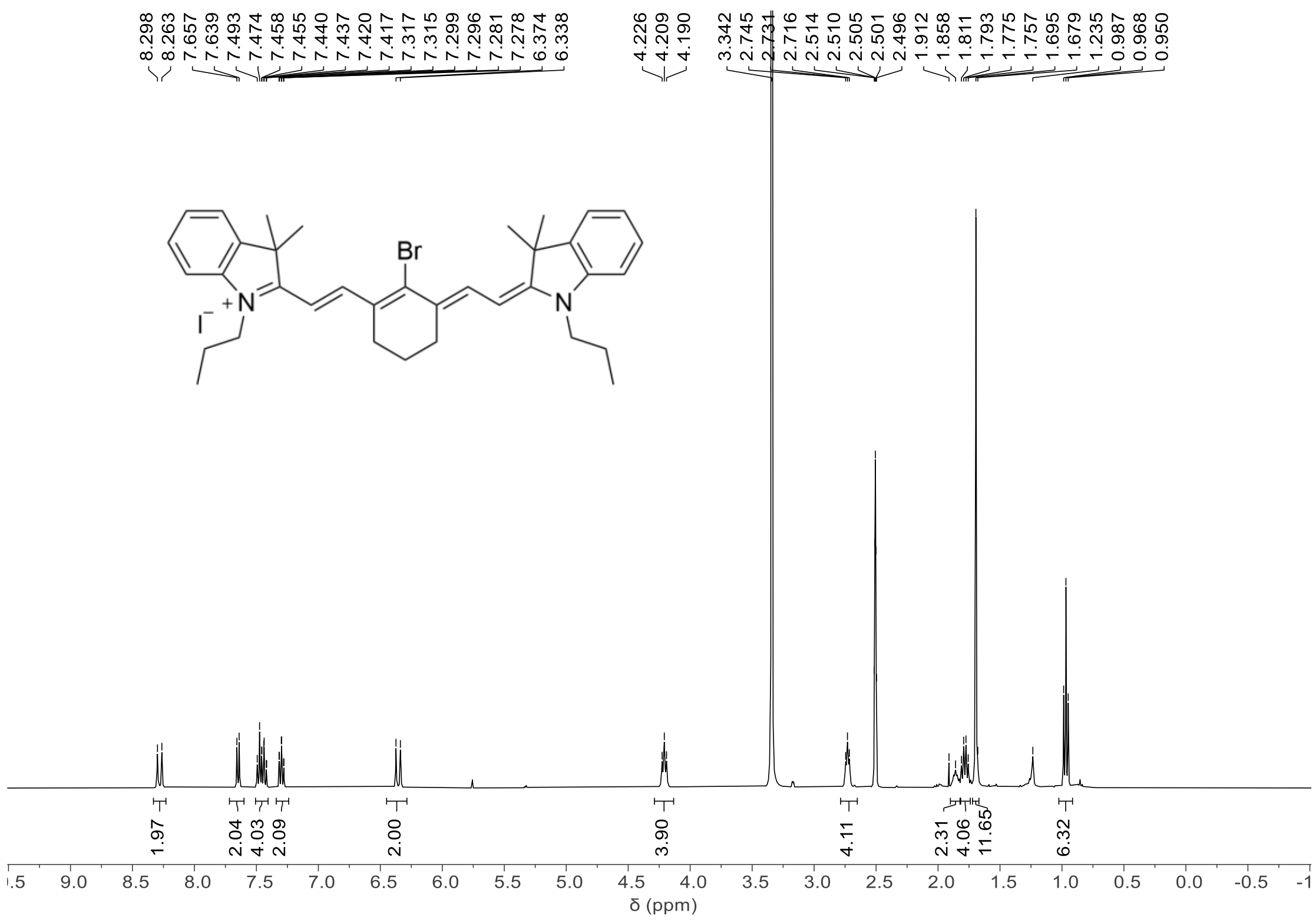

^{13}C-NMR of IR-3B3

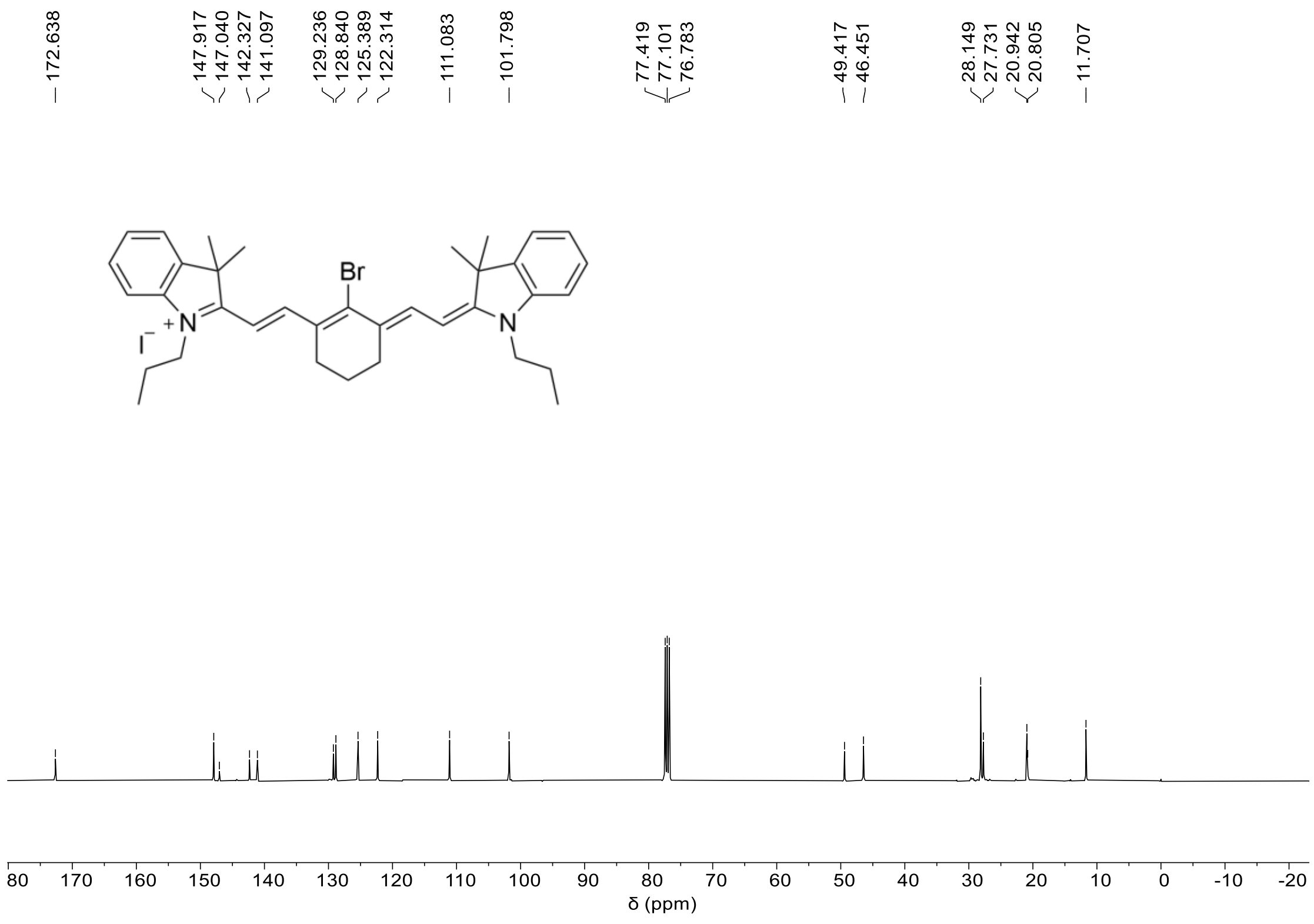

HRMS of IR-3B3

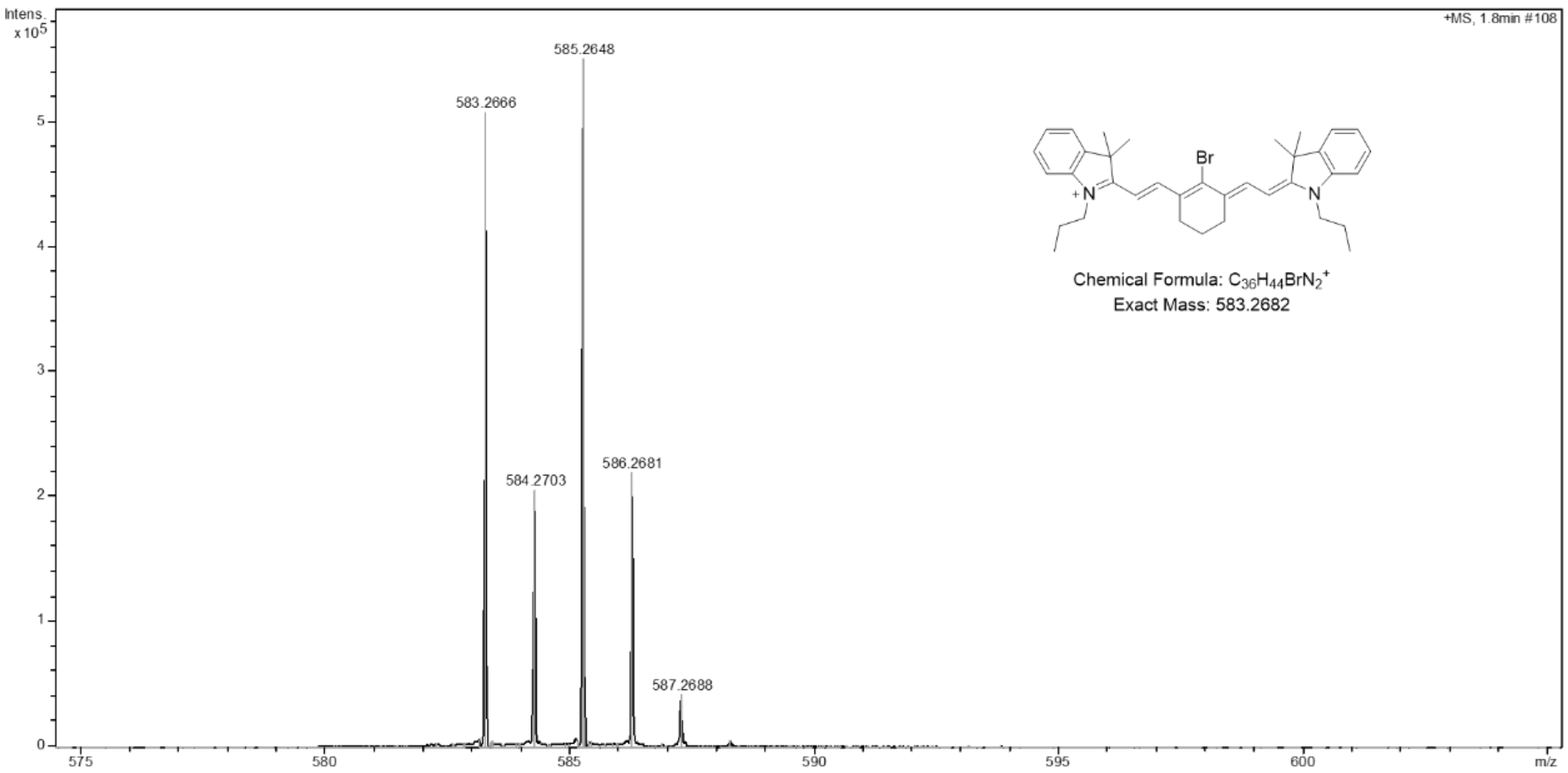

^{1}H-NMR of S4

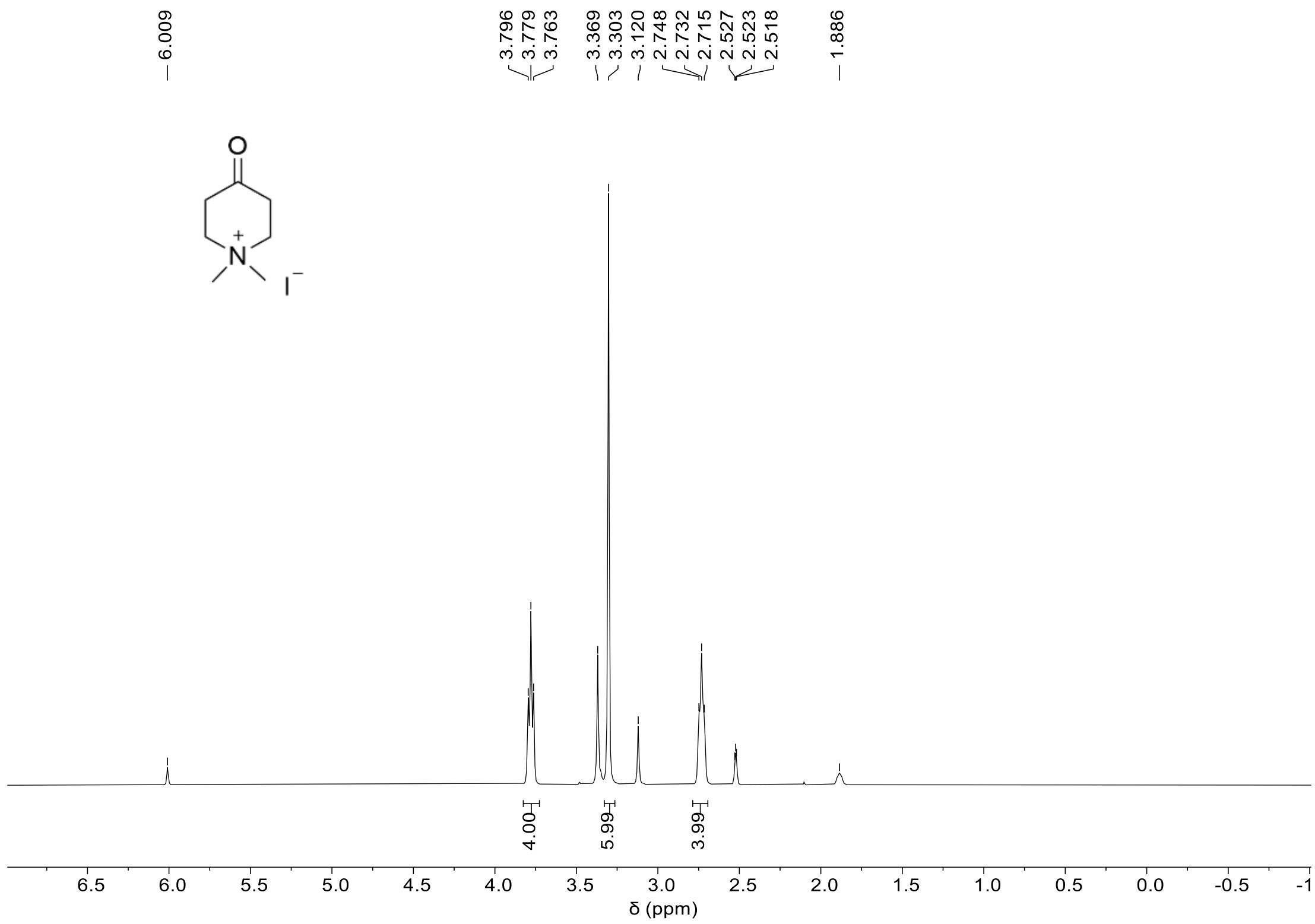

^{1}H-NMR of S5

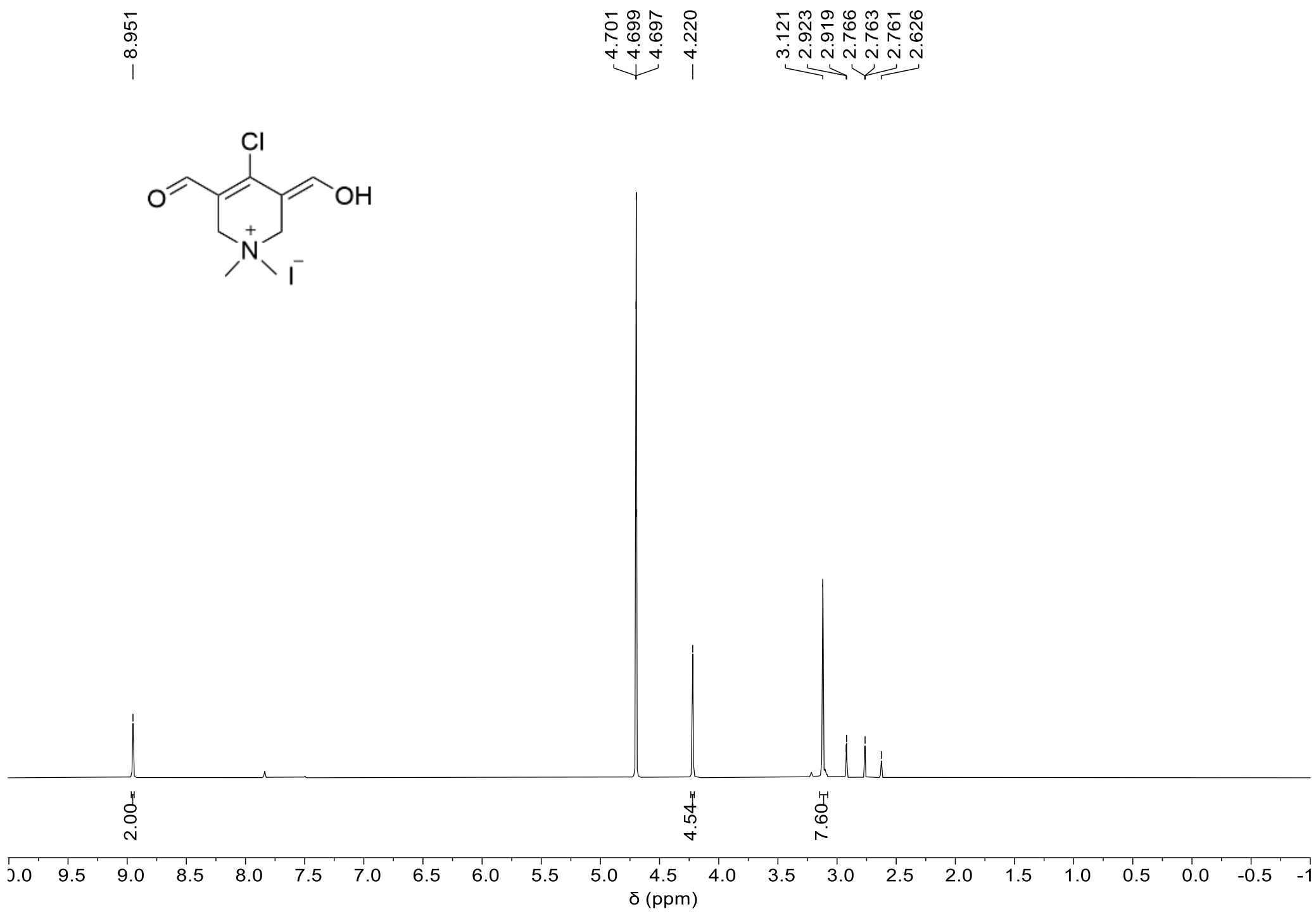

^{1}H-NMR of IR-4B3

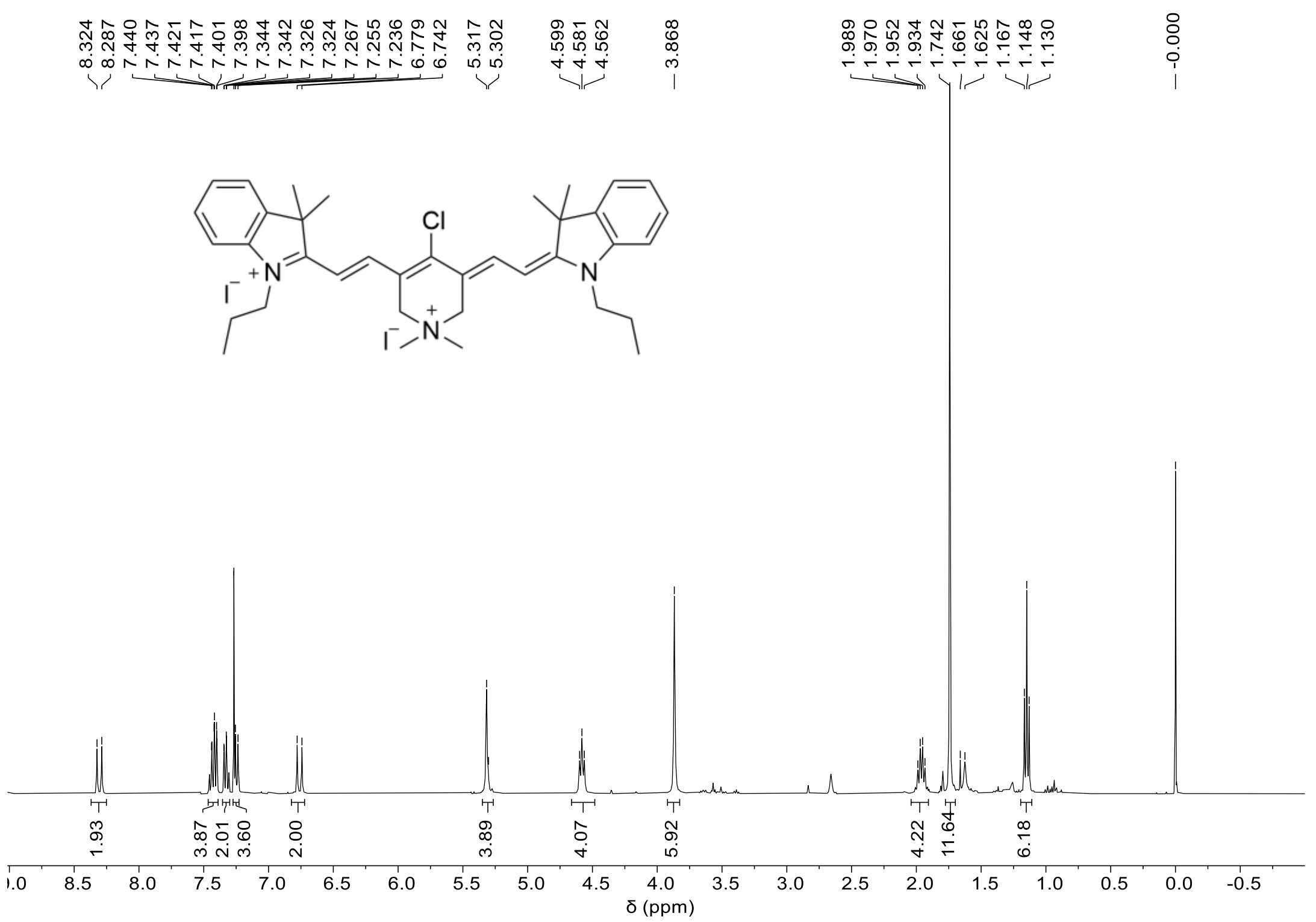

^{13}C-NMR of IR-4B3

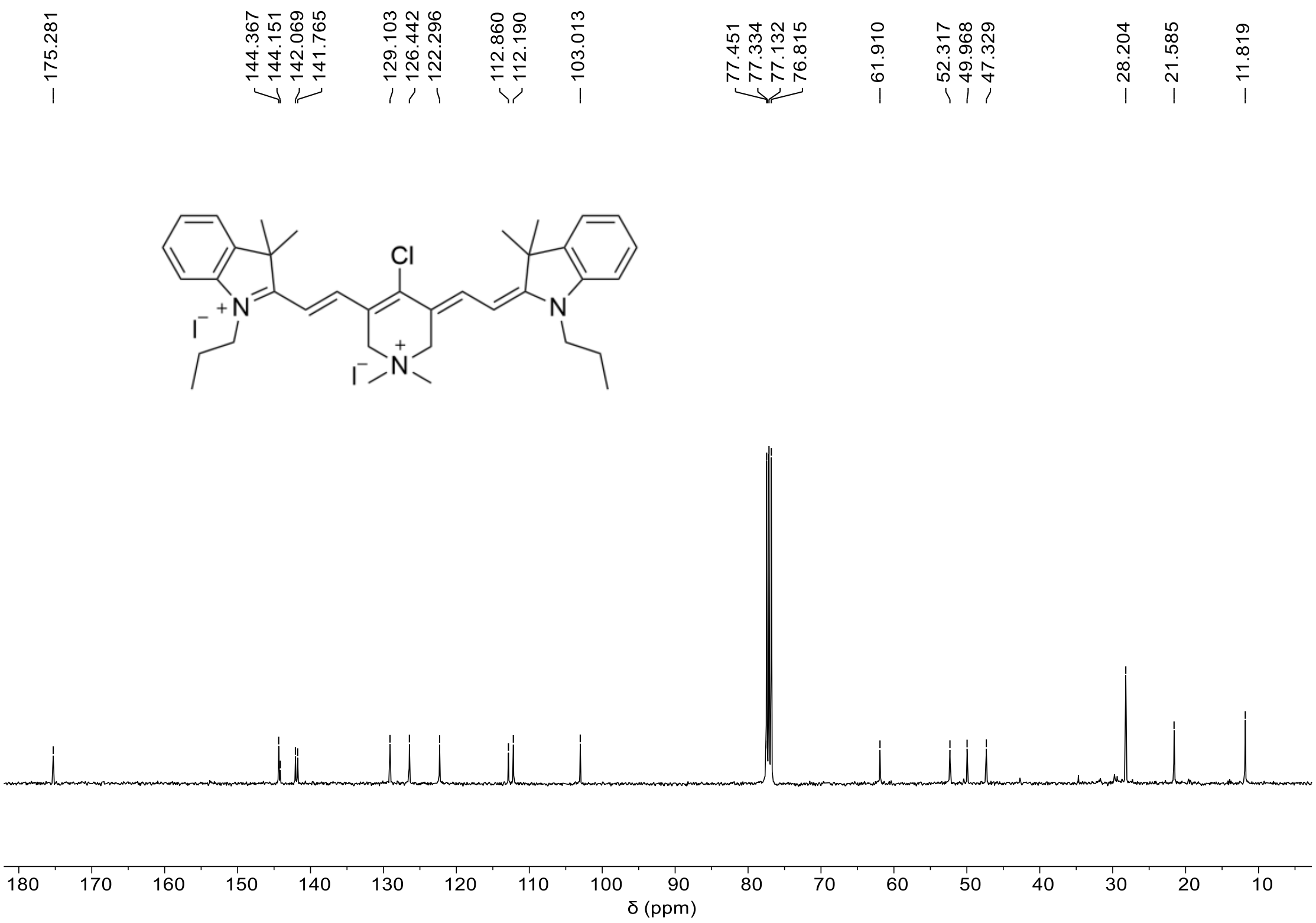

HRMS of IR-4B3

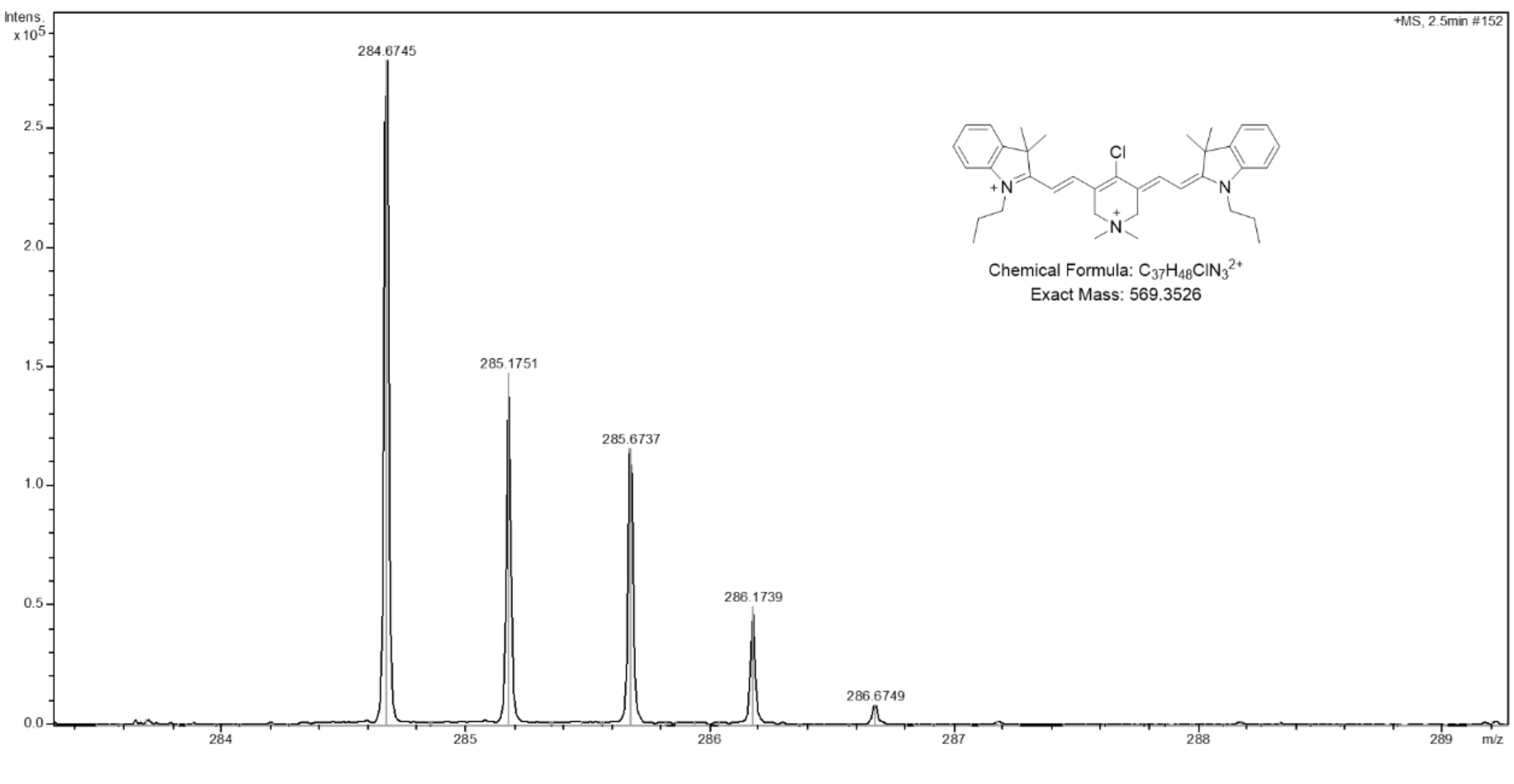

^{1}H-NMR of S6

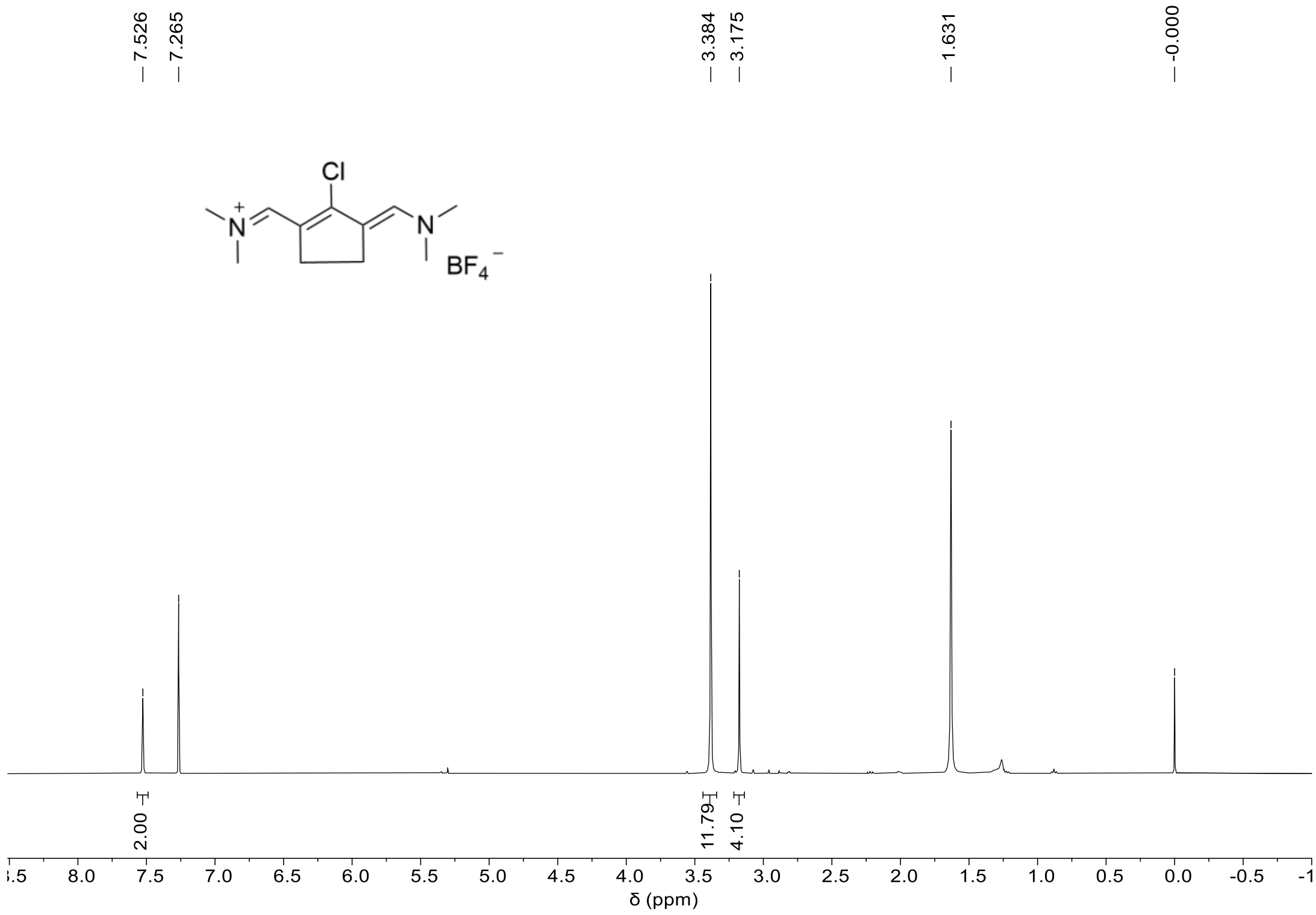

^{1}H-NMR of IR-5B3

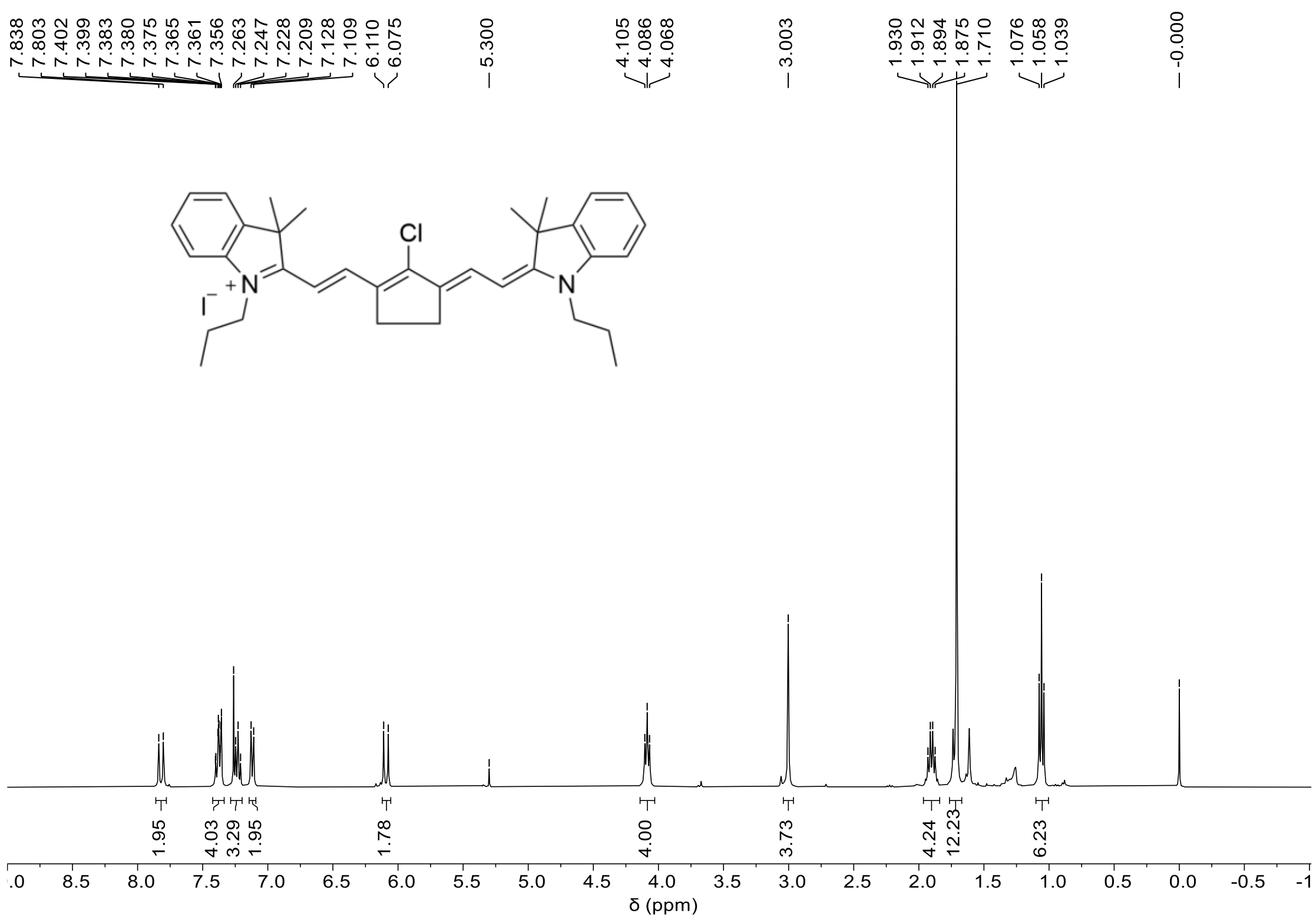

^{13}C-NMR of IR-5B3

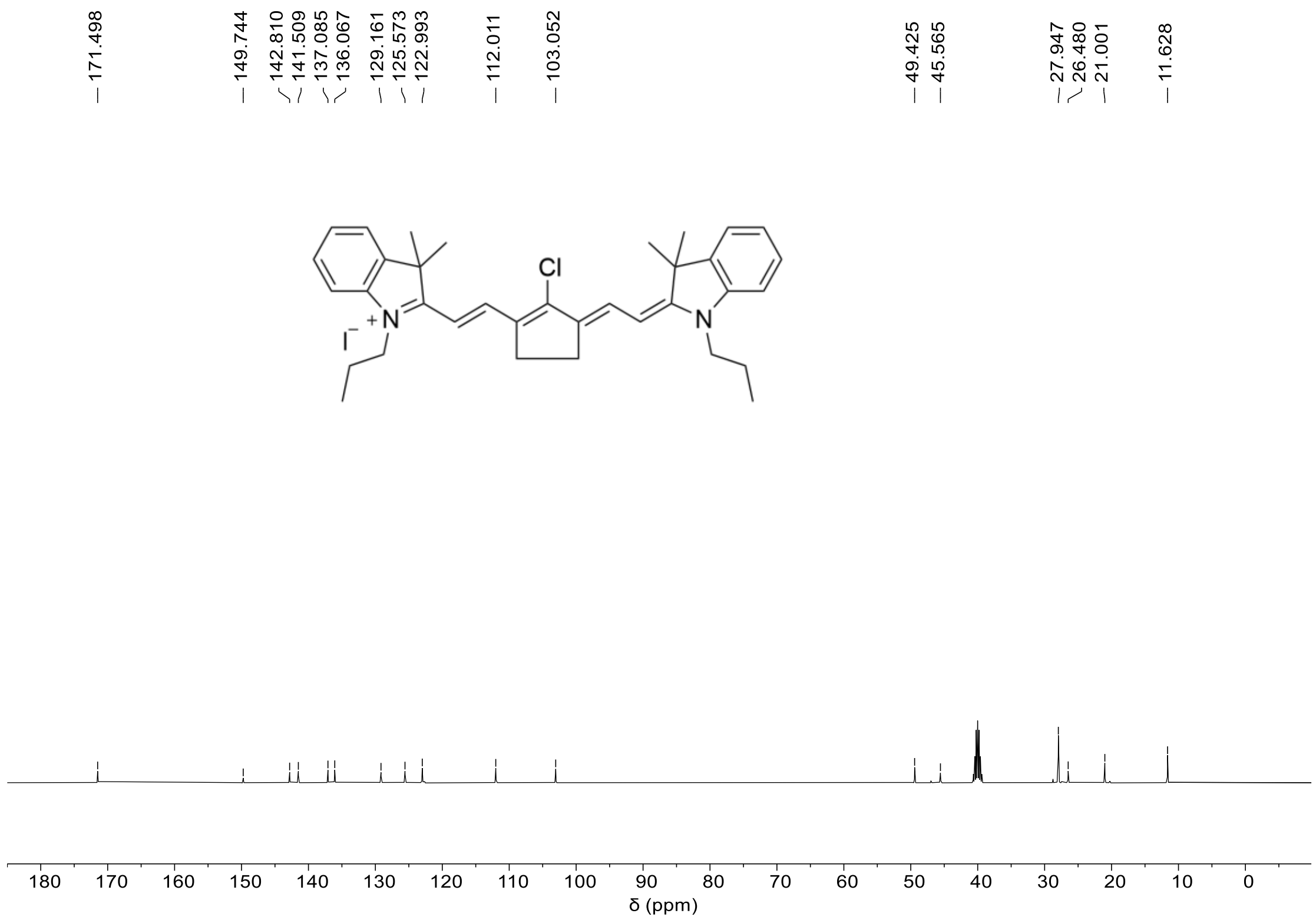

HRMS of IR-5B3

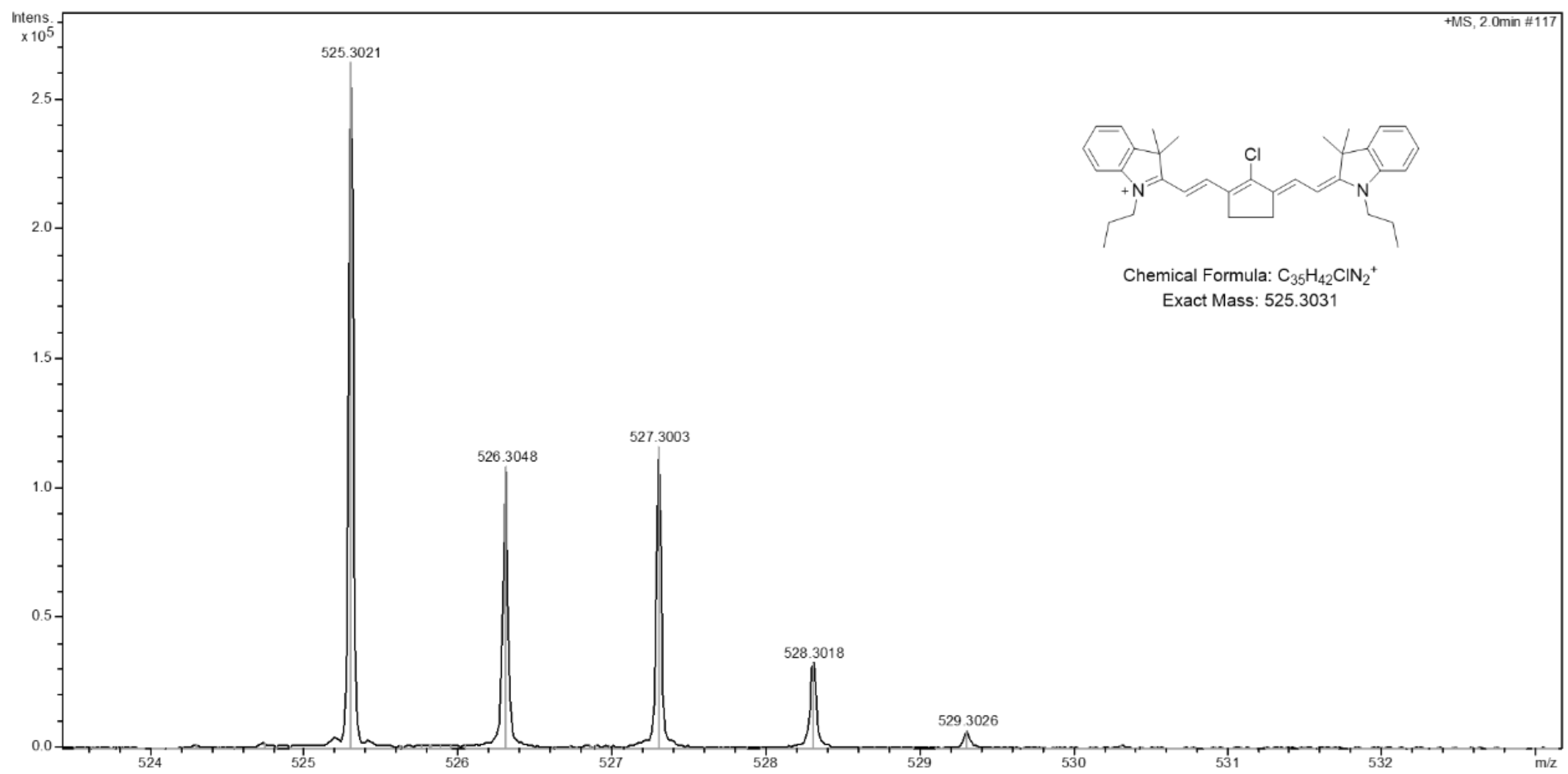

^{1}H-NMR of S7

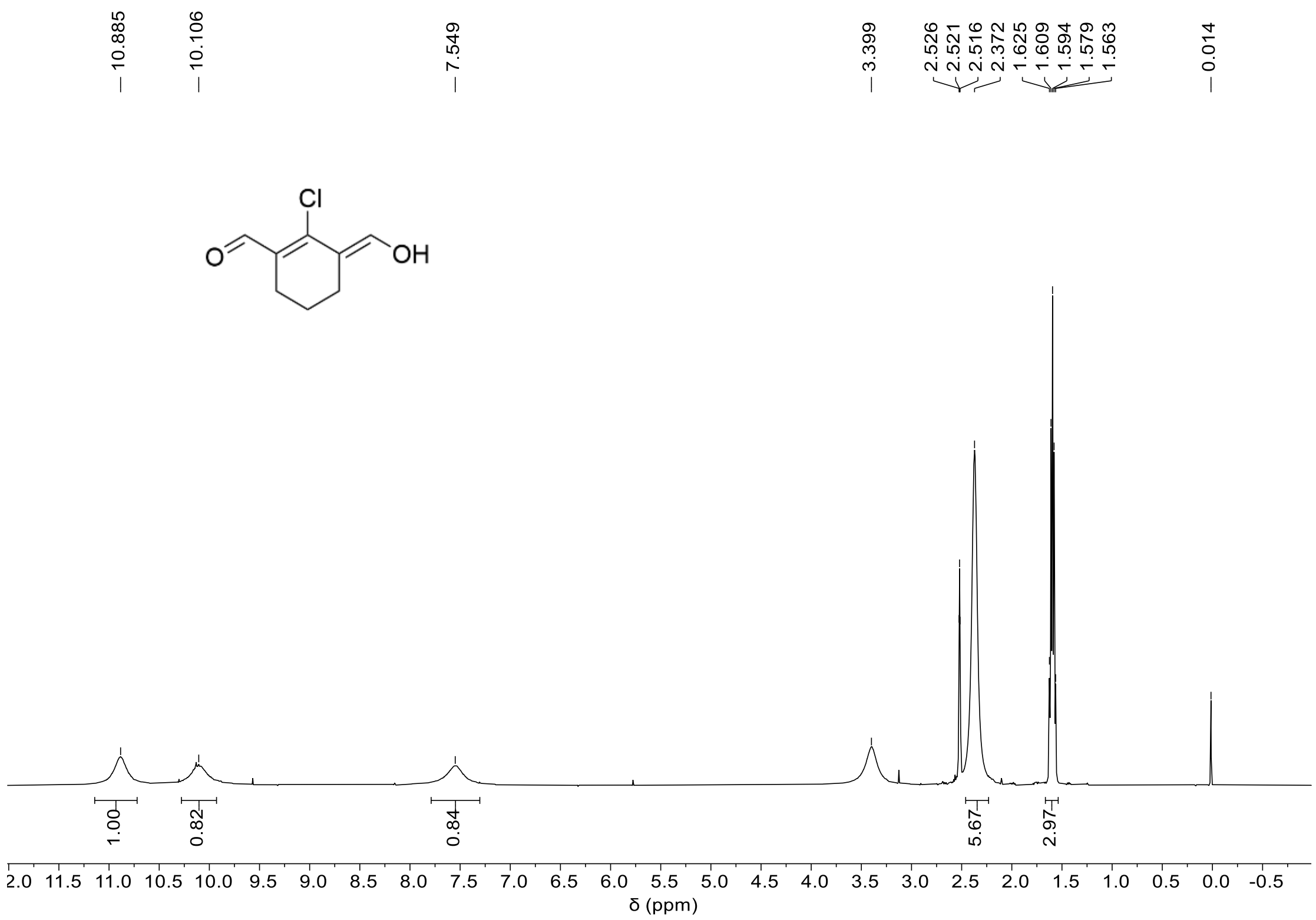

^{1}H-NMR of W2

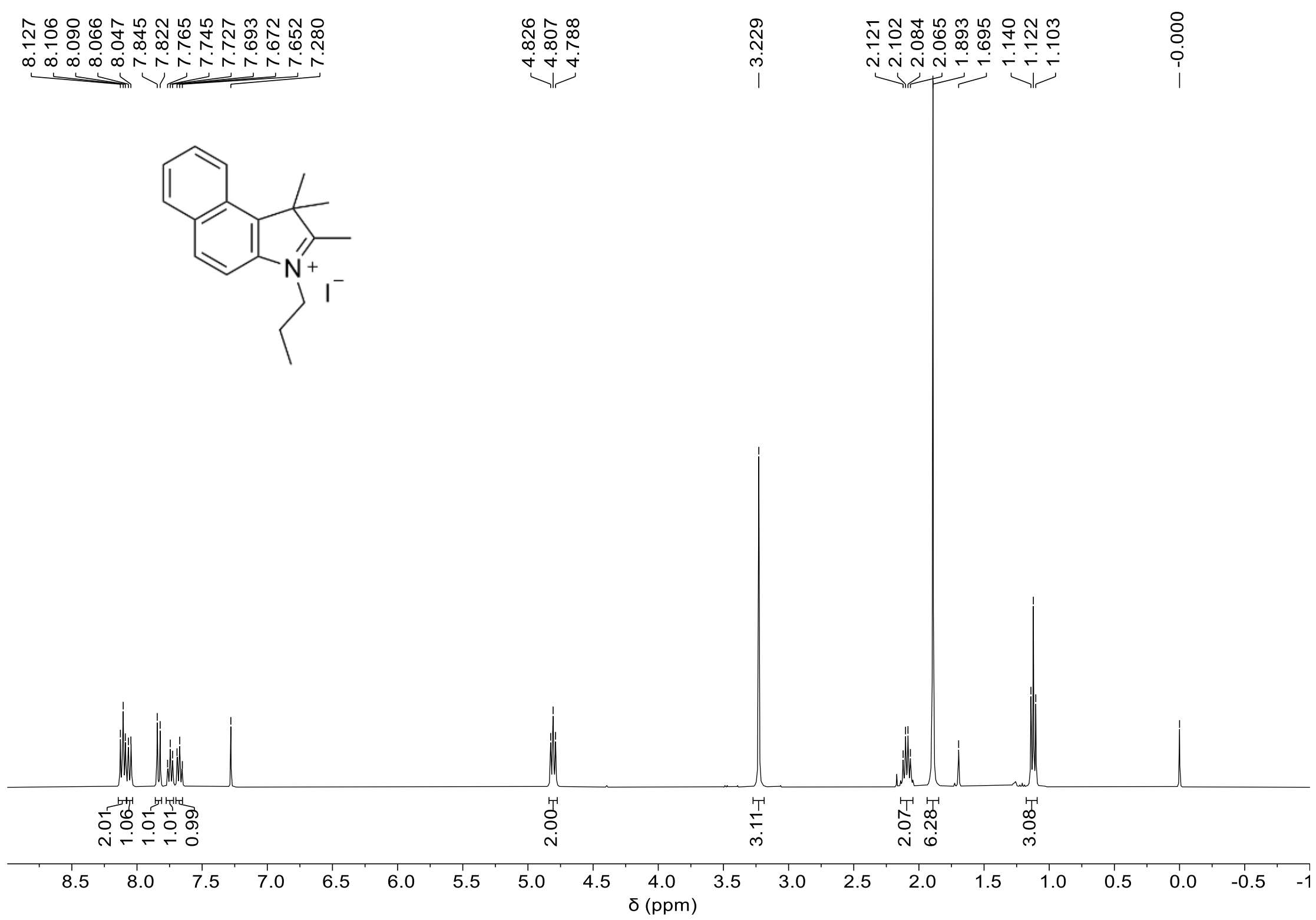

^{1}H-NMR of IR-6N3

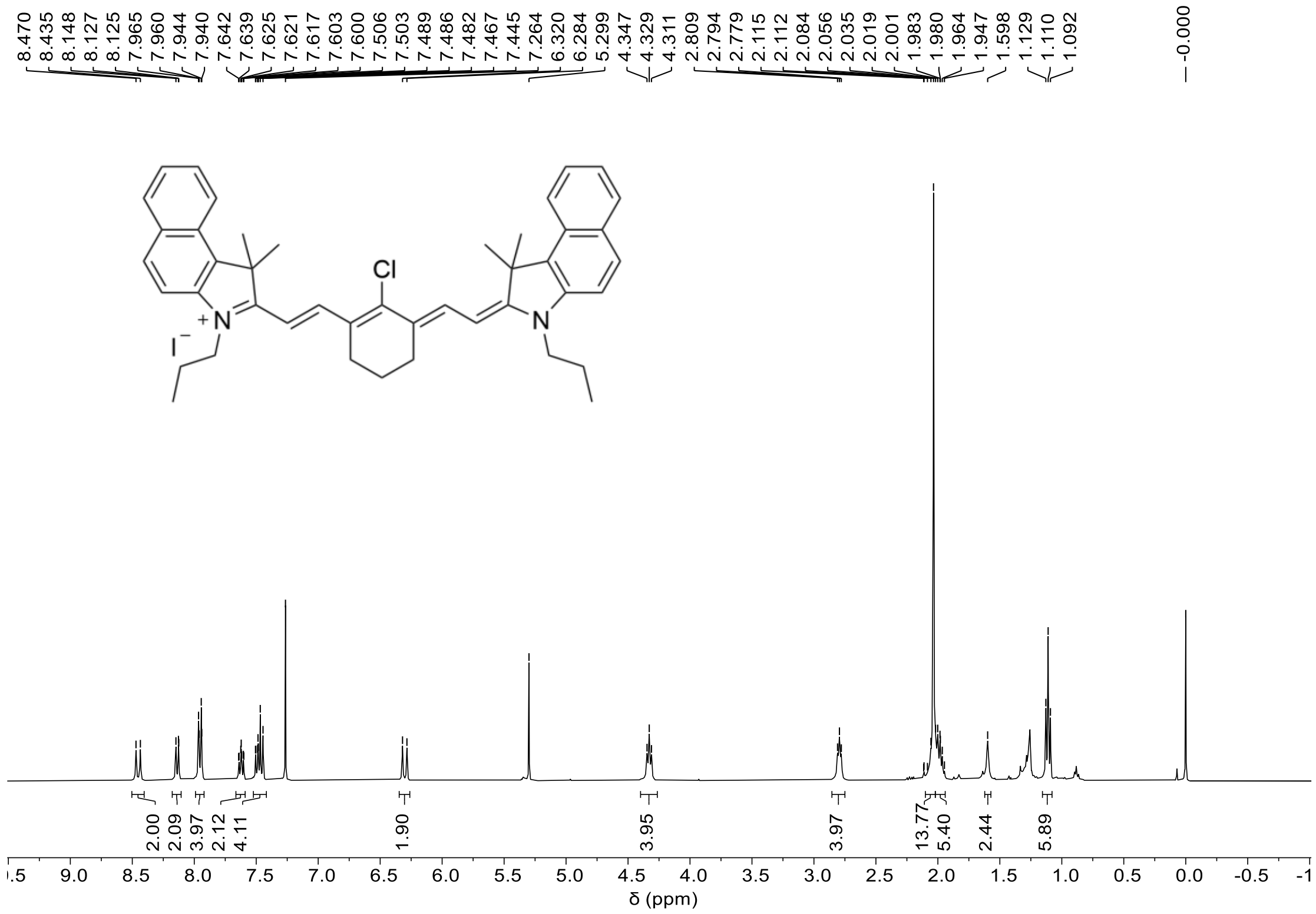

^{13}C-NMR of IR-6N3

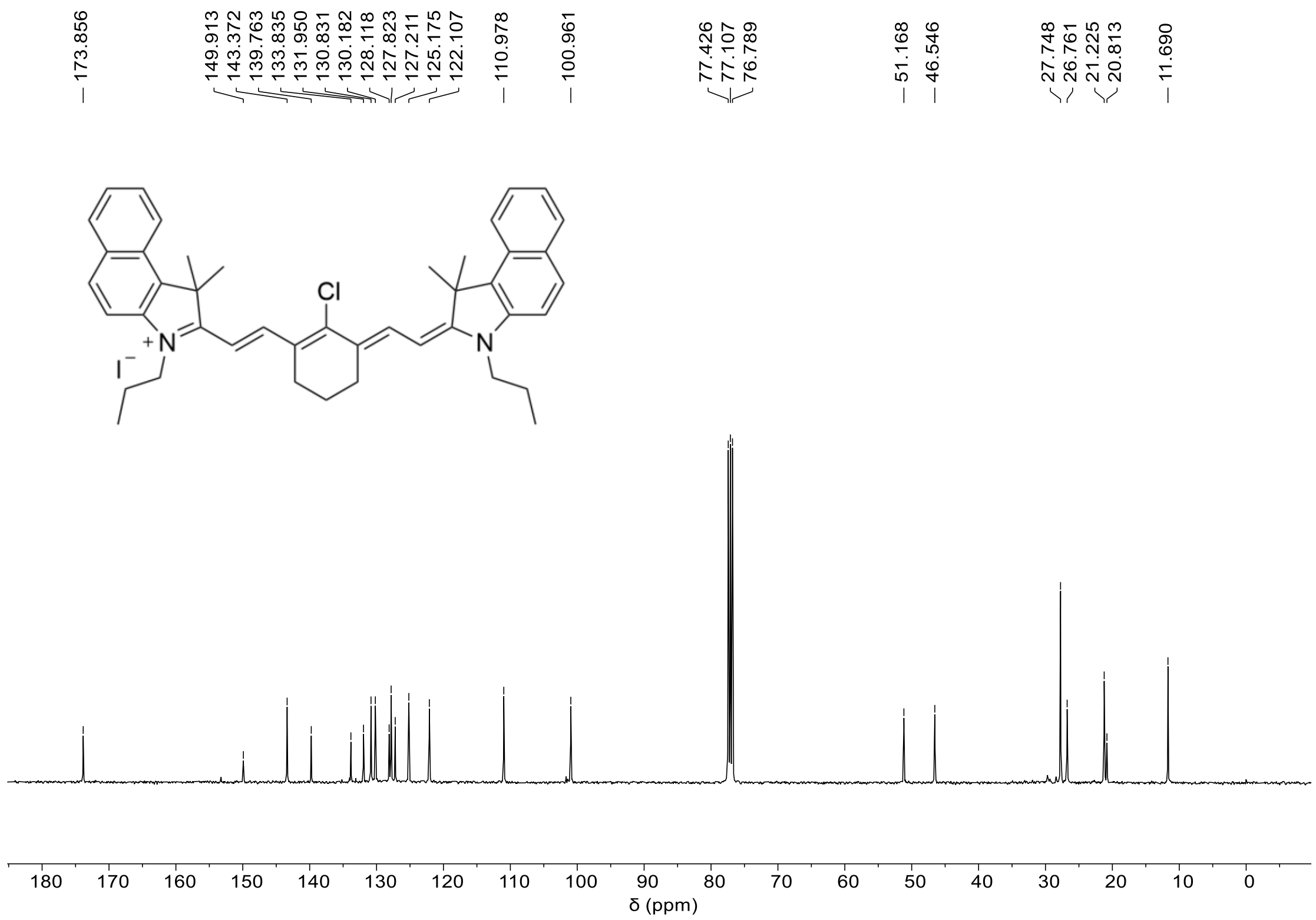

HRMS of IR-6N3

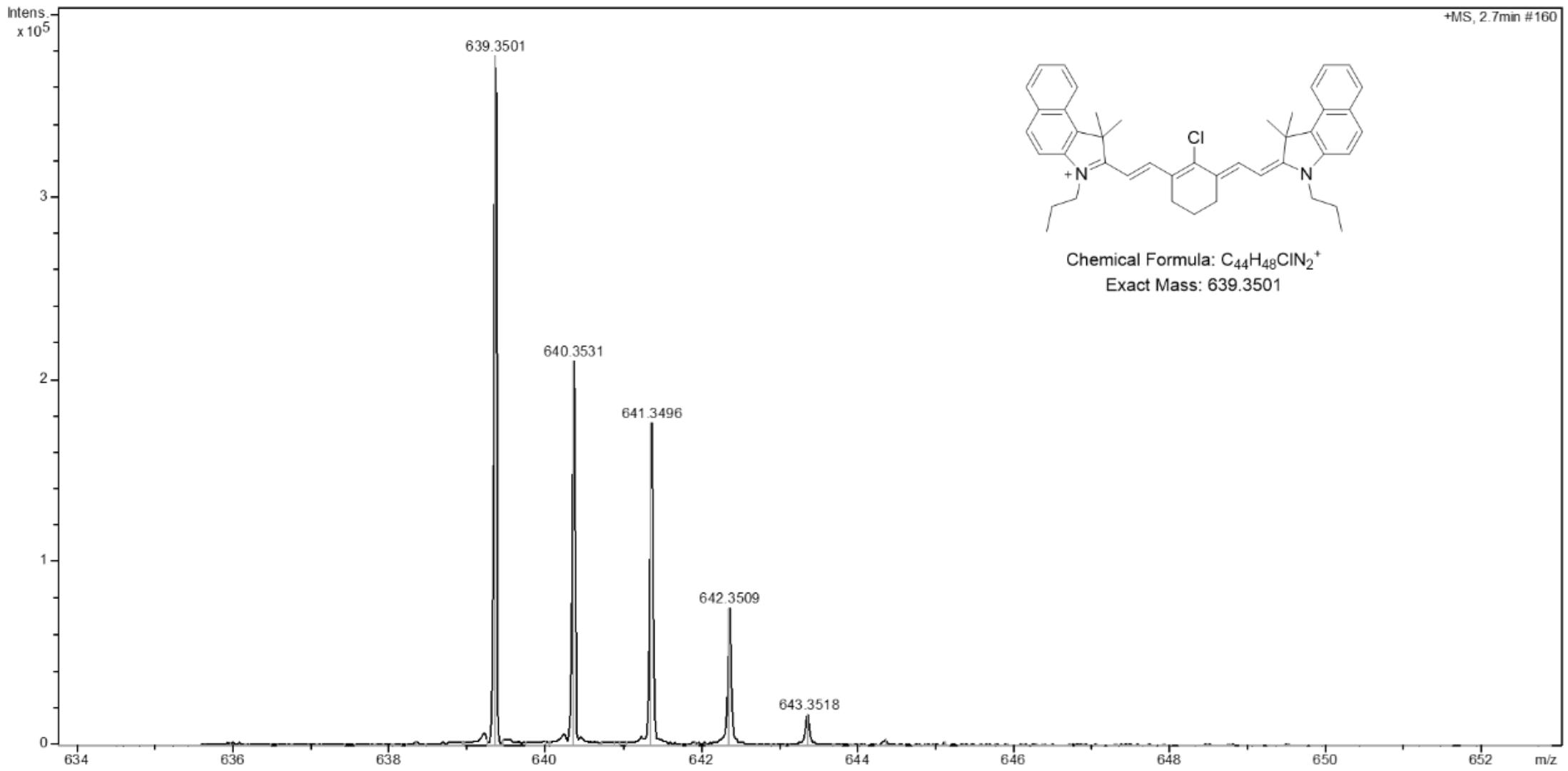

^{1}H-NMR of W3

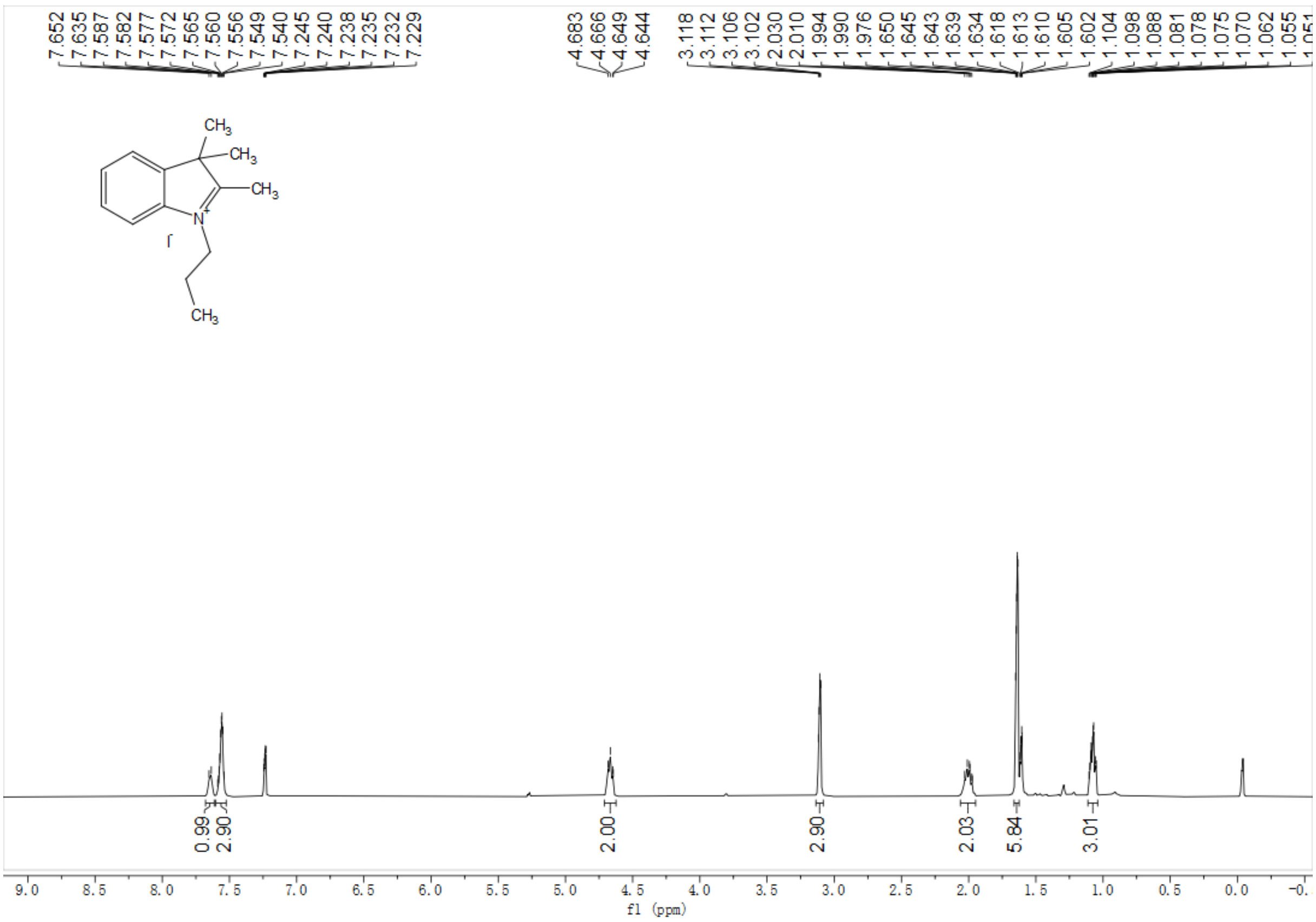

^{1}H-NMR of IR-6B3

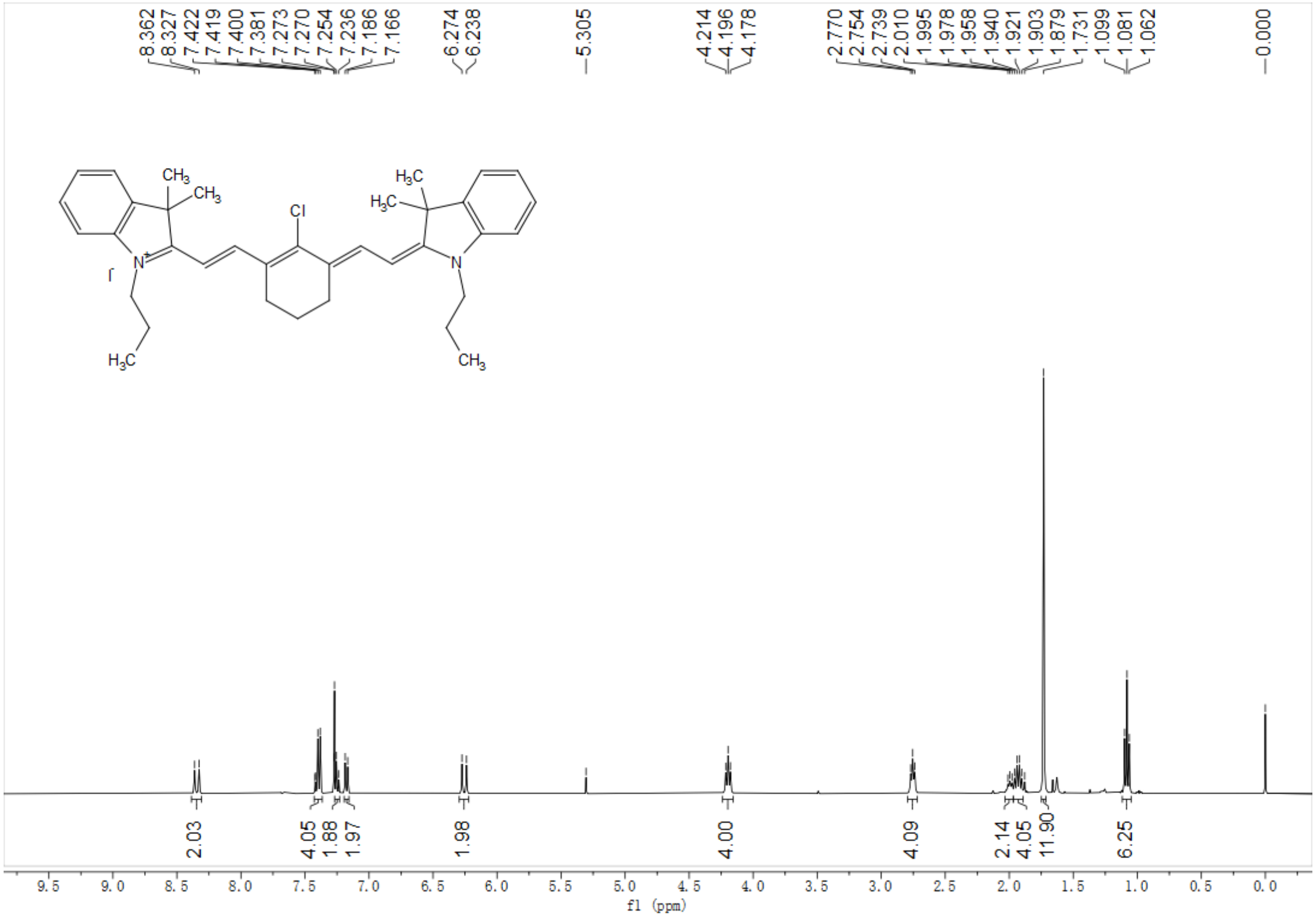

^{13}C-NMR of IR-6B3

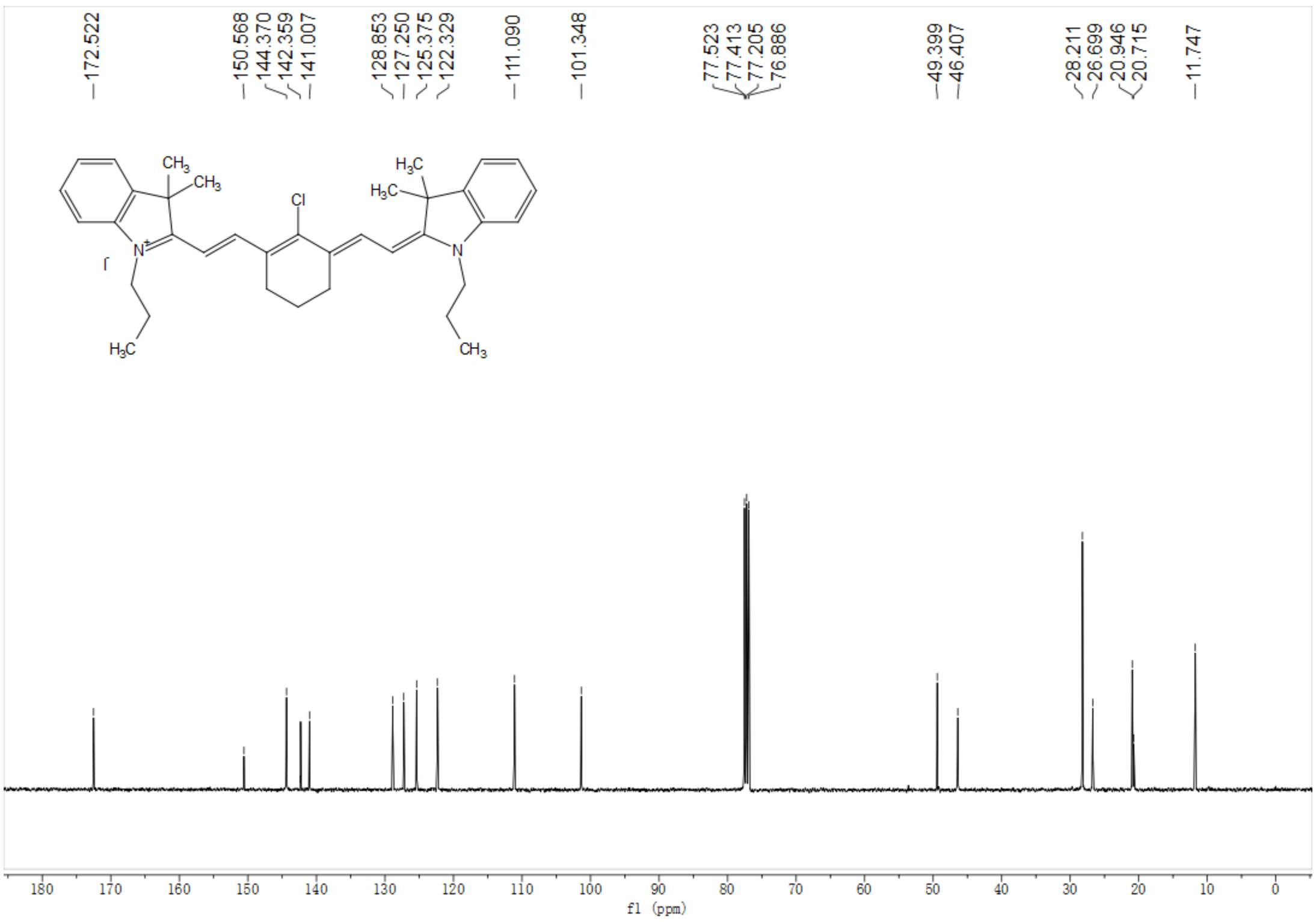

HRMS of IR-6B3

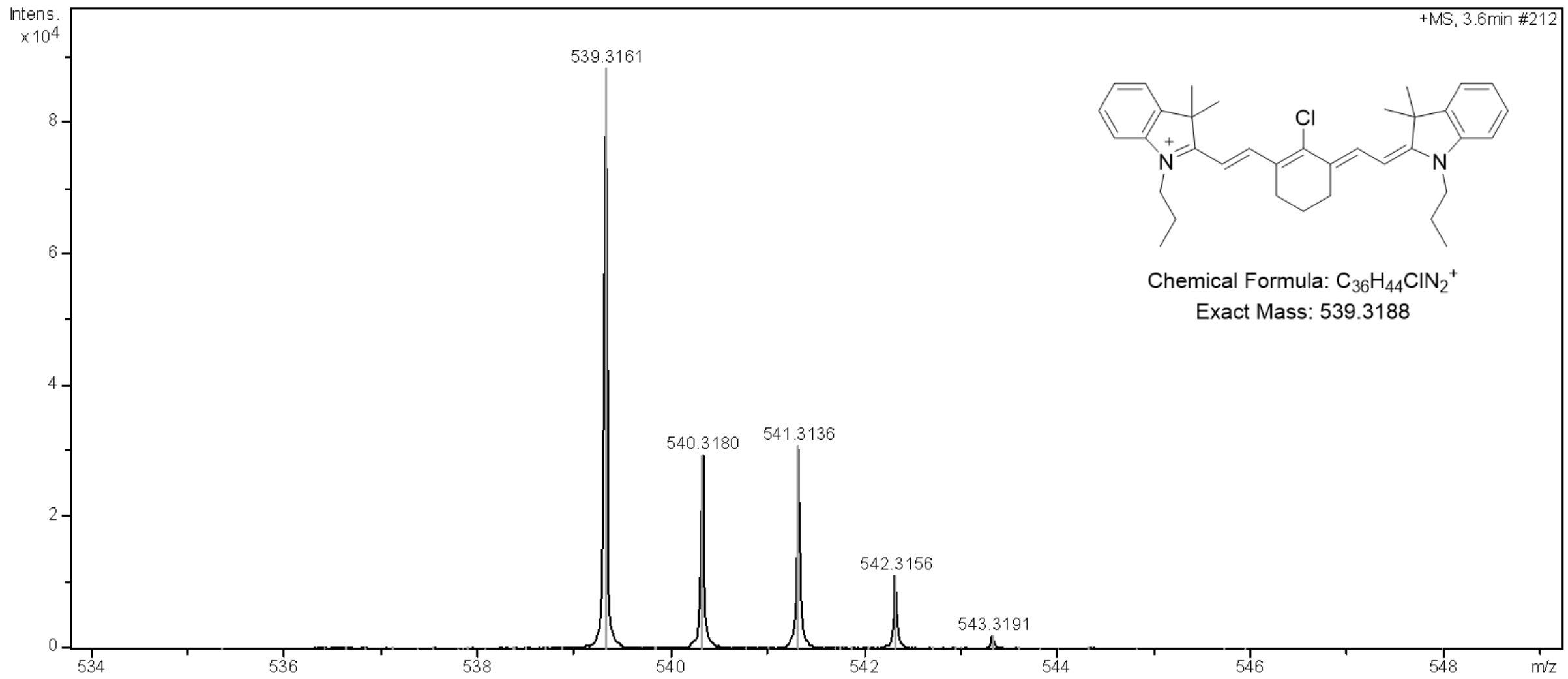

^{1}H-NMR of W4

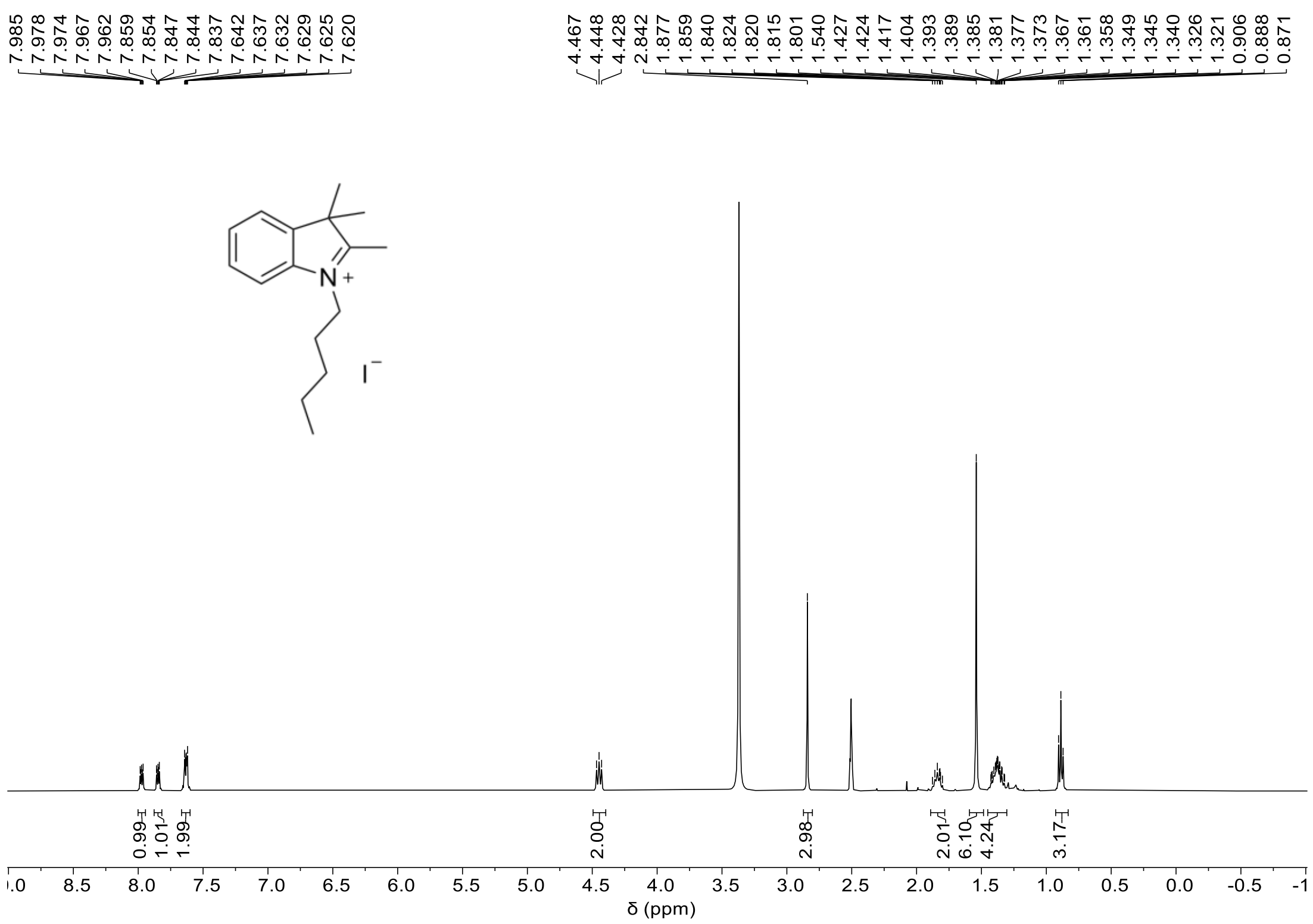

^{1}H-NMR of IR-6B5

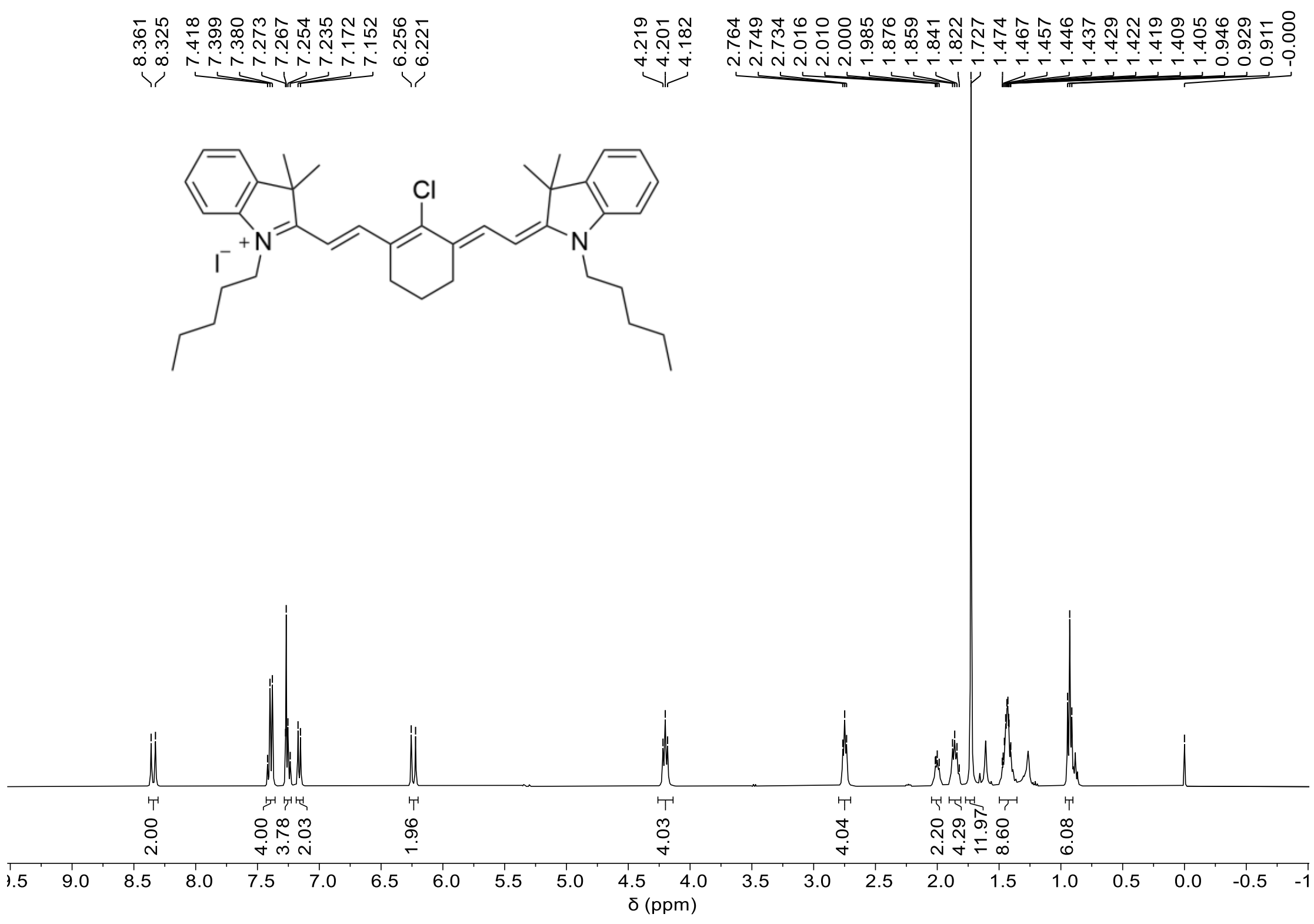

^{13}C-NMR of IR-6B5

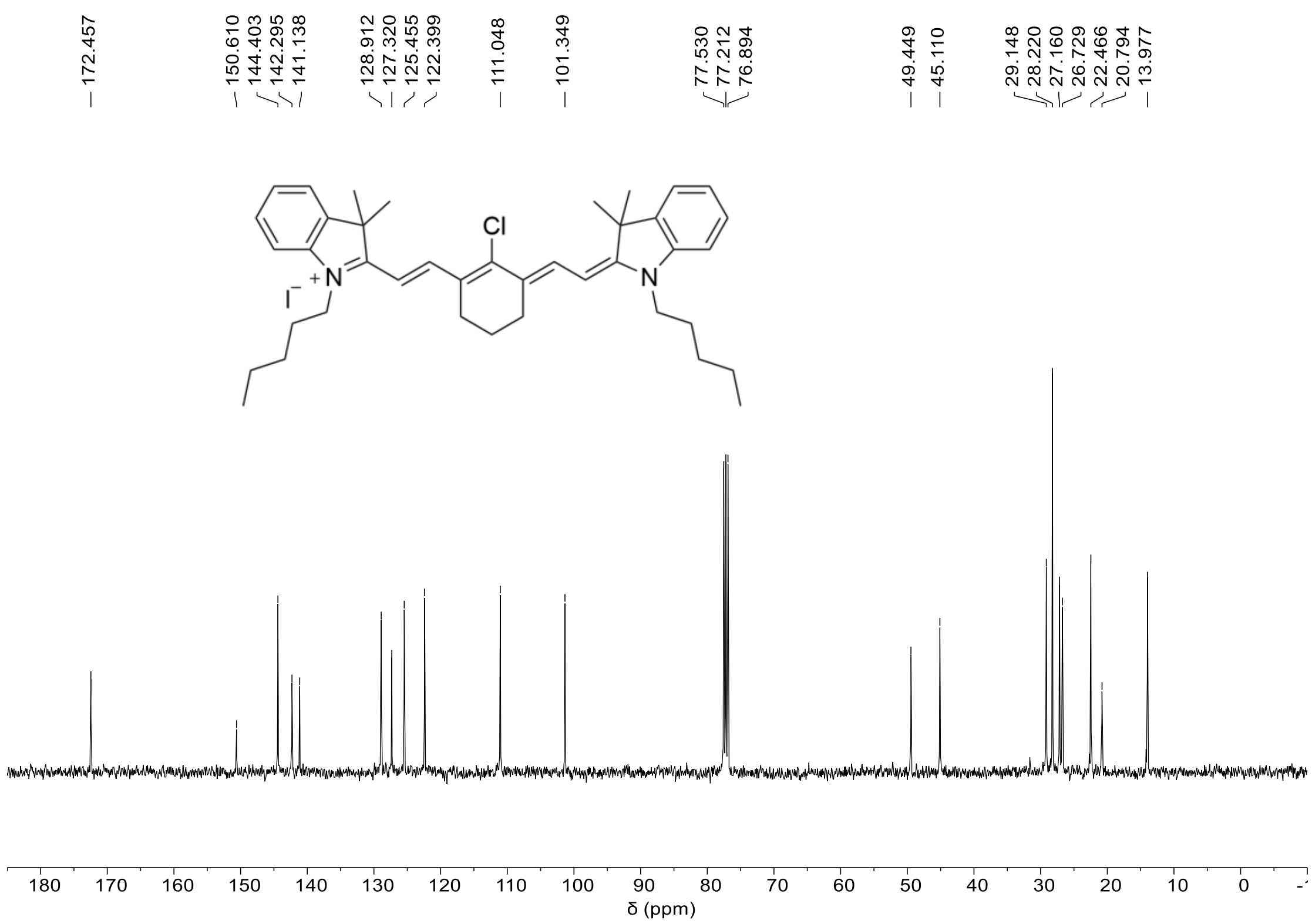

^{1}H-NMR of W5

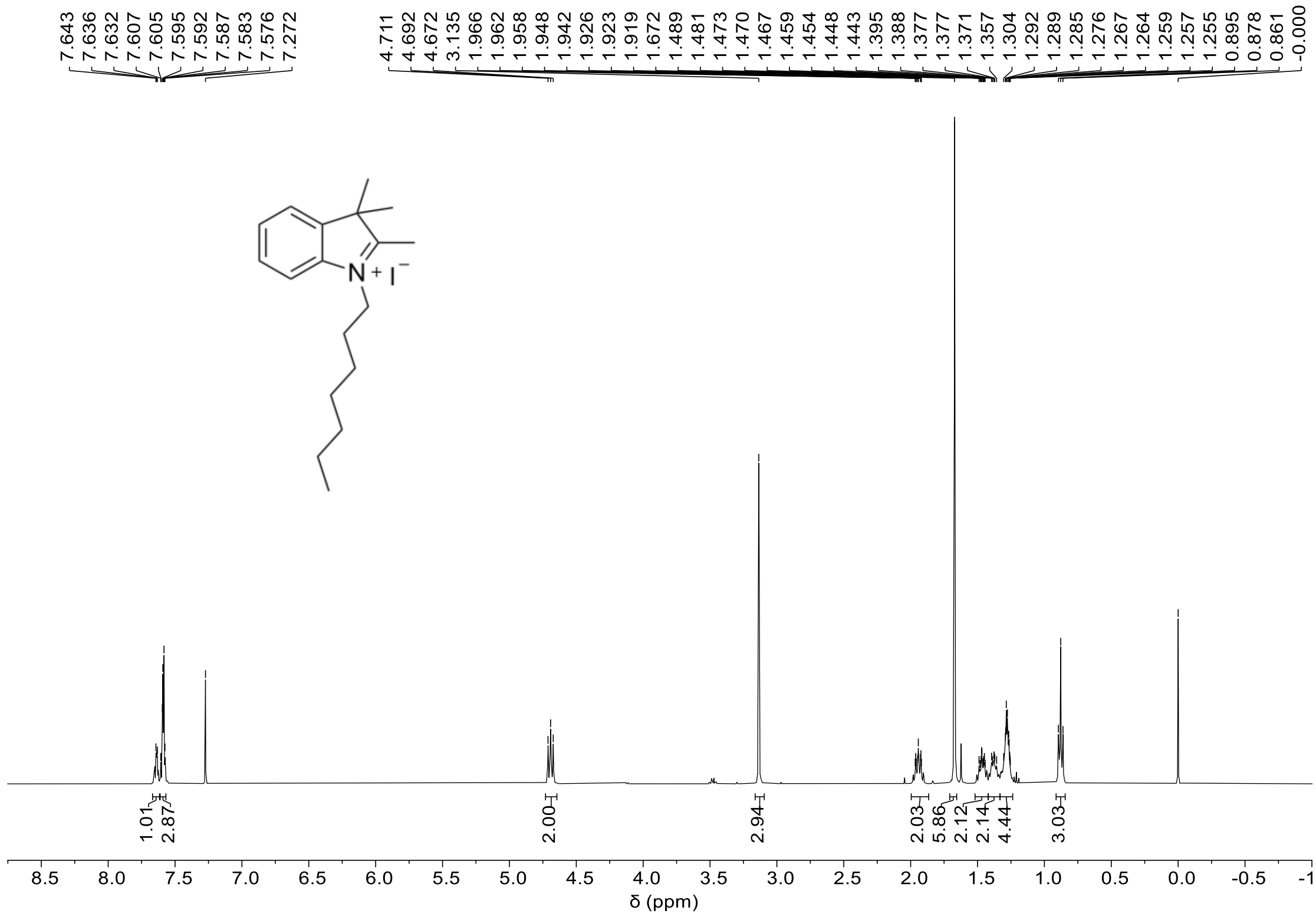

^{1}H-NMR of IR-6B7

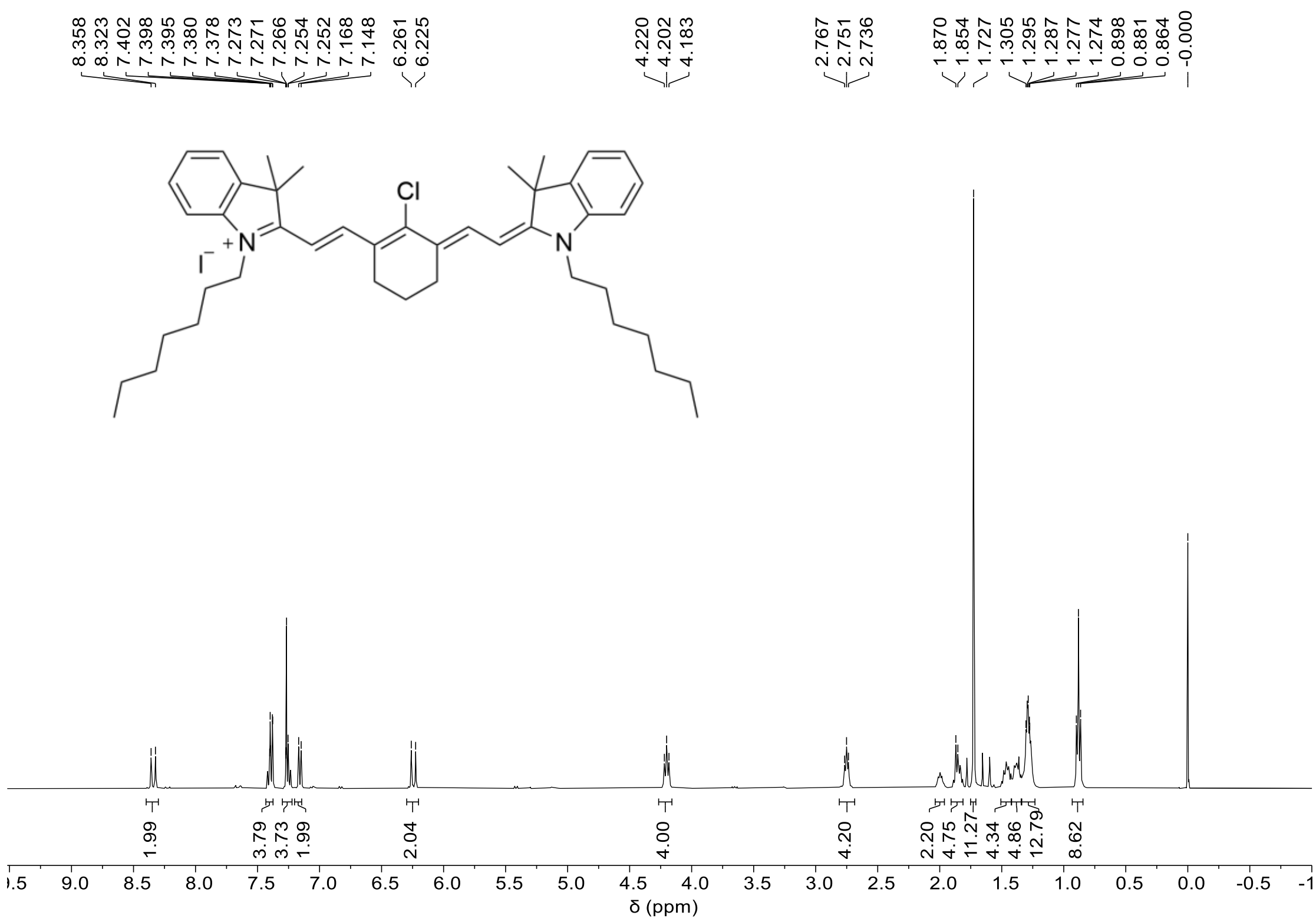

^{13}C-NMR of IR-6B7

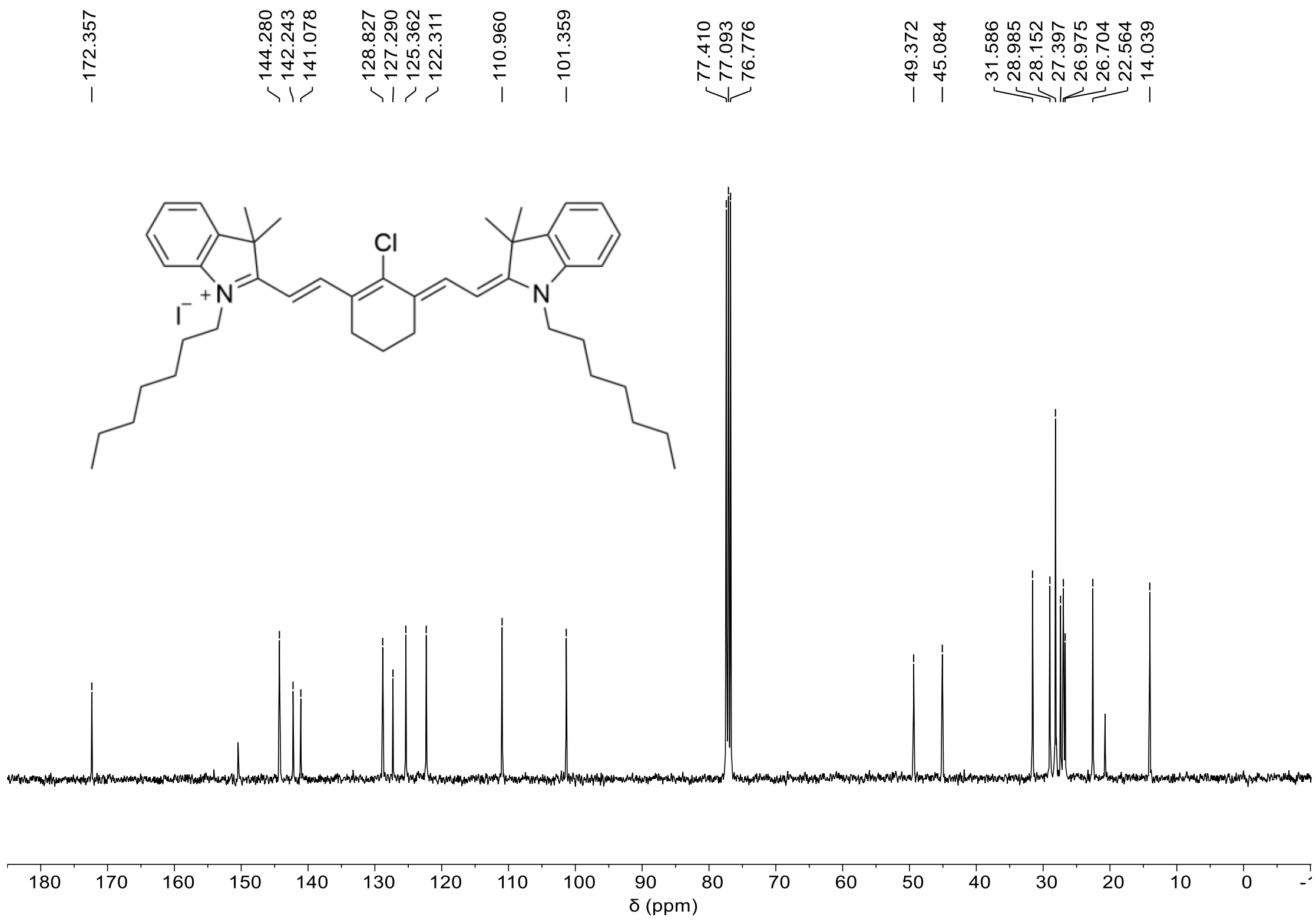

^{1}H-NMR of W6

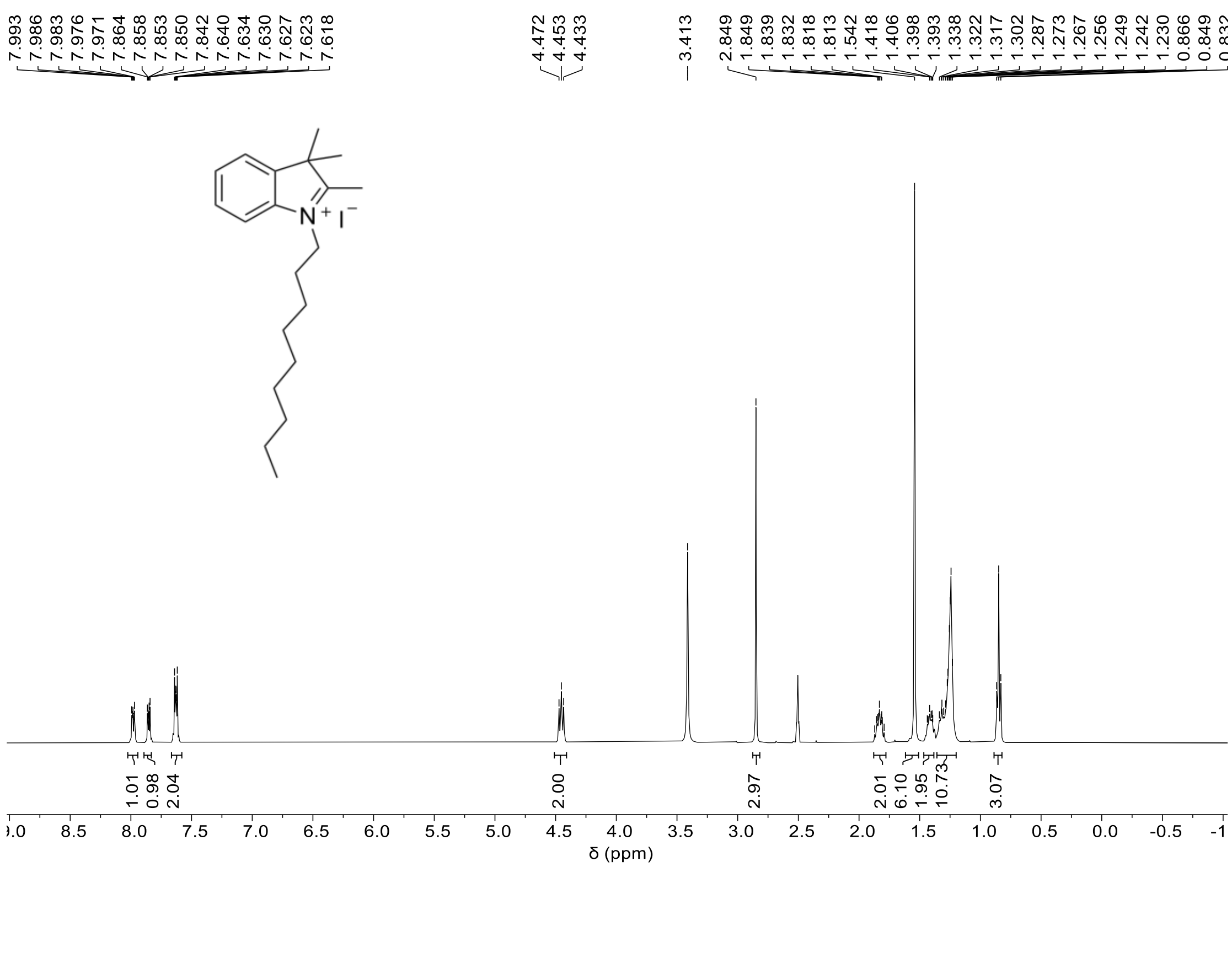

^{1}H-NMR of IR-6B9

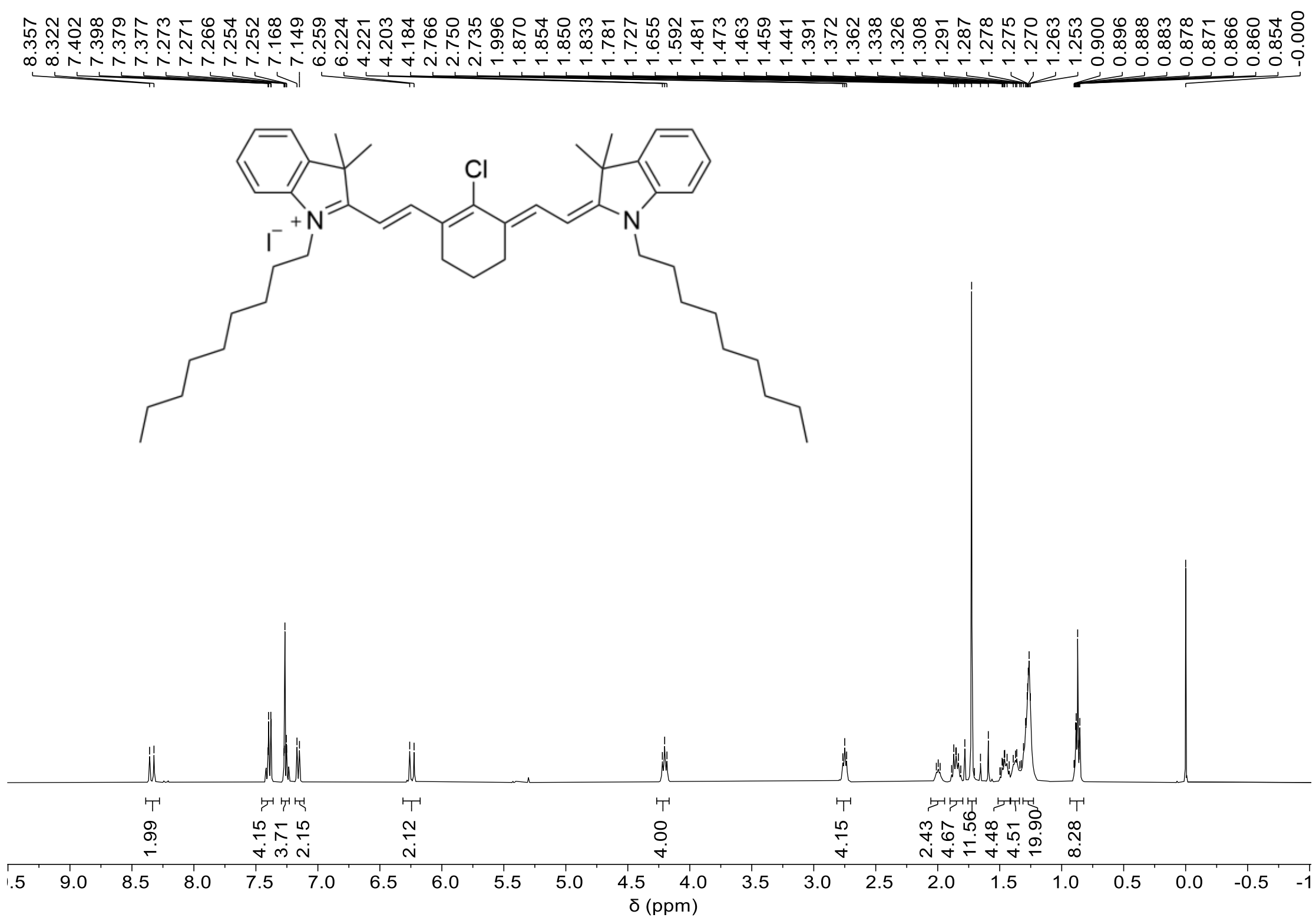

^{13}C-NMR of IR-6B9

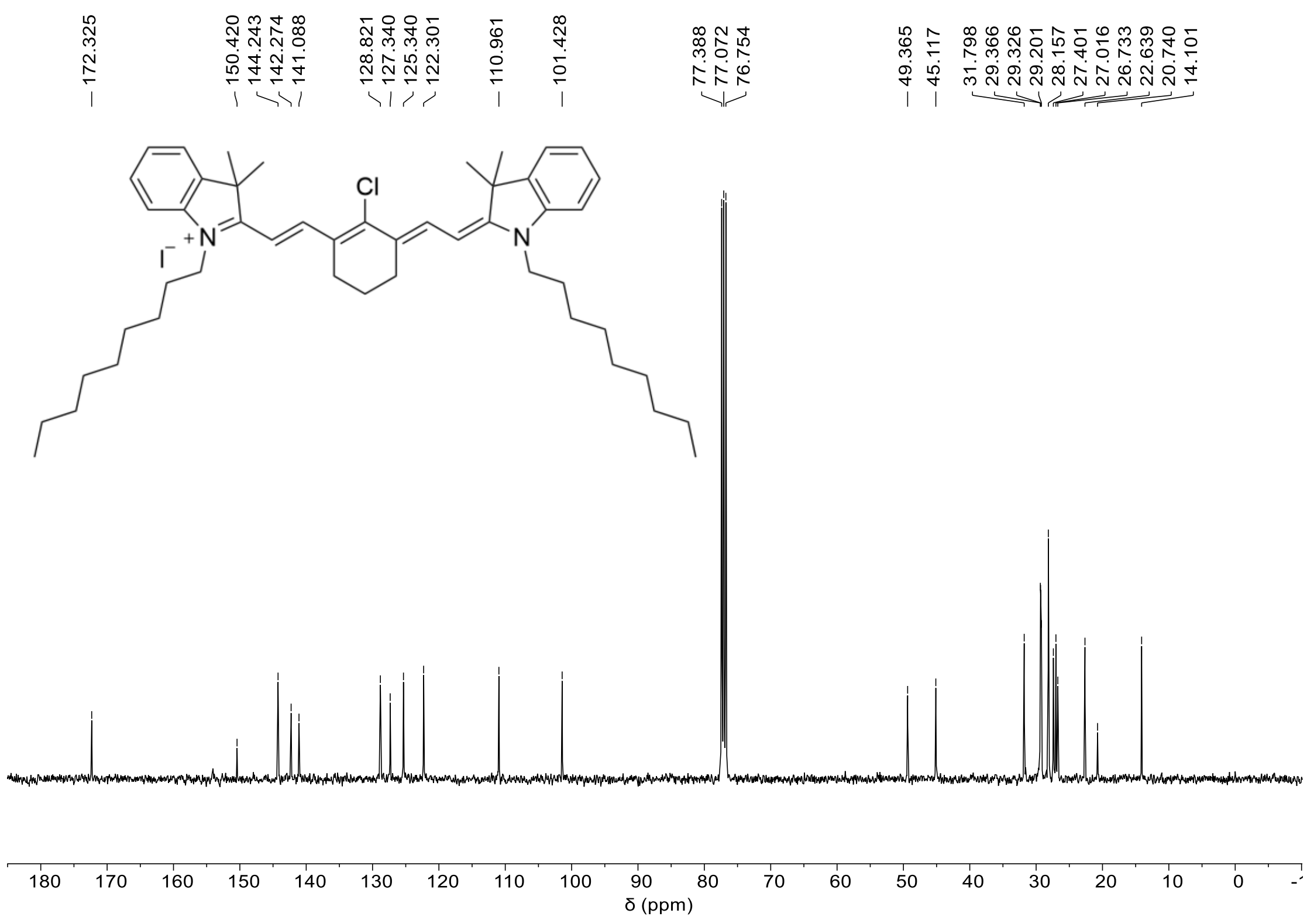

^{1}H-NMR of CETIF6a-GSH

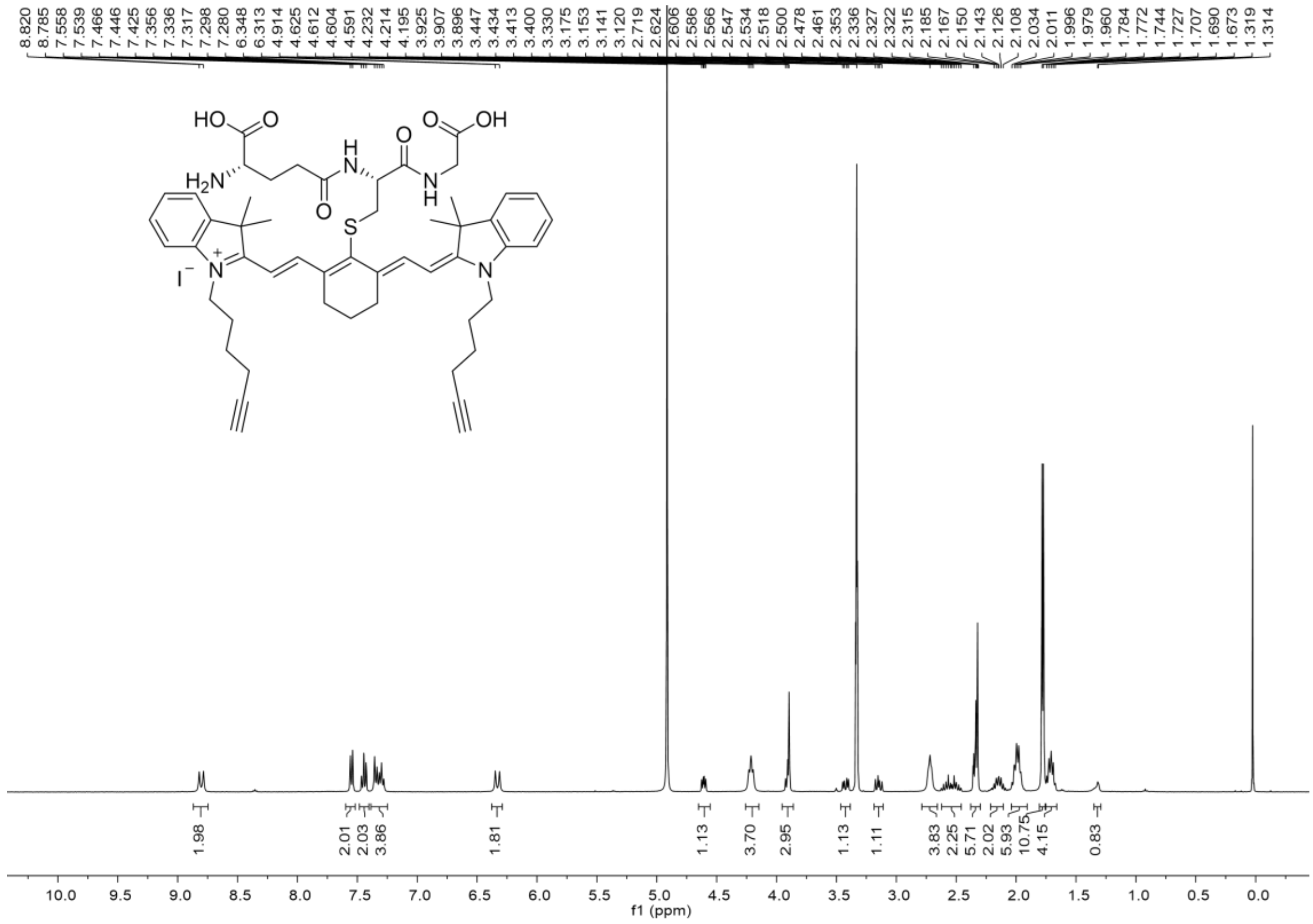

Comments: The submitted manuscript "A NIR fluorotag reporter CETIF6a enables bright pan-tumor labeling and functional proteomic profiling" that reported a click chemistry-compatible heptamethine cyanine fluorotag, CETIF6a, enables pan-cancer targeting, intraoperative histopathology, and proteome-wide target identification. The dual-functional reporter integrating tumor-seeking and activity-based protein profiling identifies hundreds of tumor-enriched targets across multiple cancer types and mechanism analysis, demonstrating that CETIF6a retains intraoperative tumor diagnosis and functional targets profiling. This study fills the gap in the application of cyanine dyes in tumor seeking, which is vitally significant for intraoperative diagnostics, and potentially theranostics. However, several critical issues have to be addressed before publication:

1. Although the authors cited several fluorescent dyes for tumor-seeking, there are missing some recent work, particularly for those imaging-guided detection probes (Angew. Chem. Int. Ed., 2022, 61, e202116807; J. Control. Release, 2024, 376, 961-971.), which should be included.
2. As the author described, CETIF6a exhibited suboptimal pan-cancer labeling efficacy in HCC due to the increased density and a greater diversity and abundance of proteins in the pathological tissues. We would like to determine if any potential non-specific or off-target binding in liver tissue, considering that the heptamethine cyanine dye is readily captured by liver tissue.
3. The azide–alkyne cycloaddition reaction requires Cu(I) catalysis, however, copper ions pose significant physiological toxicity. Please check that probe concentrations used for labeling are within clinically safe ranges, and have any relevant toxicity assessments been performed?
4. How efficient is the reaction within living tissues, and is the applicability to larger solid tumor constrained by penetration depth?
5. For the stage I of the binding, could you clarify whether all hydrophobic pockets can participate in binding? Additionally, is there any binding or modification observed for amino acids incapable of forming thioether bond? Please clarify in detail.

6. The slide staining images of A1 in Figure S2k, are the slides for CETIF4a, 5a, and 6a from the same slide? They appear to be identical. Also, the scale bars need to be added in Figure 1d and S2k.
